# Iron-Catalyzed Friedel–Crafts Reactions of Unactivated 3‑Aryl-Oxetanols Exploiting HFIP Stabilization of Carbocations

**DOI:** 10.1021/acs.joc.6c00762

**Published:** 2026-06-11

**Authors:** Maryne A. J. Dubois, Callum S. Begg, Tsz-Kan Ma, Juan J. Rojas, Andrew J. P. White, Chulho Choi, James A. Bull

**Affiliations:** † Department of Chemistry, Imperial College London, 4615 Molecular Sciences Research Hub, White City Campus, Wood Lane, London W12 0BZ, U.K.; ‡ Medicine Design, Pfizer Worldwide Research, Development and Medical, Eastern Point Rd, Groton, Connecticut 06340, United States

## Abstract

Robust methods to access 3,3-disubstituted oxetanes are desired due to the attractive physicochemical properties offered by the polar 4-membered rings and their potential use as replacement groups for carbonyl or *gem*-dimethyl functionality in medicinal chemistry. The generation of benzylic oxetane carbocations from oxetanols offers considerable potential, particularly in the generation of diaryloxetanes, a structural type that is seldom reported. To date, however, Friedel–Crafts reactions have been limited to oxetanols bearing an electron-rich aromatic group due to the carbocation destabilizing nature of the oxetane. Here, an alternative Fe-catalyzed Friedel–Crafts reaction is described, employing HFIP as a solvent, enabling oxetanyl carbocation formation directly from oxetanols bearing phenyl and other electron-poor arenes. HFIP with its strong hydrogen bond donor (HBD) ability and enhanced Brønsted acidity cooperatively promotes the generation of the carbocations in the presence of the Lewis acid. The oxetane-HFIP adduct was detected, likely formed reversibly as a stabilizing off-cycle intermediate. The developed methodology enabled the concise synthesis of oxetane analogues of Fenofibrate and Tesmilifene, replacing diarylmethane and benzophenone motifs, respectively. Additional mechanistic studies into the fate of these carbocation intermediates provide insights into the reaction mechanism.

## Graphical abstract

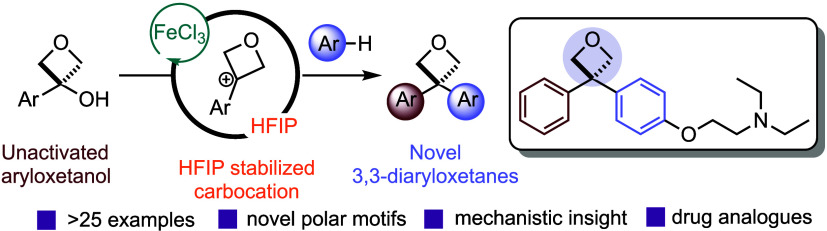

## Introduction

Oxetanes have emerged as valuable design elements for medicinal chemistry due to their small, polar nature and their ability to influence the physiochemical properties of drug compounds.^1^ These features have led to several oxetane-containing compounds entering clinical trials,^2^ and the first fully synthetic oxetane-containing drug received FDA approval very recently.^3^ Particularly relevant are 3,3-disubstituted oxetanes, which can mimic carbonyl functionality and which avoid the introduction of additional stereocenters (Figure 1 for 3,3-disubstituted oxetane clinical candidates). This interest in incorporating the oxetane motif has led to the development of several valuable methods for the preparation of oxetane derivatives.^4–6^

**Figure 1:**
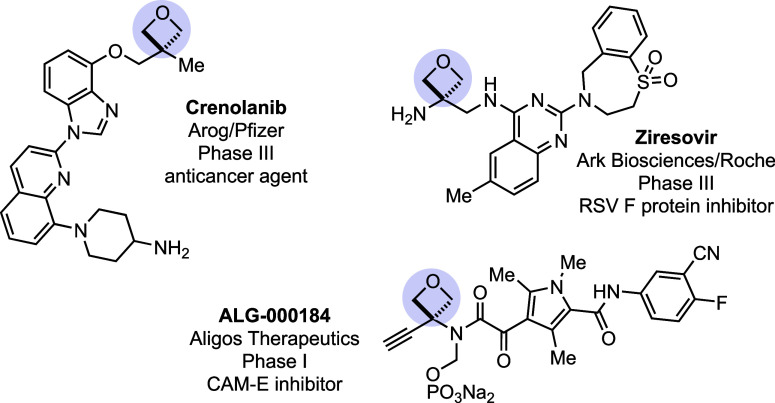
Clinical candidate compounds that contain 3,3-disubstituted oxetanes.

Diaryloxetanes have been surprisingly understudied, yet present interesting structural comparisons to diarylmethanes and benzophenones. There are to date only two synthetic approaches to these substructures. Baran recently reported a multistep sequence involving a Ni-electrocatalytic cross-coupling to form 3 examples of heteroaryl substituted variants (Figure 2A).^7^ In 2016, we demonstrated the first synthesis of 3,3-diaryloxetanes through a Friedel–Crafts approach with selective oxetanol activation, as the first example of benzylic oxetanyl-carbocations.^5a^ This method used catalytic lithium triflimide, but necessitated electron-rich aromatic derivatives to provide resonance stabilization, as well as phenol nucleophiles.^5a^ Reactions with phenols were observed to proceed through the *O*-linked phenol ether, an off-cycle intermediate that provided a stabilizing carbocation reservoir, and which would convert to the Friedel–Crafts product by regeneration of the carbocation.^8^

**Figure 2:**
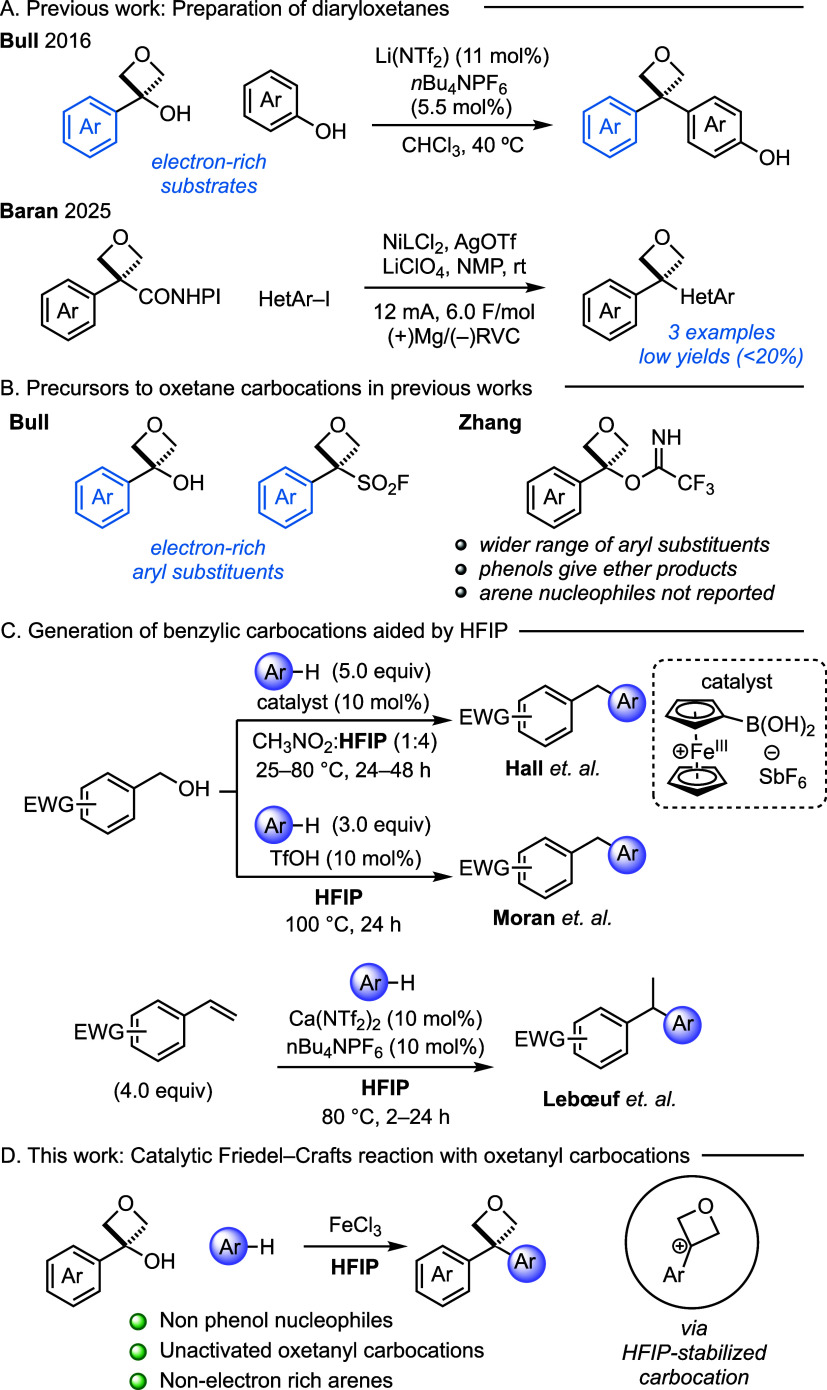
(A) Previous approaches to diaryloxetanes. (B) Previous oxetane carbocation precursors and limitations. (C) Previous Friedel–Crafts reactions and hydroarylation with deactivated benzylic alcohols and styrenes, enabled by HFIP. (D) This work: the HFIP promoted Friedel–Crafts alkylation of nonelectron-rich 3-aryloxetan-3-ols and nonphenolic arene nucleophiles.

Subsequent advances in oxetane carbocation derivatives have exploited alternative leaving groups (Figure 2B).^4b,4c^ Zhang’s trichloroacetimidate approach is notable in that less electron-rich aromatic groups were used to stabilize the carbocation intermediate, being successful for phenyl groups, for example.^4b^ While requiring an additional activation step, the use of the trichloroacetimidate allowed selective activation by preferential coordination of a copper Lewis acid to this leaving group over the oxetane or nucleophile. Friedel–Crafts reactivity was not observed, however, and phenols gave ether products, which were presumably not prone to further activation under the conditions. Direct activation of oxetanols bearing less electron-rich arenes remains unrealized and would provide a valuable addition to the preparation of these compound classes.

HFIP exhibits unique solvent properties due to its ability to aggregate as dimers or trimers, which simultaneously enhance both its hydrogen bond donor (HBD) ability and increase its Brønsted acidity (p*K*_a_ = 9.3).^9^ These strong HBD properties and dielectric constant (ε = 15.7) have been shown to accelerate a variety of chemical reactions.^9,10^ In 2012, Nájera reported the first instance of alcohol activation using TFE and HFIP, in a metal-free substitution of allylic alcohols with amines and carbamates.^11^ Subsequently, Hall and Moran independently developed Friedel–Crafts conditions for the preparation of diarylmethanes from highly electronically deactivated benzylic alcohols, catalyzed by boronic acid and TfOH respectively and enabled by HFIP (Figure 2C).^12^ Leboeuf reported the use of calcium triflimide as a catalyst in HFIP for hydroarylation of styrenes.^13^ The utility of HFIP has since been further demonstrated in the Friedel–Crafts arylation of other challenging substrates, highlighting the potential of polyfluorinated solvents to enable novel reactivities.^14^

Encouraged by the potential of HFIP to stabilize carbocation intermediates, we hypothesized that it could be employed to facilitate Friedel–Crafts reactions with less electron-rich 3-aryl-3-oxetanols and to extend the range of successful arene nucleophiles. Consequently, we would expand the accessible chemical space of diaryloxetanes. However, oxetanes have been shown to polymerize or ring-open under acidic conditions.^13^ The success of this strategy would be reliant on the stability of the oxetane, especially given the strong hydrogen bonding of HFIP with ethers and the increased acidity of the solvent O–H group in the presence of a Lewis acid.^15^

Here we report the development of an iron chloride-catalyzed Friedel–Crafts reaction of aryloxetanols. The use of HFIP as solvent was crucial to enable the productive reaction with nonelectron-rich arene substituents and nonphenolic arene nucleophiles, while minimizing unproductive oxetane ring-opening processes (Figure 2D). We demonstrate the utility of this new methodology by synthesizing oxetane-containing drug analogues of tesmilifene and fenofibrate. Moreover, we examined side products and intermediates to explain the overall reactivity profile of the oxetane derivatives under the reaction conditions.

## Results and Discussion

Initially we investigated the Friedel–Crafts reaction of unactivated 3-phenyloxetan-3-ol (**1**) with 2,6-dimethylphenol, chosen to prevent dihydrobenzofuran formation.^16^ Early attempts using HFIP solvent did indeed give the desired product in low yields, and led to very low overall recovery of oxetane. Considerable optimization of conditions was necessary to prevent degradation of the oxetane ring. Various Brønsted and Lewis acids were tested to modulate acidity and improve mass recovery employing 4 equiv of nucleophile. Brønsted acids such as TFA, TfOH, and Tf_2_NH were investigated at 80 °C (Table 1, entries 1–3), but none promoted the reaction efficiently, leading to recovered starting material or degradation. A combination of Ca­(NTf_2_)_2_/*n*Bu_4_NPF_6_ in HFIP gave 17% yield (Table 1, entry 4).^5a,13,17^ The use of lithium triflimide increased the yield of oxetane **2** to 38%, albeit with similar loss of material (entry 5). The combination of LiCl and HFIP, reported by Zhu in the Friedel–Crafts alkylation of phenol with ethyl glyoxylate, was unreactive,^14^ and F_5_C_6_B­(OH)_2_ ^18^ gave recovered starting material (See SI, Table S1 for further details of catalyst screening).

**Table 1:**
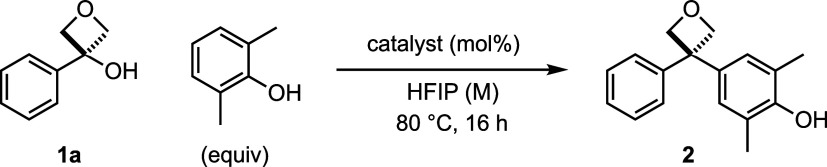
Initial Catalyst Screen, Effect of the Catalyst Loading, Concentration, Temperature, and Nucleophile Equivalents on the Friedel–Crafts Alkylation of 2,6-Dimethylphenol with **1a**

entry	catalyst	cat. loading (mol %)	nuc. equiv	conc (M)	RSM 1a (%)^a^	yield of 2 (%)^a^
1	Tf_2_NH	10	4	0.2	0	1
2	TFA	10	4	0.2	91	5
3	TfOH	10	4	0.2	0	21
4	Ca(NTf_2_)_2_/*n*Bu_4_NPF_6_	10	4	0.2	0	17
5^b^	Li(NTf_2_)_2_/*n*Bu_4_NPF_6_	11	4	0.2	0	38
6	FeCl_3_	10	4	0.2	25	53
7	Fe(acac)_3_	10	4	0.2	100	0
8	FeCl_2_	10	4	0.2	98	0
9	Fe(OTf)_2_	10	4	0.2	0	20
10	FeCl_3_	10	4	0.1	0	73
11^c^	FeCl_3_	10	1.7	0.1	0	94
12	no cat.	0	4	0.2	100	0

a Yields were calculated by ^1^H NMR spectroscopy using 1,3,5-trimethoxybenzene as the internal standard.

b Li­(NTf_2_) (11 mol %) and *n*Bu_4_NPF_6_ (5.5 mol %) were used.

c If the reaction is performed at 60 °C, 52% yield is obtained.

The use of FeCl_3_ markedly improved the transformation, affording oxetane **2** in 53% yield with 25% of starting material recovered (Table 1, entry 6), and was selected as the optimal catalyst owing to its efficiency, low cost, and earth abundance. Additional iron catalysts (Table 1, entries 6–9) including Fe­(acac)_3_ and FeCl_2_ were unreactive, while Fe­(OTf)_2_ gave only low conversion. Catalyst loading, concentration, and temperature strongly influenced the reaction outcome. Lowering the concentration (0.1 M) increased the yield to 73% (Table 1, entry 10). Reducing the temperature or increasing catalyst loading suppressed reactivity (See SI for details). The equivalents of nucleophile were crucial, where unlike typical Friedel–Crafts processes, excess phenol was detrimental to the reaction outcome. Reducing nucleophile equivalents to 1.7 provided the optimal conditions, yielding diaryloxetane **2** in 94% yield (Table 1, entry 11). The reaction was not sensitive to air and moisture, and HFIP was essential as a solvent, outperforming trifluoroethanol (TFE) and mixed solvent systems using HFIP in various proportions (See SI for further details).^19^

Extending the nucleophile classes revealed differing reactivity when using *p*-xylene and anisole as nucleophiles (Table 2). Both nucleophiles were successful in the Friedel–Crafts addition to oxetanol **1a**, affording diaryloxetanes **3** and **4**, but aldehyde side products **3b** and **4b** were also isolated. Increasing equivalents of *p*-xylene nucleophile up to 5 improved the yield of oxetane **3** to 55% (Table 2, entries 1–2). However, using *p*-xylene in large excess (as cosolvent, ∼ 32 equiv) adversely affected the reaction, reducing the yield to 40%, presumably due to reducing the HFIP stabilization. Aldehyde formation increased markedly when a larger quantity of anisole was employed as nucleophile (Table 2, entries 3,4) and a lower excess was preferable.

**Table 2:**
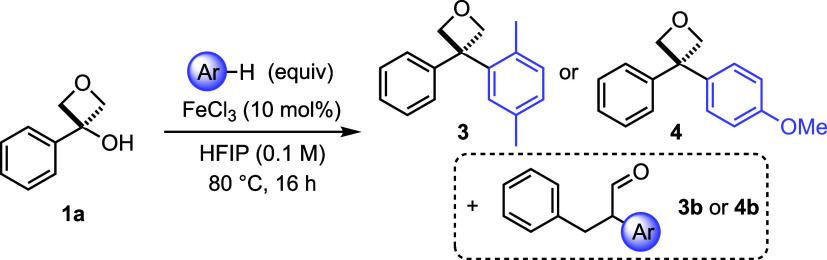
Effect of Nucleophile Equivalents Using Anisole and *p*-Xylene

entry	nucleophile	equivalents	yield (%)^a^ 3 or 4	yield (%)^a^ aldehydes 3b or 4b
1	*p*-xylene	1.7	44	13
2	*p*-xylene	5.0	55	9
3	anisole	1.7	35	11
4	anisole	3.0	38	33

a Yields calculated by ^1^H NMR spectroscopy using 1,3,5-trimethoxybenzene as the internal standard.

With two sets of conditions in hand, for the more and less reactive nucleophiles, the scope of the reaction was evaluated by varying both the nucleophile and oxetanol components (Scheme 1). Using phenyl oxetanol **1a**, *p*-xylene gave diaryloxetane **3** in 58% yield, while mesitylene and durene afforded oxetanes **5** and **6** in 73 and 71% yield, respectively. *p*-Cymene gave diaryloxetane **7** in 42% yield, with the reaction occurring regioselectively at the position adjacent to the methyl group to minimize steric hindrance (rr 92:8). Benzene and toluene gave 3,3-diaryloxetanes **8** and **9** in low yields. Attempts to incorporate *N*-methylindole or furan were unsuccessful. Notably, two different classes of aldehyde side products were observed in low quantities (see the SI for further discussion).^20^

**Scheme 1:**
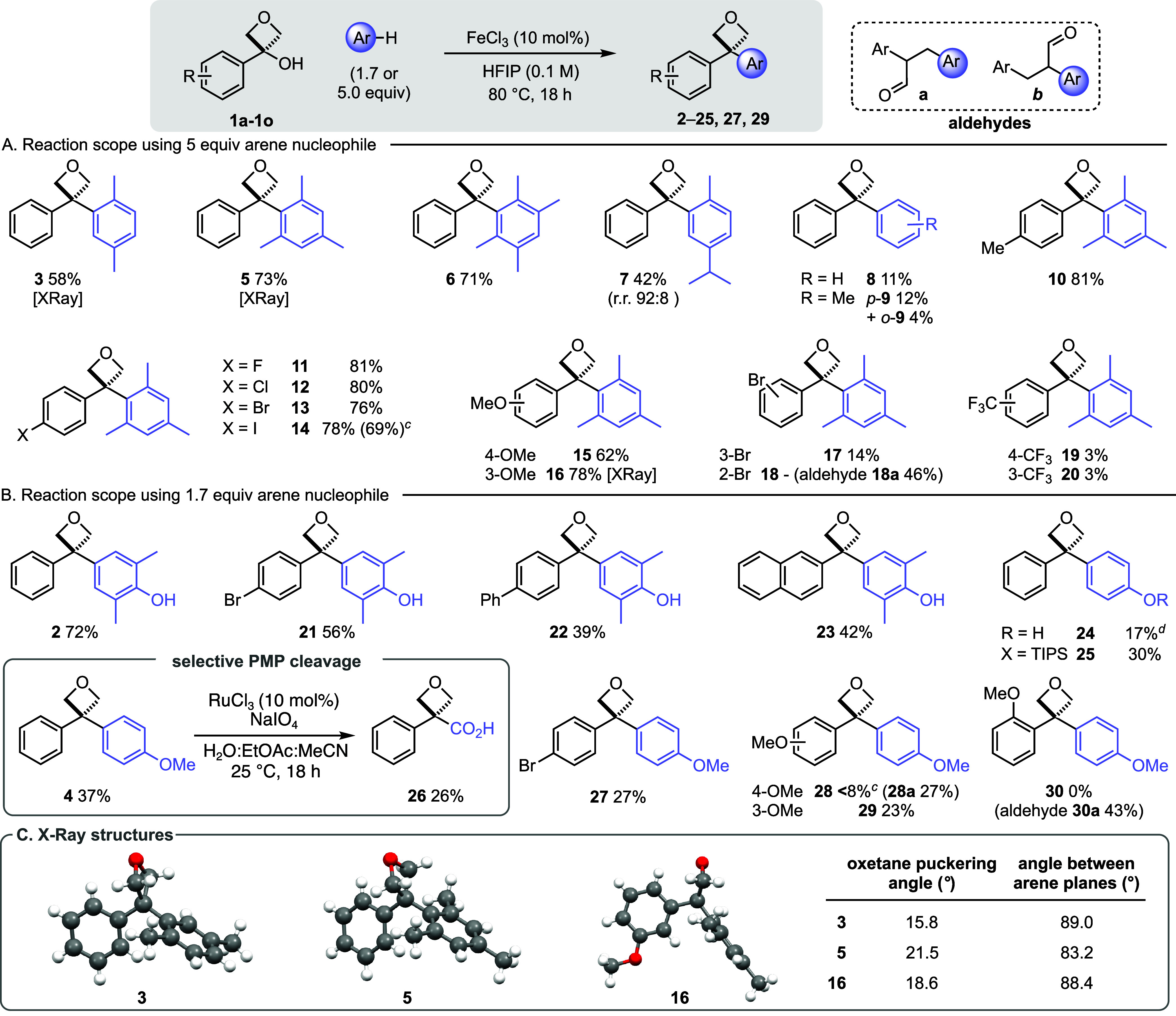
Catalytic Synthesis of Diaryloxetanes: Substrate Scope Varying Nucleophile and Oxetanol[Fn s1fn5]

A. Using 5 equiv of nonoxygenated nucleophile. B. Using 1.7 equiv of oxygenated nucleophiles. C. Single crystal X-ray structures and analysis.
b The reaction was performed on a 4.0 mmol scale.
c A ketone side product was also observed. See the SI for further details.
d Yields were calculated by ^1^H NMR spectroscopy using 1,3,5-trimethoxybenzene as an internal standard.
a Reactions were performed on a 0.50 mmol scale unless stated otherwise. Isolated yields were reported unless stated.

On varying the oxetanol, the tolyl substituent gave comparable yields with mesitylene (81% **10**). Halide substituents all reacted successfully, providing halogenated diaryloxetanes **11**–**14** in 76–81% yields. These halide-containing oxetane products offer valuable synthetic handles and such products were not accessible using previous methodologies. The reaction scaled efficiently to 4.0 mmol, producing 1 g of iodophenyl oxetane **14** (69% yield). Substrates with a methoxy group at the *meta*- and *para*-positions were both successfully reacted with mesitylene (**15**, **16**). By comparison, while 4-methoxyphenyloxetanol was successful in our previous reports with phenol nucleophiles, it was unreactive with mesitylene, and 3-methoxyphenyl was previously unreactive to Friedel–Crafts reactions in general, highlighting the increased reactivity of the HFIP system. The 3-bromophenyl substrate formed diaryloxetane **17** in low yield (17%). However, the 2-bromo derivative did not form the desired product, forming aldehyde products in 46% yield. Notably, the *o*-tolyl oxetanol led to the formation of aldehyde product exclusively, with no formation of the target oxetane (see Supporting Information). Deactivating trifluoromethylphenyl derivatives were essentially unreactive (**19**, **20**), and the oxetanol substrate was recovered.^21,22^

Reacting oxetanol **1a** with 2,6-dimethylphenol (1.7 equiv) on a 0.5 mmol scale afforded the corresponding 3,3-diaryloxetane **2** in 72% isolated yield (Scheme 1B). *p*-Bromophenyl afforded oxetane **21** in 56% yield. Biphenyl and naphthyl substituents were also tolerated, giving oxetanes **22** and **23** in 39 and 42% yields. By contrast, phenol itself gave a complex mixture with only 17% of oxetane **24** (^1^H NMR yield), alongside dihydrobenzofuran and unidentified side products, with no recovered starting material. TIPS-protected phenol (using 5 equiv of nucleophile) gave **25** in 30% isolated yield. Using anisole as the nucleophile yielded oxetane **4** in 37% yield, isolated exclusively as the *para*-substituted isomer. The 4-methoxyphenyl group could be selectively cleaved to yield the corresponding oxetane carboxylic acid **26**.^23^ With anisole nucleophile, 4-bromophenyl oxetanol gave **27** in 27% yield with full conversion of starting material. 4- and 3-methoxyphenyloxetanols gave low product yields and significant aldehyde formation **28a** (27%) and **30a** (43%; see SI for further discussion). *o*-Methoxyphenyloxetanol also led to the exclusive formation of aldehyde products.

Several of the diaryloxetanes were further characterized by single crystal X-ray diffraction (SCXRD, Scheme 1C). Analysis of **3**, **5**, and **16** revealed that each compound adopts a near-perpendicular orientation of the arene planes (83.2–89.0°) and pronounced oxetane ring puckering (15.8–21.5°). This dihedral twist feature is reminiscent of conformations typically observed in sterically hindered biaryl systems.

To demonstrate the utility of the method to provide rapid routes to potentially valuable oxetane compounds, two oxetane analogues of drug compounds were targeted (Scheme 2A). Tesmilifene is an antineoplastic agent bearing a diarylmethane.^24^ Such diarylmethanes can be metabolically vulnerable, and oxetanes may provide a valuable role in blocking sites of oxidative metabolism.^1^ The HFIP-mediated Friedel–Crafts conditions were applied to the reaction of oxetanol **1a** with commercial β–bromophenetole (2-bromoethyl phenyl ether). This nucleophile was compatible with the reaction conditions in the presence of the primary bromide, giving 3,3-diaryloxetane **31** in 39% yield.^25^ Nucleophilic substitution of bromide **31** with HNEt_2_ provided oxetano-tesmilifene **32**. Fenofibrate is used for the treatment of hypercholesterolemia, and contains a benzophenone structure that may be mimicked by the oxetane as an isostere.^26^ Attempts to react oxetanol **1d** with phenol itself were unsuccessful, but the TIPS ether of phenol successfully gave oxetane **33**, which was readily deprotected with TBAF to give phenol **34**. Nucleophilic substitution with isopropyl 2-bromo-2-methylpropanoate gave oxetano-fenofibrate **35**.

**Scheme 2:**
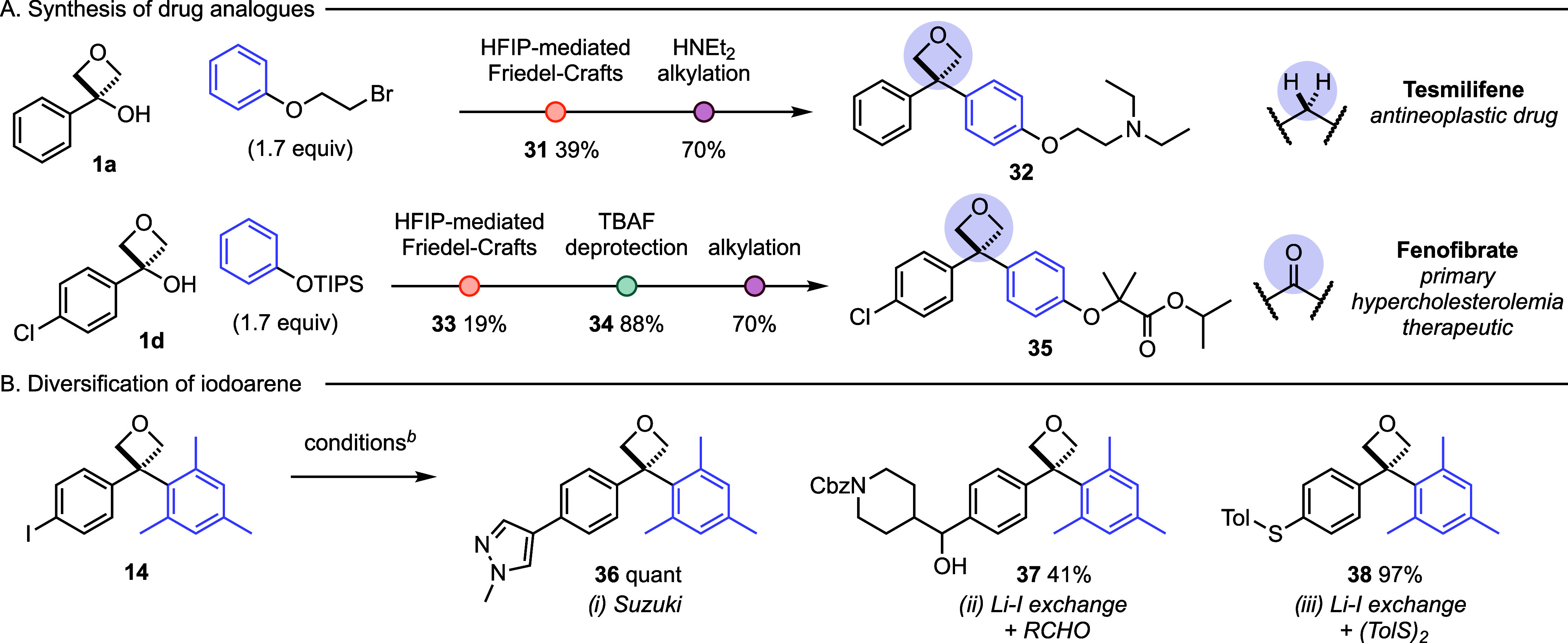
(A) Synthesis of Oxetane-Containing Fenofibrate and Tesmilifene Analogues. (B) Diversification of Iodoarene 14^b^

a Isolated Yields.
b Reaction conditions: (i) 1-methylpyrazole-4-boronic acid pinacol ester, Pd­(dppf)­Cl_2_, K_2_CO_3_, dioxane/H_2_O (2:1), 100 °C, 16 h, quant.; (ii) *n*-BuLi, benzyl 4-formylpiperidine-1-carboxylate, THF, – 78 °C, 1 h, 41%; (ii) *n*-BuLi, *p*-tolyl disulfide, THF, – 78 °C, 1 h, 97%.

In addition, a range of diversification reactions was readily applied with iodoarene **14** (Scheme 2B). A palladium-catalyzed Suzuki-Miyaura cross-coupling reaction proceeded to give pyrazole **36** in quantitative yield. The organolithium reagents derived from iodoarene **14** and *n*-BuLi readily reacted with a piperidine carboxaldehyde and a disulfide to give the corresponding alcohol **37** and thioether **38** products in 41 and 97% yield, respectively.

Next, studies were undertaken to better understand the reaction mechanism. Oxetanol **1a** was subjected to the optimized reaction conditions in the absence of nucleophile and was examined using ^1^H NMR spectroscopy. After 16 h, 21% of oxetanol **1a** was recovered along with 18% of the other two products, which could not be isolated due to their instability. One of these products was identified as ether **40**, presenting two doublets for oxetane peaks (in orange, Figure 3A) at δ_H_ = 5.10 ppm integrating for four protons and one heptet at δ_H_ = 4.3 ppm corresponding to a CH adjacent to the CF_3_ groups. Ether **40** results from the addition of HFIP to the oxetanyl carbocation intermediate. We postulate that this type of intermediate may provide a general reason for the observed HFIP carbocation-stabilizing effect, providing a stabilizing carbocation reservoir that can rerelease the carbocation under the reaction conditions. i.e., suggesting the HFIP performs a similar stabilizing role to that seen with phenols in our previous work.^8^

**Figure 3:**
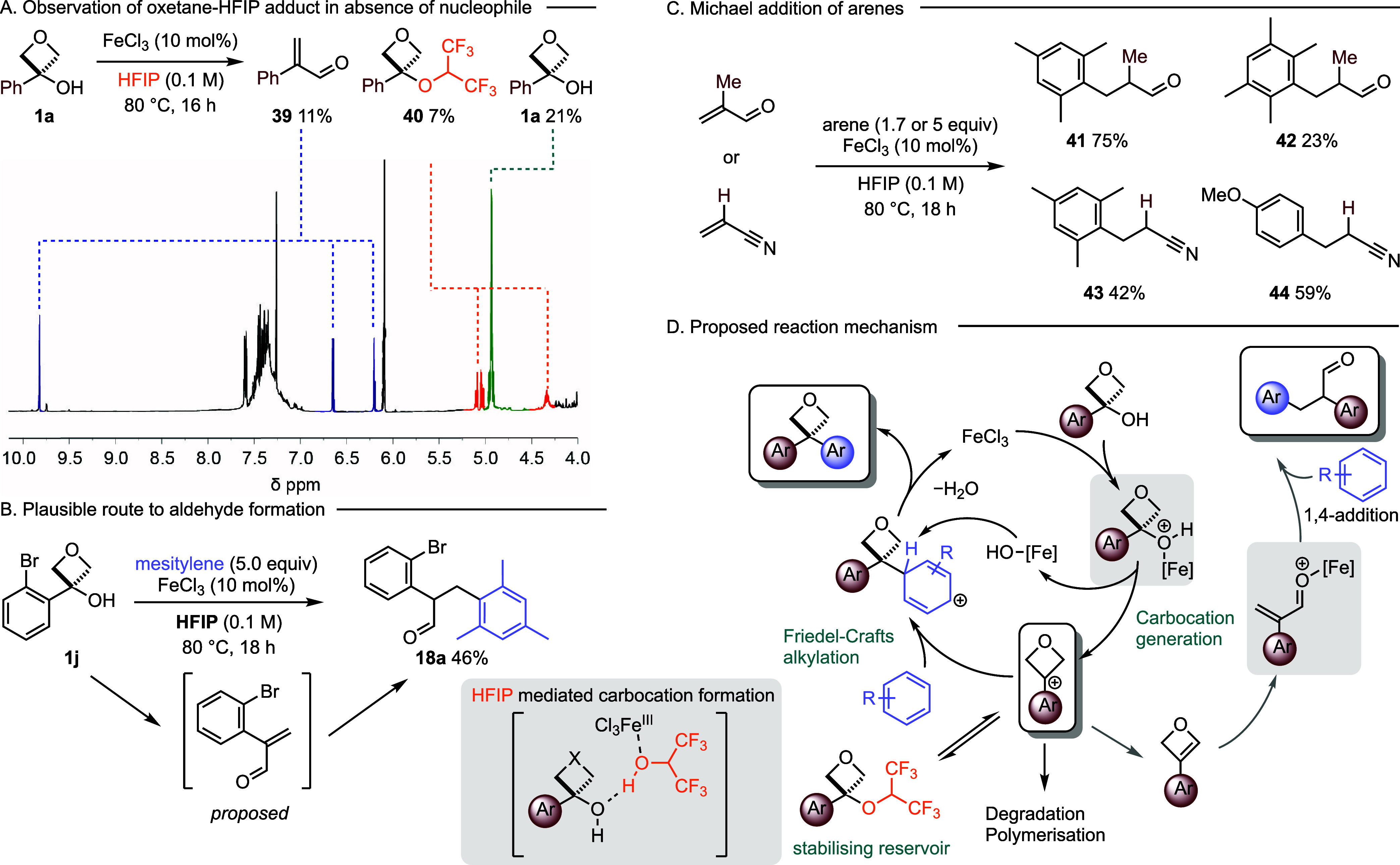
(A). Product distribution for the reaction of oxetanol **1a** in the absence of nucleophile was determined by ^1^H NMR spectroscopy of the crude reaction mixture using 1,3,5-trimethoxybenzene as the internal standard. (B) Proposed route to degradation to form a major aldehyde product. (C) Alternative Michael-acceptor reactions using the optimized HFIP Friedel–Crafts conditions. (D) Catalytic cycle for the HFIP-mediated Friedel–Crafts reaction of aromatics with oxetanols and formation of aldehyde side products.

The second product had one aldehyde peak δ_H_ = 9.8 ppm and two alkene signals at δ_H_ = 6.7 and 6.3 ppm (in blue, Figure 3A), each integrating for one proton, which is consistent with the known compound atropaldehyde (**39**, 2-phenylacrylaldehyde).^27^ The formation of this product was proposed to occur through deprotonation of the carbocation intermediate, forming an unstable oxetene species which rearranges into α,β–unsaturated aldehyde **39** (Figure 3A). We proposed that the Friedel–Crafts alkylation of the arene nucleophiles with the α,β–unsaturated aldehyde was likely to be the source of the observed aldehyde **a** side products observed in most cases through the successful reaction scope (See SI for isolation and characterization of the aldehyde products). These aldehyde products were particularly prevalent in the presence of *ortho*-substituted aryloxetanols, suggesting that the steric bulk of the *ortho*-substituents destabilize the carbocations or promote the rearrangement.^4c^ For example, *ortho*-bromophenyl oxetanol **1j** cleanly gave aldehyde **18a** in 46% isolated yield (Figure 3B).^20^

Atropaldehyde **39** proved unstable and could not be isolated; however, application of the reaction conditions to commercially available methacrolein afforded aldehyde **41** in 75% yield, consistent with aldehyde formation via 1,4-addition of aromatics (Figure 3C). Such Friedel–Crafts alkylation of arenes have previously been promoted by Lewis^28^ and Brønsted acid^29^ catalysts including Fe­(OTf)_3_,^30^ often with electron-rich arene nucleophiles.^31^ Other strategies,^32,33^ including synergistic FeCl_3_/TfOH catalysis in HFIP for nonbenzylic tertiary alcohols,^12c^ remain largely restricted to heteroarenes or electron-rich aromatics.

The application of FeCl_3_ with HFIP conditions enabled direct Friedel–Crafts alkylation of nonactivated aromatics with α,β-unsaturated aldehydes to yield 3-arylpropanal derivatives (**41**, **42**, Figure 3C). Acrylonitrile also functioned as a Michael acceptor under these conditions, reacting with mesitylene and anisole to give products **43** (42%) and **44** (59%).

A proposed mechanism for the iron-catalyzed Friedel–Crafts reaction of unactivated 3-aryl-oxetanols is outlined in Figure 3D. A [FeCl_3_·(HFIP)*_n_*]^+^ complex, akin to that proposed by Moran and Lebœuf,^13^ may activate either the oxetanol or α,β-unsaturated aldehyde. Coordination of FeCl_3_ or activated HFIP (boxed structure) to the oxetanol hydroxyl group promotes ionization to an oxetanyl carbocation, which alkylates the arene to give the 3,3-diaryloxetane. Reaction of the carbocation with HFIP directly generates an oxetane ether that can reversibly regenerate the carbocation, effectively serving as a cation reservoir, which aids stability and productive lifetime of the carbocation. Alternatively, α-deprotonation can occur if a Lewis base is present in the reaction, including competitively with Lewis basic nucleophiles (i.e., anisole, phenol), to form an unsaturated oxetene species, rationalizing the reduced yields observed with these substrates and the detrimental effect of higher nucleophile loadings in these cases. Owing to their ring strain, unsaturated oxetenes can readily rearrange to acrolein derivatives. Subsequent Fe-catalyzed activation of the aldehyde enables 1,4-addition of the arene, followed by rearomatization and tautomerization, as one route to yield ring-opened aldehyde products.

## Conclusion

We have developed an FeCl_3_-catalyzed Friedel–Crafts alkylation in HFIP that enables the efficient synthesis of 3,3-diaryloxetanes bearing electron-neutral and electron-poor arenes, overcoming previous limitations. The combination of Fe catalysis and HFIP cooperatively stabilizes the oxetanyl carbocation intermediate, allowing activation of oxetanols substituted with arenes bearing electron-donating, neutral, and electron-withdrawing substituents in both *para* and *meta* positions. A much wider range of arene nucleophiles was tolerated than previously reported for oxetane functionalization, including nonoxygenated arenes and masked phenols. The developed methodology enables concise synthetic access to two novel oxetane-containing drug analogues inaccessible by previous approaches, establishing the potential for FeCl_3_/HFIP catalysis for challenging oxetane functionalization reactions.

## Supplementary Material



## Data Availability

The data underlying this study are available in the published article, in its Supporting Information.

## Notes

Structures of aldehyde products, additional reaction optimization, mechanistic proposals for side product formation, detailed experimental procedures, characterization data, X-ray crystallography data, copies of ^1^H and ^13^C NMR spectra (PDF)
