# Supplementary material for: Iron-Catalyzed Friedel–Crafts Reactions of Unactivated 3‑Aryl-Oxetanols Exploiting HFIP Stabilization of Carbocations
Source: J Org Chem. 2026 Jun 11;91(25):8647–56. doi: 10.1021/acs.joc.6c00762 (PMC13316984; doi:10.1021/acs.joc.6c00762)
Supplement: Supplementary file 1 [file jo6c00762_si_001.pdf]

SUPPORTING INFORMATION

Iron Catalyzed Friedel–Crafts Reactions of Unactivated 3-Aryl-oxetanols Exploiting HFIP Stabilization of Carbocations

Maryne A. J. Dubois[a], Callum S. Begg[a], Tsz-Kan Ma[a], Juan J. Rojas[a], Andrew J. P. White[a], Chulho Choi[b] and James A. Bull[a]*

[a] Department of Chemistry, Imperial College London, Molecular Sciences Research Hub, White City Campus, Wood Lane W12 0BZ, UK
[b] Medicine Design, Pfizer Worldwide Research, Development and Medical, Eastern Point Rd., Groton, CT 06340, USA.

*E-mail: j.bull@imperial.ac.uk

Contents

General Experimental Considerations S2
Structures of Additional Compounds in SI S3
Selected Optimization of the Friedel–Crafts Alkylation of 2,6-Dimethylphenol with 3- Phenyloxetan-3-ol..... S5
The Relationship Between the Electronic Nature of the Aryloxetanol Substituent and the Yield of 3,3-Diaryloxetane S9
The Relationship Between the Substituent Identity of the Aryloxetanol and the Ratio of the Aldehydes Produced S10
Synthesis of 3,3-diaryloxetanes S12
Unsuccessful Substrates S27
Synthesis of Tesmilifene Analogue (32) S28
Synthesis of Fenofibrate Analogue (35) S29
Further derivatisation of iodoarene S30
Alternative Michael Acceptor Reactivity S31
X-Ray Crystal Structures S33
^{1}H and ^{13}C{^{1}H} NMR Spectra of Selected Compounds S40
References S99

General Experimental Considerations

All non-aqueous reactions were carried out under an inert atmosphere (argon) with flame-dried glassware, using standard techniques, unless specified. Friedel–Crafts reactions were carried out under inert atmosphere with flame-dried glassware, using standard techniques, using non-dried CH_2Cl_2, $CHCl_3$, toluene, EtOAc and HFIP. Anhydrous solvents were obtained by filtration through drying columns (THF, DMF, EtOH, MeCN) or used as supplied (1,4-dioxane, DME, CH_2Cl_2). Reactions in HFIP were carried out in sealed tubes using *Biotage* microwave vials (10-20 mL) and aluminium caps equipped with butyl septa. Larger scale reactions were carried out in standard round bottomed flasks. Reactions that required heating were performed in an oil bath on a hotplate.

Flash column chromatography was performed using 230–400 mesh silica, with the indicated solvent system according to standard techniques. Analytical thin-layer chromatography (TLC) was performed on precoated glass-backed silica gel plates. Visualization of the developed chromatogram was performed by UV absorbance (254 nm) and stained with aqueous potassium permanganate solution, phosphomolybdic acid solution, *para*-anisaldehyde solution or ninhydrin solution in ethanol. Infrared spectra (νmax, FTIR ATR) were recorded in reciprocal centimetres (cm^1).

Nuclear magnetic resonance spectra were recorded on either 400 or 500 MHz *Bruker* AvIII HD spectrometers with a *SampleXpress* automatic sample changer. The frequency used to record the NMR spectra is given in each assignment and spectrum (^1H NMR at 400 or 500 MHz; $^{13}C\{^1H\}$ NMR at 101 MHz or 126 MHz; ^{19}F NMR at 377 MHz). Chemical shifts for ^1H NMR spectra are recorded in parts per million (ppm) from tetramethylsilane with the solvent resonance as the internal standard ($CHCl_3$: δ = 7.27 ppm, DMSO: δ = 2.50 ppm, MeOH: δ = 3.35 ppm).[1] Data is reported as follows: chemical shift (multiplicity [s = singlet, d = doublet, t = triplet, q = quartet, p = pentet, h = heptet, m = multiplet and br = broad], coupling constant (in Hz), integration and assignment). ^{13}C NMR spectra were recorded with complete proton decoupling indicated by $^{13}C\{^1H\}$. Chemical shifts are reported in parts per million from tetramethylsilane with the solvent resonance as the internal standard ($^{13}CDCl_3$: δ = 77.0 ppm, $(^{13}CD_3)_2SO$: δ = 39.5 ppm, $^{13}CD_3OD$: δ = 49.0 ppm). Assignments of ^1H and ^{13}C spectra were based upon the analysis of δ and *J* values, as well as DEPT, COSY, HSQC and HMBC experiments where appropriate. ^{19}F and ^{31}P NMR spectra were recorded with or without complete proton decoupling. Decoupling is indicated as $^{19}F\{^1H\}$ and $^{31}P\{^1H\}$ and where relevant this is stated in each assignment. ^{19}F, ^{11}B and ^{31}P NMR spectra are indirectly referenced to $CFCl_3$, $BF_3{\bullet}OEt_2$, and H_3PO_4 automatically *via* direct measurement of the absolute frequency of the deuterium lock signal by the spectrometer hardware.

Melting points were recorded using *Optimelt* MPA100 apparatus and are uncorrected.

The high-resolution mass spectrometry (HRMS) analyses were performed using electrospray ion source (TOF ESI), atmospheric pressure chemical ionisation (FTMS APCI), or electron impact ionisation (TOF EI). ESI was performed using a *Waters* LCT Premier equipped with an ESI source operated in positive or negative ion mode. In most cases the software used was *MassLynx* 4.1. This software does not account for the electron for +ESI and the calibrations/references are calculated accordingly, i.e. $[M+H]^+$ is detected and the mass is calibrated to output [M+H]. APCI was performed using a Thermo Scientific Q-Extractive/Dionex Ultimate 3000 using an ASAP to insert samples into the APCI source operated in positive or negative mode. The sample was introduced at ambient temperature and the temperature increased until the sample vaporized.

Reagents
Commercial reagents were used as supplied or purified by standard techniques where necessary. Catalysts were purchased from the following suppliers, stored in a desiccator and used without further purification.

Iron(III) chloride (98%, CAS: 7705-08-0) was purchased from *Acros Organics* and stored under argon atmosphere..

Structures of Additional Compounds in SI

Oxetanols

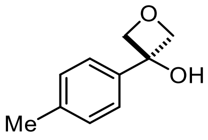

1b

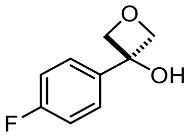

1c

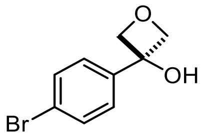

1e

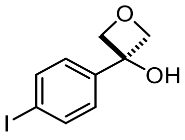

1f

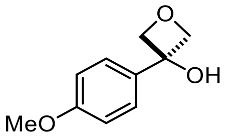

1g

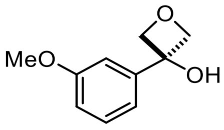

1h

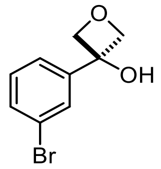

1i

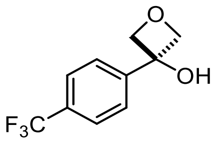

1k

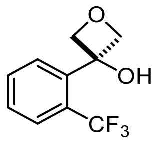

1l

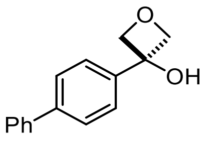

1m

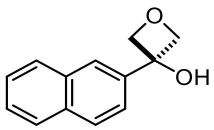

1n

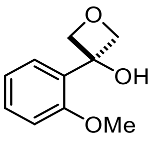

1o

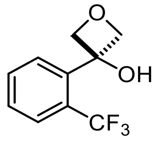

1p

Aldehydes and Ketones

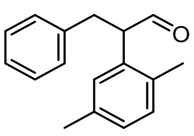

3b

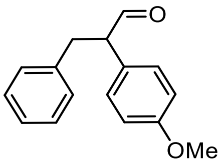

4b

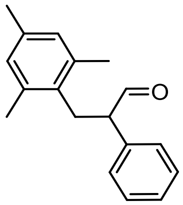

5a

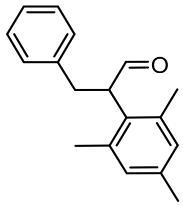

5b

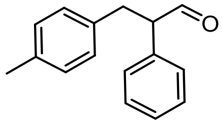

9a

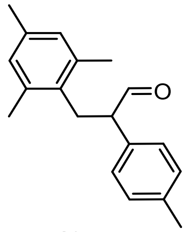

10a

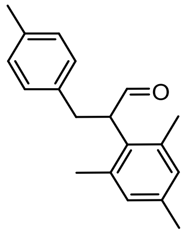

10b

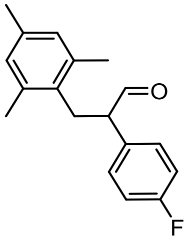

11a

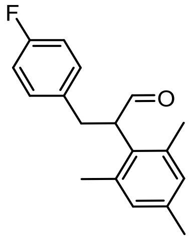

11b

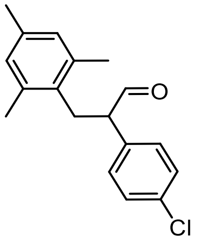

12a

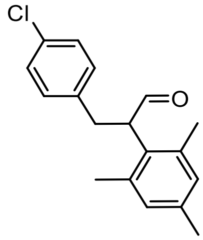

12b

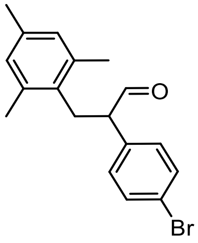

13a

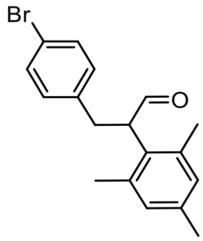

13b

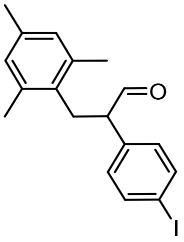

14a

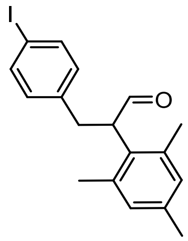

14b

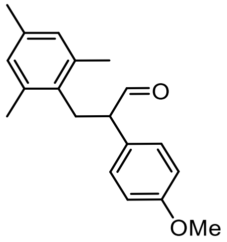

15a

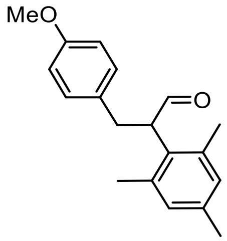

15b

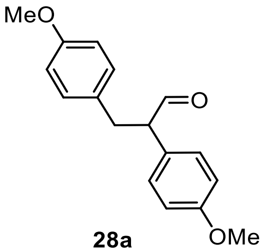

28a

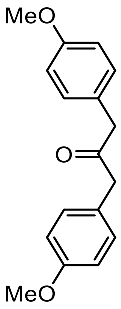

28c

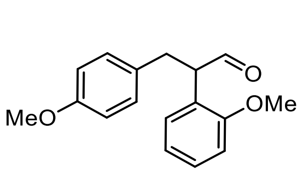

30a

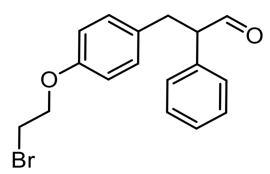

31a

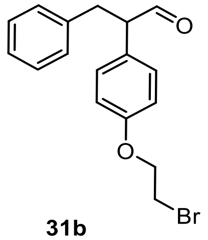

31b

Selected Optimization of the Friedel–Crafts Alkylation of 2,6-Dimethylphenol with 3- Phenyloxetan-3-ol

3- Phenyloxetan-3-ol **1a** was subjected to the reaction conditions employing 4 equivalents of nucleophile, with a combination of $Ca(NTf_2)_2$/$n$$Bu_4NPF_6$ in HFIP at 80 °C. Pleasingly, 14% of 3,3-diaryloxetane **2** was obtained. Although this result was promising, the starting material degraded under the conditions which made the improvement of this reaction challenging.

Table S1: Initial catalyst screen for the Friedel–Crafts alkylation of 2,6-dimethylphenol with oxetanol **1a**. General conditions: oxetanol (0.20 mmol), 2,6-dimethylphenol (0.80 mmol), catalyst (10 mol%), HFIP (1.0 mL), 80 °C, under argon. [a]Yields calculated by ^{1}H NMR spectroscopy using 1,3,5- trimethoxybenzene as the internal standard. [b]$Li(NTf_2)$ (11 mol%) and nBu_4NPF_6 (5.5 mol%) used. [c]20 mol% of catalyst used.

Table S1.

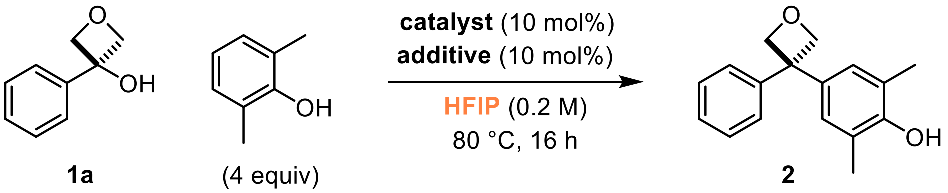

Entry	catalyst/additive	yield of **2** (%)[a]	yield of **1a** (%)[a]	Total (%)[a]
1	$Ca(NTf_2)_2$/$n$$Bu_4NPF_6$	17	0	17
2[b]	$Li(NTf_2)$/$n$$Bu_4NPF_6$	38	0	38
**3**	**$FeCl_3$/no additive**	**53**	**25**	**78**
4	LiCl/no additive	0	100	100
5	TFA	5	91	96
6	TfOH	21	0	21
7[c]	Tf_2NH	1	5	6
8	$F_5C_6B(OH)_2$	0	100	100
9	no catalyst/no additive	0	100	100
10	$FeCl_3$/$n$$Bu_4NPF_6$	4	64	68
11	$Fe(acac)_3$	0	100	100
12	$FeCl_2$	0	98	98
13	$Fe(OTf)_2$	20	0	20

Various Lewis and Brønsted acids were then tested to modulate the acidity and improve mass recovery (Table S1). Using lithium triflimide doubled the yield of oxetane **2** compared to calcium triflimide, albeit with similar loss of material (entry 2). Surprisingly, employing $FeCl_3$ resulted in a sharp increase in yield with 53% of **2** obtained and 25% of starting material recovered (entry 3). The combination of LiCl and HFIP was reported by Zhu to catalyse the Friedel–Crafts alkylation of phenol with ethylglycoxylate,[2] however this catalyst was found unreactive in our reaction (entry 4). Brønsted acids such as TFA, triflic acid and Tf_2NH were also investigated (entry 5 to 7), but none promoted efficiently the reaction leading to recovered starting material or degradation. McCubbin reported in 2010 a Friedel–Crafts alkylation with benzylic alcohol catalysed by $F_5C_6B(OH)_2$[3] Subjecting this catalyst to our reaction conditions resulted only in starting material returned (entry 8). Finally, HFIP on its own was unable to promote the reaction and oxetanol **1a** was fully recovered (entry 9).

$FeCl_3$ was the only catalyst that promoted the reaction without dramatic loss of starting material. In addition, this Lewis acid is an attractive catalyst being inexpensive and an earth-abundant metal and the reaction was further investigated with iron catalysts.

All iron catalysts were chosen for being relatively easy to handle and inexpensive (Table S1). The addition of an ammonium additive, commonly used with lithium and calcium triflimide, seemed to be detrimental to the reaction in combination with $FeCl_3$ (entry 10), while $Fe(acac)_3$ did not give any product (entry 11). Interestingly, $FeCl_2$ was unreactive compared to $FeCl_3$ (entry 12), but $Fe(OTf)_2$ gave 20% of product **2** showing the importance of the counter ion. With the optimum $FeCl_3$ catalyst in hand, the effect of the catalyst loading on the reaction was examined (Table S2).

Table S2: Effect of the catalyst loading and concentration on the Friedel–Crafts alkylation of 2,6-dimethylphenol with oxetanol **1a**. [a]Yields calculated by ^{1}H NMR spectroscopy using 1,3,5- trimethoxybenzene as the internal standard.

Table S2.

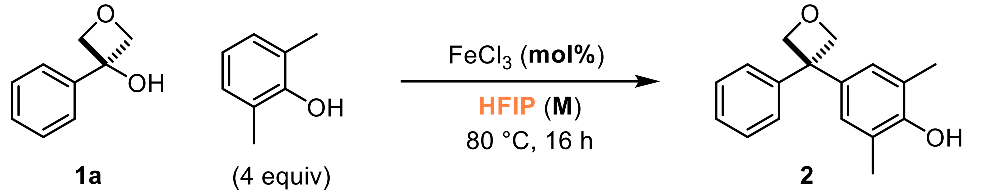

Entry	catalyst loading	Conc (M)	yield of **2** (%)[a]	yield of **1a** (%)[a]	Total (%)[a]
1	20	0.2	43	0	43
2	5	0.2	41	53	94
3	10	0.4	20	74	94
**4**	**10**	**0.1**	**73**	**0**	**73**

Doubling the catalyst loading to 20 mol% resulted in lower yield and more degradation (entry 1, Table S2). In comparison, halving the loading to 5 mol% afforded a better overall mass recovery, but with lower yield of product obtained (entry 2). Increasing the concentration to 0.4 M did not improve the yield but increased the mass recovery (entry 3). In contrast, reducing concentration to 0.1 M improved the reaction with 73% of **2** obtained and no oxetanol returned (entry 4).

Table S3: Solvents screen for the Friedel–Crafts alkylation of 2,6-dimethylphenol with oxetanol **1a**. [a]Yields calculated by ^{1}H NMR spectroscopy using 1,3,5-trimethoxybenzene as the internal standard.

Table S3.

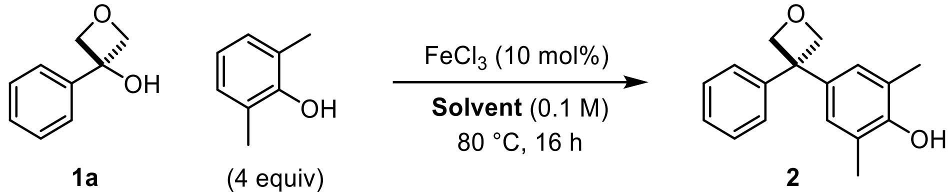

Entry	solvents (1:1)	yield of **2** (%)[a]	yield of **1** (%)[a]	Total (%)[a]
1	TFE	13	73	86
2	$MeNO_2$/HFIP	38	44	82
3	toluene/HFIP	20	75	95
4	EtOAc/HFIP	7	74	81
5	MeCN/HFIP	0	100	100

Alternative solvents were tested for this reaction (Table S3). TFE was not as efficient as HFIP to promote the reaction, with lower yield obtained (entry 1). Different combinations of solvent with HFIP were then tested. Mixtures of HFIP and $MeNO_2$ have been extensively used as solvents for the catalytic substitution of π–activated alcohols,[4] and gave 38% of product **2** when used in the reaction (entry 2). Toluene, EtOAc and acetonitrile as co-solvents all resulted in lower yield or no reaction, and HFIP was found to be the optimum medium for this reaction.

Table S4: Effect of the temperature on the Friedel–Crafts alkylation of 2,6-dimethylphenol with oxetanol **1a**. [a]Yields calculated by ^{1}H NMR spectroscopy using 1,3,5-trimethoxybenzene as the internal standard.

Table S4.

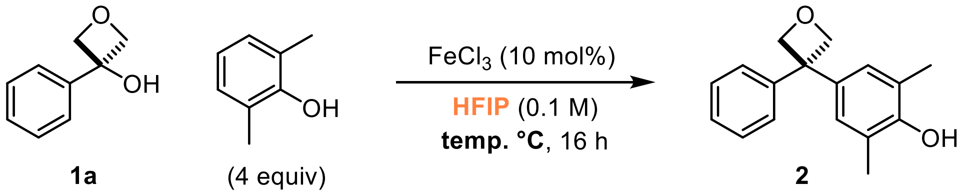

Entry	temp. (°C)	yield of **2** (%)[a]	yield of **1** (%)[a]	Total (%)[a]
1	40	53	0	53
2	60	52	0	52

The high temperature of 80 °C seemed to be important to the reaction, as running the reaction at 40 °C or 60 °C lowered the yield and the mass recovery (Table S4).

Table S5: Effect of the nucleophile loading on the Friedel–Crafts alkylation of 2,6-dimethylphenol with oxetanol **1a**. [a]Yields calculated by ^1H NMR spectroscopy using 1,3,5-trimethoxybenzene as the internal standard. [b]No action taken to avoid water and oxygen.

Table S5.

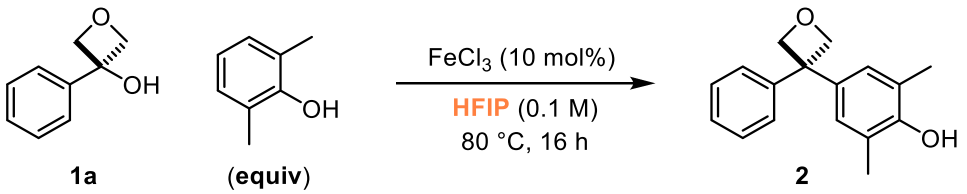

1a (**equiv**) **2**

Entry	Nucleophile equiv.	yield of **2** (%)[a]	yield of **1a** (%)[a]	Total (%)[a]
1	2	88	0	88
**2**	**1.7**	**94**	**0**	**94**
3	1.5	91	8	99
4	1.2	80	20	100
5[b]	1.7	83	10	93

The equivalents of nucleophile were found to be the most influential parameter on the reaction (Table S5). Indeed, halving the equivalents of the nucleophile to 2 improved the yield to 88% (entry 1). This was surprising as a larger amount of nucleophile is usually required to reach high yield in Friedel–Crafts reactions.[5] Reducing the equivalents further to 1.7 resulted in even higher yield of 94% (entry 2). Using 1.5 equivalents resulted in a similar outcome with 8% of oxetanol **1a** returned (entry 3). Fewer equivalents of nucleophile led to a loss of 10% yield, due to non-completion of the reaction (entry 4). Overall, reduction of the equivalents of nucleophile led to an important rise in yield but also to better mass recovery, which points out the detrimental effect of the phenol nucleophile on the reaction.

Employing 1.7 equivalents of nucleophile, in 0.1 M HFIP at 80 °C was optimal to reach high yield with minimum loss of material. Importantly, running the reaction without precaution to exclude water or oxygen had little effect on the outcome producing **2** in 83% (entry 5).

The Relationship Between the Electronic Nature of the Aryloxetanol Substituent and the Yield of 3,3-Diaryloxetane

The relationship between the electronic nature of the substituent and the yield of 3,3-diaryloxetane was examined using electrophilic substituent constants σ_p^+ and σ_m^+ calculated by Okamoto and Brown.[6] A preliminary correlation was found between the yield of oxetane products and the electronic nature of the oxetanols' substituent, when mesitylene (5.0 equiv) was used as a nucleophile . Higher yields obtained for groups with $-0.31 < \sigma^+ < 0.15$. Interestingly, the most electron-donating substituent *para*-OMe ($-0.31 < \sigma^+$) gave a lower yield. Oxetanols with substituents having $\sigma^+ > 0.15$ were unproductive or afforded very low yield due to the poor stabilisation of the carbocation intermediate.

Table S6: Effect of the electronic nature of the oxetanol and yield of the reaction with mesitylene.

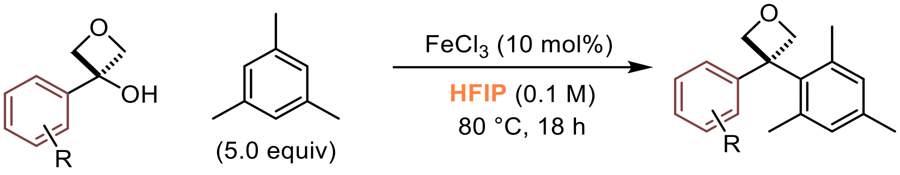

R Substituent	Electrophilic Substituent Constants (σ^+)	Yield (%)
OMe(*p*)	-0.78	62
Me(*p*)	-0.311	81
F(*p*)	-0.073	81
H	0	73
OMe(*m*)	0.047	78
Cl(*p*)	0.114	80
I(p)	0.135	78
Br(*p*)	0.15	76
Br(*m*)	0.405	14
CF_3(*m*)	0.52	3
CF_3(*p*)	0.61	3

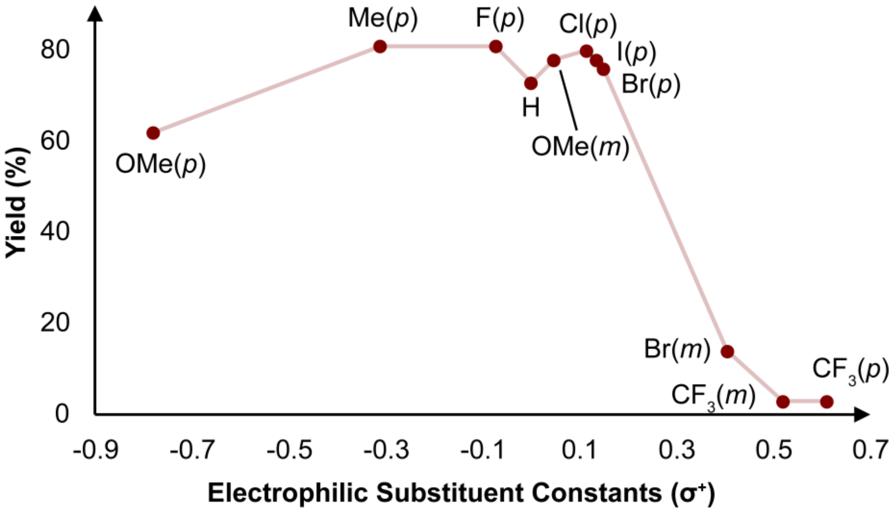

Figure S1: Graph representing the yield of 3,3-diaryloxetane of the reaction with mesitylene vs σ^+ of the corresponding substituent.

The Relationship Between the Substituent Identity of the Aryloxetanol and the Ratio of the Aldehydes Produced

During the reaction with mesitylene, aldehydes **a** and **b** were produced in different ratios according to the choice of oxetanol (**Figure S2**). The aldehydes were distinguished from one another using HSQC and HMBC NMR spectroscopy. Overall, aldehydes were only formed on substrates that were reactive to carbocation formation. For example, *para*-substituted oxetanols **1a–g** (aldehyde products **5, 10-15, 18**) gave a mixture of aldehydes **a** and **b** in 2% to 10% yields, with aldehyde **b** as major product in most cases. On the other hand, no aldehyde was formed with *para*-CF_3 substituted oxetanol **1k**. In addition, *ortho*-substitution seems to be detrimental to the formation of aldehyde **b**, and aldehyde **18a** and **30a** were obtained exclusively with the requisite oxetanols. *meta*-Substituted oxetanols exclusively reacted towards the formation of 3,3-diaryloxetanes and no aldehyde products were obtained. Finally, the distribution of aldehyde products was dependent on the scale, with only 1% of a 1:99 ratio of **14a:14b** on a 4.0 mmol scale compared to 9% of 51:49 mixture of **14a:14b** using 0.5 mmol of *p*-iodo oxetanol **1f**.

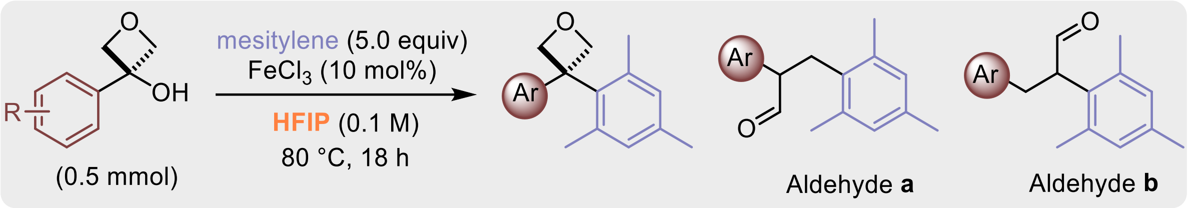

	**5**	**13**	**14**	**14**	**12**	**11**	**15**	**10**	**18**
R=	H (**1a**)	*p*-Br (**1e**)	*p*-I (**1f**)	*p*-I[a](**1f**)	*p*-Cl (**1d**)	*p*-F (**1c**)	*p*-OMe (**1g**)	*p*-Me (**1b**)	*o*-Br (**1j**)
Ratio a:b	30:70	42:58	51:49	1:99	38:62	1:99	20:80	16:84	100:0
Yield **a**+**b** (%)	10	6	9	1	7	7	2	6	46

Figure S2: Graph representing the yield of aldehydes of the reaction with mesitylene. [a]1 g scale.

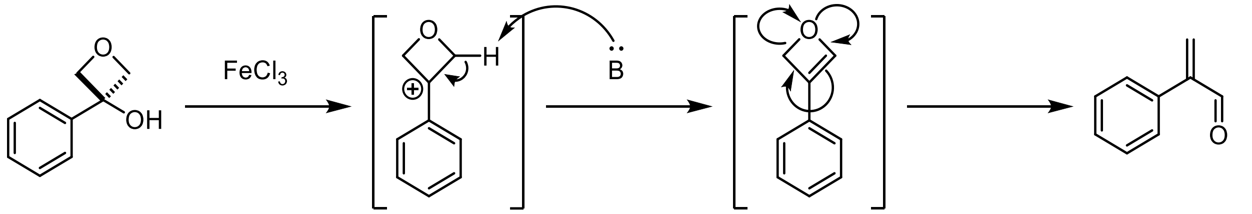

Figure S3: Proposed mechanism for the formation of α,β-unsaturated aldehyde which can act as a precursor for aldehyde **a**; see Figure 3 in manuscript.

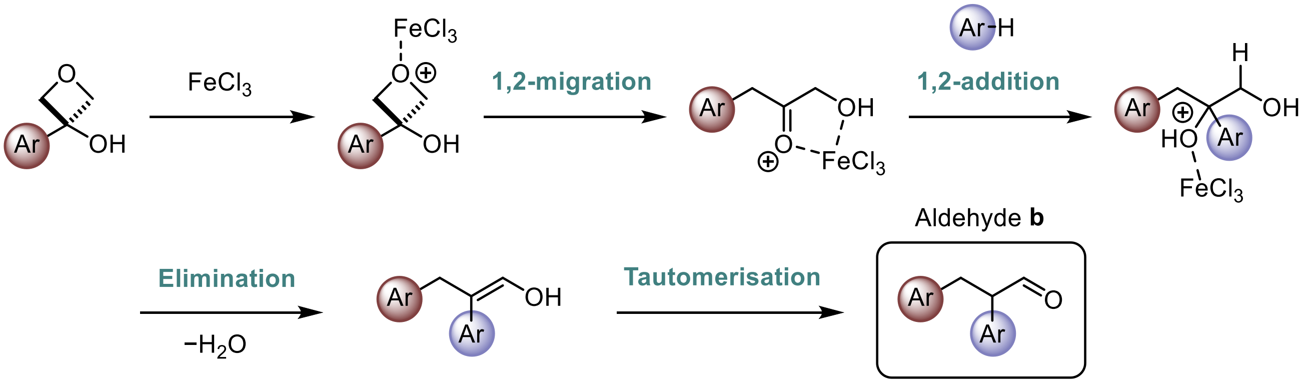

Figure S4: Proposed mechanism for the formation of aldehyde **b**.

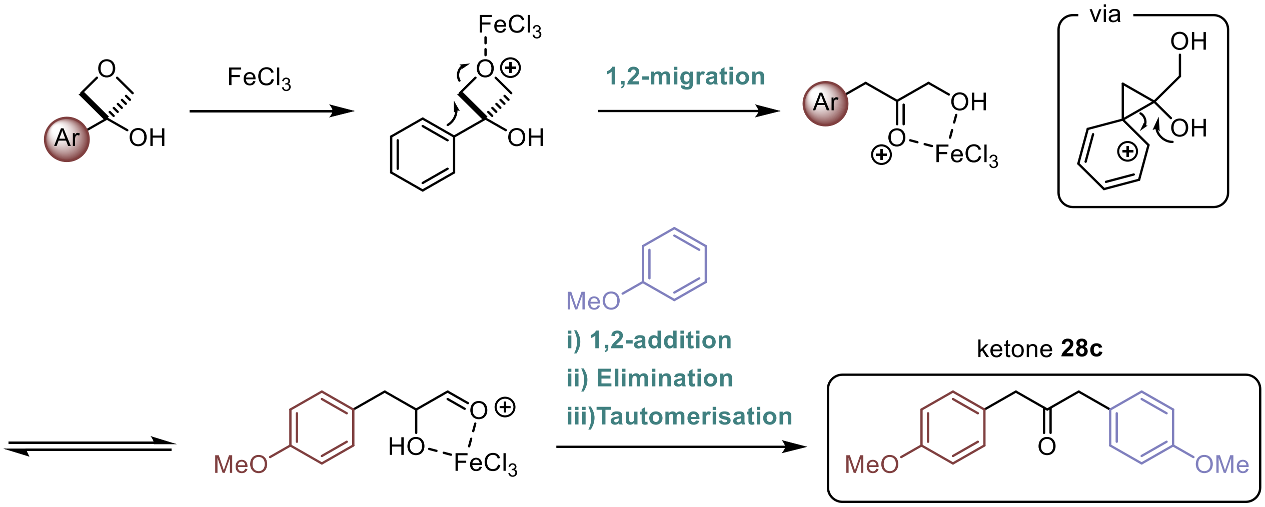

Figure S5: Proposed mechanism for the formation of ketone **28c**.

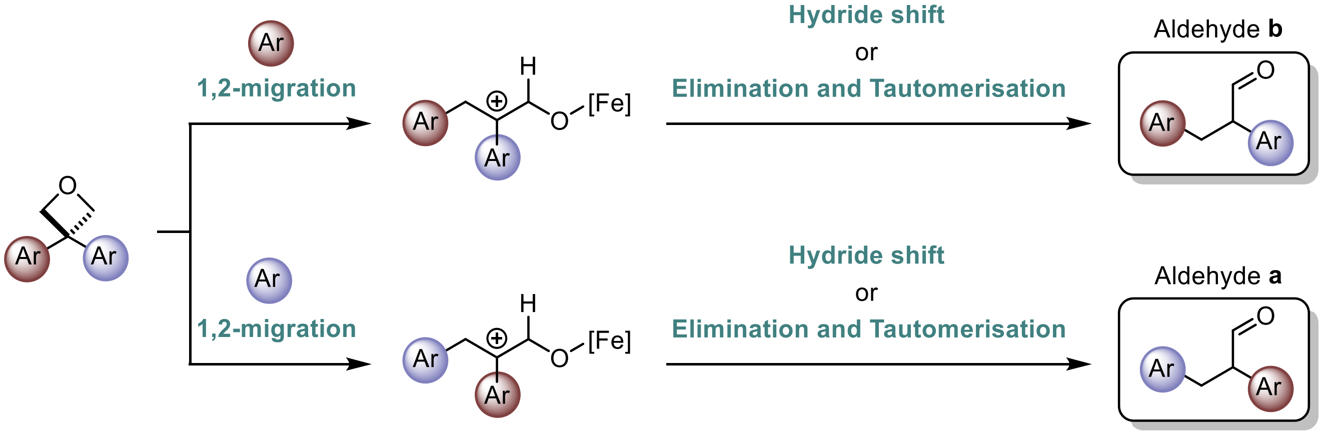

Figure S6: Alternative mechanism for the formation of aldehyde **a** and **b**.

Synthesis of 3,3-diaryloxetanes

Oxetanols **1a-p** were prepared according to previously reported procedures.[7-13]

2,6-Dimethyl-4-(3-phenyloxetan-3-yl)phenol (2)

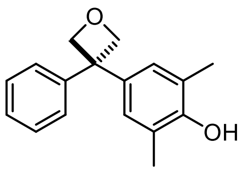

$FeCl_3$ (8.1 mg, 0.05 mmol) was weighed in a vial. 2,6-Dimethylphenol (104 mg, 0.85 mmol) and oxetanol **1a**[14] (75.1 mg, 0.50 mmol) were added. The reaction vessel was sealed and HFIP (5.0 mL) was added. The reaction was stirred for 18 h at 80 °C, then cooled to rt and sat. aq. $NaHCO_3$ (20 mL) was added followed by Et_2O (20 mL). The layers were separated, and the aqueous portion was extracted with Et_2O (2 × 20 mL). The organic extracts were combined, dried over Na_2SO_4, filtered and concentrated under reduced pressure. Purification by flash chromatography (30% Et_2O/pentane) afforded oxetane **2** (91.8 mg, 72%) as a yellow solid. R_f = 0.23 (30% Et_2O/pentane); mp = 167–170 °C; IR (film)/cm^{-1} 3880 (br, OH), 2959, 2881, 2363, 1490, 1211, 1181, 976, 700; ^1H NMR (400 MHz, $CDCl_3$) δ 7.41–7.33 (m, 2 H, 2 × Ar-CH), 7.32–7.20 (m, 3 H, 3 × Ar-CH), 6.82 (s, 2 H, 2 × Ar-CH), 5.24 (m, 4 H, CH_2OCH_2), 4.72 (m, 1 H, OH), 2.24 (s, 6 H, 2 × CH_3); $^{13}C\{^1H\}$ NMR (101 MHz, $CDCl_3$) δ 150.8 (Ar-C_q-OH), 146.2 (Ar-C_q-C_q), 137.4 (Ar-C_q-C_q), 128.5 (2 × Ar-CH), 126.7 (2 × Ar-CH), 126.5 (Ar-CH), 126.4 (2 × Ar-CH), 123.1 (2 × Ar-C_q-CH_3), 84.8 (CH_2OCH_2), 50.8 (C_q), 16.1 (2 × Ar-CH_3); HRMS (TOF MS ES–) *m/z* calcd for $C_{17}H_{17}O_2$ $[M–H]^-$: 253.1229, found: 253.1226.

3-(2,5-Dimethylphenyl)-3-phenyloxetane (3) and **3-(2,5-dimethylphenyl)-2-phenylpropanal (3b)**

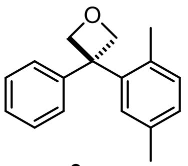

3

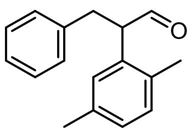

3b

$FeCl_3$ (8.1 mg, 0.05 mmol) was weighed in a vial. Oxetanol **1a** (75.1 mg, 0.50 mmol) and *para*-xylene (309 µL, 2.50 mmol) were added. The reaction vessel was sealed and HFIP (5.0 mL) was added. The reaction was stirred for 18 h at 80 °C, then cooled to rt and sat. aq. $NaHCO_3$ (20 mL) was added followed by Et_2O (15 mL). The layers were separated, and the aqueous portion was extracted with Et_2O (2 × 15 mL). The organic extracts were combined, dried over Na_2SO_4, filtered and concentrated under reduced pressure. Purification by flash chromatography (4% Et_2O/hexane) afforded impure aldehyde **3b** (12.3 mg, ca. 10%) as a colourless oil followed by oxetane **3** (65.1 mg, 55%) as a yellow oil.

Aldehyde **3b**: R_f = 0.31 (4% Et_2O/hexane); IR (film)/cm^{-1} 3026, 2922, 1722 (C=O), 1498, 1453, 1140, 812, 752, 700; ^1H NMR (400 MHz, $CDCl_3$) δ 9.70 (d, *J* = 1.2 Hz, 1 H, HC=O), 7.25–7.17 (m, 3 H, 3 × Ar-CH), 7.25–7.17 (m, 4 H, 4 × Ar-CH), 6.90 (s, 1 H, Ar-CH), 4.09–4.01 (m, 1 H, (C=O)-CH), 3.49 (dd, *J* = 13.8, 6.7 Hz, 1 H, CH-C*H*H), 2.87 (dd, *J* = 13.8, 7.4 Hz, 1 H, CH-CH*H*), 2.33 (s, 3 H, CH_3), 2.08 (s, 3 H, CH_3); $^{13}C\{^1H\}$ NMR (101 MHz, $CDCl_3$) δ 199.9 (C=O), 139.2 (Ar-C_q-CH_2), 136.1 (Ar-C_q-CH_3), 134.1 (Ar-C_q-CH and Ar-C_q-CH_3), 130.8 (Ar-CH), 129.1 (3 × Ar-CH), 128.9 (Ar-CH), 128.3 (2 × Ar-CH), 126.2 (Ar-CH), 56.9 ((C=O)-*C*H), 36.2 (CH-*C*H_2), 21.0 (Ar-CH_3), 19.1 (Ar-CH_3); HRMS (Magnet EI+) *m/z* calcd for $C_{17}H_{18}O$ [M]: 238.1358, found: 238.1352.

Oxetane **3**: R_f = 0.26 (4% Et_2O/hexane); IR (film)/cm^{-1} 2952, 2873, 1494, 1446, 1028, 991, 890, 812, 700; ^1H NMR (400 MHz, $CDCl_3$) δ 7.44–7.37 (m, 2 H, 2 × Ar-CH), 7.37–7.30 (m, 2 H, 2 × Ar-CH), 7.30–7.23 (m, 1 H, Ar-CH), 7.12–7.04 (m, 2 H, 2 × Ar-CH), 6.82 (s, 1 H, Ar-CH), 5.45 (d, *J* = 5.9 Hz, 2 H, C*H*HOC*H*H), 4.95 (d, *J* = 5.9 Hz, 2 H, CH*H*OCH*H*), 2.38 (s, 3 H, CH_3), 1.81 (s, 3 H, CH_3); $^{13}C\{^1H\}$ NMR (101 MHz, $CDCl_3$) δ 145.6 (Ar-C_q-C_q), 141.8 (Ar-C_q-C_q), 135.5 (Ar-C_q-CH_3), 132.8 (Ar-C_q-CH_3), 131.1 (Ar-CH), 128.5 (2 × Ar-CH), 128.1 (Ar-CH), 127.8 (Ar-CH), 126.8 (Ar-CH), 125.8 (2 × Ar-CH), 84.4 (CH_2OCH_2), 51.7 (C_q), 21.0 (Ar-CH_3), 19.1 (Ar-CH_3); HRMS (Magnet EI+) *m/z* calcd for $C_{17}H_{18}O$ [M]: 238.1358, found: 238.1355.

3-(4-Methoxyphenyl)-3-phenyloxetane (4) and **2-(4-methoxyphenyl)-3-phenylpropanal (4b)**

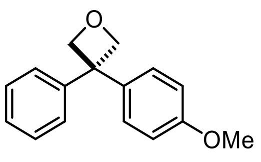

4

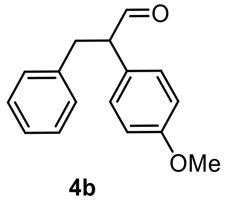

4b

$FeCl_3$ (8.1 mg, 0.05 mmol) was weighed in a vial. Oxetanol **1a** (75.1 mg, 0.50 mmol) and anisole (92.4 μL, 0.85 mmol) were added. The reaction vessel was sealed and HFIP (5.0 mL) was added. The reaction was stirred for 18 h at 80 °C. The reaction was then cooled to rt and sat. aq. $NaHCO_3$ (20 mL) was added followed by Et_2O (20 mL). The layers were separated, and the aqueous portion was extracted with Et_2O (2 × 20 mL). The organic extracts were combined, dried over Na_2SO_4, filtered and concentrated under reduced pressure. Purification by flash chromatography (4 to 6% Et_2O/hexane) afforded aldehyde **4b** (13.7 mg, 11%) as a colourless oil followed by oxetane **4** (43.9 mg, 37%) as a yellow solid.

Aldehyde **4b**: R_f = 0.23 (4% Et_2O/hexane); IR (film)/cm^{-1} 2938, 2832, 1722 (C=O), 1610, 1513, 1453, 1300, 1244, 1177, 1032, 823, 756, 700; ^{1}H NMR (400 MHz, $CDCl_3$) δ 9.75 (d, *J* = 1.6 Hz, 1 H, HC=O), 7.39–7.27 (m, 3 H, 3 × Ar-CH), 7.18–7.13 (m, 2 H, 2 × Ar-CH), 7.00–6.95 (m, 2 H, 2 × Ar-CH), 6.79–6.73 (m, 2 H, 2 × Ar-CH), 3.84–3.78 (m, 1 H, (C=O)-CH), 3.77 (s, 3 H, OCH_3), 3.42 (dd, *J* = 14.1, 6.8 Hz, 1 H, CH-C*H*H), 2.94 (dd, *J* = 14.1, 7.8 Hz, 1 H, CH-CH*H*); $^{13}C\{^1H\}$ NMR (101 MHz, $CDCl_3$) δ 200.1 (C=O), 158.0 (Ar-C_q-OCH_3), 135.8 (Ar-C_q), 130.7 (Ar-C_q), 130.0 (2 × Ar-CH), 128.99 (2 × Ar-CH), 128.98 (2 × Ar-CH), 127.6 (Ar-CH), 113.7 (2 × Ar-CH), 61.2 ((C=O)-*C*H), 55.2 (OCH_3), 35.3 (CH-CH_2); HRMS (FTMS +APCI) *m/z* calcd for $C_{16}H_{17}O_2^+$ $[M+H]^+$: 241.1223, found: 241.1224.

Oxetane **4**: R_f = 0.14 (4% Et_2O/hexane); mp = 50–51 °C; IR (film)/cm^{-1} 2952, 2877, 1610, 1513, 1248, 1028, 991, 890, 812, 700; ^{1}H NMR (400 MHz, $CDCl_3$) δ 7.41–7.33 (m, 2 H, 2 × Ar-CH), 7.32–7.23 (m, 1 H, Ar-CH), 7.24–7.20 (m, 2 H, 2 × Ar-CH), 7.18–7.12 (m, 2 H, 2 × Ar-CH), 6.93–6.86 (m, 2 H, 2 × Ar-CH), 5.26 (d, *J* = 5.6 Hz, 2 H, C*H*HOC*H*H), 5.22 (d, *J* = 5.6 Hz, 2 H, CH*H*OCH*H*), 3.82 (s, 3 H, OCH_3); $^{13}C\{^1H\}$ NMR (101 MHz, $CDCl_3$) δ 158.2 (Ar-C_q-OCH_3), 146.0 (Ar-C_q-C_q), 138.0 (Ar-C_q-C_q), 128.5 (2 × Ar-CH), 127.6 (2 × Ar-CH), 126.6 (Ar-CH), 126.5 (2 × Ar-CH), 113.9 (2 × Ar-CH), 84.8 (CH_2OCH_2), 55.3 (OCH_3), 50.9 (C_q); HRMS (FTMS +APCI) *m/z* calcd for $C_{16}H_{17}O_2^+$ $[M+H]^+$: 241.1223, found: 241.1224.

3-Mesityl-3-phenyloxetane (5), 3-mesityl-2-phenylpropanal (5a) and **2-mesityl-3-phenylpropanal (5b)**

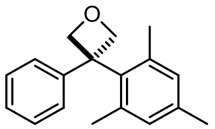

5

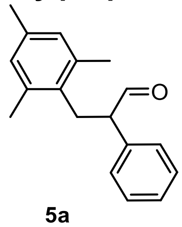

5a

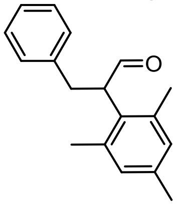

5b

$FeCl_3$ (8.1 mg, 0.05 mmol) was weighed in a vial. Oxetanol **1a** (75.1 mg, 0.50 mmol) and mesitylene (348 μL, 2.50 mmol) were added. The reaction vessel was sealed and HFIP (5.0 mL) was added. The reaction was stirred for 18 h at 80 °C, then cooled to rt and sat. aq. $NaHCO_3$ (20 mL) was added followed by Et_2O (15 mL). The layers were separated, and the aqueous portion was extracted with Et_2O (2 × 15 mL). The organic extracts were combined, dried over Na_2SO_4, filtered and concentrated under reduced pressure. Purification by flash chromatography (4% Et_2O/pentane) afforded a mixture of aldehyde **5a** and **5b** (24:76, 12.9 mg, 10%) as a yellow solid, followed by oxetane **5** (91.6 mg, 73%) as a yellow solid.

Aldehyde **5a** and **5b**: R_f = 0.53 (4% Et_2O/pentane); ^{1}H NMR (400 MHz, $CDCl_3$) δ 9.84 (d, *J* = 1.5 Hz, 1 H, $H_aC=O$), 9.79 (s, 1 H, $H_bC=O$), 7.39–7.31 (m, 3 H, 3 × Ar-CH_a), 7.26–7.16 (m, 3 H, 3 × Ar-CH_b), 7.12–7.08 (m, 2 H, 2 × Ar-CH_a), 7.05–7.00 (m, 2 H, 2 × Ar-CH_b), 6.87 (s, 2 H, 2 × Ar-CH_b), 6.80 (s, 2 H, 2 × Ar-CH_a), 3.97 (dd, *J* = 8.1, 4.9 Hz, 1 H, (C=O)-CH_b), 3.69 (dd, *J* = 13.6, 4.9 Hz, 1 H, CH-C*H*H_b), 3.69 (m, 1 H, (C=O)-CH_a), 3.53 (dd, *J* = 14.2, 5.6 Hz, 1 H, CH-C*H*H_a), 2.91 (dd, *J* = 14.2, 8.2 Hz, 1 H, CH-CHH_a), 2.85 (dd, *J* = 13.6, 8.1 Hz, 1 H, CH-CHH_b), 2.29 (s, 3 H, $CH_{3,b}$), 2.26 (s, 3 H, $CH_{3,a}$), 2.07 (m, 12 H, 2 × $CH_{3(a+b)}$); $^{13}C\{^1H\}$

NMR (101 MHz, $CDCl_3$) δ 201.6 (C_b=O), 200.3 (C_a=O), 140.0 (Ar-$C_{q,b}$), 137.2 (Ar-$C_{q,b}$), 136.6 (Ar-$C_{q,a}$), 136.5 (Ar-$C_{q,a}$), 135.6 (Ar-$C_{q,a}$), 132.6 (Ar-$C_{q,b}$), 130.6 (2 × Ar-CH_a), 129.9 (br, 4 × Ar-C_q-CH_3), 129.13 (2 × Ar-CH_b), 129.07 (2 × Ar-CH_b), 129.06 (2 × Ar-CH_a), 128.9 (2 × Ar-CH_a), 128.2 (2 × Ar-CH_b), 127.6 (Ar-CH_a), 126.1 (Ar-CH_b), 59.4 ((C=O)-CH_a), 57.0 ((C=O)-CH_b), 34.1 (CH-$CH_{2,b}$), 30.3 (CH-$CH_{2,a}$), 20.80 (Ar-$CH_{3,b}$), 20.78 (Ar-$CH_{3,a}$), 20.6 (2 × Ar-$CH_{3,b}$), 19.9 (2 × Ar-$CH_{3,a}$).

Oxetane **5**: R_f = 0.27 (4% Et_2O/pentane); mp = 96–99 °C; IR (film)/cm^{-1} 2952, 2870, 1610, 1490, 1446, 1379, 1189, 1032, 943, 849, 760, 700; ^1H NMR (400 MHz, $CDCl_3$) δ 7.52–7.47 (m, 2 H, 2 × Ar-CH), 7.39–7.28 (m, 3 H, 3 × Ar-CH), 6.90 (s, 2 H, 2 × Ar-CH), 5.41 (d, *J* = 6.0 Hz, 2 H, C*H*HOC*H*H), 4.83 (d, *J* = 6.0 Hz, 2 H, CH*H*OCH*H*), 2.33 (s, 3 H, CH_3), 1.87 (s, 6 H, 2 × CH_3); $^{13}C\{^1H\}$ NMR (101 MHz, $CDCl_3$) δ 144.9 (2 × Ar-C_q-C_q), 137.8 (Ar-C_q-CH_3), 136.2 (2 × Ar-C_q-CH_3), 129.8 (2 × Ar-CH), 128.6 (2 × Ar-CH), 127.0 (Ar-CH), 125.7 (2 × Ar-CH), 85.2 (CH_2OCH_2), 52.6 (C_q), 20.7 (Ar-CH_3), 20.2 (2 × Ar-CH_3); HRMS (FTMS +APCI) *m/z* calcd for $C_{18}H_{21}O^+$ $[M+H]^+$: 253.1587, found: 253.1587.

3-Phenyl-3-(2,3,5,6-tetramethylphenyl)oxetane (6)

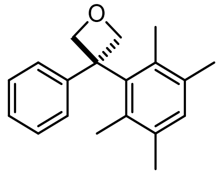

$FeCl_3$ (10.8 mg, 0.07 mmol) was weighed in a vial. Oxetanol **1a** (77 mg, 0.51 mmol) and durene (337 mg, 5.0 mmol) were added. The reaction vessel was sealed and HFIP (5.0 mL) was added. The reaction was stirred for 18 h at 80 °C, then cooled to rt and sat. aq. $NaHCO_3$ (20 mL) was added followed by Et_2O (15 mL). The layers were separated, and the aqueous portion was extracted with Et_2O (2 × 15 mL). The organic extracts were combined, dried over Na_2SO_4, filtered and concentrated under reduced pressure. Purification by flash chromatography (5% Et_2O/pentane) afforded oxetane **6** (97.2 mg, 71%) as an off-white. R_f = 0.32 (5% Et_2O/pentane); mp = 99–100 °C (Et_2O/pentane ppt); IR (film)/cm^{-1} 2947, 2871, 1720, 1492, 1466, 992, 701; ^1H NMR (400 MHz, $CDCl_3$) δ 7.56–7.51 (m, 2 H, 2 × Ar-CH), 7.38–7.33 (m, 2 H, 2 × Ar-CH), 7.32–7.28 (m, 1 H, Ar-CH), 6.98 (s, 1 H, Ar-CH), 5.29 (d, *J* = 5.7 Hz, 2 H, C*H*HOC*H*H), 4.88 (d, *J* = 5.7 Hz, 2 H, CH*H*OCH*H*), 2.24 (s, 6 H, 2 × Ar-CH_3), 1.70 (s, 6 H, 2 × Ar-CH_3); $^{13}C\{^1H\}$ NMR (101 MHz, $CDCl_3$) δ 145.5 (Ar-C_q-C_q), 141.6 (Ar-C_q-C_q), 134.8 (2 × Ar-C_q-CH_3), 132.5 (2 × Ar-C_q-CH_3), 130.3 (Ar-CH), 128.7 (2 × Ar-CH), 127.2 (Ar-CH), 126.0 (2 × Ar-CH), 85.9 (CH_2OCH_2), 53.4 (C_q), 20.2 (Ar-CH_3), 17.1 (Ar-CH_3); HRMS (FTMS –ESI) *m/z* calcd for $C_{19}H_{21}O$ $[M–H]^-$: 265.1598, found: 265.1598.

3-(5-Isopropyl-2-methylphenyl)-3-phenyloxetane (7)

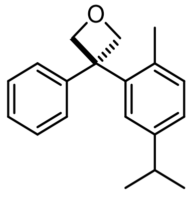

$FeCl_3$ (10.4 mg, 0.06 mmol) was weighed in a vial. Oxetanol **1a** (79 mg, 0.53 mmol) and *p*-cymene (390 uL, 5.0 mmol) were added. The reaction vessel was sealed and HFIP (5.0 mL) was added. The reaction was stirred for 18 h at 80 °C, then cooled to rt and sat. aq. $NaHCO_3$ (20 mL) was added followed by Et_2O (15 mL). The layers were separated, and the aqueous portion was extracted with Et_2O (2 × 15 mL). The organic extracts were combined, dried over Na_2SO_4, filtered and concentrated under reduced pressure. Purification by flash chromatography (5% Et_2O/pentane) afforded oxetane **7** (50.4 mg, 42%) as a brown oil. R_f = 0.34 (5% Et_2O/pentane); IR (film)/cm^{-1} 2958, 2872, 1493, 992, 759, 701; ^1H NMR (400 MHz, $CDCl_3$) δ 7.41 (dd, *J* = 8.4, 1.4 Hz, 2 H, 2 × Ar-CH), 7.34 (t, *J* = 7.5 Hz, 3 H, 3 × Ar-CH), 7.26 (d, *J* = 2.9 Hz, 1 H, Ar-CH), 7.13 (d, *J* = 1.3 Hz, 2 H, 2 × Ar-CH), 6.90 (s, 1 H, Ar-CH), 5.47 (d, *J* = 5.9 Hz, 2 H, C*H*HOC*H*H), 4.97 (d, *J* = 5.8 Hz, 2 H, CH*H*OCH*H*), 2.93 (p, *J* = 6.9 Hz, 1 H, Ar-C*H*$(CH_3)_2$), 1.82 (s, 3 H, Ar-CH_3), 1.29 (d, *J* = 6.9 Hz, 6 H, Ar-CH$(CH_3)_2$);$^{13}C\{^1H\}$ NMR (101 MHz, $CDCl_3$) δ 146.8 (Ar-C_q-CH$(CH_3)_2$), 145.8 (Ar-C_q-C_q), 141.9 (Ar-$C_{q(cymene)}$-C_q), 133.3 (Ar-C_q-CH_3), 131.3 (Ar-CH), 128.7 (2 × Ar-CH), 126.9 (Ar-CH), 126.0 (2 × Ar-CH), 125.8 (Ar-CH), 125.1 (Ar-CH), 84.6 (CH_2OCH_2), 52.0 (C_q), 33.9 (Ar-CH_3), 24.3 (Ar-CH$(CH_3)_2$), 19.3 (Ar-CH$(CH_3)_2$); HRMS (FTMS –ESI) *m/z* calcd for $C_{19}H_{21}O$ $[M−H]^-$: 265.1598, found: 265.1598. The major regioisomer was determined by ^1H-^{13}C HMBC NMR spectroscopy, with Ar-C<u>*H*</u>$(CH_3)_2$ coupling with Ar-<u>$C_{q(cymene)}$</u>-C_q.

3,3-Diphenyloxetane (8)

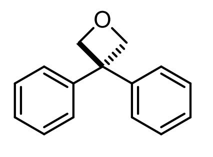

$FeCl_3$ (8.1 mg, 0.05 mmol) was weighed in a vial. Oxetanol **1a** (75.1 mg, 0.50 mmol) and benzene (222 μL, 2.50 mmol) were added. The reaction vessel was sealed and HFIP (5.0 mL) was added. The reaction was stirred for 18 h at 80 °C, then cooled to rt and sat. aq. $NaHCO_3$ (20 mL) was added followed by Et_2O (15 mL). The layers were separated, and the aqueous portion was extracted with Et_2O (2 × 15 mL). The organic extracts were combined, dried over Na_2SO_4, filtered and concentrated under reduced pressure. Purification by flash chromatography (4% Et_2O/pentane) afforded oxetane **8** (11.7 mg, 11%) as a yellow solid. R_f = 0.40 (4% Et_2O/pentane); mp = 72–74 °C; IR (film)/cm^{-1} 2955, 2881, 1490, 1084, 1021, 984, 935, 831, 760, 700; ^1H NMR (400 MHz, $CDCl_3$) δ 7.41–7.34 (m, 4 H, 4 × Ar-CH), 7.31–7.27 (m, 2 H, 2 × Ar-CH), 7.26–7.21 (m, 4 H, 4 × Ar-CH), 5.28 (s, 4 H, CH_2OCH_2); $^{13}C\{^1H\}$ NMR (101 MHz, $CDCl_3$) δ 145.8 (2 × Ar-C_q-C_q), 128.6 (4 × Ar-CH), 126.6 (2 × Ar-CH), 126.5 (4 × Ar-CH), 84.6 (CH_2OCH_2), 51.5 (C_q); HRMS *Mass not found*.

3-Phenyl-3-(*p*-tolyl)oxetane (*p*-9), 3-phenyl-3-(*o*-tolyl)oxetane (*o*-9) and **2-phenyl-3-(p-tolyl)propanal (9a)**

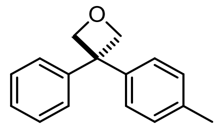

***p*-9**

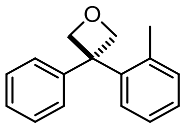

***o*-9**

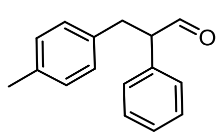

9a

$FeCl_3$ (8.1 mg, 0.05 mmol) was weighed in a vial. Oxetanol **1a** (75.1 mg, 0.50 mmol) and toluene (266 μL, 2.50 mmol) were added. The reaction vessel was sealed and HFIP (5.0 mL) was added. The reaction was stirred for 18 h at 80 °C, then cooled to rt and sat. aq. $NaHCO_3$ (20 mL) was added followed by Et_2O (15 mL). The layers were separated, and the aqueous portion was extracted with Et_2O (2 × 15 mL). The organic extracts were combined, dried over Na_2SO_4, filtered and concentrated under reduced pressure. Purification by flash chromatography (30% to 40% CH_2Cl_2/pentane) afforded aldehyde **9a** (88% pure, 31.5 mg, 28%) as a yellow oil, oxetane ***o*-9** (4.1 mg, 4%) as a yellow paste and oxetane ***p*-9** (11.7 mg, 12%) as an orange oil.

Aldehyde **9a**: R_f = 0.41 (30% CH_2Cl_2/pentane); IR (film)/cm^{-1} 3026, 2922, 2858, 2817, 1722 (C=O), 1513, 1453, 1136, 1028, 812, 760, 700; ^1H NMR (400 MHz, $CDCl_3$) δ 9.75 (d, *J* = 1.6 Hz, 1 H, HC=O), 7.39–7.28 (m, 3 H, 3 × Ar-CH), 7.18–7.13 (m, 2 H, 2 × Ar-CH), 7.04 (d, *J* = 7.7 Hz, 2 H, 2 × Ar-CH), 6.96 (d, *J* = 8.0 Hz, 2 H, 2 × Ar-CH), 3.83 (ddd, *J* = 8.2, 7.1, 1.6 Hz, 1 H, (C=O)-CH), 3.44 (dd, *J* = 14.1, 7.1 Hz, 1 H, CH-C*H*H), 2.95 (dd, *J* = 14.1, 8.2 Hz, 1 H, CH-CH*H*), 2.29 (s, 3 H, CH_3); $^{13}C\{^1H\}$ NMR (101 MHz, $CDCl_3$) δ 200.1 (C=O), 135.8 (Ar-C_q), 135.6 (Ar-C_q), 129.01 (2 × Ar-CH), 129.00 (2 × Ar-CH), 128.99 (2 × Ar-CH), 128.9 (2 × Ar-CH), 128.3 (Ar-C_q-CH_3), 127.6 (Ar-CH), 61.0 ((C=O)-*C*H), 35.7 (CH-CH_2), 21.0 (Ar-CH_3).

Oxetane ***o*-9**: R_f = 0.29 (30% CH_2Cl_2/pentane); IR (film)/cm^{-1} 3056, 3022, 2952, 2922, 2873, 1490, 1446, 1032, 991, 756, 700; ^1H NMR (400 MHz, $CDCl_3$) δ 7.42–7.37 (m, 2 H, 2 × Ar-CH), 7.37–7.31 (m, 2 H, 2 × Ar-CH), 7.31–7.23 (m, 3 H, 3 × Ar-CH), 7.22–7.18 (m, 1 H, Ar-CH), 7.06 (dd, *J* = 7.0, 2.0 Hz, 1 H, Ar-CH), 5.45 (d, *J* = 5.8 Hz, 2 H, C*H*HOC*H*H), 4.97 (d, *J* = 5.8 Hz, 2 H, CH*H*OCH*H*), 1.85 (s, 3 H, CH_3); $^{13}C\{^1H\}$ NMR (101 MHz, $CDCl_3$) δ 145.4 (Ar-C_q-C_q), 142.0 (Ar-C_q-C_q), 136.0 (Ar-C_q-CH_3), 131.3 (Ar-CH), 128.6 (2 × Ar-CH), 127.3 (Ar-CH), 127.2 (Ar-CH), 126.8 (Ar-CH), 126.0 (Ar-CH), 125.8 (2 × Ar-CH), 84.3 (CH_2OCH_2), 51.7 (C_q), 19.6 (Ar-CH_3); HRMS (FTMS +ESI) *m/z* calcd for $C_{16}H_{17}O^+$ $[M+H]^+$: 225.1274, found: 225.1271.

Oxetane ***p*-9**: R_f = 0.21 (30% CH_2Cl_2/pentane); IR (film)/cm^{-1} 3026, 2952, 2877, 1513, 1446, 1021, 991, 939, 816, 760, 700; ^1H NMR (400 MHz, $CDCl_3$) δ 7.40–7.35 (m, 2 H, 2 × Ar-CH), 7.30–7.27 (m, 1 H, Ar-CH), 7.26–7.22 (m, 2 H, 2 × Ar-CH), 7.19 (d, *J* = 8.0 Hz, 2 H, 2 × Ar-CH), 7.15–7.11 (m, 2 H, 2 × Ar-CH), 5.29–5.24 (m, 4 H, CH_2OCH_2), 2.37 (s, 3 H, CH_3); $^{13}C\{^1H\}$ NMR (101 MHz, $CDCl_3$) δ 145.9 (Ar-C_q-C_q), 142.8 (Ar-C_q-C_q), 136.3 (Ar-C_q-CH_3), 129.2 (2 × Ar-CH), 128.5 (2 × Ar-CH), 126.6 (Ar-CH), 126.43 (2 × Ar-CH), 126.35 (2 × Ar-CH), 84.7 (CH_2OCH_2), 51.1 (C_q), 21.0 (Ar-CH_3); HRMS (+EI) *m/z* calcd for $C_{16}H_{16}O^{\bullet+}$ $[M]^{\bullet+}$: 224.1196, found: 224.1201.

3-Mesityl-3-(*p*-tolyl)oxetane (10), 3-mesityl-2-(*p*-tolyl)propanal (10a) and **2-mesityl-3-(*p*-tolyl)propanal (10b)**

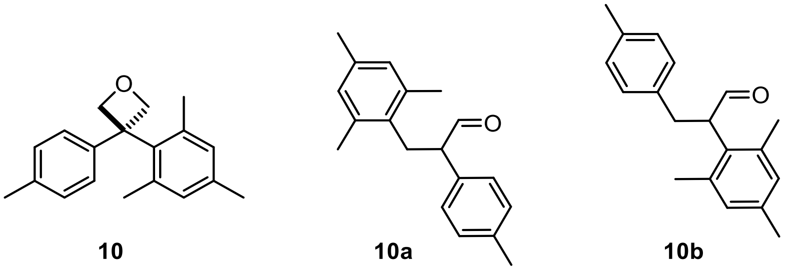

10 **10a** **10b**

$FeCl_3$ (8.1 mg, 0.05 mmol) was weighed in a vial. *p*-Tolyl-oxetanol **1b** (82.0 mg, 0.50 mmol) and mesitylene (350 µL, 2.50 mmol) were added. The reaction vessel was sealed and HFIP (5.0 mL) was added. The reaction was stirred for 18 h at 80 °C, then cooled to rt and sat. aq. $NaHCO_3$ (20 mL) was added followed by Et_2O (15 mL). The layers were separated, and the aqueous portion was extracted with Et_2O (2 × 15 mL). The organic extracts were combined, dried over Na_2SO_4, filtered and concentrated under reduced pressure. Purification by flash chromatography (4% Et_2O/pentane) afforded a mixture of aldehyde **10a** and **10b** (16:84, 7.4 mg, 6%) as a yellow oil, followed by oxetane **10** (108 mg, 81%) as a yellow oil.

Aldehyde **10a** and **10b**: R_f = 0.63 (4% Et_2O/pentane); IR (film)/cm^{-1} 3004, 2922, 2862, 2810, 1725 (C=O), 1610, 1513, 1449, 1025, 853, 812; ^{1}H NMR (400 MHz, $CDCl_3$) δ 9.79 (d, *J* = 1.5 Hz, 1 H, H_aC=O), 9.77 (s, 1 H, H_bC=O), 7.16–7.13 (m, 2 H, 2 × Ar-CH_a), 7.03–6.99 (m, 2 H, 2 × Ar-CH_b), 6.99–6.96 (m, 2 H, 2 × Ar-CH_a), 6.92–6.89 (m, 2 H, 2 × Ar-CH_b), 6.86 (s, 2 H, 2 × Ar-CH_b), 6.79 (s, 2 H, 2 × Ar-CH_a), 3.94 (dd, *J* = 7.9, 5.2 Hz, 1 H, (C=O)-CH_b), 3.67–3.60 (m, 1 H, (C=O)-CH_a, and dd, *J* = 13.7, 5.2 Hz, 1 H, CH-C*H*H_b), 3.49 (dd, *J* = 14.3, 5.5 Hz, 1 H, CH-C*H*H_a), 2.87 (dd, *J* = 14.3, 7.9 Hz, 1 H, CH-CHH_a), 2.79 (dd, *J* = 13.7, 7.9 Hz, 1 H, CH-CHH_b), 2.35 (s, 3 H, $CH_{3,a}$), 2.30 (s, 3 H, $CH_{3,b}$), 2.28 (s, 3 H, $CH_{3,b}$), 2.24 (s, 3 H, $CH_{3,a}$), 2.06 (s, 6 H, 2 × $CH_{3,a}$), 2.06 (s, br, 6 H, 2 × $CH_{3,b}$); ^{13}C{^{1}H} NMR (101 MHz, $CDCl_3$)* δ 201.8 (C_b=O), 201.7 (C_a=O), 137.1 (Ar-$C_{q,b}$-CH_2), 136.9 (Ar-$C_{q,b}$-CH), 135.6 (Ar-$C_{q,b}$-CH_3), 130.9 (2 × Ar-CH_a), 129.9 (br, 3 × Ar-$C_{q,b}$-CH_3), 129.6 (2 × Ar-CH_a), 129.04 (2 × Ar-CH_a), 128.97 (2 × Ar-CH_b), 128.9 (4 × Ar-CH_b), 59.0 ((C=O)-CH_a), 57.1 ((C=O)-CH_b), 33.7 (CH-$CH_{2,b}$), 30.3 (CH-$CH_{2,a}$), 21.0 (Ar-$CH_{3,b}$), 20.81 (Ar-$CH_{3,b}$), 20.79 (Ar-$CH_{3,a}$), 20.7 (2 × Ar-$CH_{3,b}$), 20.6 (Ar-$CH_{3,a}$), 20.0 (2 × Ar-$CH_{3,a}$); HRMS (TOF +ESI) *m/z* calcd for $C_{19}H_{23}O$ $[M+H]^+$: 267.1749, found: 267.1745.

Oxetane **10**: R_f = 0.47 (4% Et_2O/pentane); IR (film)/cm^{-1} 2952, 2870, 1613, 1509, 1453, 1379, 1036, 991, 943, 849, 816, 719; ^{1}H NMR (400 MHz, $CDCl_3$) δ 7.41–7.34 (m, 2 H, 2 × Ar-CH), 7.19–7.12 (m, 2 H, 2 × Ar-CH), 6.90 (s, 2 H, 2 × Ar-CH), 5.40 (d, *J* = 5.9 Hz, 2 H, C*H*HOC*H*H), 4.81 (d, *J* = 5.9 Hz, 2 H, CH*H*OCH*H*), 2.36 (s, 3 H, CH_3), 2.33 (s, 3 H, CH_3), 1.87 (s, 6 H, 2 × CH_3); ^{13}C{^{1}H} NMR (101 MHz, $CDCl_3$) δ 141.9 (Ar-C_q-C_q), 138.0 (Ar-C_q-C_q), 136.6 (Ar-C_q-CH_3), 136.2 (2 × Ar-C_q-CH_3), 136.1 (Ar-C_q-CH_3), 129.7 (2 × Ar-CH), 129.3 (2 × Ar-CH), 125.6 (2 × Ar-CH), 85.3 (CH_2OCH_2), 52.3 (C_q), 21.0 (Ar-CH_3), 20.7 (Ar-CH_3), 20.2 (2 × Ar-CH_3); HRMS (FTMS +APCI) *m/z* calcd for $C_{19}H_{23}O^+$ $[M+H]^+$: 267.1743, found: 267.1732.

* The Ar-$C_{q,}$ signals for **10a** were not observed due to the small amount of **10a** in the mixture.

3-(4-Fluorophenyl)-3-mesityloxetane (11), 2-(4-fluorophenyl)-3-mesitylpropanal (11a) and **3-(4-fluorophenyl)-2-mesitylpropanal (11b)**

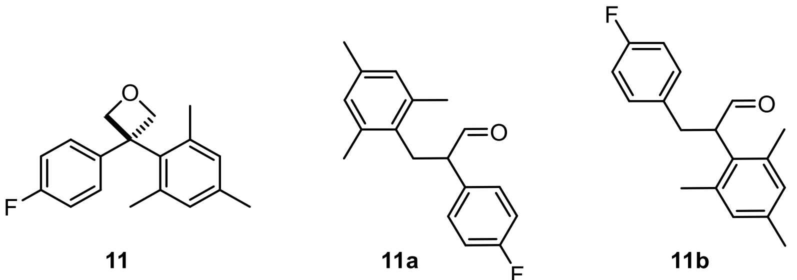

11 **11a** **11b**

$FeCl_3$ (8.1 mg, 0.05 mmol) was weighed in a vial. Oxetanol **1c** (92.3 mg, 0.50 mmol) and mesitylene (350 μL, 2.50 mmol) were added. The reaction vessel was sealed and HFIP (5.0 mL) was added. The reaction was stirred for 18 h at 80 °C, then cooled to rt and sat. aq. $NaHCO_3$ (20 mL) was added followed by Et_2O (15 mL). The layers were separated, and the aqueous portion was extracted with Et_2O (2 × 15 mL). The organic extracts were combined, dried over Na_2SO_4, filtered and concentrated under reduced pressure. Purification by flash chromatography (4% Et_2O/pentane) afforded a mixture of aldehyde **11a** and **11b** (7:93, 10.0 mg, 7%) as a yellow oil, followed by oxetane **11** (109 mg, 81%) as a yellow oil.

Aldehyde **11a** and **11b**: R_f = 0.50 (4% Et_2O/pentane); IR (film)/cm^{-1} 2952, 2922, 2866, 1722 (C=O), 1602, 1509, 1483, 1449, 1222, 1159, 853, 782; ^{1}H NMR (400 MHz, $CDCl_3$) δ 9.81 (d, *J* = 1.4 Hz, 1 H, H_aC=O), 9.77 (s, 1 H, H_bC=O), 7.04 (s, 2 H, 2 × Ar-CH_a), 7.02 (s, 2 H, 2 × Ar-CH_a), 6.97–6.82 (m, 6 H, 6 × Ar-CH_b), 6.79 (s, 2 H, 2 × Ar-CH_a), 3.91 (dd, *J* = 8.3, 4.9 Hz, 1 H, (C=O)-CH_b), 3.70–3.66 (m, 1 H, (C=O)-CH_a), 3.63 (dd, *J* = 13.8, 4.9 Hz, 1 H, CH-C*H*H_b), 3.47 (dd, *J* = 14.2, 5.6 Hz, 1 H, CH-C*H*H_a), 2.93 (dd, *J* = 14.2, 8.3 Hz, 1 H, CH-CHH_a), 2.82 (dd, *J* = 13.8, 8.3 Hz, 1 H, CH-CHH_b), 2.28 (s, 3 H, $CH_{3,b}$), 2.24 (s, 3 H, $CH_{3,a}$), 2.10 (s, 6 H, 2 × $CH_{3,a}$), 2.05 (s, br, 6 H, 2 × $CH_{3,b}$); ^{13}C{^{1}H} NMR (101 MHz, $CDCl_3$)† δ 201.5 (C_b=O), 200.8 (C_a=O), 161.5 (d, ^1J = 244.0 Hz, Ar-$C_{q,b}$-F), 137.4 (Ar-$C_{q,a}$-CH_2), 137.3 (Ar-$C_{q,b}$-CH), 136.4 (2 × Ar-$C_{q,a}$-CH_3), 135.5 (d, ^4J = 3.2 Hz, Ar-$C_{q,b}$-CH_2), 135.4 (d, ^4J = 2.7 Hz, Ar-$C_{q,a}$-CH), 130.6 (d, ^3J = 7.8 Hz, 2 × Ar-CH_b), 130.2 (2 × Ar-CH_b), 130.0 (br, 3 × Ar-$C_{q,b}$-CH_3), 129.1 (2 × Ar-CH_a), 115.9 (d, ^2J = 21.9 Hz, 2 × Ar-CH_a), 115.0 (d, ^2J = 21.4 Hz, 2 × Ar-CH_b), 58.4 ((C=O)-CH_a), 57.0 ((C=O)-CH_b), 33.2 (CH-$CH_{2,b}$), 30.3 (CH-$CH_{2,a}$), 20.80 (Ar-$CH_{3,b}$), 20.78 (Ar-$CH_{3,a}$), 20.6 (br, 2 × Ar-$CH_{3,b}$), 20.0 (2 × Ar-$CH_{3,a}$); ^{19}F{^{1}H} NMR (377 MHz, $CDCl_3$) δ –114.6 (F_a), –117.1 (F_b); HRMS (FTMS +APCI) *m/z* calcd for $C_{18}H_{18}FO^{+\bullet}$ $[M–H]^{+\bullet}$: 269.1336, found: 269.1345.

Oxetane **11**: R_f = 0.44 (4% Et_2O/pentane); IR (film)/cm^{-1} 2955, 2873, 1632, 1505, 1453, 1222, 1162, 1036, 991, 834; ^{1}H NMR (400 MHz, $CDCl_3$) δ 7.50–7.42 (m, 2 H, 2 × Ar-CH), 7.07–6.99 (m, 2 H, 2 × Ar-CH), 6.91 (s, 2 H, 2 × Ar-CH), 5.41 (dd, *J* = 5.0, 1.1 Hz, 2 H, C*H*HOC*H*H), 4.79 (dd, *J* = 5.0, 1.2 Hz, 2 H, CH*H*OCH*H*), 2.33 (s, 3 H, CH_3), 1.87 (s, 6 H, 2 × CH_3); ^{13}C{^{1}H} NMR (101 MHz, $CDCl_3$) δ 162.0 (d, ^1J = 245.4 Hz, Ar-C_q-F), 140.7 (d, ^4J = 3.3 Hz, Ar-C_q-C_q), 137.5 (Ar-C_q-C_q), 136.4 (Ar-C_q-CH_3), 136.1 (2 × Ar-C_q-CH_3), 129.8 (2 × Ar-CH), 127.4 (d, ^3J = 7.8 Hz, 2 × Ar-CH), 115.3 (d, ^2J = 21.1 Hz, 2 × Ar-CH), 85.2 (CH_2OCH_2), 52.3 (C_q), 20.7 (Ar-CH_3), 20.1 (2 × Ar-CH_3); ^{19}F{^{1}H} NMR (377 MHz, $CDCl_3$) δ –116.2; HRMS (FTMS +APCI) *m/z* calcd for $C_{18}H_{18}FO^+$ $[M–H]^+$: 269.1336, found: 269.1344.

† The signal for Ar-$C_{q,a}$-F, 2 × Ar-CH_a and Ar-$C_{q,a}$-CH_3 were not observed due to the small amount of **11a** in the mixture.

3-(4-Chlorophenyl)-3-mesityloxetane (12), 2-(4-chlorophenyl)-3-mesitylpropanal (12a) and **3-(4-chlorophenyl)-2-mesitylpropanal (12b)**

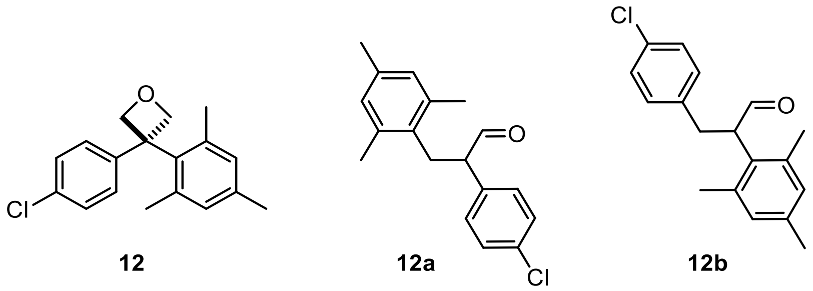

12 **12a** **12b**

$FeCl_3$ (8.1 mg, 0.05 mmol) was weighed in a vial. Oxetanol **1d** (92.3 mg, 0.50 mmol) and mesitylene (350 µL, 2.50 mmol) were added. The reaction vessel was sealed and HFIP (5.0 mL) was added. The reaction was stirred for 18 h at 80 °C, then cooled to rt and sat. aq. $NaHCO_3$ (20 mL) was added followed by Et_2O (15 mL). The layers were separated, and the aqueous portion was extracted with Et_2O (2 × 15 mL). The organic extracts were combined, dried over Na_2SO_4, filtered and concentrated under reduced pressure. Purification by flash chromatography (4% Et_2O/pentane) afforded a mixture of aldehyde **12a** and **12b** (39:61, 10.3 mg, 7%) as a yellow oil, followed by oxetane **12** (115 mg, 80%) as a yellow oil.

Aldehyde **12a** and **12b**: R_f = 0.59 (4% Et_2O/pentane); IR (film)/cm^{-1} 2955, 2922, 2862, 2810, 2717, 1725 (C=O), 1610, 1490, 1092, 1013, 853, 820, 719; ^1H NMR (400 MHz, $CDCl_3$) δ 9.80 (d, *J* = 1.4 Hz, 1 H, H_aC=O), 9.75 (s, 1 H, H_bC=O), 7.34–7.28 (m, 2 H, 2 × Ar-CH_a), 7.19–7.13 (m, 2 H, 2 × Ar-CH_b), 7.03–6.97 (m, 2 H, 2 × Ar-CH_a), 6.95–6.89 (m, 2 H, 2 × Ar-CH_b), 6.86 (s, 2 H, 2 × Ar-CH_b), 6.79 (s, 2 H, 2 × Ar-CH_a), 3.91 (dd, *J* = 8.3, 4.9 Hz, 1 H, (C=O)-CH_b), 3.70–3.65 (m, 1 H, (C=O)-CH_a), 3.62 (dd, *J* = 13.7, 4.9 Hz, 1 H, CH-C*H*H_b), 3.47 (dd, *J* = 14.2, 5.6 Hz, 1 H, CH-C*H*H_a), 2.87 (dd, *J* = 14.2, 8.3 Hz, 1 H, CH-CHH_a), 2.81 (dd, *J* = 13.7, 8.3 Hz, 1 H, CH-CHH_b), 2.28 (s, 3 H, $CH_{3,b}$), 2.24 (s, 3 H, $CH_{3,a}$), 2.10 (s, 6 H, 2 × $CH_{3,a}$), 2.00 (s, br, 6 H, 2 × $CH_{3,b}$); $^{13}C\{^1H\}$ NMR (101 MHz, $CDCl_3$) δ 201.3 (C_b=O), 199.8 (C_a=O), 138.4 (Ar-$C_{q,b}$-CH_2), 137.4 (Ar-$C_{q,b}$-CH), 136.4 (2 × Ar-$C_{q,a}$-CH_3), 135.8 (Ar-$C_{q,a}$-CH_2), 135.1 (Ar-$C_{q,a}$-CH), 133.7 (Ar-$C_{q,a}$-Cl), 132.2 (Ar-$C_{q,a}$-CH_3), 132.0 (Ar-$C_{q,b}$-Cl), 130.5 (2 × Ar-CH_b), 130.4 (2 × Ar-CH_a), 130.1 (2 × Ar-CH_b), 130.0 (br, 3 × Ar-$C_{q,b}$-CH_3), 129.2 (2 × Ar-CH_a), 129.1 (2 × Ar-CH_a), 128.3 (2 × Ar-CH_b), 58.6 ((C=O)-CH_a), 56.8 ((C=O)-CH_b), 33.4 (CH-$CH_{2,b}$), 30.3 (CH-$CH_{2,a}$), 20.80 (Ar-$CH_{3,b}$), 20.78 (Ar-$CH_{3,a}$), 20.6 (br, 2 × Ar-$CH_{3,b}$), 20.0 (2 × Ar-$CH_{3,a}$); HRMS (+APCI) *m/z* calcd for $C_{18}H_{18}{}^{35}ClO^{+\bullet}$ $[M–H]^{+\bullet}$: 285.1041, found: 285.1044.

Oxetane **12**: R_f = 0.53 (4% Et_2O/pentane); IR (film)/cm^{-1} 2952, 2870, 1610, 1490, 1401, 1095, 1013, 849, 827, 771, 745, 674; ^1H NMR (400 MHz, $CDCl_3$) δ 7.46–7.38 (m, 2 H, 2 × Ar-CH), 7.34–7.28 (m, 2 H, 2 × Ar-CH), 6.90 (s, 2 H, 2 × Ar-CH), 5.39 (dd, *J* = 5.0, 1.1 Hz, 2 H, C*H*HOC*H*H), 4.77 (dd, *J* = 5.0, 1.2 Hz, 2 H, CH*H*OCH*H*), 2.32 (s, 3 H, CH_3), 1.85 (s, 6 H, 2 × CH_3); $^{13}C\{^1H\}$ NMR (101 MHz, $CDCl_3$) δ 143.5 (Ar-C_q-C_q), 137.2 (Ar-C_q-C_q), 136.5 (Ar-C_q-CH_3), 136.1 (2 × Ar-C_q-CH_3), 132.9 (Ar-C_q-Cl), 129.9 (2 × Ar-CH), 128.7 (2 × Ar-CH), 127.2 (2 × Ar-CH), 85.0 (CH_2OCH_2), 52.3 (C_q), 20.7 (Ar-CH_3), 20.1 (2 × Ar-CH_3); HRMS (FTMS +APCI) *m/z* calcd for $C_{18}H_{20}{}^{35}ClO^+$ $[M+H]^+$: 287.1197, found: 287.1192.

3-(4-Bromophenyl)-3-mesityloxetane (13), 2-(4-bromophenyl)-3-mesitylpropanal (13a) and **3-(4-bromophenyl)-2-mesitylpropanal (13b)**

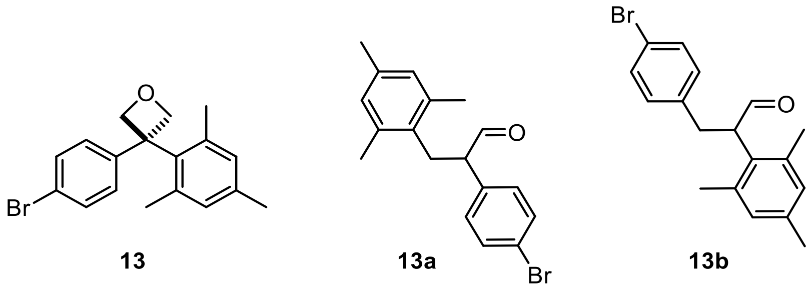

13 **13a** **13b**

$FeCl_3$ (8.1 mg, 0.05 mmol) was weighed in a vial. Oxetanol **1e** (114 mg, 0.50 mmol) and mesitylene (350 µL, 2.50 mmol) were added. The reaction vessel was sealed and HFIP (5.0 mL) was added. The reaction was stirred for 18 h at 80 °C, then cooled to rt and sat. aq. $NaHCO_3$ (20 mL) was added followed by Et_2O (15 mL). The layers were separated, and the aqueous portion was extracted with Et_2O (2 × 15 mL). The organic extracts were combined, dried over Na_2SO_4, filtered and concentrated under reduced pressure. Purification by flash chromatography (4% Et_2O/pentane) afforded a mixture of aldehyde **13a** and **13b** (38:62, 10.2 mg, 6%) as a yellow oil, followed by oxetane **13** (125 mg, 76%) as a yellow oil.

Aldehyde **13a** and **13b**: R_f = 0.40 (4% Et_2O/pentane); IR (film)/cm^{-1} 2918, 2862, 2810, 2713, 1722 (C=O), 1610, 1487, 1446, 1401, 1073, 1010, 853, 812, 711; ^{1}H NMR (400 MHz, $CDCl_3$) δ 9.79 (d, *J* = 1.4 Hz, 1 H, H_aC=O), 9.75 (s, 1 H, H_bC=O), 7.49–7.43 (m, 2 H, 2 × Ar-CH_a), 7.34–7.28 (m, 2 H, 2 × Ar-CH_b), 6.98–6.92 (m, 2 H, 2 × Ar-CH_a), 6.90–6.82 (m, 4 H, 4 × Ar-CH_b), 6.79 (s, 2 H, 2 × Ar-CH_a), 3.91 (dd, *J* = 8.2, 4.9 Hz, 1 H, (C=O)-CH_b), 3.68–3.63 (m, 1 H, (C=O)-CH_a), 3.61 (dd, *J* = 13.7, 4.9 Hz, 1 H, CH-C*H*H_b), 3.47 (dd, *J* = 14.2, 5.7 Hz, 1 H, CH-C*H*H_a), 2.88 (dd, *J* = 14.2, 8.3 Hz, 1 H, CH-CHH_a), 2.79 (dd, *J* = 13.7, 8.2 Hz, 1 H, CH-CHH_b), 2.28 (s, 3 H, $CH_{3,b}$), 2.24 (s, 3 H, $CH_{3,a}$), 2.12–1.95 (m, 12 H, 4 × $CH_{3(a+b)}$); $^{13}C\{^1H\}$ NMR (101 MHz, $CDCl_3$) δ 201.3 (C_b=O), 199.7 (C_a=O), 138.9 (Ar-$C_{q,b}$-CH_2), 137.4 (Ar-$C_{q,b}$-CH), 136.4 (Ar-$C_{q,a}$-CH_2), 135.8 (Ar-$C_{q,a}$-CH), 135.6 (Ar-$C_{q,a}$-CH_3), 132.4 (2 × Ar-$C_{q,a}$-CH_3), 132.1 (2 × Ar-$C_{q,b}$-CH_3), 132.0 (2 × Ar-CH_a), 131.3 (2 × Ar-CH_b), 130.9 (2 × Ar-CH_b), 130.7 (2 × Ar-CH_a), 130.1 (2 × Ar-CH_b and Ar-$C_{q,b}$-CH_3), 129.2 (2 × Ar-CH_a), 121.7 (Ar-$C_{q,a}$-Br), 120.3 (Ar-$C_{q,b}$-Br), 58.6 ((C=O)-CH_a), 56.7 ((C=O)-CH_b), 33.4 (CH-$CH_{2,b}$), 30.3 (CH-$CH_{2,a}$), 20.80 (Ar-$CH_{3,b}$), 20.78 (Ar-$CH_{3,a}$), 20.6 (2 × Ar-$CH_{3,b}$), 20.0 (2 × Ar-$CH_{3,a}$); HRMS (FTMS +APCI) *m/z* calcd. for $C_{18}H_{20}{}^{79}BrO^+$ $[M+H]^+$: 331.0692, found: 331.0707.

Oxetane **13**: R_f = 0.36 (4% Et_2O/pentane); IR (film)/cm^{-1} 2952, 2870, 1487, 1387, 1036, 987, 943, 846, 767, 730; ^{1}H NMR (400 MHz, $CDCl_3$) δ 7.51–7.42 (m, 2 H, 2 × Ar-CH), 7.41–7.33 (m, 2 H, 2 × Ar-CH), 6.90 (s, 2 H, 2 × Ar-CH), 5.40 (d, *J* = 5.1 Hz, 2 H, C*H*HOC*H*H), 4.77 (d, *J* = 5.1 Hz, 2 H, CH*H*OCH*H*), 2.33 (s, 3 H, CH_3), 1.86 (s, 6 H, 2 × CH_3); $^{13}C\{^1H\}$ NMR (101 MHz, $CDCl_3$) δ 144.1 (Ar-C_q-C_q), 137.1 (Ar-C_q-C_q), 136.5 (Ar-C_q-CH_3), 136.1 (2 × Ar-C_q-CH_3), 131.7 (2 × Ar-CH), 129.9 (2 × Ar-CH), 127.6 (2 × Ar-CH), 121.0 (Ar-C_q-Br), 84.9 (CH_2OCH_2), 52.4 (C_q), 20.7 (Ar-CH_3), 20.1 (2 × Ar-CH_3); HRMS (FTMS +APCI) *m/z* calcd for $C_{18}H_{20}{}^{79}BrO^+$ $[M+H]^+$: 331.0692, found: 331.0698.

3-(4-Iodophenyl)-3-mesityloxetane (14), 2-(4-iodophenyl)-3-mesitylpropanal (14a) and **3-(4-iodophenyl)-2-mesitylpropanal (14b)**

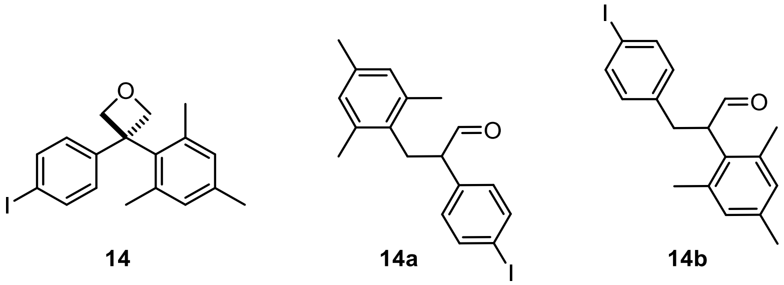

14 **14a** **14b**

$FeCl_3$ (8.1 mg, 0.05 mmol) was weighed in a vial. Oxetanol **1f** (138 mg, 0.50 mmol) and mesitylene (350 µL, 2.50 mmol) were added. The reaction vessel was sealed and HFIP (5.0 mL) was added. The reaction was stirred for 18 h at 80 °C, then cooled to rt and sat. aq. $NaHCO_3$ (20 mL) was added followed by Et_2O (15 mL). The layers were separated, and the aqueous portion was extracted with Et_2O (2 × 15 mL). The organic extracts were combined, dried over Na_2SO_4, filtered and concentrated under reduced pressure. Purification by flash chromatography (4% Et_2O/pentane) afforded a mixture of aldehyde **14a** and **14b** (53:47, 16.4 mg, 9%) as a yellow oil, followed by oxetane **14** (148 mg, 78%) as a yellow oil.

The reaction was also scaled to 4.0 mmol and performed in a round bottom flask: $FeCl_3$ (64.9 mg, 0.40 mmol), oxetanol **1f** (1.10 g, 4.00 mmol), mesitylene (2.8 mL, 20.0 mmol) and HFIP (40 mL). Purification by flash chromatography (4% Et_2O/pentane) afforded aldehyde **14b** (13.8 mg, 0.9%) as a yellow oil, followed by oxetane **14** (1.04 g, 69%) as a yellow sticky solid.

Aldehyde **14a**‡: R_f = 0.29 (4% Et_2O/pentane); IR (film)/cm^{-1} 2955, 2918, 2862, 2814, 2717, 1725 (C=O), 1483, 1010, 853, 812; ^{1}H NMR (400 MHz, $CDCl_3$) δ 9.79 (d, *J* = 1.4 Hz, 1 H, HC=O), 7.70–7.61 (m, 2 H, 2 × Ar-CH), 6.85–6.81 (m, 2 H, 2 × Ar-CH), 6.80 (s, 2 H, 2 × Ar-CH), 3.66–3.62 (m, 1 H, (C=O)-CH), 3.47 (dd, *J* = 14.2, 5.7 Hz, 1 H, CH-C*H*H), 2.87 (dd, *J* = 14.2, 8.3 Hz, 1 H, CH-CH*H*), 2.24 (s, 3 H, CH_3), 2.06 (s, 6 H, 2 × CH_3); ^{13}C{^{1}H} NMR (101 MHz, $CDCl_3$) δ 199.7 (C=O), 138.0 (2 × Ar-CH), 136.3 (2 × Ar-C_q-CH_3), 136.2 (Ar-C_q-CH_2), 135.8 (Ar-C_q-CH), 132.1 (Ar-C_q-CH_3), 131.0 (2 × Ar-CH), 129.2 (2 × Ar-CH), 93.2 (Ar-C_q-I), 58.7 ((C=O)-*C*H), 30.2 (CH-*C*H_2), 20.8 (Ar-CH_3), 20.0 (2 × Ar-CH_3); HRMS (+APCI) *m/z* calcd for $C_{18}H_{18}IO^{+\bullet}$ $[M–H]^{+\bullet}$: 377.0397, found: 377.0390.

Aldehyde **14b**: R_f = 0.29 (4% Et_2O/pentane); IR (film)/cm^{-1} 2918, 2863, 2713, 1710 (C=O), 1483, 1446, 1401, 1062, 1006, 853, 808, 708; ^{1}H NMR (400 MHz, $CDCl_3$) δ 9.74 (s, 1 H, HC=O), 7.54–7.48 (m, 2 H, 2 × Ar-CH), 6.86 (s, 2 H, 2 × Ar-CH), 6.77–6.70 (m, 2 H, 2 × Ar-CH), 3.91 (dd, *J* = 8.2, 4.9 Hz, 1 H, (C=O)-CH), 3.60 (dd, *J* = 13.7, 4.9 Hz, 1 H, CH-C*H*H), 2.77 (dd, *J* = 13.7, 8.2 Hz, 1 H, CH-CH*H*), 2.28 (s, 3 H, CH_3), 2.04 (s, 6 H, 2 × CH_3); ^{13}C{^{1}H} NMR (101 MHz, $CDCl_3$) δ 201.3 (C=O), 139.6 (Ar-C_q-CH_2), 137.4 (Ar-C_q-CH), 137.3 (2 × Ar-CH), 131.2 (2 × Ar-CH), 130.1 (2 × Ar-CH), 130.0 (br, 3 × Ar-C_q-CH_3), 91.4 (Ar-C_q-I), 56.7 ((C=O)-*C*H), 33.5 (CH-*C*H_2), 20.8 (Ar-CH_3), 20.6 (2 × Ar-CH_3). HRMS (FTMS +APCI) *m/z* calcd for $C_{18}H_{20}IO^+$ $[M+H]^+$: 379.0553, found: 379.0544.

Oxetane **14**: R_f = 0.26 (4% Et_2O/pentane); mp = 41–42 °C; IR (film)/cm^{-1} 2962, 2870, 1610, 1476, 1453, 1073, 1032, 987, 943, 842, 764; ^{1}H NMR (400 MHz, $CDCl_3$) δ 7.71–7.61 (m, 2 H, 2 × Ar-CH), 7.29–7.20 (m, 2 H, 2 × Ar-CH), 6.90 (s, 2 H, 2 × Ar-CH), 5.40 (dd, *J* = 5.0 , 1.1 Hz, 2 H, C*H*HOC*H*H), 4.76 (dd, *J* = 5.0, 1.3 Hz, 2 H, CH*H*OCH*H*), 2.33 (s, 3 H, CH_3), 1.85 (s, 6 H, 2 × CH_3); ^{13}C{^{1}H} NMR (101 MHz, $CDCl_3$) δ 144.8 (Ar-C_q-C_q), 137.7 (2 × Ar-CH), 137.0 (Ar-C_q-C_q), 136.5 (Ar-C_q-CH_3), 136.1 (2 × Ar-C_q-CH_3), 129.8 (2 × Ar-CH), 127.9 (2 × Ar-CH), 92.6 (Ar-C_q-I), 84.8 (*C*H_2O*C*H_2), 52.4 (C_q), 20.7 (Ar-CH_3), 20.1 (2 × Ar-CH_3); HRMS (FTMS +APCI) *m/z* calcd for $C_{18}H_{20}IO^+$ $[M+H]^+$: 379.0553, found: 379.0571.

‡ Assigned comparing the ^{1}H and ^{13}C NMR spectra of aldehyde **14b** with the mixture of **14a**/**14b**.

3-Mesityl-3-(4-methoxyphenyl)oxetane (15), 3-mesityl-2-(4-methoxyphenyl)propanal (15a) and **2-mesityl-3-(4-methoxyphenyl)propanal (15b)**

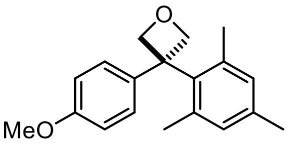

15

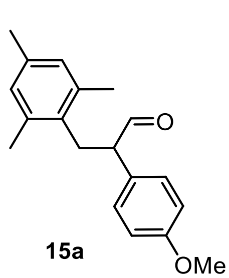

15a

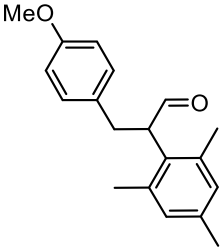

15b

$FeCl_3$ (8.1 mg, 0.05 mmol) was weighed in a vial. *p*-Methoxyphenol-oxetanol **1g** (90.0 mg, 0.50 mmol) and mesitylene (350 µL, 2.50 mmol) were added. The reaction vessel was sealed and HFIP (5.0 mL) was added. The reaction was stirred for 18 h at 80 °C, then cooled to rt and sat. aq. $NaHCO_3$ (20 mL) was added followed by Et_2O (15 mL). The layers were separated, and the aqueous portion was extracted with Et_2O (2 × 15 mL). The organic extracts were combined, dried over Na_2SO_4, filtered and concentrated under reduced pressure. Purification by flash chromatography (4% Et_2O/pentane) afforded a mixture of aldehyde **15a** and **15b** (20:80, 3.2 mg, 2%) as a yellow oil, followed by oxetane **15** (87.9 mg, 62%) as a colourless paste.

Aldehyde **15a** and **15b**: R_f = 0.38 (4% Et_2O/pentane); IR (film)/cm^{-1} 2952, 1722 (C=O), 1610, 1513, 1300, 1244, 1177, 1036, 849, 827; ^{1}H NMR (400 MHz, $CDCl_3$) δ 9.78 (d, *J* = 1.5 Hz, 1 H, H_aC=O), 9.77 (s, 1 H, H_bC=O), 7.00–6.97 (m, 2 H, 2 × Ar-CH_a), 6.93–6.89 (m, 2 H, 2 × Ar-CH_b), 6.89–6.87 (m, 2 H, 2 × Ar-CH_a), 6.85 (s, 2 H, 2 × Ar-CH_b), 6.78 (s, 2 H, 2 × Ar-CH_a), 6.76–6.71 (m, 2 H, 2 × Ar-CH_b), 3.91 (dd, *J* = 8.1, 5.0 Hz, 1 H, (C=O)-CH_b), 3.81 (s, 3 H, $OCH_{3,a}$), 3.77 (s, 3 H, $OCH_{3,b}$), 3.65–3.60 (m, 1 H, (C=O)-CH_a), 3.60 (dd, *J* = 13.7, 5.0 Hz, 1 H, CH-C*H*H_b), 3.47 (dd, *J* = 14.2, 5.6 Hz, 1 H, CH-C*H*H_a), 2.86 (dd, *J* = 14.2, 8.2 Hz, 1 H, CH-CHH_a), 2.78 (dd, *J* = 13.7, 8.1 Hz, 1 H, CH-CHH_b), 2.28 (s, 3 H, $CH_{3,b}$), 2.24 (s, 3 H, $CH_{3,a}$), 2.05 (s, 6 H, 2 × $CH_{3,a}$), 2.05 (s, br, 6 H, 2 × $CH_{3,b}$); $^{13}C\{^1H\}$ NMR (101 MHz, $CDCl_3$)§ δ 201.9 (C_b=O), 153.9 (Ar-$C_{q,b}$-OCH_3), 137.1 (Ar-$C_{q,b}$-CH), 132.0 (Ar-$C_{q,b}$-CH_2), 130.1 (4 × Ar-CH_b), 129.9 (br, 3 × Ar-$C_{q,b}$-CH_3), 113.6 (2 × Ar-CH_b), 57.2 ((C=O)-CH_b), 55.2 ($OCH_{3,b}$), 33.2 (CH-$CH_{2,b}$), 20.8 (Ar-$CH_{3,b}$), 20.7 (2 × Ar-$CH_{3,b}$); HRMS (FTMS +APCI) *m/z* calcd for $C_{19}H_{23}O_2^+$ $[M+H]^+$: 283.1693, found: 283.1703.

Oxetane **15**: R_f = 0.30 (4% Et_2O/pentane); IR (film)/cm^{-1} 2952, 2870, 1610, 1509, 1461, 1297, 1248, 1185, 1036, 991, 943, 831; ^{1}H NMR (400 MHz, $CDCl_3$) δ 7.43–7.36 (m, 2 H, 2 × Ar-CH), 6.91–6.83 (m, 4 H, 4 × Ar-CH), 5.39 (dd, *J* = 5.0, 1.1 Hz, 2 H, C*H*HOC*H*H), 4.79 (dd, *J* = 5.0, 1.2 Hz, 2 H, CH*H*OCH*H*), 3.81 (s, 3 H, OCH_3), 2.32 (s, 3 H, CH_3), 1.87 (s, 6 H, 2 × CH_3); $^{13}C\{^1H\}$ NMR (101 MHz, $CDCl_3$) δ 158.6 (Ar-C_q-OCH_3), 138.0 (Ar-C_q-C_q), 137.0 (Ar-C_q-C_q), 136.2 (Ar-C_q-CH_3), 136.1 (2 × Ar-C_q-CH_3), 129.7 (2 × Ar-CH), 126.9 (2 × Ar-CH), 113.9 (2 × Ar-CH), 85.4 (CH_2OCH_2), 55.2 (OCH_3), 52.1 (C_q), 20.7 (Ar-CH_3), 20.2 (2 × Ar-CH_3); HRMS (TOF +ESI) *m/z* calcd for $C_{21}H_{26}NO_2$ [M+MeCN+H]: 324.1964, found: 324.1959.

3-Mesityl-3-(3-methoxyphenyl)oxetane (16)

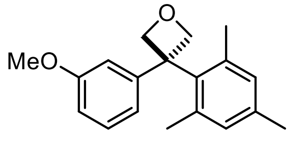

$FeCl_3$ (8.1 mg, 0.05 mmol) was weighed in a vial. *m*-Methoxyphenol-oxetanol **1h** (90.0 mg, 0.50 mmol) and mesitylene (350 µL, 2.50 mmol) were added. The reaction vessel was sealed and HFIP (5.0 mL) was added. The reaction was stirred for 18 h at 80 °C, then cooled to rt and sat. aq. $NaHCO_3$ (20 mL) was added followed by Et_2O (15 mL). The layers were separated, and the aqueous portion was extracted with Et_2O (2 × 15 mL). The organic extracts were combined, dried over Na_2SO_4, filtered and concentrated under reduced pressure. Purification by flash chromatography (8% Et_2O/pentane) afforded oxetane **16** (110 mg, 78%) as a yellow solid. R_f = 0.42 (10% Et_2O/pentane); mp = 112–113 °C; IR (film)/cm^1 2955, 2870, 1606, 1483, 1461, 1293, 1244, 1166, 1051, 991, 853, 782, 700; ^{1}H NMR (400 MHz, $CDCl_3$) δ 7.30–7.21 (m, 1 H, Ar-CH), 7.11–7.08 (m, 1 H, Ar-CH), 7.06 (ddd, *J* = 7.8, 1.8, 0.9 Hz, 1 H, Ar-CH), 6.88 (s, 2 H, 2 × Ar-CH), 6.83 (dd, *J* = 2.6, 0.9 Hz, 1 H, Ar-CH), 5.44–5.35 (m, 2 H, C*H*HOC*H*H), 4.86–4.78 (m, 2 H, CH*H*OCH*H*), 3.80 (s, 3 H, OCH_3), 2.31 (s, 3 H, CH_3), 1.87 (s, 6 H, 2 × CH_3); $^{13}C\{^1H\}$ NMR (101 MHz, $CDCl_3$) δ 160.0 (Ar-C_q-OCH_3),

§ Only signals for **15b** could be attributed due to the low amount of material.

146.8 (Ar-C_q-C_q), 137.7 (Ar-C_q-C_q), 136.20 (Ar-C_q-CH_3), 136.16 (2 × Ar-C_q-CH_3), 129.8 (2 × Ar-CH), 129.5 (Ar-CH), 118.3 (Ar-CH), 112.1 (Ar-CH), 111.8 (Ar-CH), 85.0 (CH_2OCH_2), 55.2 (OCH_3), 52.7 (C_q), 20.7 (Ar-CH_3), 20.2 (2 × Ar-CH_3); HRMS (TOF +ESI) *m/z* calcd for $C_{21}H_{26}NO_2$ [M+MeCN+H]: 324.1964, found: 324.1957.

3-(3-Bromophenyl)-3-mesityloxetane (17)

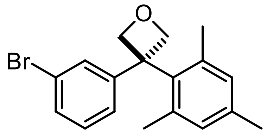

$FeCl_3$ (8.1 mg, 0.05 mmol) was weighed in a vial. Oxetanol **1i** (115 mg, 0.50 mmol) and mesitylene (350 µL, 2.50 mmol) were added. The reaction vessel was sealed and HFIP (5.0 mL) was added. The reaction was stirred for 18 h at 80 °C, then cooled to rt and sat. aq. $NaHCO_3$ (20 mL) was added followed by Et_2O (15 mL). The layers were separated, and the aqueous portion was extracted with Et_2O (2 × 15 mL). The organic extracts were combined, dried over Na_2SO_4, filtered and concentrated under reduced pressure. Purification by flash chromatography (4% Et_2O/pentane) afforded oxetane **17** (23.7 mg, 14%) as a yellow solid. R_f = 0.33 (4% Et_2O/pentane); mp = 91–94 °C; IR (film)/cm^{-1} 2955, 2873, 1610, 1561, 1468, 1013, 943, 853, 697; ^1H NMR (400 MHz, $CDCl_3$) δ 7.76 (t, *J* = 1.9 Hz, 1 H, Ar-CH), 7.43 (ddd, *J* = 7.9, 2.0, 1.9 Hz, 1 H, Ar-CH), 7.30 (ddd, *J* = 8.0, 1.9, 1.1 Hz, 1 H, Ar-CH), 7.22–7.14 (m, 1 H, Ar-CH), 6.90 (s, 2 H, 2 × Ar-CH), 5.43–5.32 (m, 2 H, C*H*HOC*H*H), 4.83–4.74 (m, 2 H, CH*H*OCH*H*), 2.33 (s, 3 H, CH_3), 1.86 (s, 6 H, 2 × CH_3); $^{13}C\{^1H\}$ NMR (101 MHz, $CDCl_3$) δ 147.5 (Ar-C_q-C_q), 136.9 (Ar-C_q-C_q), 136.5 (Ar-C_q-CH_3), 136.1 (2 × Ar-C_q-CH_3), 130.3 (Ar-CH), 130.1 (Ar-CH), 129.9 (2 × Ar-CH), 128.7 (Ar-CH), 124.5 (Ar-CH), 123.2 (Ar-C_q-Br), 84.8 (CH_2OCH_2), 52.6 (C_q), 20.7 (Ar-CH_3), 20.2 (2 × Ar-CH_3); HRMS (FTMS +APCI) *m/z* calcd for $C_{18}H_{20}{}^{79}BrO^+$ $[M+H]^+$: 331.0692, found: 331.0690.

2-(2-Bromophenyl)-3-mesitylpropanal (18a)

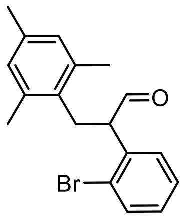

$FeCl_3$ (8.1 mg, 0.05 mmol) was weighed in a vial. Oxetanol **1j** (115 mg, 0.50 mmol) and mesitylene (350 µL, 2.50 mmol) were added. The reaction vessel was sealed and HFIP (5.0 mL) was added. The reaction was stirred for 18 h at 80 °C, then cooled to rt and sat. aq. $NaHCO_3$ (20 mL) was added followed by Et_2O (15 mL). The layers were separated, and the aqueous portion was extracted with Et_2O (2 × 15 mL). The organic extracts were combined, dried over Na_2SO_4, filtered and concentrated under reduced pressure. Purification by flash chromatography (4% Et_2O/pentane) afforded an aldehyde **18a** (77.0 mg, 46%) as a yellow oil. R_f = 0.33 (4% Et_2O/pentane); IR (film)/cm^{-1} 2955, 2918, 2862, 2817, 2728, 1725 (C=O), 1610, 1472, 1028, 853, 756; ^1H NMR (400 MHz, $CDCl_3$) δ 9.79 (d, *J* = 0.6 Hz, 1 H, HC=O), 7.57 (dd, *J* = 8.0, 1.3 Hz, 1 H, Ar-CH), 7.32 (td, *J* = 7.5, 1.3 Hz, 1 H, Ar-CH), 7.21–7.11 (m, 2 H, 2 × Ar-CH), 6.79 (s, 2 H, 2 × Ar-CH), 4.17 (dd, *J* = 8.3, 5.7 Hz, 1 H, (C=O)-CH), 3.59 (dd, *J* = 14.2, 5.7 Hz, 1 H, CH-C*H*H), 2.98 (dd, *J* = 14.2, 8.3 Hz, 1 H, CH-CH*H*), 2.24 (s, 3 H, CH_3), 2.10 (s, 6 H, 2 × CH_3); $^{13}C\{^1H\}$ NMR (101 MHz, $CDCl_3$) δ 199.7 (C=O), 136.9 (Ar-C_q-CH_2), 136.6 (2 × Ar-C_q-CH_3), 135.8 (Ar-C_q-CH), 133.3 (Ar-CH), 132.0 (Ar-C_q-CH_3), 131.1 (Ar-CH), 129.2 (Ar-CH), 129.1 (2 × Ar-CH), 127.8 (Ar-CH), 125.2 (Ar-C_q-Br), 58.1 ((C=O)-*C*H), 29.7 (CH-CH_2), 20.8 (Ar-CH_3), 19.9 (2 × Ar-CH_3); HRMS (FTMS –APCI) *m/z* calcd for $C_{18}H_{18}{}^{79}BrO^-$ $[M–H]^-$: 329.0536, found: 329.0543.

3-Mesityl-3-(4-(trifluoromethyl)phenyl)oxetane (19)

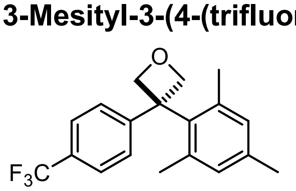

$FeCl_3$ (8.1 mg, 0.05 mmol) was weighed in a vial. Oxetanol **1k** (109 mg, 0.50 mmol) and mesitylene (350 µL, 2.50 mmol) were added. The reaction vessel was sealed and HFIP (5.0 mL) was added. The reaction was stirred for 18 h at 80 °C, then cooled to rt and sat. aq. $NaHCO_3$ (20 mL) was added followed by Et_2O (15 mL). The layers were separated, and the aqueous portion was extracted with Et_2O (2 × 15 mL). The organic extracts were combined, dried over Na_2SO_4, filtered and concentrated under reduced pressure. Purification by flash chromatography (4% Et_2O/pentane) afforded oxetane **19** (4.5 mg, 3%) as a yellow oil. R_f = 0.45 (4% Et_2O/pentane); IR (film)/cm^{-1} 2959, 2877, 1617, 1326, 1166, 1125, 1080, 1036, 842; ^1H NMR (400 MHz, $CDCl_3$) δ 7.65–7.56 (m, 4 H, 4 × Ar-CH), 6.91 (s, 2 H, 2 × Ar-CH), 5.42 (dd, *J* = 5.0, 1.3 Hz, 2 H, C*H*HOC*H*H), 4.80 (dd, *J* = 5.0, 1.3 Hz, 2 H, CH*H*OCH*H*), 2.33 (s, 3 H, CH_3), 1.85 (s, 6 H, 2 × CH_3); $^{13}C\{^1H\}$ NMR (101 MHz, $CDCl_3$)** δ 149.1 (Ar-C_q-C_q), 136.8 (Ar-C_q-C_q), 136.7 (Ar-C_q-CH_3), 136.1 (2 × Ar-C_q-CH_3), 130.0 (2 × Ar-CH),

** The signal for Ar-C_q-CF_3 and CF_3 were not observed due to the small amount of product isolated.

126.1 (2 × Ar-CH), 125.6 (d, ^3J = 3.6 Hz, 2 × Ar-CH), 84.8 (*C*H$_2$O*C*H$_2$), 52.7 (C$_q$), 20.7 (Ar-CH$_3$), 20.1 (2 × Ar-CH$_3$); ^{19}F{^{1}H} NMR (377 MHz, CDCl$_3$) δ –62.4; HRMS (FTMS +APCI) *m/z* calcd for $C_{19}H_{20}F_3O^+$ [M+H]$^+$: 321.1461, found: 321.1462.

3-Mesityl-3-(3-(trifluoromethyl)phenyl)oxetane (20)

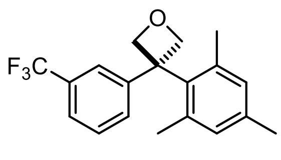

FeCl$_3$ (8.1 mg, 0.05 mmol) was weighed in a vial. Oxetanol **1p** (109 mg, 0.50 mmol) and mesitylene (350 µL, 2.50 mmol) were added. The reaction vessel was sealed and HFIP (5.0 mL) was added. The reaction was stirred for 18 h at 80 °C, then cooled to rt and sat. aq. NaHCO$_3$ (20 mL) was added followed by Et$_2$O (15 mL). The layers were separated, and the aqueous portion was extracted with Et$_2$O (2 × 15 mL). The organic extracts were combined, dried over Na$_2$SO$_4$, filtered and concentrated under reduced pressure. Purification by flash chromatography (4% Et$_2$O/pentane) afforded oxetane **20** (5.5 mg, 3%) as a white solid. R$_f$ = 0.47 (4% Et$_2$O/pentane); mp = 86–88 °C, IR (film)/cm^{-1} 2959, 2929, 2877, 1438, 1326, 1166, 1125, 1073, 805, 704; ^{1}H NMR (400 MHz, CDCl$_3$) δ 7.90 (s, 1 H, Ar-CH), 7.59–7.49 (m, 2 H, 2 × Ar-CH), 7.47–7.38 (m, 1 H, Ar-CH), 6.91 (s, 2 H, 2 × Ar-CH), 5.48–5.35 (m, 2 H, C*H*HOC*H*H), 4.85–4.74 (m, 2 H, CH*H*OCH*H*), 2.33 (s, 3 H, CH$_3$), 1.85 (s, 6 H, 2 × CH$_3$); ^{13}C{^{1}H} NMR (101 MHz, CDCl$_3$)†† δ 146.2 (Ar-*C$_q$*-C$_q$), 136.68 (Ar-*C$_q$*-C$_q$), 136.66 (Ar-*C$_q$*-CH$_3$), 136.1 (2 × Ar-*C$_q$*-CH$_3$), 131.3 (q, ^2J = 32.3 Hz, Ar-*C$_q$*-CF$_3$), 130.0 (2 × Ar-CH), 129.2 (Ar-CH), 129.0 (Ar-CH), 124.0 (q, ^3J = 3.5 Hz, Ar-CH), 122.4 (q, ^3J = 3.9 Hz, Ar-CH), 84.8 (*C*H$_2$O*C*H$_2$), 52.7 (C$_q$), 20.7 (Ar-CH$_3$), 20.1 (2 × Ar-CH$_3$); ^{19}F{^{1}H} NMR (377 MHz, CDCl$_3$) δ –62.4; HRMS (FTMS +APCI) *m/z* calcd for $C_{19}H_{20}F_3O^+$ [M+H]$^+$: 321.1472, found: 321.1461.

4-(3-(4-Bromophenyl)oxetan-3-yl)-2,6-dimethylphenol (21)

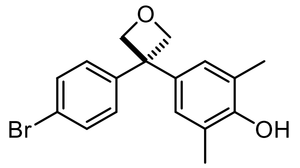

FeCl$_3$ (8.1 mg, 0.05 mmol) was weighed in a vial. Oxetanol **1e** (114 mg, 0.50 mmol) and 2,6-dimethylphenol (104 mg, 0.85 mmol) were added. The reaction vessel was sealed and HFIP (5.0 mL) was added. The reaction was stirred for 18 h at 80 °C, then cooled to rt and sat. aq. NaHCO$_3$ (20 mL) was added followed by Et$_2$O (15 mL). The layers were separated, and the aqueous portion was extracted with Et$_2$O (2 × 15 mL). The organic extracts were combined, dried over Na$_2$SO$_4$, filtered and concentrated under reduced pressure. Purification by flash chromatography (20% Et$_2$O/pentane) afforded oxetane **21** (93.9 mg, 56%) as a yellow solid. R$_f$ = 0.17 (20% Et$_2$O/pentane); mp = 164–166 °C; IR (film)/cm^{-1} 3339 (br, OH), 2959, 2877, 1487, 1394, 1312, 1203, 1077, 990, 976, 950, 905, 820, 730; ^{1}H NMR (400 MHz, CDCl$_3$) δ 7.51–7.44 (m, 2 H, 2 × Ar-CH), 7.15–7.09 (m, 2 H, 2 × Ar-CH), 6.77 (s, 2 H, 2 × Ar-CH), 5.23 (d, *J* = 5.7 Hz, 2 H, C*H*HOC*H*H), 5.14 (d, *J* = 5.7 Hz, 2 H, CH*H*OCH*H*), 4.70 (s, 1 H, OH), 2.24 (s, 6 H, 2 × CH$_3$); ^{13}C{^{1}H} NMR (101 MHz, CDCl$_3$) δ 151.0 (Ar-C$_q$-OH), 145.4 (Ar-*C$_q$*-C$_q$), 136.6 (Ar-*C$_q$*-C$_q$), 131.6 (2 × Ar-CH), 128.3 (2 × Ar-CH), 126.6 (2 × Ar-CH), 123.2 (2 × Ar-*C$_q$*-CH$_3$), 120.4 (Ar-C$_q$-Br), 84.5 (*C*H$_2$O*C*H$_2$), 50.5 (C$_q$), 16.1 (2 × Ar-CH$_3$); HRMS (+EI) *m/z* calcd for $C_{16}H_{15}{}^{79}BrO_2{}^{\bullet+}$ [M–CH$_3$+H]$^{\bullet+}$: 318.0250, found: 318.0259.

4-(3-([1,1'-Biphenyl]-4-yl)oxetan-3-yl)-2,6-dimethylphenol (22)

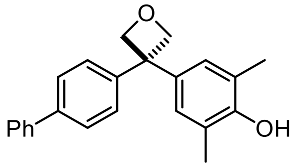

FeCl$_3$ (8.1 mg, 0.05 mmol) was weighed in a vial. Oxetanol **1m** (113 mg, 0.50 mmol) and 2,6-dimethylphenol (104 mg, 0.85 mmol) were added. The reaction vessel was sealed and HFIP (5.0 mL) was added. The reaction was stirred for 18 h at 80 °C, then cooled to rt and sat. aq. NaHCO$_3$ (20 mL) was added followed by Et$_2$O (15 mL). The layers were separated and the aqueous portion was extracted with Et$_2$O (2 × 15 mL). The organic extracts were combined, dried over Na$_2$SO$_4$, filtered and concentrated under reduced pressure. Purification by flash chromatography (25% Et$_2$O/pentane) afforded oxetane **217** (64.9 mg, 39%) as a yellow solid. R$_f$ = 0.16 (20% Et$_2$O/pentane); mp = 160–164 °C; IR (film)/cm^{-1} 3369 (br, OH), 3026, 2955, 2881, 1487, 1312, 976, 909, 838, 697; ^{1}H NMR (400 MHz, CDCl$_3$) δ 7.63–7.57 (m, 4 H, 4 × Ar-CH), 7.50–7.42 (m, 2 H, 2 × Ar-CH), 7.40–7.29 (m, 3 H, 3 × Ar-CH), 6.86 (s, 2 H, 2 × Ar-CH), 5.33–5.20 (m, 4 H, C*H$_2$*OC*H$_2$*), 4.69 (s, 1 H, OH), 2.26 (s, 6 H, 2 × CH$_3$); ^{13}C{^{1}H} NMR (101 MHz, CDCl$_3$) δ 150.9 (Ar-C$_q$-OH), 145.4 (Ar-*C$_q$*-C$_q$), 140.6 (Ar-*C$_q$*-C$_q$), 139.4 (Ar-*C$_q$*-C$_q$), 137.2 (Ar-*C$_q$*-C$_q$), 128.8 (2 × Ar-CH),

†† The signal for CF$_3$ was not observed due to the small amount of product isolated.

127.3 (Ar-CH), 127.2 (2 × Ar-CH), 127.0 (2 × Ar-CH), 126.8 (2 × Ar-CH), 126.7 (2 × Ar-CH), 123.1 (2 × Ar-C_q-CH_3), 84.8 (CH$_2$OCH$_2$), 50.7 (C_q), 16.1 (2 × Ar-CH_3); HRMS (FTMS +APCI) *m/z* calcd for $C_{23}H_{23}O_2^+$ [M+H]$^+$: 331.1693, Found: 331.1682.

2,6-Dimethyl-4-(3-(naphthalen-2-yl)oxetan-3-yl)phenol (23)

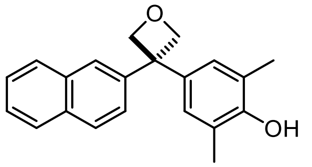

$FeCl_3$ (8.1 mg, 0.05 mmol) was weighed in a vial. Oxetanol **1n** (100 mg, 0.50 mmol) and 2,6-dimethylphenol (104 mg, 0.85 mmol) were added. The reaction vessel was sealed and HFIP (5.0 mL) was added. The reaction was stirred for 18 h at 80 °C, then cooled to rt and sat. aq. $NaHCO_3$ (20 mL) was added followed by Et_2O (15 mL). The layers were separated, and the aqueous portion was extracted with Et_2O (2 × 15 mL). The organic extracts were combined, dried over Na_2SO_4, filtered and concentrated under reduced pressure. Purification by flash chromatography (20% Et_2O/pentane) afforded oxetane **23** (63.6 mg, 42%) as a yellow solid. R_f = 0.16 (20% Et_2O/pentane); mp = 144–145 °C; IR (film)/cm^{-1} 3369 (br, OH), 3052, 2959, 2881, 1602, 1490, 1312, 1196, 980, 909, 861, 816, 749; ^{1}H NMR (400 MHz, $CDCl_3$) δ 7.90–7.75 (m, 3 H, 3 × Ar-CH), 7.59 (d, *J* = 1.9 Hz, 1 H, Ar-CH), 7.53–7.45 (m, 2 H, 2 × Ar-CH), 7.42 (dd, *J* = 8.6, 1.9 Hz, 1 H, Ar-CH), 6.88 (s, 2 H, 2 × Ar-CH), 5.34 (d, *J* = 5.7 Hz, 2 H, C*H*HOC*H*H), 5.29 (d, *J* = 5.7 Hz, 2 H, CH*H*OCH*H*), 4.71–4.66 (m, 1 H, OH), 2.24 (s, 6 H, 2 × CH_3); ^{13}C{^{1}H} NMR (101 MHz, $CDCl_3$) δ 150.9 (Ar-C_q-OH), 143.4 (Ar-C_q-C_q), 137.3 (Ar-C_q-C_q), 133.1 (Ar-C_q-C_q), 132.1 (Ar-C_q-C_q), 128.5 (Ar-CH), 127.9 (Ar-CH), 127.5 (Ar-CH), 126.8 (2 × Ar-CH), 126.3 (Ar-CH), 125.8 (Ar-CH), 125.0 (Ar-CH), 124.9 (Ar-CH), 123.1 (2 × Ar-C_q-CH_3), 84.5 (CH$_2$OCH$_2$), 51.0 (C_q), 16.1 (2 × Ar-CH_3); HRMS (TOF –EI) *m/z* calcd for $C_{21}H_{19}O_2^-$ [M–H]$^-$: 303.1385, found: 303.1394.

Triisopropyl(4-(3-phenyloxetan-3-yl)phenoxy)silane (25)

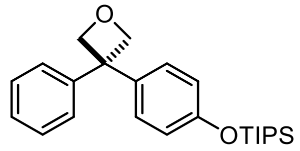

$FeCl_3$ (8.1 mg, 0.05 mmol) was weighed in a vial. Oxetanol **1a** (75 mg, 0.50 mmol) and triisopropyl(phenoxy)silane (626 mg, 2.50 mmol) were added. The reaction vessel was sealed and HFIP (5.0 mL) was added. The reaction was stirred for 18 h at 80 °C, then cooled to rt and sat. aq. $NaHCO_3$ (20 mL) was added followed by Et_2O (15 mL). The layers were separated, and the aqueous portion was extracted with Et_2O (2 × 15 mL). The organic extracts were combined, dried over Na_2SO_4, filtered and concentrated under reduced pressure. Purification by flash chromatography (2% Et_2O/pentane) afforded oxetane **25** (57 mg, 0.15 mmol, 30%) as a colourless oil. R_f = 0.26 (5% Et_2O/pentane); IR (film)/cm^{-1} 2943, 2866, 1607, 1510, 1463, 1264, 992, 913, 883, 701, 684; ^{1}H NMR (400 MHz, $CDCl_3$) δ 7.41 – 7.35 (m, 2H, 2 × Ar-CH), 7.31 – 7.29 (m, 1H, Ar-CH), 7.25 (s, 2H, 2 × Ar-CH), 7.08 (d, *J* = 8.7 Hz, 2H, 2 × Ar-CH), 6.88 (d, *J* = 8.7 Hz, 2H, 2 × Ar-CH), 5.26 (d, *J* = 5.6 Hz, 2H, C*H*HOC*H*H), 5.24 (d, *J* = 5.6 Hz, 2H, CH*H*OCH*H*), 1.36 – 1.21 (m, 3H, CH(^{i}Pr)$_3$), 1.13 (d, *J* = 7.3 Hz, 18H, CH_3). ^{13}C{^{1}H} NMR (101 MHz, $CDCl_3$) δ 154.7 (Ar-C_q), 146.1 (Ar-C_q), 138.1 (Ar-C_q), 128.5 (2 × Ar-CH), 127.5 (2 × Ar-CH), 126.5 (3 × Ar-CH), 119.8 (2 × Ar-CH), 84.9 (CH$_2$OCH$_2$), 50.9 (C_q), 17.9 (3 × CH(CH$_3$)$_2$), 12.6 (3 × (CH(CH$_3$)$_2$)); HRMS (FTMS–ESI$^+$) m/z calcd. for $C_{24}H_{35}O_2Si$ [M + H]$^+$: 383.2401; found 383.2396.

3-Phenyloxetane-3-carboxylic acid (26)

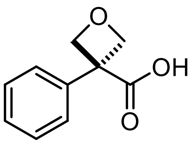

Oxetane **4** (334 mg, 1.40 mmol, 1.0 equiv), $RuCl_3$ (29.0 mg, 0.14 mmol, 0.1 equiv) and $NaIO_4$ (7.19 g, 33.6 mmol, 24.0 equiv) were added to a 100 mL round bottom flask. Then, distilled H_2O (18.7 mL), EtOAc (4.67 mL) and MeCN (4.67 mL) (H_2O:EtOAc:MeCN = 4:1:1, 0.05 M) were added sequentially and the reaction mixture was stirred at 25 °C for 18 h. Distilled water was added (50 mL) followed by EtOAc (50 mL) and the layers were separated. The aqueous phase was extracted with EtOAc (3 × 50 mL). The combined organic extracts were dried over Na_2SO_4, filtered through Celite and concentrated *in vacuo* using a rotatory evaporator. Purification by flash column chromatography (30% EtOAc/pentane + 0.5% AcOH) afforded phenyloxetane acid **26** as colourless crystals (64.0 mg, 26%). R_f = 0.25 (30% EtOAc/pentane + 0.5% AcOH); ^{1}H NMR (400 MHz, $CDCl_3$) δ 7.43–7.33 (m, 3 H, 3 × Ph-CH), 7.28–7.26 (m, 2 H, 2 × Ph-CH), 5.28 (d, *J* = 6.2 Hz, 2 H, C*H*HOC*H*H), 5.06 (d, *J* = 6.2 Hz, 2 H, CH*H*OCH*H*). The observed characterisation data (R_f, ^{1}H) were consistent with that previously reported.[15]

3-(4-Bromophenyl)-3-(4-methoxyphenyl)oxetane (27)

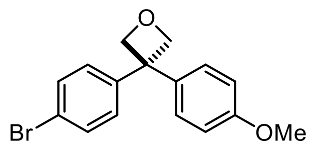

$FeCl_3$ (8.1 mg, 0.05 mmol) was weighed in a vial. Oxetanol **1e** (114 mg, 0.50 mmol) and anisole (92.4 µL, 0.85 mmol) were added. The reaction vessel was sealed and HFIP (5.0 mL) was added. The reaction was stirred for 18 h at 80 °C, then cooled to rt and sat. aq. $NaHCO_3$ (20 mL) was added followed by Et_2O (15 mL). The layers were separated, and the aqueous portion was extracted with Et_2O (2 × 15 mL). The organic extracts were combined, dried over Na_2SO_4, filtered and concentrated under reduced pressure. Purification by flash chromatography (8% Et_2O/pentane) afforded oxetane **27** (43.8 mg, 27%) as a white solid. R_f = 0.19 (8% Et_2O/pentane); mp = 76–77 °C; IR (film)/cm^{-1} 3004, 2952, 2885, 2840, 1610, 1513, 1397, 1244, 1185, 1077, 1025, 997, 935, 812; ^1H NMR (400 MHz, $CDCl_3$) δ 7.52–7.44 (m, 2 H, 2 × Ar-CH), 7.15–7.07 (m, 4 H, 4 × Ar-CH), 6.94–6.86 (m, 2 H, 2 × Ar-CH), 5.22 (d, *J* = 5.7 Hz, 2 H, C*H*HOC*H*H), 5.17 (d, *J* = 5.7 Hz, 2 H, CH*H*OCH*H*), 3.82 (s, 3 H, OCH_3); $^{13}C\{^1H\}$ NMR (101 MHz, $CDCl_3$) δ 158.3 (Ar-C_q-OCH_3), 145.1 (Ar-C_q-C_q), 137.1 (Ar-C_q-C_q), 131.6 (2 × Ar-CH), 128.3 (2 × Ar-CH), 127.5 (2 × Ar-CH), 120.5 (Ar-C_q-Br), 114.0 (2 × Ar-CH), 84.5 (CH_2OCH_2), 55.3 (OCH_3), 50.6 (C_q); HRMS (FTMS +APCI) *m/z* calcd for $C_{16}H_{16}{}^{79}BrO_2^+$ $[M+H]^+$: 319.0328, found: 319.0314.

3,3-Bis(4-methoxyphenyl)oxetane (28), 1,3-bis(4-methoxyphenyl)propan-2-one (28c) and 2,3-bis(4-methoxyphenyl)propanal (28a)

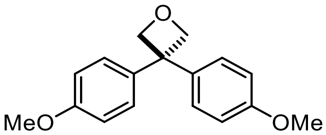

28

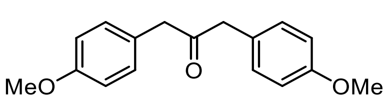

28c

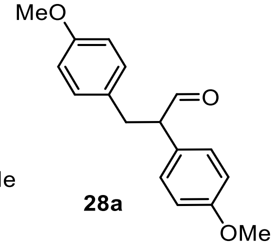

28a

$FeCl_3$ (8.1 mg, 0.05 mmol) was weighed in a vial. *p*-Methoxyphenol-oxetanol **1g**[7] (90.0 mg, 0.50 mmol) and anisole (92.4 µL, 0.85 mmol) were added. The reaction vessel was sealed and HFIP (5.0 mL) was added. The reaction was stirred for 18 h at 80 °C, then cooled to rt and sat. aq. $NaHCO_3$ (20 mL) was added followed by Et_2O (15 mL). The layers were separated, and the aqueous portion was extracted with Et_2O (2 × 15 mL). The organic extracts were combined, dried over Na_2SO_4, filtered and concentrated under reduced pressure. Purification by flash chromatography (10% Et_2O/pentane) afforded aldehyde **28a** (37.1 mg, 27%) as a while solid, followed by a mixture of oxetane **28** and ketone **28c** (42:58, 10.5 mg, 8%) as a yellow oil.

Aldehyde **28a**: R_f = 0.19 (10% Et_2O/pentane); mp = 61–62 °C, IR (film)/cm^{-1} 2996, 2952, 2907, 2832, 1710 (C=O), 1610, 1461, 1300, 1244, 1174, 1107, 1028, 872, 812, 730; ^1H NMR (400 MHz, $CDCl_3$) δ 9.71 (d, *J* = 1.6 Hz, 1 H, HC=O), 7.08–7.01 (m, 2 H, 2 × Ar-CH), 6.99–6.93 (m, 2 H, 2 × Ar-CH), 6.91–6.84 (m, 2 H, 2 × Ar-CH), 6.80–6.72 (m, 2 H, 2 × Ar-CH), 3.81 (s, 3 H, OCH_3), 3.76 (s, 3 H, OCH_3), 3.76–3.71 (m, 1 H, (C=O)-CH), 3.37 (dd, *J* = 14.1, 6.7 Hz, 1 H, CH-C*H*H), 2.89 (dd, *J* = 14.1, 8.0 Hz, 1 H, CH-CH*H*); $^{13}C\{^1H\}$ NMR (101 MHz, $CDCl_3$) δ 200.2 (C=O), 160.0 (Ar-C_q-OCH_3), 159.1 (Ar-C_q-OCH_3), 130.9 (Ar-C_q-CH), 130.1 (2 × Ar-CH), 130.0 (2 × Ar-CH), 127.6 (Ar-C_q-CH_2), 114.4 (2 × Ar-CH), 113.7 (2 × Ar-CH), 60.3 ((C=O)-*C*H), 55.3 (OCH_3), 55.2 (OCH_3), 35.3 (CH-CH_2); HRMS (TOF +ESI) *m/z* calcd for $C_{17}H_{19}O_3$ [M+H]: 271.1334, found: 271.1334.

Oxetane **28** (ox) and ketone **28c** (ket): R_f = 0.09 (10% Et_2O/pentane); ^1H NMR (400 MHz, $CDCl_3$) δ 7.17–7.11 (m, 4 H, 4 × Ar-CH_{ox}), 7.10–7.05 (m, 4 H, 4 × Ar-CH_{ket}), 6.92–6.88 (m, 4 H, 4 × Ar-CH_{ox}), 6.88–6.84 (m, 4 H, 4 × Ar-CH_{ket}), 5.21 (s, 4 H, CH_2OCH_2), 3.82 (s, 6 H, 2 × $OCH_{3,ox}$), 3.81 (s, 6 H, 2 × $OCH_{3,ket}$), 3.66 (s, 4 H, 2 × Ar-CH_2); $^{13}C\{^1H\}$ NMR (101 MHz, $CDCl_3$) δ 206.5 (C=O), 158.6 (2 × Ar-C_q-$OCH_{3,ket}$), 158.2 (2 × Ar-C_q-$OCH_{3,ox}$), 138.2 (2 × Ar-C_q-$C_{q,ox}$), 130.5 (4 × Ar-CH_{ket}), 127.6 (4 × Ar-CH_{ox}), 126.1 (2 × Ar-C_q-$CH_{2,ket}$), 114.1 (4 × Ar-CH_{ket}), 113.9 (4 × Ar-CH_{ox}), 84.9 (CH_2OCH_2), 55.3 (2 × $OCH_{3,ox}$), 55.2 (2 × $OCH_{3,ket}$), 50.3 ($C_{q,ox}$), 48.0 (2 × $CH_{2,ket}$). The observed spectroscopic data for ketone **28c** (^1H and ^{13}C NMR) was consistent with that previously reported.[17]

3-(3-Methoxyphenyl)-3-(4-methoxyphenyl)oxetane (29)

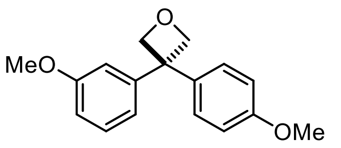

$FeCl_3$ (8.1 mg, 0.05 mmol) was weighed in a vial. *meta*-Methoxyphenyl-oxetanol **1h** (90.0 mg, 0.50 mmol) and anisole (92.4 µL, 0.85 mmol) were added. The reaction vessel was sealed and HFIP (5.0 mL) was added. The reaction was stirred for 18 h at 80 °C, then cooled to rt and sat. aq. $NaHCO_3$ (20 mL) was added followed by Et_2O (15 mL). The layers were separated, and the aqueous portion was extracted with Et_2O (2 × 15 mL). The organic extracts were combined, dried over Na_2SO_4, filtered and concentrated under reduced pressure. Purification by flash chromatography (10% Et_2O/pentane) afforded oxetane **29** (31.5 mg, 23%) as a yellow oil. R_f = 0.19 (10% Et_2O/pentane); IR (film)/cm^{-1} 2952, 2873, 2832, 1602, 1513, 1289, 1244, 1177, 1028, 984, 943, 875, 827, 779, 700; ^{1}H NMR (400 MHz, $CDCl_3$) δ 7.32–7.27 (m, 1 H, Ar-CH), 7.19–7.11 (m, 2 H, 2 × Ar-CH), 6.93–6.85 (m, 2 H, 2 × Ar-CH), 6.84–6.78 (m, 2 H, 2 × Ar-CH), 6.74 (dd, *J* = 2.5, 1.8 Hz, 1 H, Ar-CH), 5.25 (d, *J* = 5.7 Hz, 2 H, C*H*HOC*H*H), 5.20 (d, *J* = 5.7 Hz, 2 H, CH*H*OCH*H*), 3.81 (s, 3 H, OCH_3), 3.80 (s, 3 H, OCH_3); ^{13}C{^{1}H} NMR (101 MHz, $CDCl_3$) δ 159.7 (Ar-C_q-OCH_3), 158.2 (Ar-C_q-OCH_3), 147.6 (Ar-C_q-C_q), 137.8 (Ar-C_q-C_q), 129.6 (Ar-CH), 127.6 (2 × Ar-CH), 118.9 (Ar-CH), 113.9 (2 × Ar-CH), 112.8 (Ar-CH), 111.5 (Ar-CH), 84.6 (CH_2OCH_2), 55.3 (OCH_3), 55.2 (OCH_3), 50.9 (C_q); HRMS (+EI) *m/z* calcd for $C_{17}H_{18}O_3^{\bullet+}$ $[M]^{\bullet+}$: 270.1250, Found: 270.1258.

2-(2-Methoxyphenyl)-3-(4-methoxyphenyl)propanal (30a)

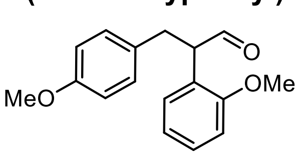

$FeCl_3$ (8.1 mg, 0.05 mmol) was weighed in a vial. *o*-Methoxyphenol-oxetanol **1o** (90.0 mg, 0.50 mmol) and anisole (92.4 µL, 0.85 mmol) were added. The reaction vessel was sealed and HFIP (5.0 mL) was added. The reaction was stirred for 18 h at 80 °C. The reaction was then cooled to rt and sat. aq. $NaHCO_3$ (20 mL) was added followed by Et_2O (20 mL). The layers were separated, and the aqueous portion was extracted with Et_2O (2 × 20 mL). The organic extracts were combined, dried over Na_2SO_4, filtered and concentrated under reduced pressure. Purification by flash chromatography (8% Et_2O/pentane) afforded aldehyde **30a** (57.6 mg, 43%) as a yellow oil. R_f = 0.24 (8% Et_2O/hexane); IR (film)/cm^{-1} 2937, 2832, 1718 (C=O), 1610, 1513, 1461, 1283, 1241, 1177, 1103, 1028, 823, 752; ^{1}H NMR (400 MHz, $CDCl_3$) δ 9.74 (d, *J* = 0.7 Hz, 1 H, HC=O), 7.32–7.23 (m, 1 H, Ar-CH), 7.00–6.93 (m, 3 H, 3 × Ar-CH), 6.93–6.88 (m, 2 H, 2 × Ar-CH), 6.77–6.72 (m, 2 H, 2 × Ar-CH), 4.01 (dd, *J* = 7.9, 6.4 Hz, 1 H, (C=O)-CH), 3.78 (s, 3 H, OCH_3), 3.76 (s, 3 H, OCH_3), 3.42 (dd, *J* = 14.0, 6.4 Hz, 1 H, CH-C*H*H), 2.90 (dd, *J* = 14.0, 7.9 Hz, 1 H, CH-CH*H*); ^{13}C{^{1}H} NMR (101 MHz, $CDCl_3$) δ 201.0 (C=O), 157.9 (Ar-C_q-$OCH_{3,para}$), 157.3 (Ar-C_q-$OCH_{3,ortho}$), 131.6 (Ar-C_q), 130.3 (Ar-CH), 130.0 (2 × Ar-CH), 128.8 (Ar-CH), 125.1 (Ar-C_q), 120.8 (Ar-CH), 113.5 (2 × Ar-CH), 110.9 (Ar-CH), 55.6 (OCH_3), 55.4 (OCH_3), 55.2 (*C*H-CH_2), 33.8 (CH-*C*H_2); HRMS (TOF +ESI) *m/z* calcd for $C_{17}H_{19}O_3$ [M+H]: 271.1334, found: 271.1343.

Unsuccessful Substrates

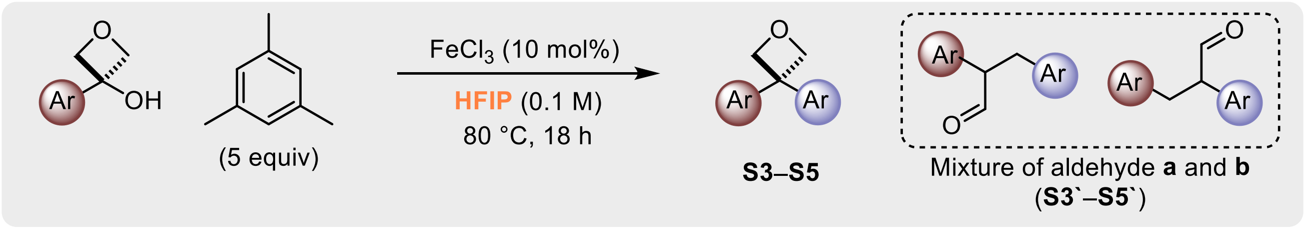

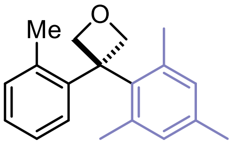

S3 traces
S3` 27%

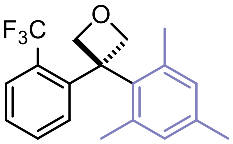

S4 traces
S4` traces

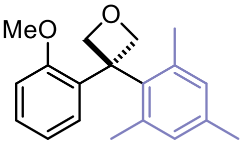

S5 traces
S5` traces

Synthesis of Tesmilifene Analogue (32)

3-(4-(2-Bromoethoxy)phenyl)-3-phenyloxetane (31), 3-(2-(2-bromoethoxy)phenyl)-3-phenyloxetane (*o*-31), 3-(4-(2-bromoethoxy)phenyl)-2-phenylpropanal (31a) and **2-(4-(2-bromoethoxy)phenyl)-3-phenylpropanal (31b)**

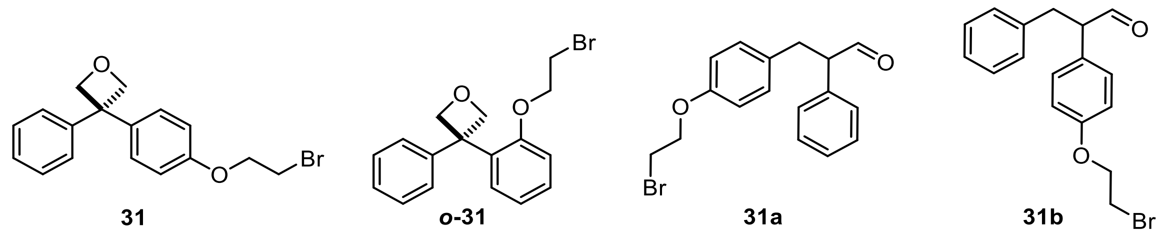

31 ***o*-31** **31a** **31b**

$FeCl_3$ (8.1 mg, 0.05 mmol) was weighed in a vial. Oxetanol **1a** (75.1 mg, 0.50 mmol) and β–bromophenetole (171 mg, 0.85 mmol) were added. The reaction vessel was sealed and HFIP (5.0 mL) was added. The reaction was stirred for 18 h at 80 °C, then cooled to rt and sat. aq. $NaHCO_3$ (20 mL) was added followed by Et_2O (15 mL). The layers were separated, and the aqueous portion was extracted with Et_2O (2 × 15 mL). The organic extracts were combined, dried over Na_2SO_4, filtered and concentrated under reduced pressure. Purification by flash chromatography (10% Et_2O/pentane) afforded a mixture of aldehyde **31a** and **31b** (54:46, 15.1 mg, 9%) as a colourless oil, followed by oxetane **31** (65.4 mg, 39%) as an off-white solid, and oxetane ***o*-31** (25.2 mg, 80% pure, 15%) as a yellow oil.

Aldehyde **31a** and **31b**: R_f = 0.55 (10% Et_2O/pentane); IR (film)/cm^{-1} 3060, 3026, 2926, 2821, 2721, 1722 (C=O), 1602, 1490, 1453, 1282, 1244, 1177, 1017, 752, 700; ^{1}H NMR (400 MHz, $CDCl_3$) δ 9.79 (d, J = 0.6 Hz, 1 H, H_aC=O), 9.75 (d, J = 1.6 Hz, 1 H, H_bC=O), 7.40–7.22 (m, 4 H, 4 × Ar-CH), 7.23–7.11 (m, 5 H, 5 × Ar-CH), 7.10–7.03 (m, 2 H, 2 × Ar-CH), 7.03–6.91 (m, 4 H, 4 × Ar-CH), 6.85 (dd, J = 8.2, 1.1 Hz, 1 H, Ar-CH), 6.80–6.74 (m, 2 H, 2 × Ar-CH_b), 4.32–4.24 (m, 1 H, OC*H*H_a), 4.24 (t, J = 6.3 Hz, 2 H, $OCH_{2,b}$), 4.19–4.10 (m, 2 H, OCH*H*$_a$, (C=O)-CH_a), 3.80 (dd, J = 7.9, 6.8, 1.6 Hz, 1 H, (C=O)-CH_b), 3.61 (t, J = 6.3 Hz, 2 H, $CH_{2,b}$Br), 3.56 (t, J = 6.0 Hz, 2 H, $CH_{2,a}$Br), 3.51 (dd, J = 13.9, 6.2 Hz, 1 H, CH-C*H*H_a), 3.42 (dd, J = 14.1, 6.8 Hz, 1 H, CH-C*H*H_b), 3.00 (dd, J = 13.9, 8.3 Hz, 1 H, CH-CH*H*$_a$), 2.94 (dd, J = 14.1, 7.9 Hz, 1 H, CH-CH*H*$_b$); ^{13}C{^{1}H} NMR (101 MHz, $CDCl_3$) δ 200.6 (C_a=O), 200.0 (C_b=O), 156.6 (Ar-$C_{q,b}$-O), 155.8 (Ar-$C_{q,a}$-O), 139.4 (Ar-$C_{q,a}$-CH_2), 135.9 (Ar-$C_{q,b}$-CH_2), 131.6 (Ar-$C_{q,b}$-CH), 130.6 (Ar-$C_{q,a}$-CH), 130.1 (2 × Ar-CH_b), 129.2 (2 × Ar-CH_a), 129.0 (2 × Ar-CH), 128.9 (2 × Ar-CH), 128.1 (2 × Ar-CH_a), 127.7 (Ar-CH), 126.1 (2 × Ar-CH_a), 121.6 (2 × Ar-CH_b), 114.6 (2 × Ar-CH_b), 111.7 (Ar-CH), 67.9 ($OCH_{2,a}$ and $OCH_{2,b}$), 61.1 ((C=O)-CH_b), 55.2 ((C=O)-CH_a), 35.3 (CH-$CH_{2,b}$), 35.0 (CH-$CH_{2,a}$), 29.1 ($CH_{2,a}$Br), 29.0 ($CH_{2,b}$Br); HRMS (FTMS +APCI) *m/z* calcd for $C_{17}H_{16}{}^{79}BrO_2{}^+$ [M–H]$^+$: 331.0328, found: 331.0327.

Oxetane **31**: R_f = 0.38 (10% Et_2O/pentane); mp = 85–86 °C (Et_2O/pentane ppt); IR (film)/cm^{-1} 2952, 2877, 1610, 1513, 1446, 1282, 1244, 1185, 1013, 967, 831, 760, 700; ^{1}H NMR (400 MHz, $CDCl_3$) δ 7.42–7.32 (m, 2 H, 2 × Ar-CH), 7.32–7.24 (m, 1 H, Ar-CH), 7.25–7.19 (m, 2 H, 2 × Ar-CH), 7.19–7.11 (m, 2 H, 2 × Ar-CH), 6.94–6.87 (m, 2 H, 2 × Ar-CH), 5.27 (d, J = 5.7 Hz, 2 H, C*H*HOC*H*H), 5.21 (d, J = 5.7 Hz, 2 H, CH*H*OCH*H*), 4.30 (t, J = 6.3 Hz, 2 H, OCH_2), 3.65 (t, J = 6.3 Hz, 2 H, CH_2Br); ^{13}C{^{1}H} NMR (101 MHz, $CDCl_3$) δ 156.7 (Ar-C_q-O), 145.8 (Ar-C_q-C_q), 138.8 (Ar-C_q-C_q), 128.6 (2 × Ar-CH), 127.7 (2 × Ar-CH), 126.6 (Ar-CH), 126.4 (2 × Ar-CH), 114.8 (2 × Ar-CH), 84.7 (CH_2OCH_2), 67.9 (OCH_2), 50.9 (C_q), 29.1 (CH_2Br); HRMS (FTMS +APCI) *m/z* calcd for $C_{17}H_{16}{}^{79}BrO_2{}^+$ [M–H]$^+$: 331.0328, found: 331.0339.

Oxetane ***o*-31**: R_f = 0.18 (10% Et_2O/pentane); IR (film)/cm^{-1} 3428, 3656, 2952, 2877, 1595, 1479, 1215, 1185, 1013, 969, 827, 749, 697; ^{1}H NMR (400 MHz, $CDCl_3$) δ 7.35–7.33 (m, 3 H, 3 × Ar-CH), 7.26–7.21 (m, 2 H, 2 × Ar-CH), 7.01–6.94 (m, 2 H, 2 × Ar-CH), 6.91 (d, J = 8.0 Hz, 2 H, 2 × Ar-CH), 4.81 (d, J = 8.9 Hz, 2 H, C*H*HOC*H*H), 4.63 (d, J = 8.9 Hz, 2 H, CH*H*OCH*H*), 4.15 (dd, J = 11.2, 5.1 Hz, 2 H, OCH_2), 4.08 (dd, J = 11.2, 8.0 Hz, 2 H, CH_2Br); ^{13}C{^{1}H} NMR (101 MHz, $CDCl_3$) δ 160.8 (Ar-C_q-O), 142.7 (Ar-C_q-C_q), 129.8 (Ar-C_q-C_q), 129.3 (Ar-CH), 128.8 (2 × Ar-CH), 127.1 (Ar-CH), 126.9 (2 × Ar-CH), 125.2 (Ar-CH), 120.8 (Ar-CH), 110.2 (Ar-CH), 81.2 (CH_2OCH_2), 67.6 (OCH_2), 56.4 (C_q), 30.3 (CH_2Br); HRMS (FTMS +APCI) *m/z* calcd for $C_{17}H_{16}{}^{79}BrO_2{}^+$ [M–H]$^+$: 331.0328, found: 331.0326.

N,N-Diethyl-2-(4-(3-phenyloxetan-3-yl)phenoxy)ethan-1-amine (32)

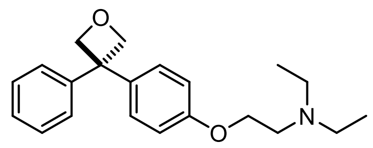

Diethylamine (40.3 μL, 0.39 mmol) was added to a solution of oxetane **31** (65 mg, 0.20 mmol) and K_2CO_3 (56.6 mg, 0.41 mmol) in DMF (1.5 mL). The reaction mixture was stirred at 80 °C for 23 h, then cooled to rt and water (10 mL) was added followed by Et_2O (10 mL). The layers were separated, and the aqueous portion was extracted with Et_2O (2 × 10 mL). The organic extracts were combined, washed with sat. aq. NaCl (2 × 5 mL), dried over Na_2SO_4, filtered and concentrated under reduced pressure. Purification by flash chromatography (10% MeOH/CH_2Cl_2) afforded oxetane **32** (44.4 mg, 70%) as a light-yellow paste. R_f = 0.34 (10% MeOH/CH_2Cl_2); IR (film)/cm^{-1} 2967, 2873, 1610, 1513, 1464, 1297, 1244, 1181, 1028, 987, 831, 760, 700; ^{1}H NMR (400 MHz, $CDCl_3$) δ 7.40–7.32 (m, 2 H, 2 × Ar-CH), 7.31–7.24 (m, 1 H, Ar-CH), 7.24–7.18 (m, 2 H, 2 × Ar-CH), 7.17–7.10 (m, 2 H, 2 × Ar-CH), 6.93–6.85 (m, 2 H, 2 × Ar-CH), 5.25 (d, *J* = 5.6 Hz, 2 H, C*H*HOC*H*H), 5.21 (d, *J* = 5.6 Hz, 2 H, CH*H*OCH*H*), 4.13 (t, *J* = 6.3 Hz, 2 H, OCH_2), 2.98 (t, *J* = 6.3 Hz, 2 H, OCH_2CH_2N), 2.75 (q, *J* = 7.2 Hz, 4 H, 2 × NCH_2), 1.14 (t, *J* = 7.2 Hz, 6 H, 2 × NCH_2CH_3); $^{13}C\{^1H\}$ NMR (101 MHz, $CDCl_3$) δ 157.2 (Ar-C_q-O), 145.9 (Ar-C_q-C_q), 138.1 (Ar-C_q-C_q), 128.5 (2 × Ar-CH), 127.6 (2 × Ar-CH), 126.5 (Ar-CH), 126.4 (2 × Ar-CH), 114.4 (2 × Ar-CH), 84.7 (CH_2OCH_2), 65.8 (OCH_2), 51.5 (OCH_2CH_2N), 50.8 (C_q), 47.6 (2 × NCH_2CH_3), 11.2 (2 × NCH_2CH_3); HRMS (TOF ES+) *m/z* calcd for $C_{21}H_{28}NO_2^+$ $[M+H]^+$: 326.2120, found: 326.2119.

Synthesis of Fenofibrate Analogue (35)

(4-(3-(4-Chlorophenyl)oxetan-3-yl)phenoxy)triisopropylsilane (33)

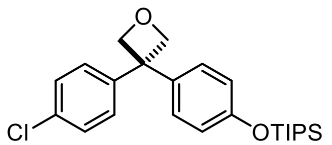

$FeCl_3$ (8.1 mg, 0.05 mmol) was weighed in a vial. Oxetanol **1d** (92.3 mg, 0.50 mmol) and triisopropyl(phenoxy)silane[18] (213 mg, 0.85 mmol) were added. The reaction vessel was sealed and HFIP (5.0 mL) was added. The reaction was stirred for 18 h at 80 °C, then cooled to rt and sat. aq. $NaHCO_3$ (20 mL) was added followed by Et_2O (15 mL). The layers were separated, and the aqueous portion was extracted with Et_2O (2 × 15 mL). The organic extracts were combined, dried over Na_2SO_4, filtered and concentrated under reduced pressure. Purification by flash chromatography (4% Et_2O/pentane) afforded oxetane **33** (40.1 mg, 19%) as a yellow oil. R_f = 0.34 (4% Et_2O/pentane); IR (film)/cm^{-1} 2944, 2870, 1729, 1600, 1513, 1464, 1267, 1177, 1155, 1095, 1013, 916, 834, 685; ^{1}H NMR (400 MHz, $CDCl_3$) δ 7.35–7.30 (m, 2 H, 2 × Ar-CH), 7.19–7.13 (m, 2 H, 2 × Ar-CH), 7.04–6.98 (m, 2 H, 2 × Ar-CH), 6.90–6.84 (m, 2 H, 2 × Ar-CH), 5.22 (d, *J* = 5.7 Hz, 2 H, C*H*HOC*H*H), 5.15 (d, *J* = 5.7 Hz, 2 H, CH*H*OCH*H*), 1.32–1.20 (m, 3 H, 3 × CH), 1.11 (d, *J* = 7.3 Hz, 18 H, 6 × CH_3); $^{13}C\{^1H\}$ NMR (101 MHz, $CDCl_3$) δ 154.9 (Ar-C_q-OTIPS), 144.7 (Ar-C_q-C_q), 137.4 (Ar-C_q-C_q), 132.4 (Ar-C_q-Cl), 128.6 (2 × Ar-CH), 128.0 (2 × Ar-CH), 127.5 (2 × Ar-CH), 119.9 (2 × Ar-CH), 84.7 (CH_2OCH_2), 50.6 (C_q), 17.9 (3 × $CH(CH_3)_2$), 12.6 (3 × $CH(CH_3)_2$); HRMS (FTMS +APCI / TOF ES+) *Mass not found*.

4-(3-(4-Chlorophenyl)oxetan-3-yl)phenol (34)

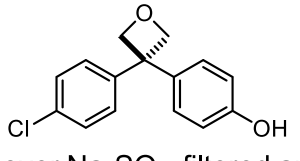

A solution of TBAF (1 M in THF, 145 μL, 0.145 mmol) was added dropwise to a solution of **33** (40.0 mg, 0.096 mmol) in THF (1.0 mL) at 25 °C. The reaction mixture was stirred for 1 h, and then water (10 mL) was added followed by Et_2O (10 mL). The layers were separated, and the aqueous portion was extracted with Et_2O (2 × 10 mL). The organic extracts were combined, washed with sat. aq. NH_4Cl (2 × 5 mL), dried over Na_2SO_4, filtered and concentrated under reduced pressure. Purification by flash column chromatography (30% Et_2O/pentane) afforded oxetane **34** (21.9 mg, 88%) as a white solid. R_f = 0.25 (30% Et_2O/pentane); mp = 158–160 °C (Et_2O/pentane ppt); IR (film)/cm^{-1} 3302 (br, OH), 2959, 2885, 1610, 1513, 1438, 1263, 1230, 1177, 1095, 976, 931, 827, 730; ^{1}H NMR (400 MHz, $CDCl_3$) δ 7.37–7.30 (m, 2 H, 2 × Ar-CH), 7.20–7.13 (m, 2 H, 2 × Ar-CH), 7.09–7.02 (m, 2 H, 2 × Ar-CH), 6.87–6.78 (m, 2 H, 2 × Ar-CH), 5.27 (br, s, 1 H, OH), 5.23 (d, *J* = 5.7 Hz, 2 H, C*H*HOC*H*H), 5.19 (d, *J* = 5.7 Hz, 2 H, CH*H*OCH*H*); $^{13}C\{^1H\}$ NMR (101 MHz, $CDCl_3$) δ 154.5 (Ar-C_q-OH), 144.4 (Ar-C_q-C_q), 137.2 (Ar-C_q-C_q), 132.5 (Ar-C_q-Cl), 128.7 (2 × Ar-CH), 127.9 (2 × Ar-CH), 127.7 (2 × Ar-CH), 115.5 (2 × Ar-CH), 84.6 (CH_2OCH_2), 50.5 (C_q); HRMS (TOF –ESI) *m/z* calcd for $C_{15}H_{12}{}^{35}ClO_2$ $[M-H]^-$: 259.0526, found: 259.0531.

Isopropyl 2-(4-(3-(4-chlorophenyl)oxetan-3-yl)phenoxy)-2-methylpropanoate (35)

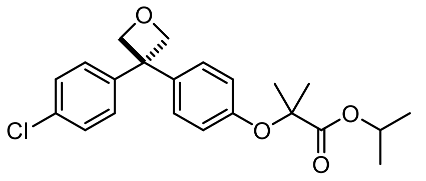

Isopropyl 2-bromo-2methylpropanoate (20.6 µL, 0.122 mmol) was added to a solution of oxetane **34** (10.6 mg, 0.041 mmol) and K_2CO_3 (18.0 mg, 0.13 mmol) in DMF (1.0 mL). The reaction mixture was stirred at 90 °C for 23 h, then cooled to rt and water (10 mL) was added followed by Et_2O (10 mL). The layers were separated, and the aqueous portion was extracted with Et_2O (2 × 10 mL). The organic extracts were combined, dried over Na_2SO_4, filtered and concentrated under reduced pressure. Purification by flash chromatography (30% Et_2O/pentane) afforded oxetane **35** (11.1 mg, 70%) as a colourless oil. R_f = 0.53 (30% Et_2O/pentane); IR (film)/cm^{-1} 2981, 2877, 1729 (C=O), 1509, 1382, 1285, 1241, 1177, 1148, 1103, 1013, 935, 827; ^{1}H NMR (400 MHz, $CDCl_3$) δ 7.36–7.28 (m, 2 H, 2 × Ar-CH), 7.20–7.10 (m, 2 H, 2 × Ar-CH), 7.08–7.01 (m, 2 H, 2 × Ar-CH), 6.88–6.79 (m, 2 H, 2 × Ar-CH), 5.20 (d, *J* = 5.7 Hz, 2 H, C*H*HOC*H*H), 5.16 (d, *J* = 5.7 Hz, 2 H, CH*H*OCH*H*), 5.09 (h, *J* = 6.3 Hz, 1 H, C*H*(CH_3)$_2$), 1.60 (s, 6 H, 2 × CH_3), 1.23 (d, *J* = 6.3 Hz, 6 H, CH(C*H*$_3$)$_2$); ^{13}C{^{1}H} NMR (101 MHz, $CDCl_3$) δ 173.6 (C=O), 154.3 (Ar-C_q-O), 144.4 (Ar-*C*$_q$-C_q), 138.4 (Ar-*C*$_q$-C_q), 132.5 (Ar-C_q-Cl), 128.6 (2 × Ar-CH), 127.9 (2 × Ar-CH), 127.2 (2 × Ar-CH), 118.9 (2 × Ar-CH), 84.6 (*C*H_2O*C*H_2), 79.1 (*C*$_q$(CH_3)$_2$), 69.0 (*C*H(CH_3)$_2$), 50.5 (C_q), 25.4 (C_q(*C*H_3)$_2$), 21.5 (CH(*C*H_3)$_2$); HRMS (FTMS +ESI) *m/z* calcd for $C_{22}H_{26}{}^{35}ClO_4^+$ $[M+H]^+$: 389.1514, found: 389.1508.

Further derivatisation of iodoarene

4-(4-(3-Mesityloxetan-3-yl)phenyl)-1-methyl-1H-pyrazole (36)

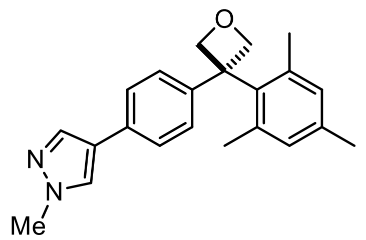

1-Methylpyrazole-4-boronic acid pinacol ester (50 mg, 0.24 mmol), iodoarene **14** (76 mg, 0.20 mmol), Pd(dppf)Cl_2 (14 mg, 0.02 mmol) and K_2CO_3 (84 mg, 0.60 mmol) were dissolved in dioxane/H_2O (2:1, 1.2 mL) and the resulting mixture was stirred at 100°C under Ar for 16 h. The reaction mixture was quenched with H_2O (2 mL) then extracted with EtOAc (2 × 5 mL). The organic extracts were combined, dried over Na_2SO_4, filtered and concentrated under reduced pressure. Purification by flash chromatography (50% Et_2O/pentane) afforded the pyrazole **36** (66 mg, 0.199 mmol, 99%) as a white foam. R_f = 0.18 (50% Et_2O/pentane); IR (film)/cm^{-1} 2950, 2872, 1571, 1446, 1374, 986, 956, 833, 734, 580; ^{1}H NMR (400 MHz, $CDCl_3$) δ 7.76 (s, 1H, Ar-CH), 7.61 (s, 1H, Ar-CH), 7.48 (d, *J* = 8.8 Hz, 2H, 2 × Ar-CH), 7.43 (d, *J* = 8.7 Hz, 2H, 2 × Ar-CH), 6.90 (s, 2H, 2 × Ar-CH), 5.41 (d, *J* = 6.0 Hz, 2H, CHHOCHH), 4.83 (d, *J* = 6.0 Hz, 2H, CHHOCHH), 3.96 (s, 3H, CH_3), 2.33 (s, 3H, CH_3), 1.88 (s, 6H, 2 × CH_3); ^{13}C{^{1}H} NMR (101 MHz, $CDCl_3$) δ 143.0 (Ar-C_q), 137.7 (Ar-C_q), 136.7 (Ar-CH), 136.2 (Ar-CH), 131.4 (Ar-C_q), 129.8 (2 × Ar-CH), 126.8 (Ar-C_q), 126.3 (2 × Ar-CH), 125.7 (2 × Ar-CH), 122.9 (Ar-C_q), 85.2 (CH_2OCH_2), 52.5 (C_q), 39.1 (Ar-CH_3), 20.7 (Ar-CH_3), 20.2 (2 × Ar-CH_3); HRMS (TOF ESI+) m/z calcd. for $C_{22}H_{25}N_2O$ $[M+H]^+$: 333.1967; found 333.1970.

Benzyl 4-(hydroxy(4-(3-mesityloxetan-3-yl)phenyl)methyl)piperidine-1-carboxylate (37)

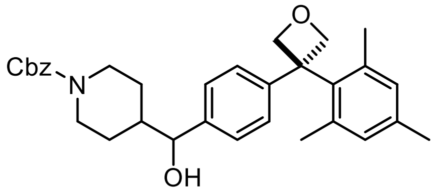

Iodoarene **14** (76 mg, 0.20 mmol) was dissolved in THF (0.5 mL) and the resulting solution was cooled to -78°C. *n*-BuLi (0.15 mL of 2 M solution in hexane, 0.30 mmol) was added dropwise and the mixture was stirred for 30 min. A solution of benzyl 4-formylpiperidine-1-carboxylate (69 mg, 0.30 mmol) in THF (0.5 mL) was added dropwise and the resulting mixture was stirred for 1 h at -78°C. The reaction mixture was warmed to 0°C and quenched with saturated aqueous NH_4Cl (1 mL). Phases were separated, and the aqueous layer was extracted with Et_2O (2 × 2 mL). The organic extracts were combined, dried over Na_2SO_4, filtered and concentrated under reduced pressure. Purification by flash chromatography (50% Et_2O/pentane) afforded the alcohol **37** (41 mg, 0.082 mmol, 41%) as a colourless oil. R_f = 0.23 (50% Et_2O/pentane); IR (film)/cm^{-1} 3443, 2952, 2870, 1696, 1446, 1431, 1278, 1202, 1017, 732; ^{1}H NMR (400 MHz, $CDCl_3$) δ 7.47 (d, *J* = 8.4 Hz, 2H, 2 × Ar-CH), 7.41–7.30 (m, 5H, 5 × Ar-CH), 7.27 (d, *J* = 8.5 Hz, 2H, 2 × Ar-CH), 6.91 (s, 2H, 2 × Ar-CH), 5.41 (d, *J* = 5.5 Hz, 2H, C*H*HOC*H*H), 5.13 (s, 2H, PhCH_2), 4.81 (d, *J* = 5.6 Hz, 2H, CH*H*OCH*H*), 4.40 (d, *J* = 7.4 Hz, 1H, CHOH), 4.34–4.07 (m, 2H, NCH_2), 2.85–2.60 (m, 2H, NCH_2), 2.34 (s, 3H, CH_3), 2.02 (d, *J* = 12.9 Hz, 1H, C*H*(CH_2)$_2$), 1.87 (s, 6H, 2 × CH_3), 1.85–1.76 (m, 1H, CH(C*H*$_2$)$_2$), 1.39–1.27 (m, 2H, CH(C*H*$_2$)$_2$), 1.25–1.10 (m, 1H, CH(C*H*$_2$)$_2$); ^{13}C{^{1}H} NMR (101 MHz, $CDCl_3$) δ 155.2 (C=O), 144.6

(Ar-C$_q$), 141.5 (Ar-C$_q$), 137.6 (Ar-C$_q$), 136.9 (Ar-C$_q$), 136.3 (Ar-C$_q$), 136.1 (Ar-C$_q$), 129.8 (2 × Ar-CH), 128.4 (2 × Ar-CH), 127.9 (Ar-CH), 127.8 (2 × Ar-CH), 126.8 (2 × Ar-CH), 125.8 (2 × Ar-CH), 85.0 (CH_2OCH_2), 78.2 (CHOH), 67.0 ($PhCH_2$), 52.5 (C$_q$), 44.0 (NCH_2), 43.9 (NCH_2), 43.2 (*C*H(CH_2)$_2$), 28.2 (CH(*C*H_2)$_2$), 20.7 (Ar-CH_3), 20.2 (2 × Ar-CH_3); HRMS (TOF ESI+) m/z calcd. for $C_{32}H_{38}NO_4$ [M+H]$^+$: 500.2801; found 500.2818.

3-Mesityl-3-(4-(*p*-tolylthio)phenyl)oxetane (38)

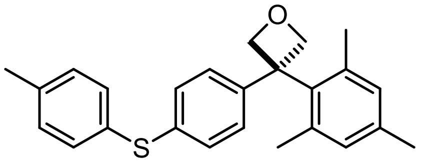

Iodoarene **14** (76 mg, 0.20 mmol) was dissolved in THF (0.5 mL) and the resulting solution was cooled to -78°C. *n*-BuLi (0.15 mL of 2 M solution in hexane, 0.30 mmol) was added dropwise and the mixture was stirred for 30 min. A solution of *p*-tolyl disulfide (74 mg, 0.30 mmol) in THF (0.5 mL) was added dropwise and the resulting mixture was stirred for 1 h at -78°C. The reaction mixture was warmed to 0°C and quenched with saturated aqueous NH_4Cl (1 mL). Phases were separated, and the aqueous layer was extracted with Et_2O (2 × 2 mL). The organic extracts were combined, dried over Na_2SO_4, filtered and concentrated under reduced pressure. Purification by flash chromatography (50% Et_2O/pentane) afforded the thioether **38** (73 mg, 0.19 mmol, 97%) as a colourless oil. R_f = 0.27 (5% Et_2O/pentane); IR (film)/cm^{-1} 2952, 2920, 2870, 1491, 1452, 1014, 990, 811, 580; ^{1}H NMR (400 MHz, $CDCl_3$) δ 7.42 – 7.31 (m, 4H, 4 × Ar-CH), 7.20 (d, *J* = 8.5 Hz, 2H, 2 × Ar-CH), 7.16 (d, *J* = 7.6 Hz, 2H, 2 × Ar-CH), 6.88 (s, 2H, 2 × Ar-CH), 5.39 (d, *J* = 6.0 Hz, 2H, CHHOCHH), 4.78 (d, *J* = 6.1 Hz, 2H, CHHOCHH), 2.36 (s, 3H, CH_3), 2.31 (s, 3H, CH_3), 1.86 (s, 6H, 2 × CH_3); ^{13}C{^{1}H} NMR (101 MHz, $CDCl_3$) δ 143.1 (Ar-C$_q$), 137.8 (Ar-C$_q$), 137. 5 (Ar-C$_q$), 136.3 (Ar-C$_q$), 136.1 (Ar-C$_q$), 135.9 (Ar-C$_q$), 132.7 (2 × Ar-CH), 130.1 (2 × Ar-CH), 129.8 (2 × Ar-CH), 129.4 (2 × Ar-CH), 126.5 (2 × Ar-CH), 85.0 (CH_2OCH_2), 52.4 (C$_q$), 21.1 (Ar-CH_3), 20. 7 (Ar-CH_3), 20.2 (2 × Ar-CH_3); HRMS (FTMS ESI+) m/z calcd. for $C_{25}H_{27}OS$ [M+H]: 375.1777; found 375.1770.

Alternative Michael Acceptor Reactivity

3-Mesityl-2-methylpropanal (41)

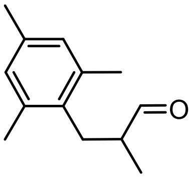

$FeCl_3$ (4.9 mg, 0.03 mmol) was weighed in a vial. Methacrolein (25 µL, 0.30 mmol) and mesitylene (209 µL, 1.50 mmol) were added. The reaction vessel was sealed and HFIP (5.0 mL) was added. The reaction was stirred for 23 h at 80 °C, then cooled to rt and sat. aq. $NaHCO_3$ (15 mL) was added followed by Et_2O (15 mL). The layers were separated, and the aqueous portion was extracted with Et_2O (2 × 15 mL). The organic extracts were combined, dried over Na_2SO_4, filtered and concentrated under reduced pressure. Purification by flash chromatography (4% Et_2O/pentane) afforded aldehyde **41** (43.1 mg, 75%) as a yellow oil. R_f = 0.43 (4% Et_2O/pentane); IR (film)/cm^{-1} 2963, 2922, 2873, 1722 (C=O), 1613, 1461, 1379, 920, 849; ^{1}H NMR (400 MHz, $CDCl_3$) δ 9.73 (d, *J* = 1.3 Hz, 1 H, HC=O), 6.88 (s, 2 H, 2 × Ar-CH), 3.11–2.96 (m, 1 H, CH-CH*H*), 2.75–2.59 (m, 2 H, CH-C*H*H, (C=O)-CH), 2.31 (s, 6 H, 2 × CH_3), 2.28 (s, 3 H, CH_3), 1.14 (d, *J* = 6.7 Hz, 3 H, CH_3); ^{13}C{^{1}H} NMR (101 MHz, $CDCl_3$) δ 204.6 (C=O), 136.3 (2 × Ar-*C$_q$*-CH_3), 135.6 (Ar-*C$_q$*-CH_3), 132.6 (Ar-*C$_q$*-CH_2), 129.2 (2 × Ar-CH), 46.6 ((C=O)-*C*H), 29.8 (CH-*C*H_2), 20.7 (Ar-CH_3), 20.2 (2 × Ar-CH_3), 13.4 (CH_3); HRMS (+EI) *m/z* calcd for $C_{13}H_{18}O^•$ [M]$^•$: 190.1358, found: 190.1363.

2-Methyl-3-(2,3,5,6-tetramethylphenyl)propanal (42)

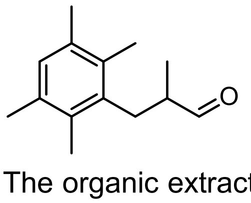

$FeCl_3$ (8 mg, 0.05 mmol) was weighed in a vial. Methacrolein (41 µL, 0.50 mmol) and 1,2,4,5-tetramethylbenzene (336 mg, 2.50 mmol) were added. The reaction vessel was sealed and HFIP (5.0 mL) was added. The reaction was stirred for 23 h at 80 °C, then cooled to rt and sat. aq. $NaHCO_3$ (15 mL) was added followed by Et_2O (15 mL). The layers were separated, and the aqueous portion was extracted with Et_2O (2 × 15 mL). The organic extracts were combined, dried over Na_2SO_4, filtered and concentrated under reduced pressure. Purification by flash chromatography (5% Et_2O/pentane) afforded aldehyde **42** (23 mg, 0.11 mmol, 23%) as a yellow oil. R_f = 0.28 (5% Et_2O/pentane); IR (film)/cm^{-1} 2969, 2932, 1722, 1456, 1470, 1385; ^{1}H NMR (400 MHz, $CDCl_3$) δ 9.71 (d, *J* = 1.6 Hz, 1H, HC=O), 6.90 (s, 1H, Ar-CH), 3.12 (dd, *J* = 14.3, 6.6 Hz, 1H, Ar-C*H*H), 2.80 (dd, *J* = 14.3, 8.6 Hz, 1H, Ar-CH*H*), 2.67 – 2.57 (m, 1H, CHMe), 2.24 (s, 6H, 2 × Ar-CH), 2.20 (s, 6H, 2 × Ar-CH), 1.12 (d, *J* = 7.0 Hz, 3H, Ar-CH); ^{13}C{^{1}H} NMR (101 MHz, $CDCl_3$) δ 204.8 (C=O), 135.4 (Ar-C$_q$), 134.0 (2

× Ar-C_q), 132.4 (2 × Ar-C_q), 130.0 (Ar-CH), 47.1 (*C*HCHO), 30.6 ($ArCH_2$), 20.7 (2 × Ar-CH_3), 16.1 (2 × Ar-CH_3), 13.3 (Ar-CH_3); HRMS (FTMS + ESI^+) m/z calcd. for $C_{14}H_{21}O$ $[M + H]^+$: 205.1587; found 205.1584.

3-Mesitylpropanenitrile (43)

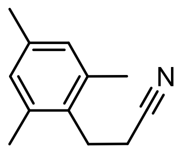

$FeCl_3$ (21.1 mg, 0.13 mmol) was weighed in a vial. Acrylonitrile (33 μL, 0.5 mmol) and mesitylene (350 μL, 2.5 mmol) were added. The reaction vessel was sealed and HFIP (5.0 mL) was added. The reaction was stirred for 18 h at 80 °C, then cooled to rt and sat. aq. $NaHCO_3$ (15 mL) was added followed by Et_2O (15 mL). The layers were separated, and the aqueous portion was extracted with Et_2O (2 × 15 mL). The organic extracts were combined, dried over Na_2SO_4, filtered and concentrated under reduced pressure. Purification by flash chromatography (5% Et_2O/pentane) afforded **43** (36.2 mg, 42%) as a white solid. R_f = 0.30 (5% Et_2O/pentane); mp = 83 °C; ^1H NMR (400 MHz, $CDCl_3$) δ 6.88 (s, 2 H, 2 × Ar-CH), 3.02 (dd, *J* = 8.7, 7.3 Hz, 2 H, Ar-CH_2), 2.51 – 2.42 (m, 2 H, CH_2CN), 2.33 (s, 6 H, 2 × Ar-CH_3), 2.25 (s, 3 H, Ar-CH_3); $^{13}C\{^1H\}$ NMR (101 MHz, $CDCl_3$) δ 136.6 (Ar-C_q), 136.0 (Ar-C_q), 131.8 (Ar-C_q), 129.3 (2 × Ar-CH), 119.4 (Ar-C_q), 25.2 ($ArCH_2$), 20.8 (Ar-CH_3), 19.7 (2 × Ar-CH_3), 16.7 (CH_2CN). The observed spectroscopic data for this compound (^1H, ^{13}C, R_f and mp) was consistent with that previously reported.[19]

3-(4-Methoxyphenyl)propanenitrile (44)

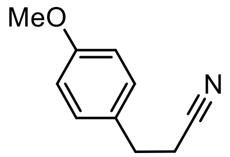

$FeCl_3$ (21 mg, 0.13 mmol) was weighed in a vial. Acrylonitrile (33 μL, 0.5 mmol) and anisole (92 μL, 0.85 mmol) were added. The reaction vessel was sealed and HFIP (5.0 mL) was added. The reaction was stirred for 18 h at 80 °C, then cooled to rt and sat. aq. $NaHCO_3$ (15 mL) was added followed by Et_2O (15 mL). The layers were separated, and the aqueous portion was extracted with Et_2O (2 × 15 mL). The organic extracts were combined, dried over Na_2SO_4, filtered and concentrated under reduced pressure. Purification by flash chromatography (5% Et_2O/pentane) afforded aldehyde **44** (47.3 mg, 59%) as a yellow oil. R_f = 0.20 (4% Et_2O/pentane); ^1H NMR (400 MHz, $CDCl_3$) δ 7.16 (d, *J* = 8.6 Hz, 2H, 2 × Ar-CH), 6.88 (d, *J* = 8.6 Hz, 2H, 2 × Ar-CH), 3.81 (s, 3H, OCH_3), 2.91 (t, *J* = 7.3 Hz, 2H, $ArCH_2$), 2.59 (t, *J* = 7.4 Hz, 2H, $CNCH_2$); $^{13}C\{^1H\}$ NMR (101 MHz, $CDCl_3$) δ 158.7 (CN), 130.1 (Ar-C_q), 129.3 (2 × Ar-CH), 119.2 (Ar-C_q), 114.2 (2 × Ar-CH), 55.2 (OCH_3), 30.7 ($ArCH_2$), 19.6 ($CNCH_2$). The observed spectroscopic data for this compound (^1H and R_f) was consistent with that previously reported.[20]

X-Ray Crystal Structures

Crystals suitable for X-ray analysis were grown by slow evaporation from $CDCl_3$ at 25 °C.

X-Ray structure and analysis of diaryloxetanes **3, 5, 16**

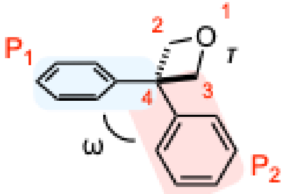

τ = torsion angle (1-2-3-4)
ω = angle between arene planes (P_1-P_2)

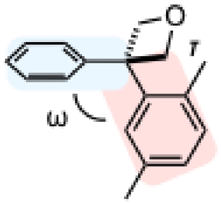

3
τ_A = 17.0(4)°, ω_A = 87.8°
τ_B = 16.0(4)°, ω_B = 89.9°
τ_C = 14.3(4)°, ω_C = 89.3°
τ_D = 15.9(4)°, ω_D = 89.0°

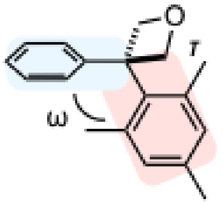

5
τ = 21.5(2)°
ω = 83.2°

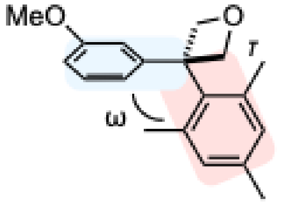

16
τ = puckering angle = 18.6(4)°
ω = angle between arene planes = 88.4°

	oxetane puckering angle (°)	angle between arene planes (°)
**3**	15.8	89.0
**5**	21.5	83.2
**16**	18.6	88.4

The X-ray crystal structure of 3

Crystal data for **3**: $C_{17}H_{18}O$, M = 238.31, triclinic, P–1 (no. 2), a = 10.4461(6), b = 16.1469(10), c = 16.5492(10) Å, α = 95.868(5), β = 92.580(5), γ = 104.933(5)°, V = 2675.6(3) Å^3, Z = 8 (4 independent molecules), D_c = 1.183 g cm^{-3}, μ(Mo-Kα) = 0.072 mm^{-1}, T = 173 K, colourless blocks, Agilent Xcalibur 3 E diffractometer; 23260 independent measured reflections (R_{int} = 0.0595), F^2 refinement,[21-23] R_1(obs) = 0.0608, wR_2(all) = 0.2119, 10061 independent observed absorption-corrected reflections [$|F_o|$ > 4σ($|F_o|$), completeness to $θ_{full}$(25.2°) = 97.8%], 659 parameters. CCDC 2542557.

The crystal of **3** that was studied was found to be a three component twin in a *ca*. 48:46:6 ratio, with the two major lattices related by the approximate twin law [–1.00 0.00 0.00 0.00 –1.00 0.00 0.24 0.25 1.00]. The crystal was found to contain four independent molecules (**3-A**, **3-B**, **3-C** and **3-D**) in the asymmetric unit.

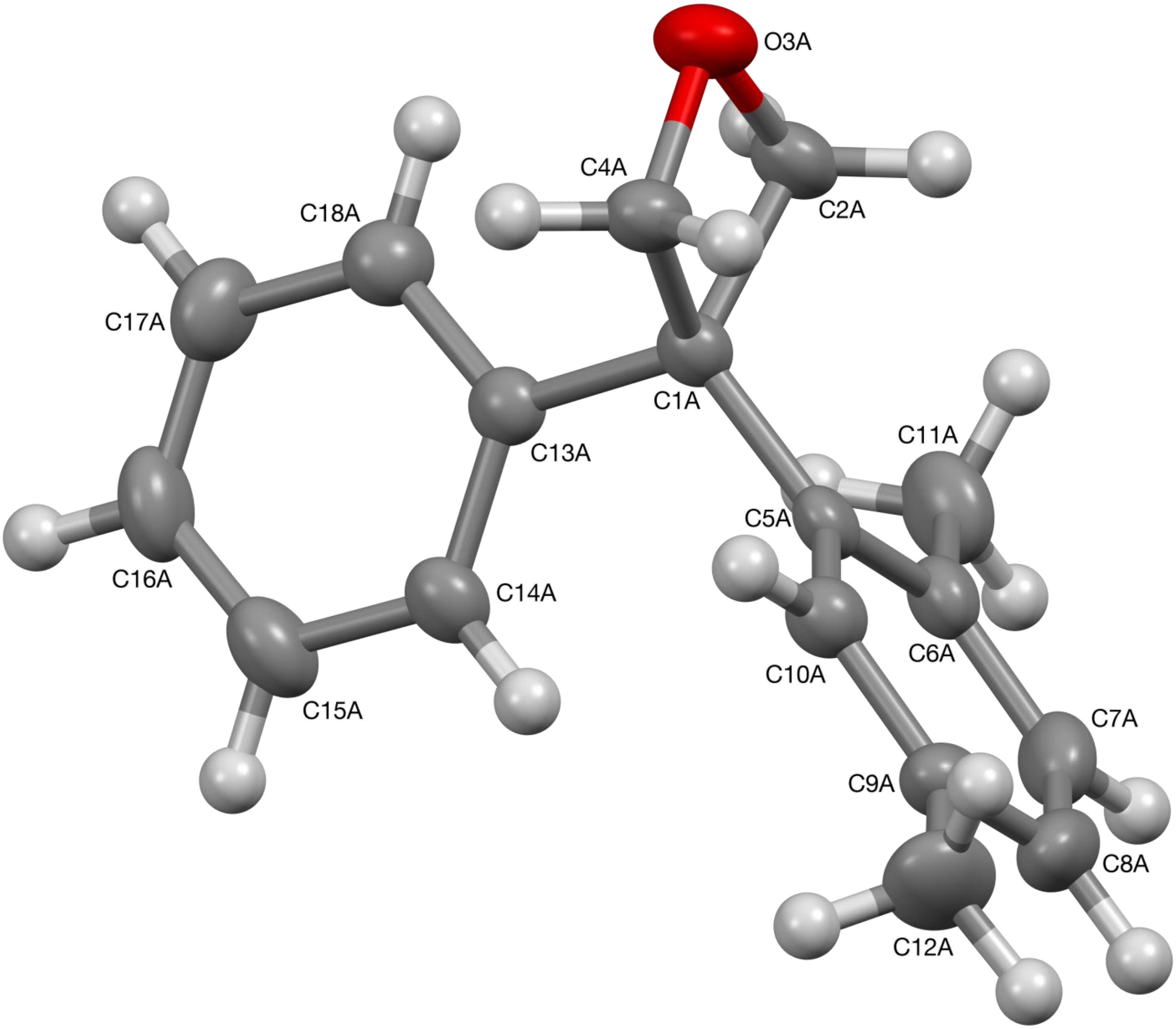

Figure S7. The structure of **3-A**, one of the four independent molecules present in the crystal of **3** (50% probability ellipsoids).

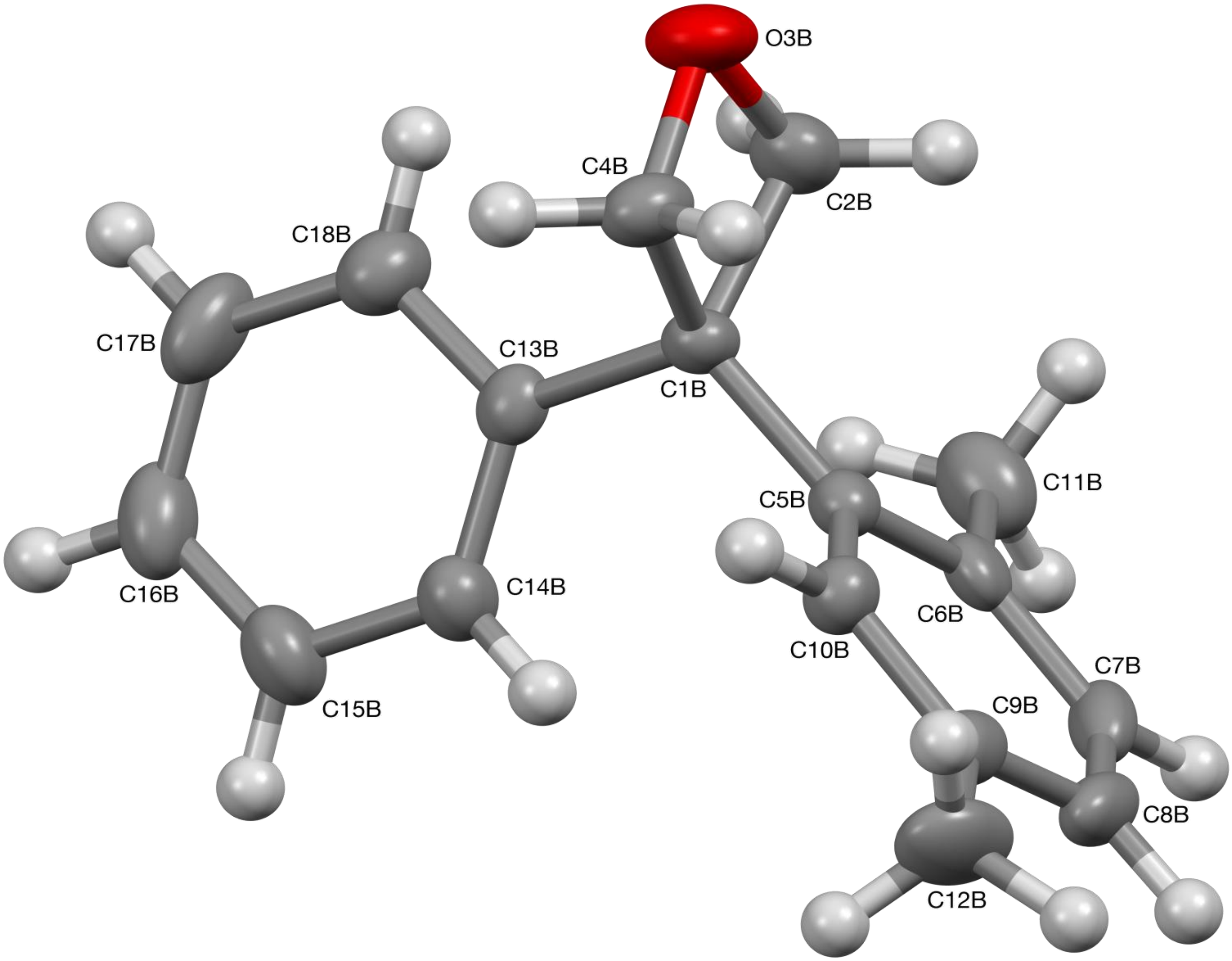

Figure S8. The structure of **3-B**, one of the four independent molecules present in the crystal of **3** (50% probability ellipsoids).

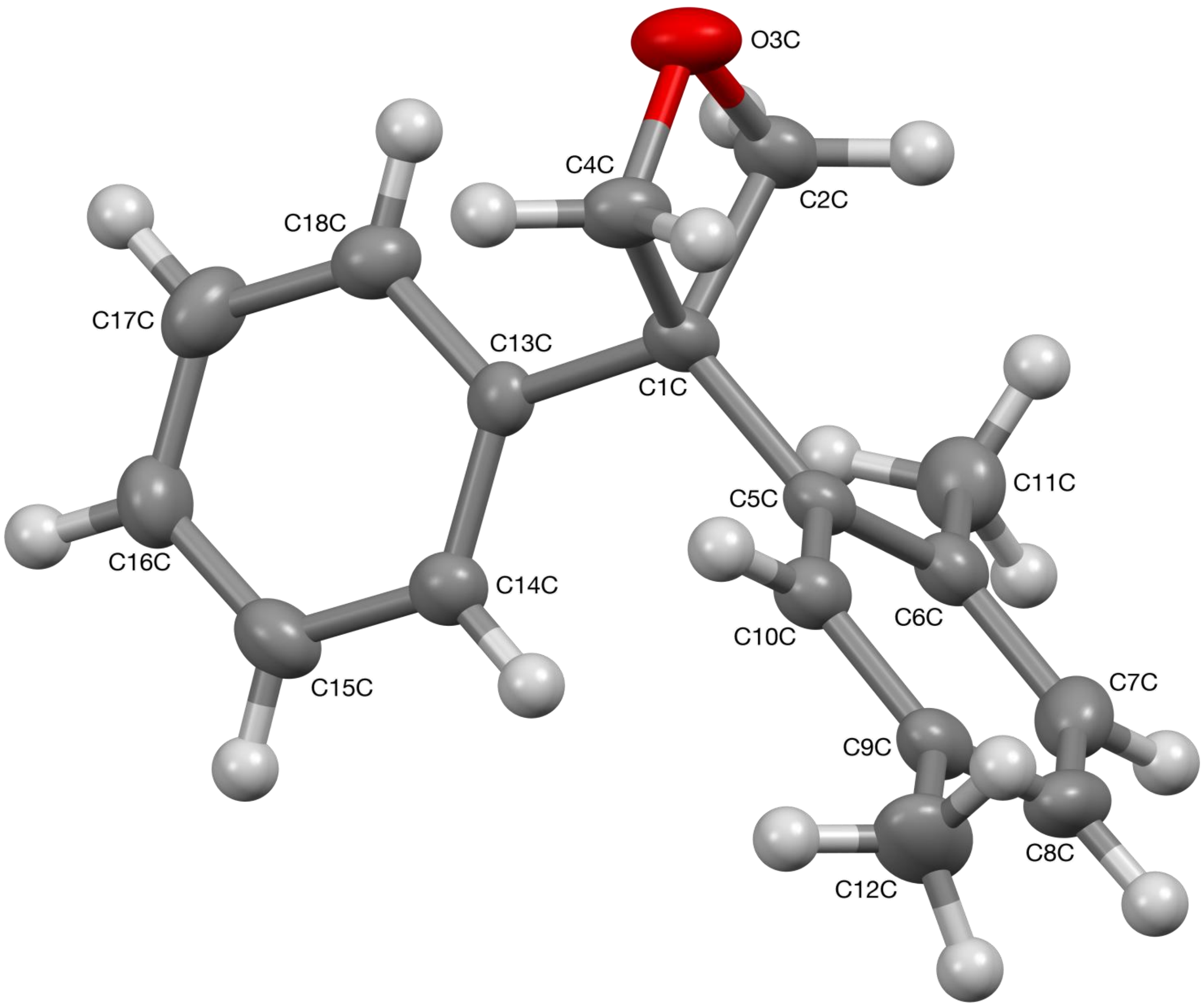

Figure S9. The structure of **3-C**, one of the four independent molecules present in the crystal of **3** (50% probability ellipsoids).

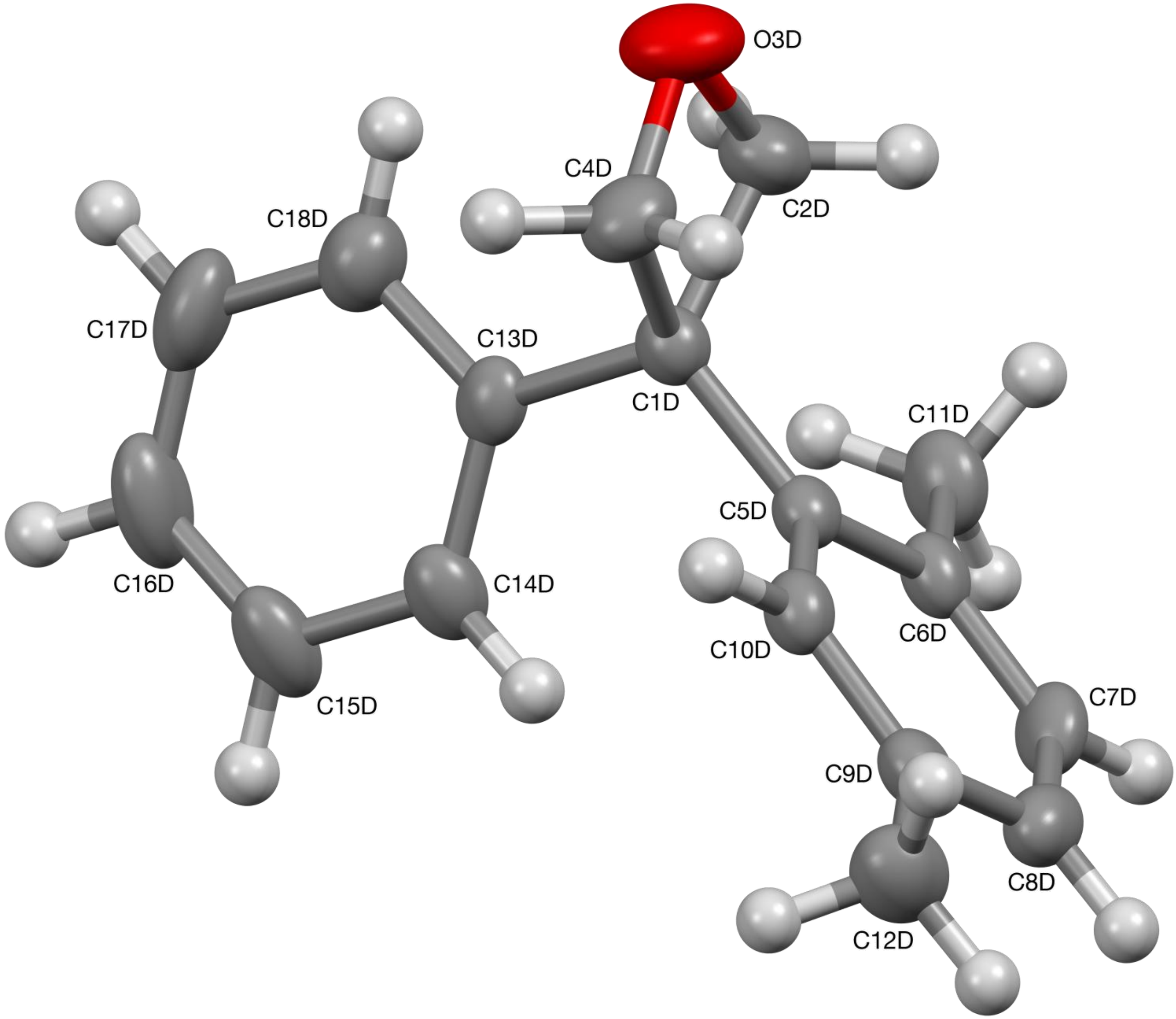

Figure S10. The structure of **3-D**, one of the four independent molecules present in the crystal of **3** (50% probability ellipsoids).

The X-ray crystal structure of 5

Crystal data for **5**: $C_{18}H_{20}O$, M = 252.34, monoclinic, $P2_1/c$ (no. 14), a = 12.0481(5), b = 14.6911(6), c = 8.2772(4) Å, β = 107.553(4)°, V = 1396.85(10) Å^3, Z = 4, D_c = 1.200 g cm^{-3}, μ(Cu-Kα) = 0.554 mm^{-1}, T = 173 K, colourless tabular needles, Agilent Xcalibur PX Ultra A diffractometer; 2660 independent measured reflections (R_{int} = 0.0429), F^2 refinement,[21-23] R_1(obs) = 0.0504, wR_2(all) = 0.1461, 1792 independent observed absorption-corrected reflections [$|F_o| > 4\sigma(|F_o|)$, completeness to θ_{full}(67.7°) = 98.1%], 176 parameters. CCDC 2542558.

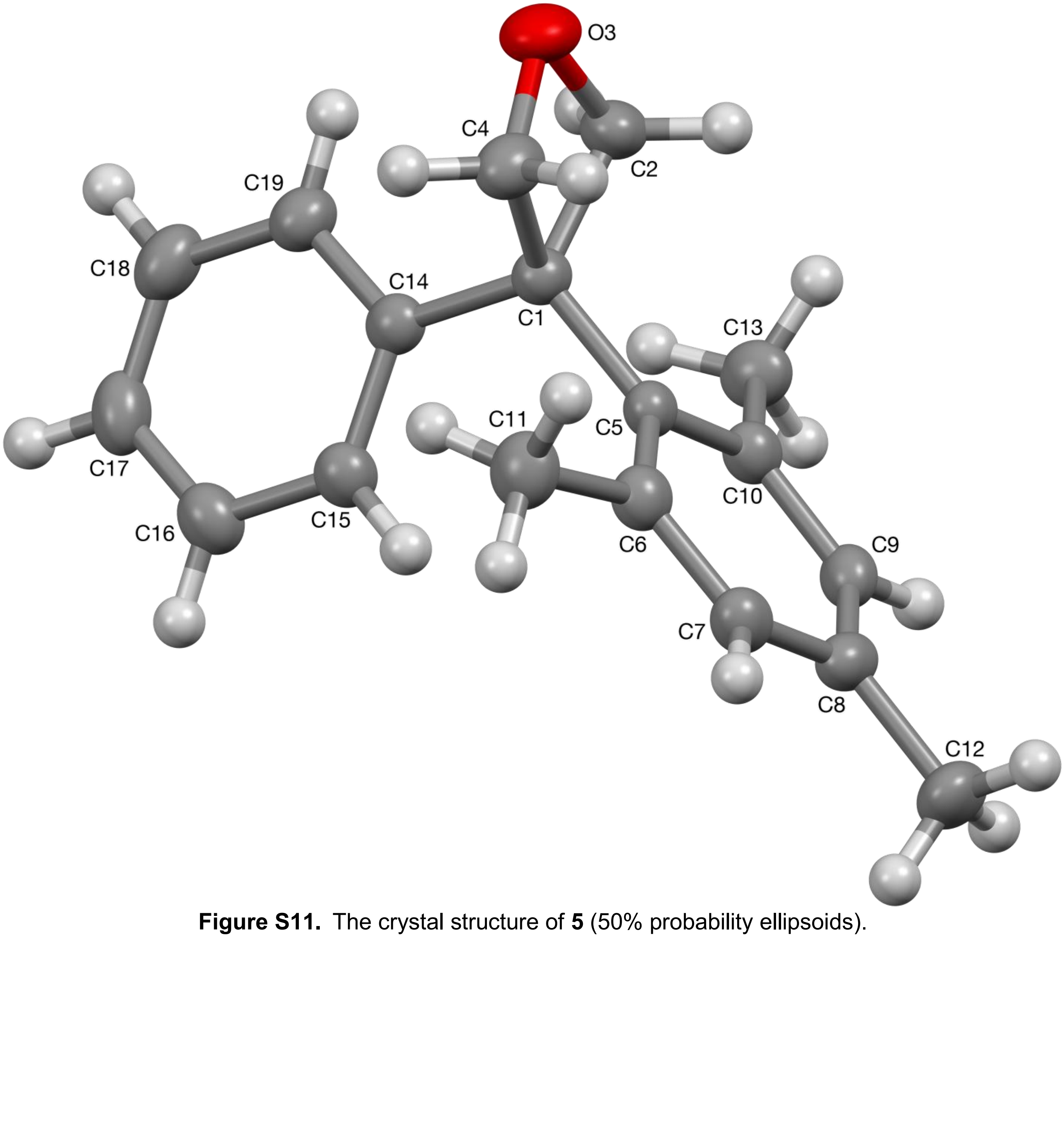

Figure S11. The crystal structure of **5** (50% probability ellipsoids).

The X-ray crystal structure of 16

Crystal data for **16**: $C_{19}H_{22}O_2$, M = 282.36, monoclinic, $P2_1/c$ (no. 14), a = 13.3448(7), b = 14.0437(9), c = 8.2165(5) Å, β = 94.130(6)°, V = 1535.84(16) Å^3, Z = 4, D_c = 1.221 g cm^{-3}, μ(Cu-Kα) = 0.607 mm^{-1}, T = 173 K, colourless blocks, Agilent Xcalibur PX Ultra A diffractometer; 2907 independent measured reflections (R_{int} = 0.0639), F^2 refinement,[21-23] R_1(obs) = 0.0767, wR_2(all) = 0.2659, 1754 independent observed absorption-corrected reflections [$|F_o| > 4\sigma(|F_o|)$, completeness to θ_{full}(67.7°) = 97.8%], 194 parameters. CCDC 2542559.

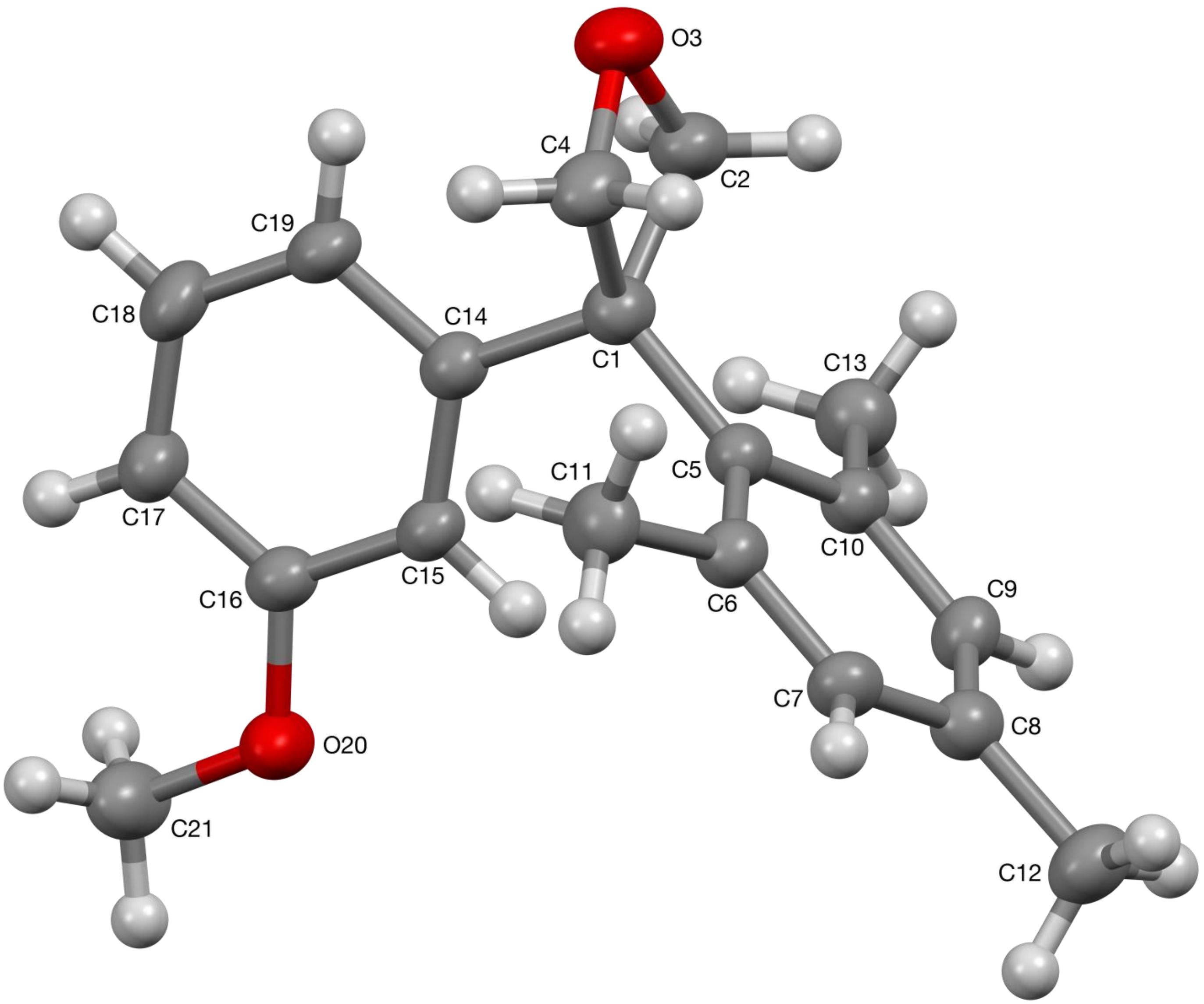

Figure S12. The crystal structure of **16** (50% probability ellipsoids).

^{1}H and $^{13}C\{^{1}H\}$ NMR Spectra of Selected Compounds

2,6-Dimethyl-4-(3-phenyloxetan-3-yl)phenol (2)

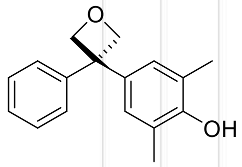

2
^{1}H NMR
(400 MHz, $CDCl_3$)

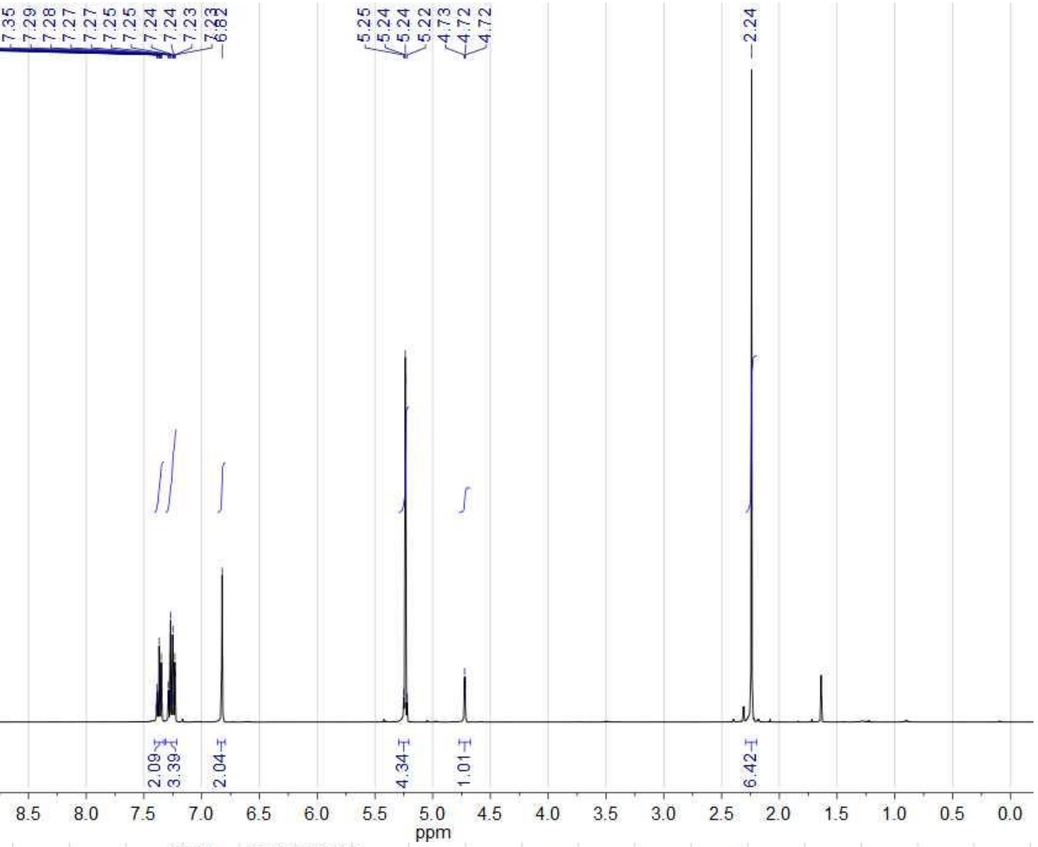

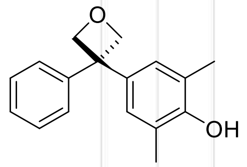

2
$^{13}C\{^1H\}$ NMR
(101 MHz, $CDCl_3$)

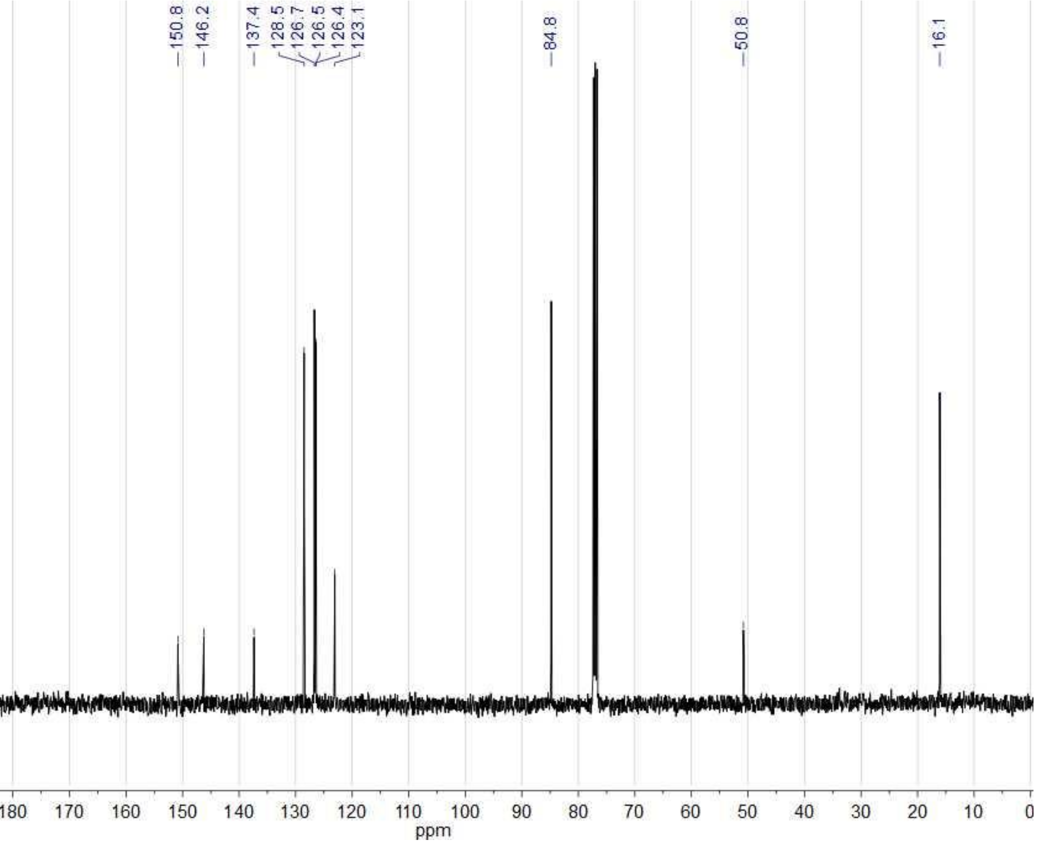

3-(2,5-Dimethylphenyl)-3-phenyloxetane (3)

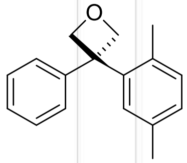

3
^{1}H NMR
(400 MHz, $CDCl_3$)

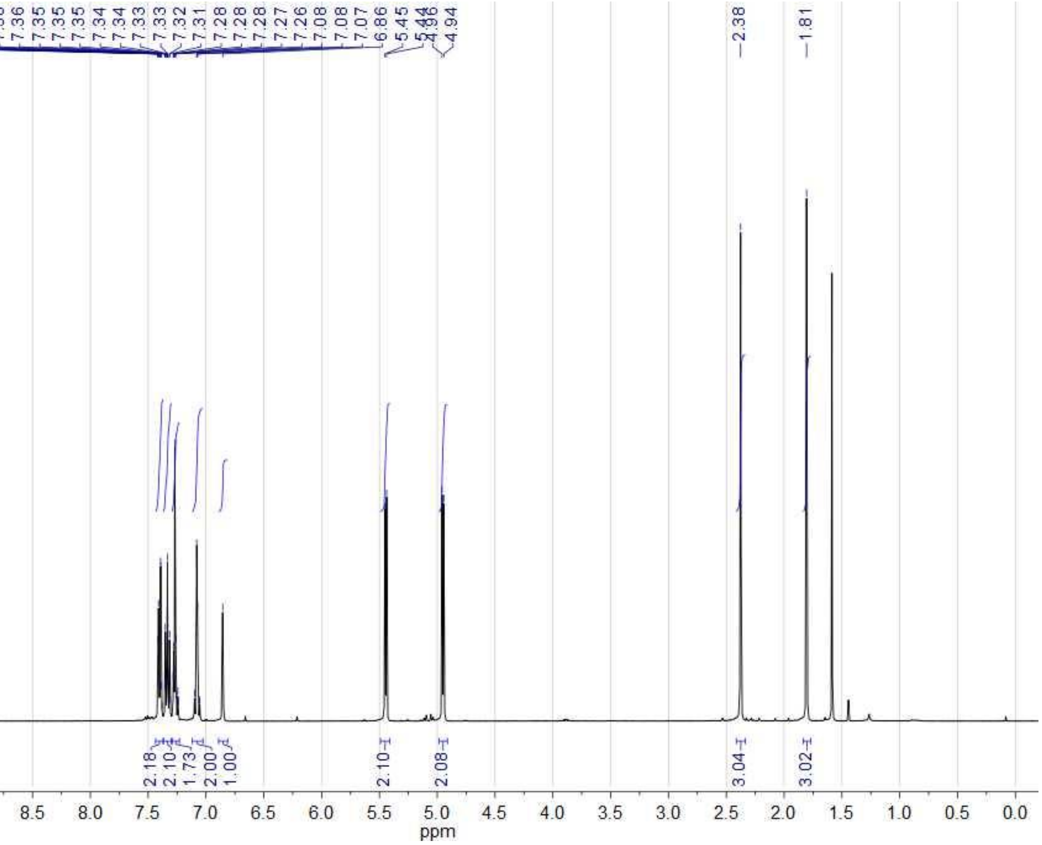

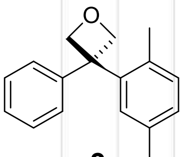

3
$^{13}C\{^1H\}$ NMR
(101 MHz, $CDCl_3$)

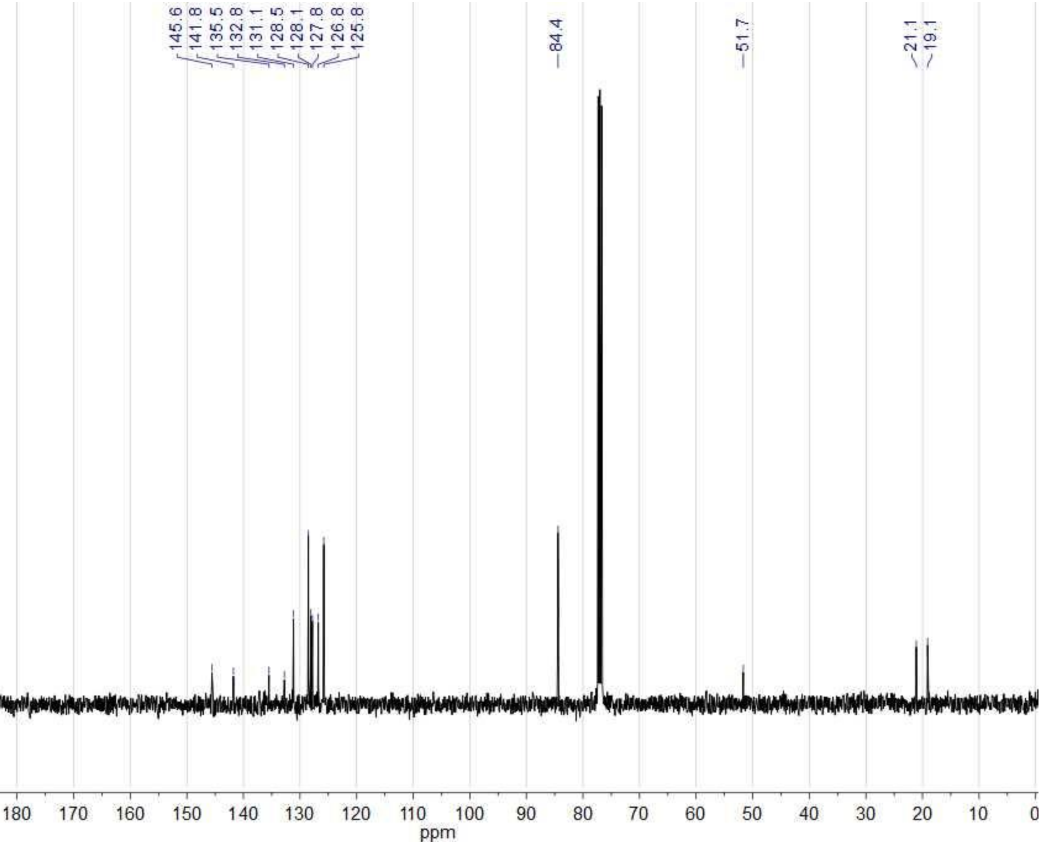

3-(2,5-Dimethylphenyl)-2-phenylpropanal (3b)

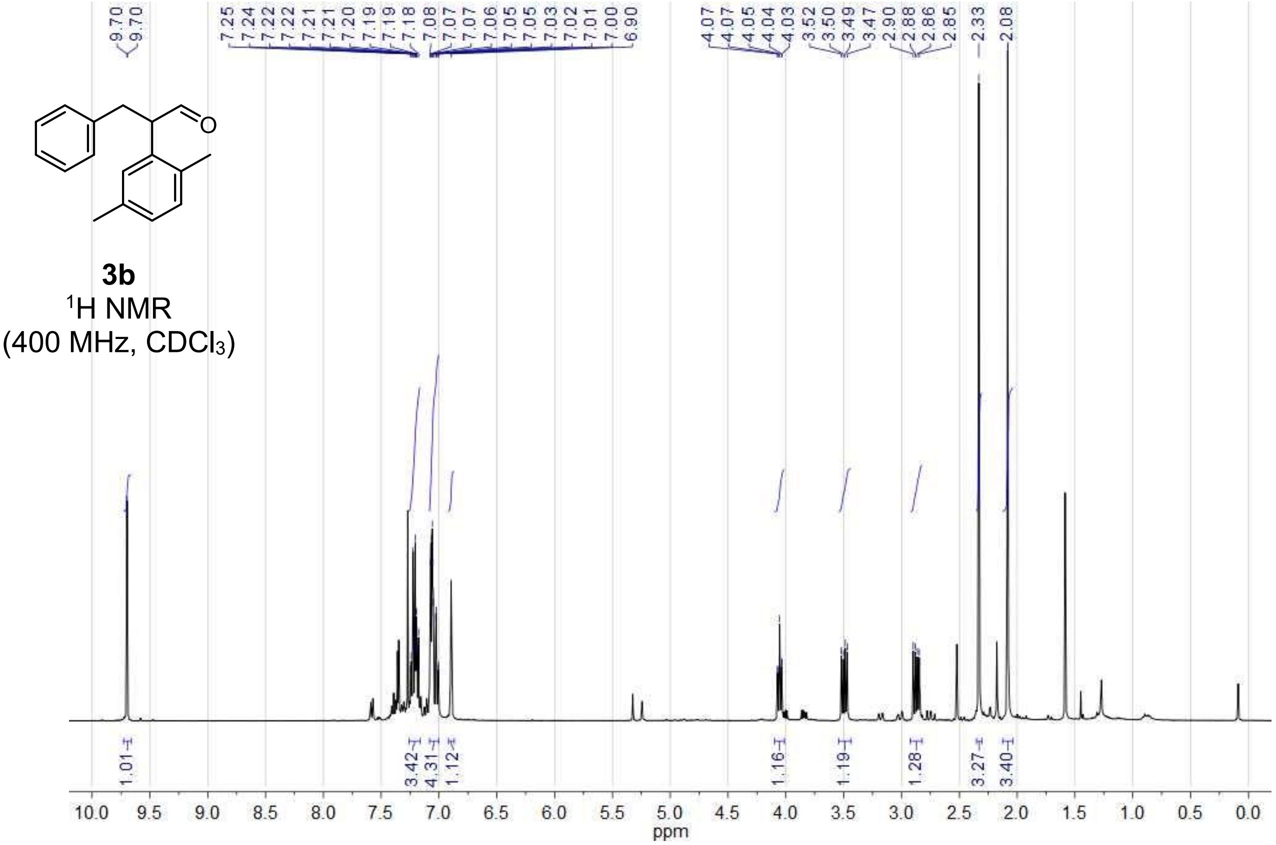

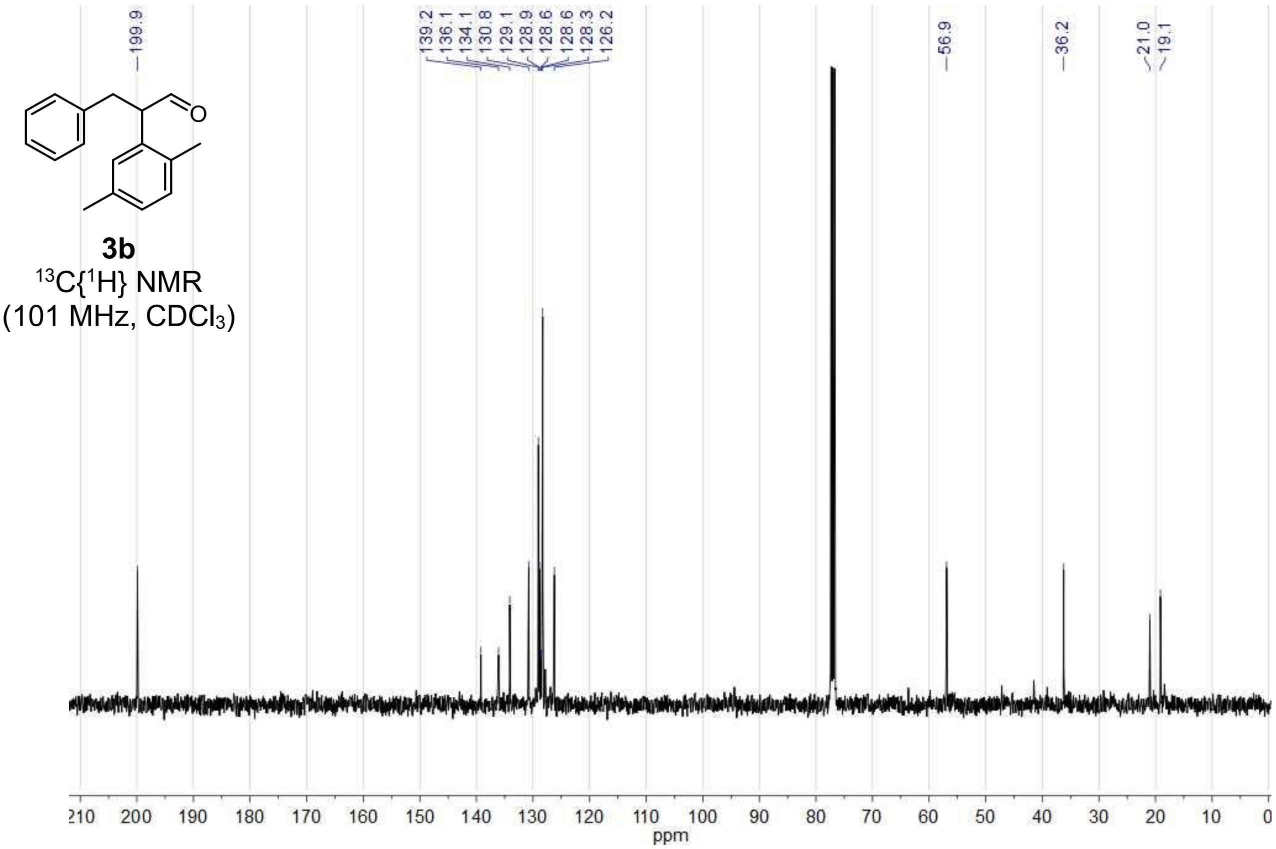

3-(4-Methoxyphenyl)-3-phenyloxetane (4)

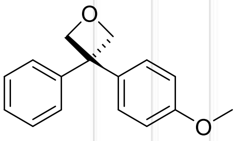

4
^{1}H NMR
(400 MHz, $CDCl_3$)

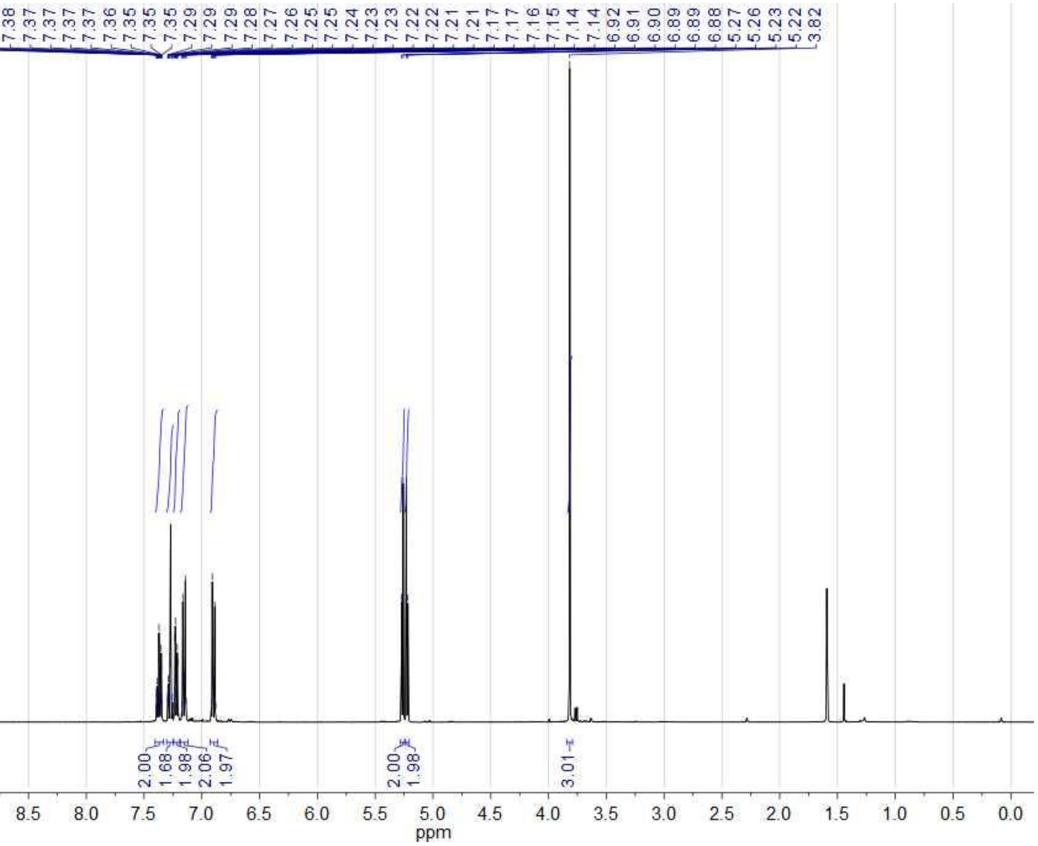

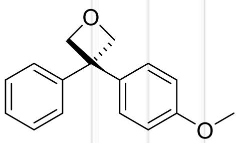

4
$^{13}C\{^{1}H\}$ NMR
(101 MHz, $CDCl_3$)

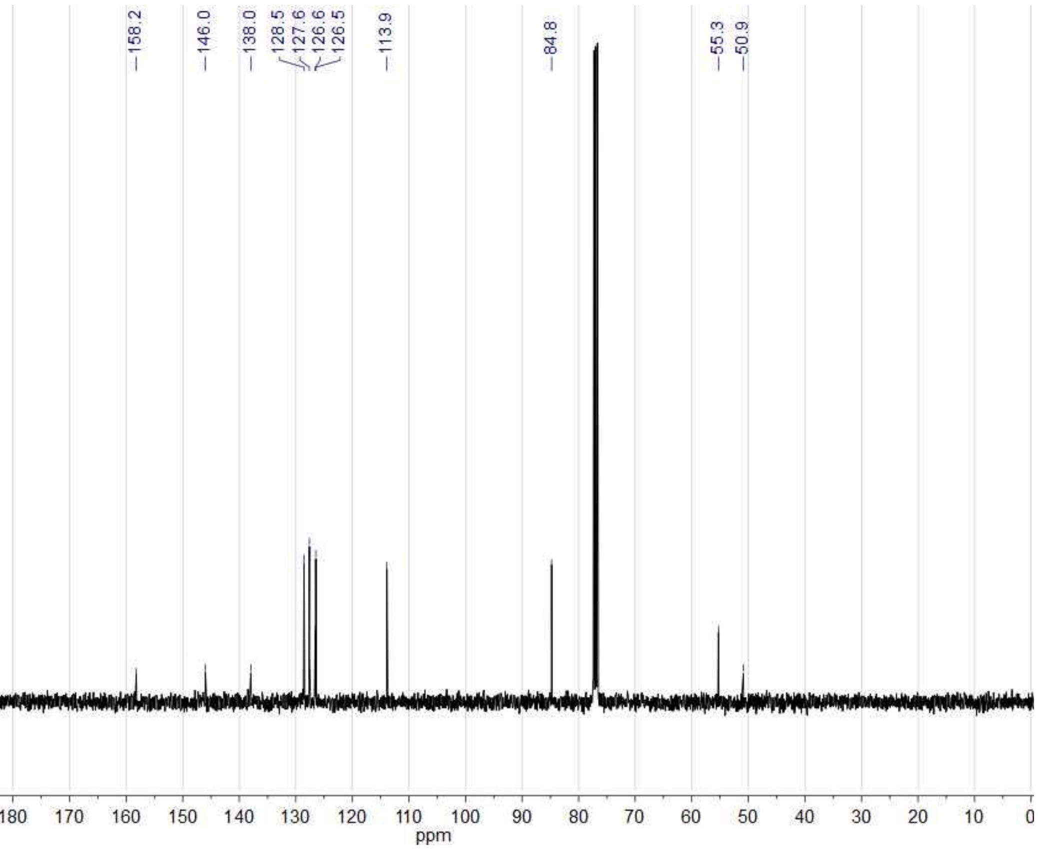

2-(4-Methoxyphenyl)-3-phenylpropanal (4b)

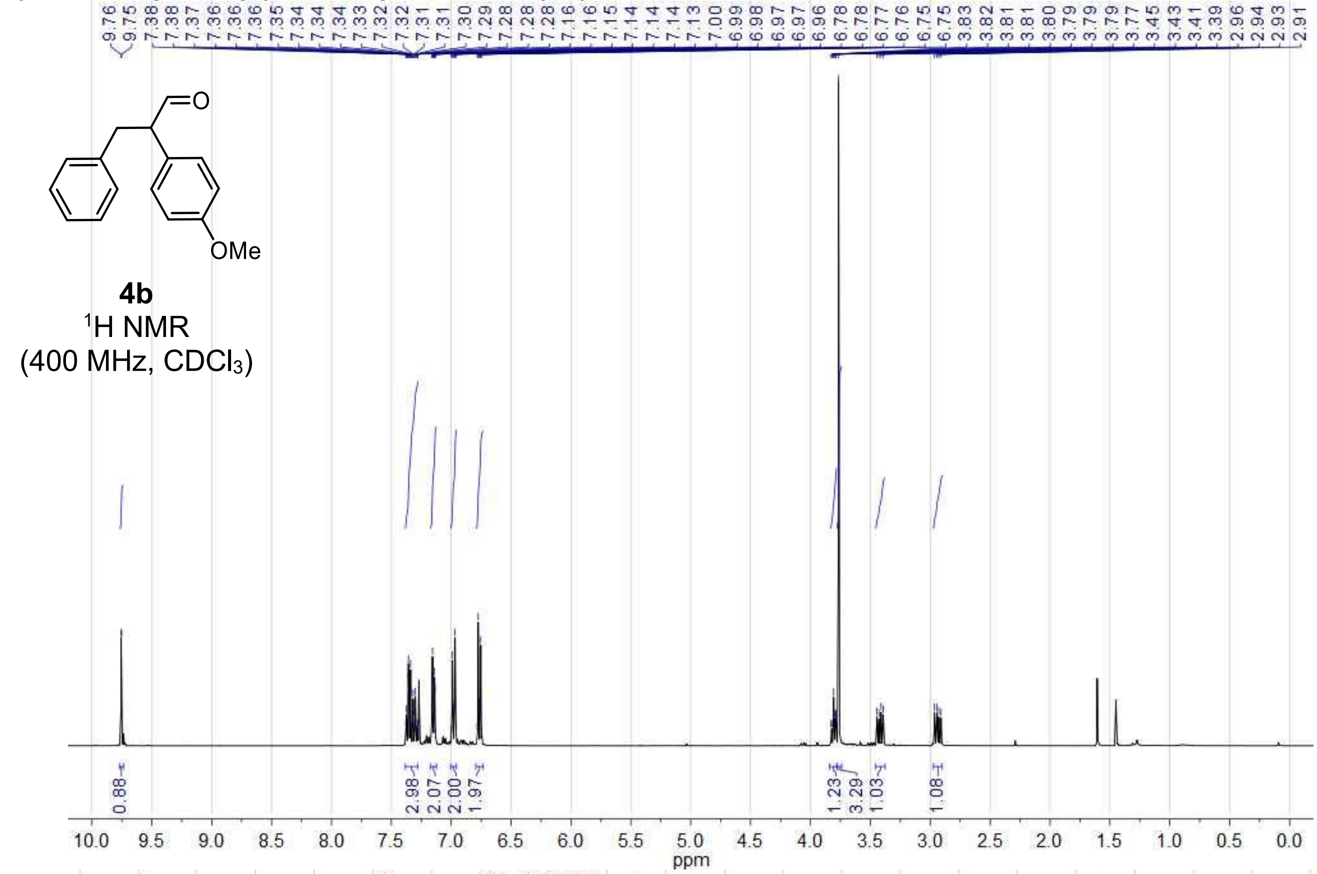

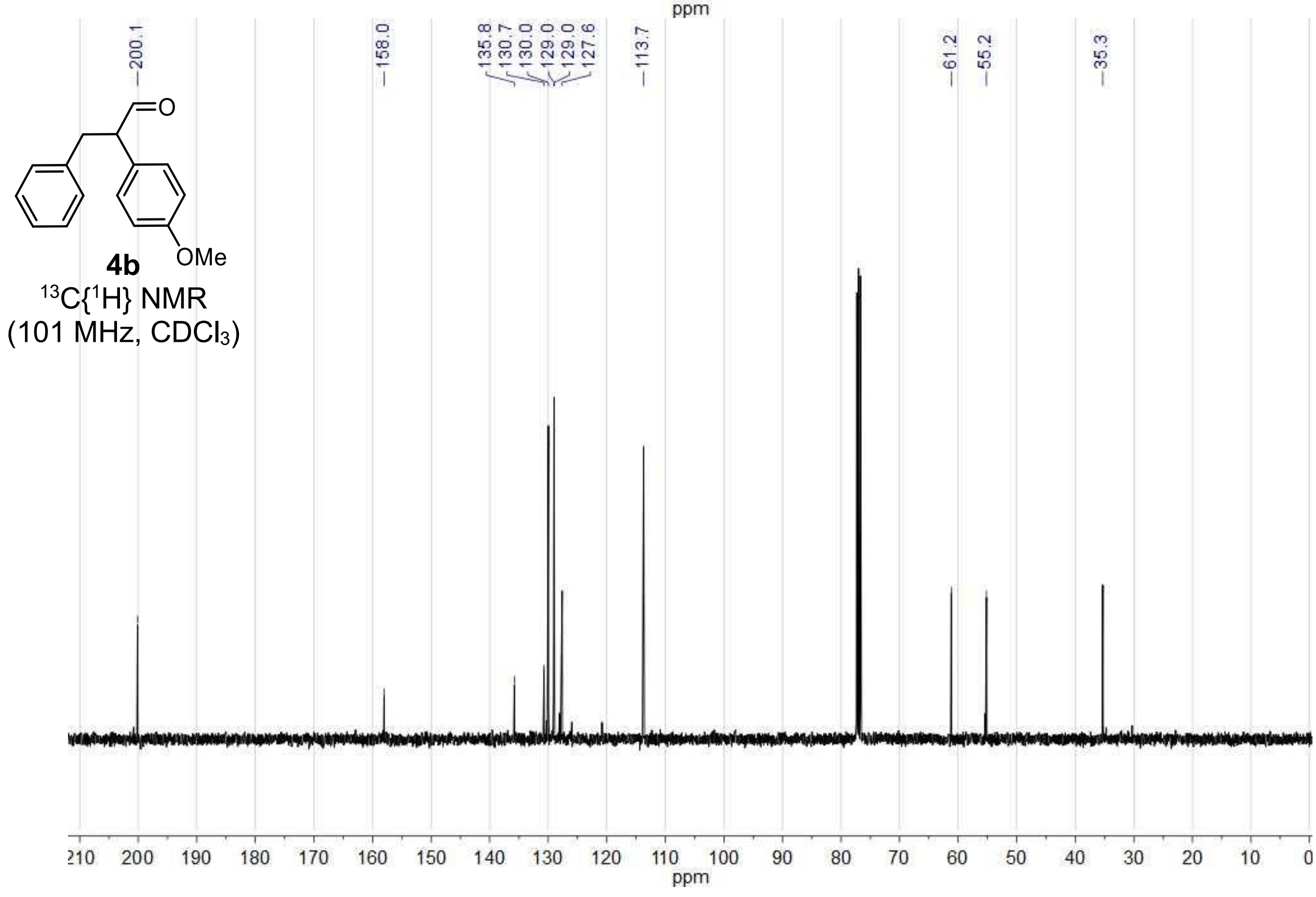

3-Mesityl-3-phenyloxetane (5)

5
^{1}H NMR
(400 MHz, $CDCl_3$)

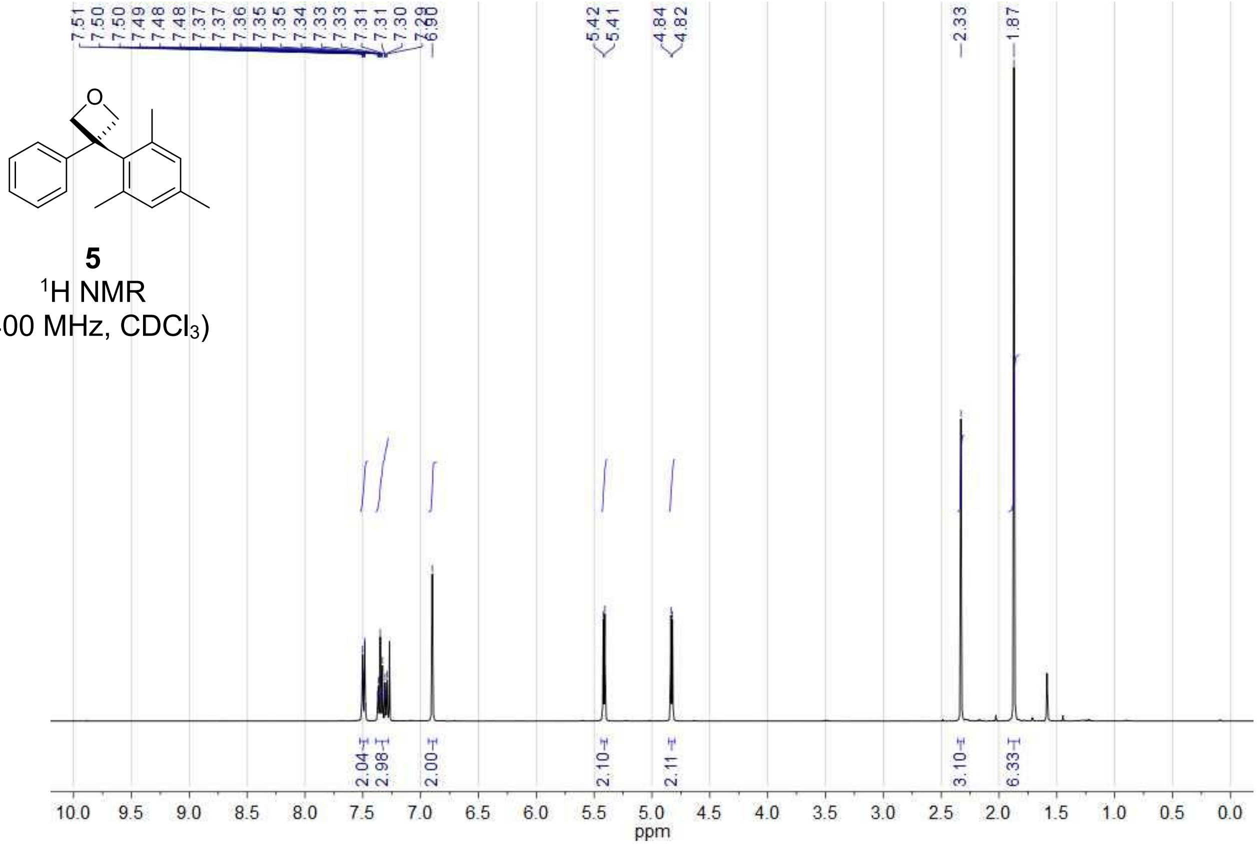

5
$^{13}C\{^{1}H\}$ NMR
(101 MHz, $CDCl_3$)

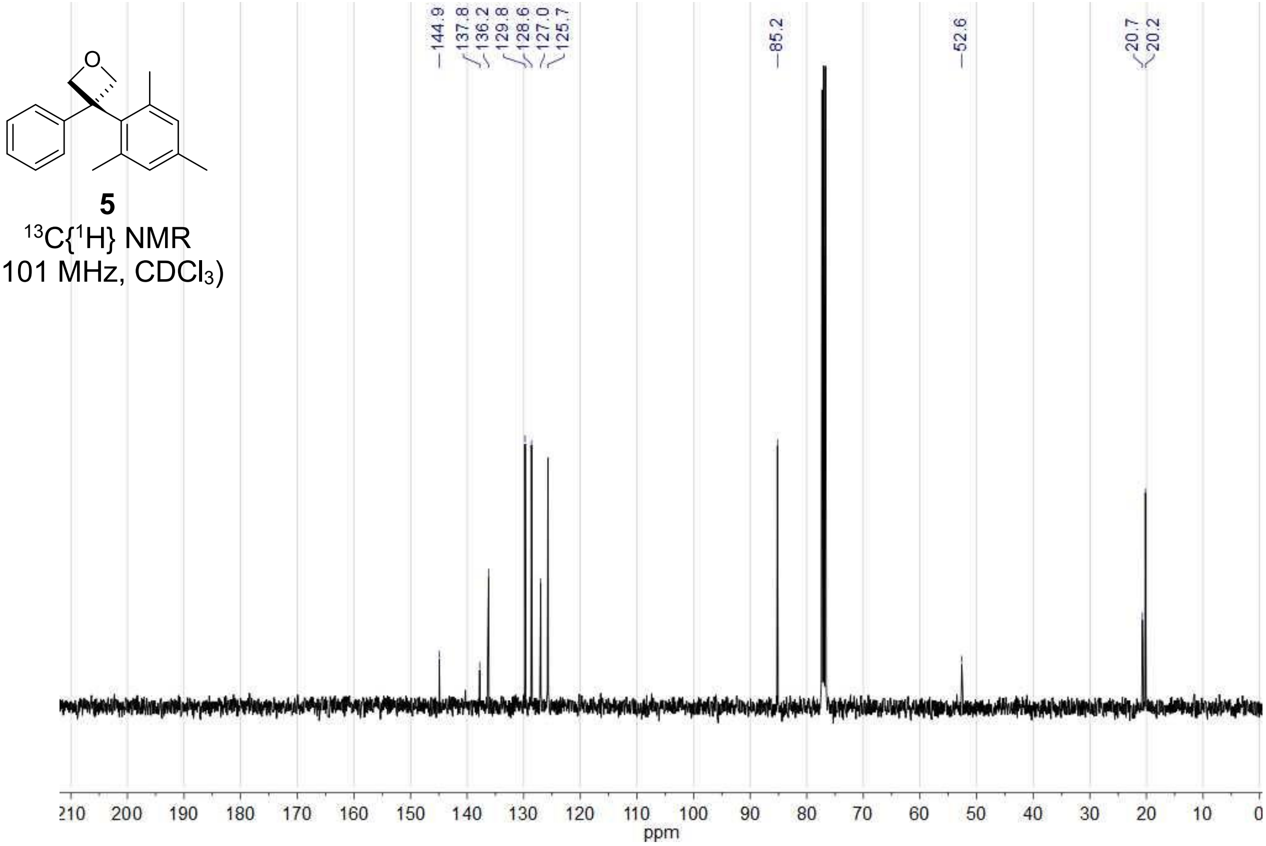

3-Phenyl-3-(2,3,5,6-tetramethylphenyl)oxetane (6)

6
^{1}H NMR
(400 MHz, $CDCl_3$)

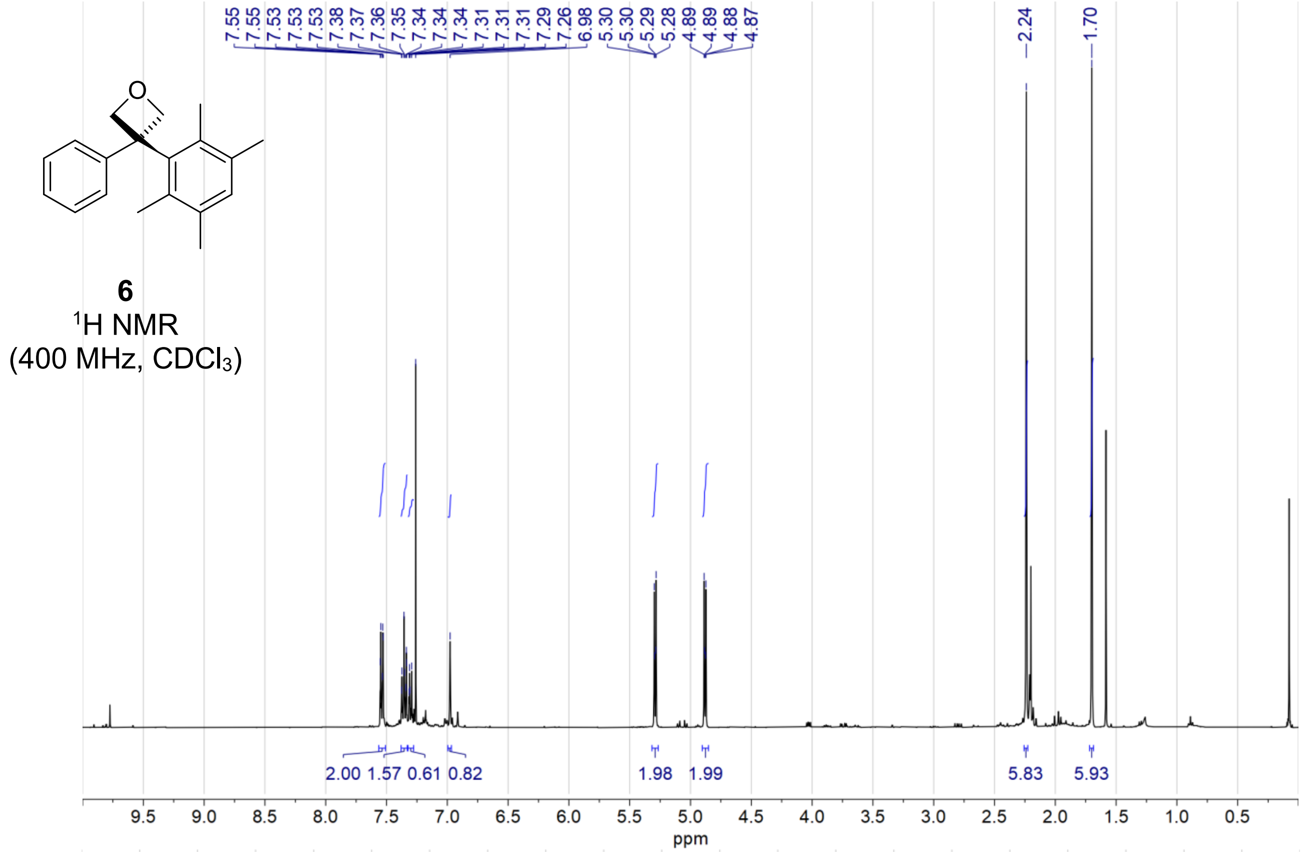

6
^{13}C{^{1}H} NMR
(101 MHz, $CDCl_3$)

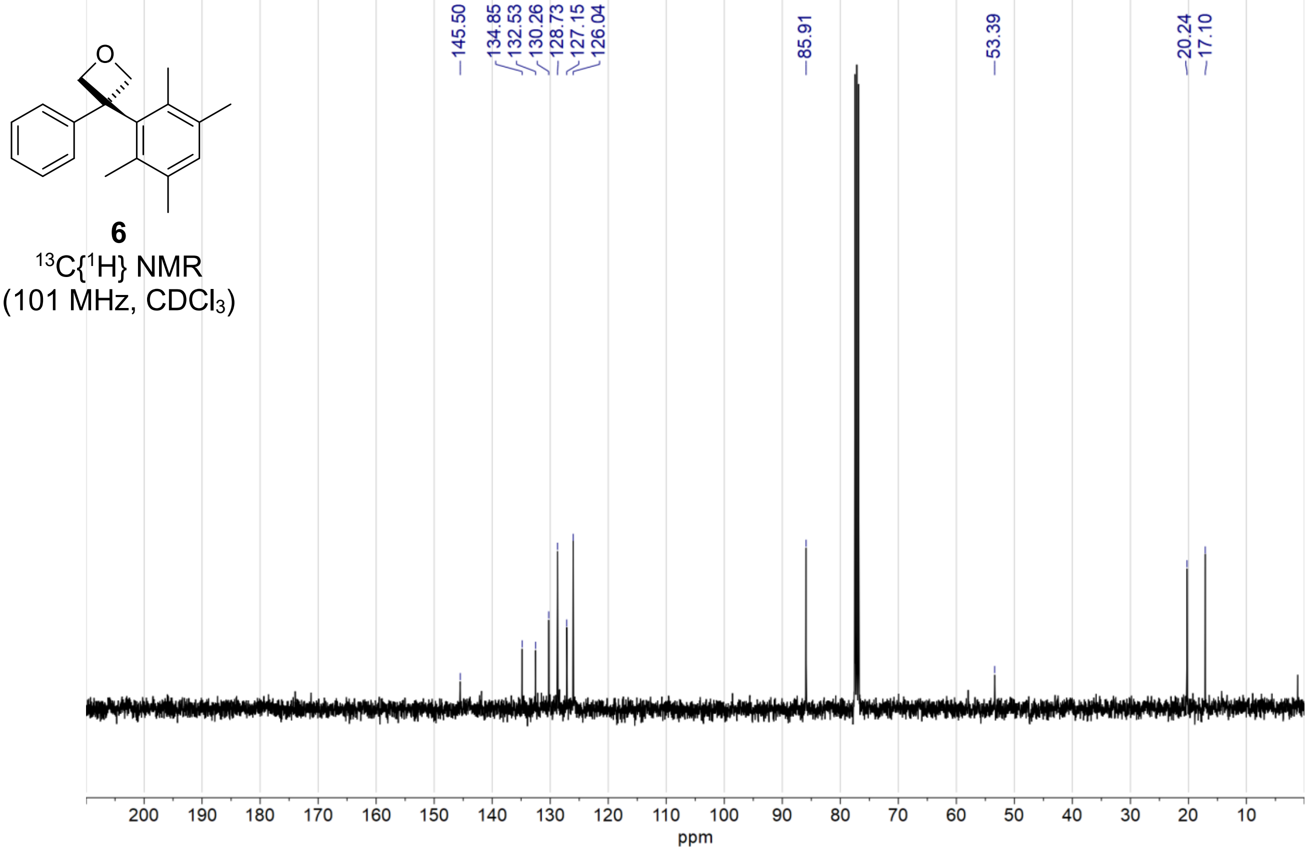

3-(5-Isopropyl-2-methylphenyl)-3-phenyloxetane (7)

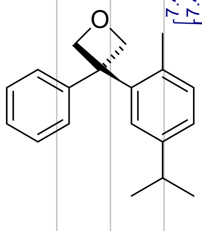

7

^{1}H NMR

(400 MHz, $CDCl_3$)

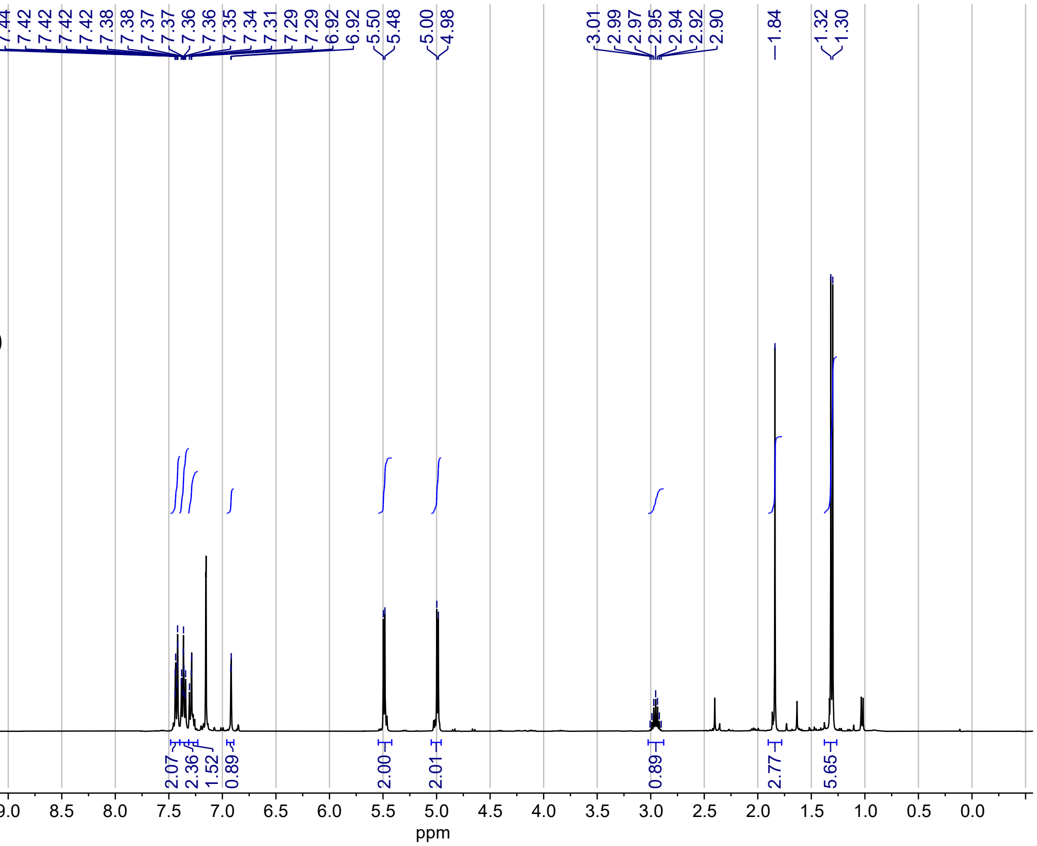

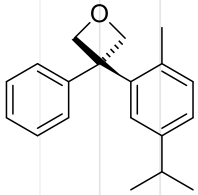

7

$^{13}C\{^{1}H\}$ NMR

(101 MHz, $CDCl_3$)

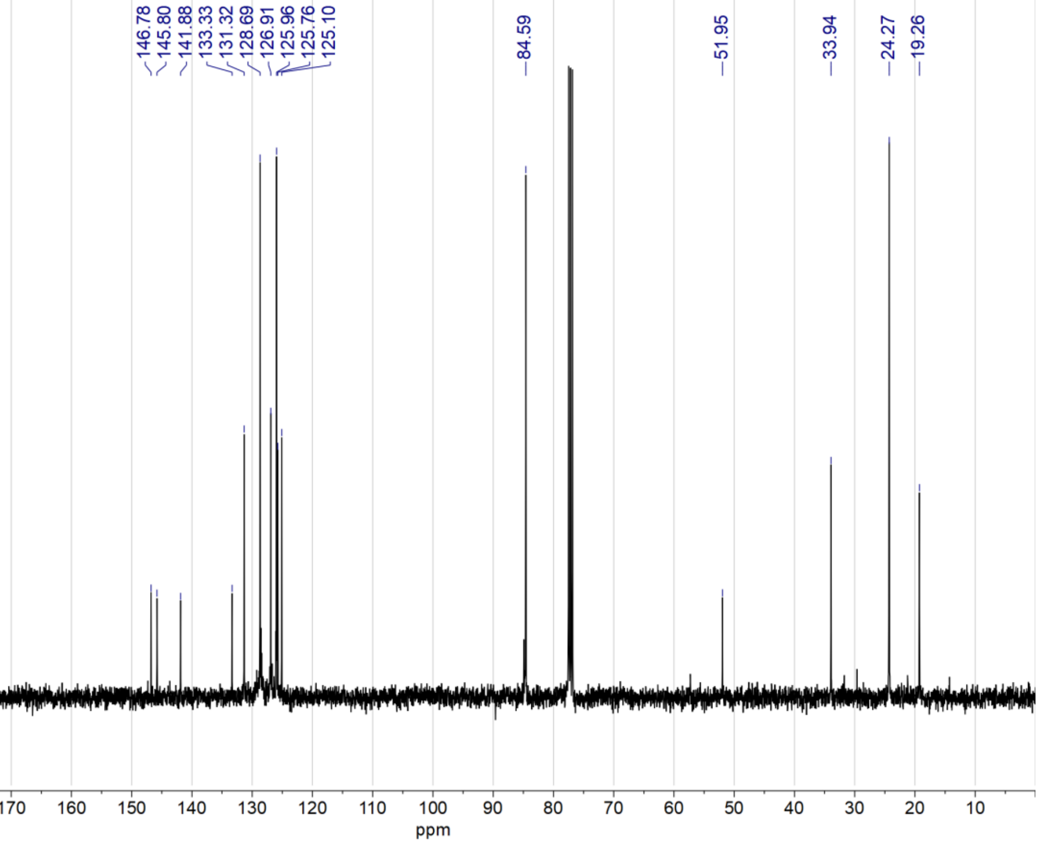

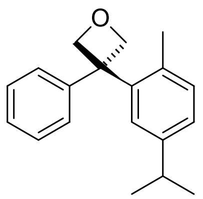

7

$^1H - ^{13}C$ HSQC

(400 MHz, $CDCl_3$)

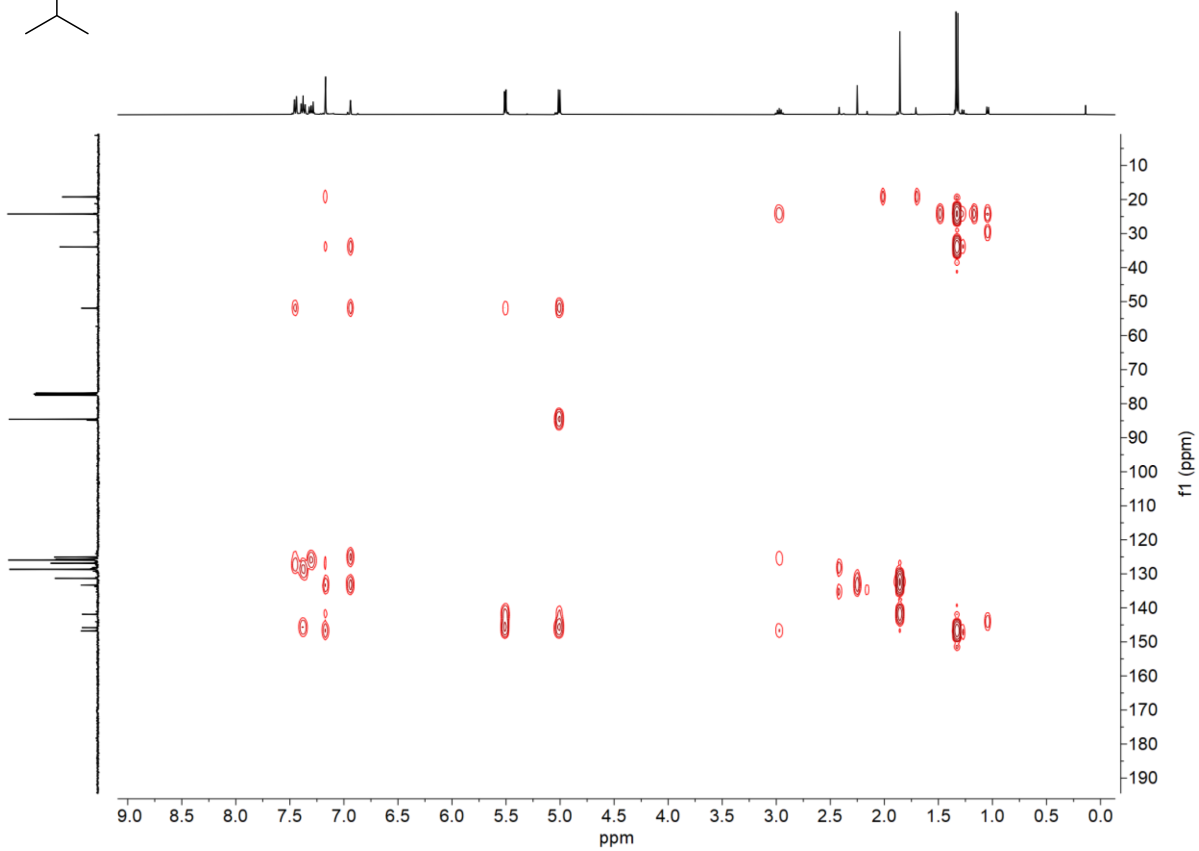

3,3-Diphenyloxetane (8)

8
^{1}H NMR
(400 MHz, $CDCl_3$)

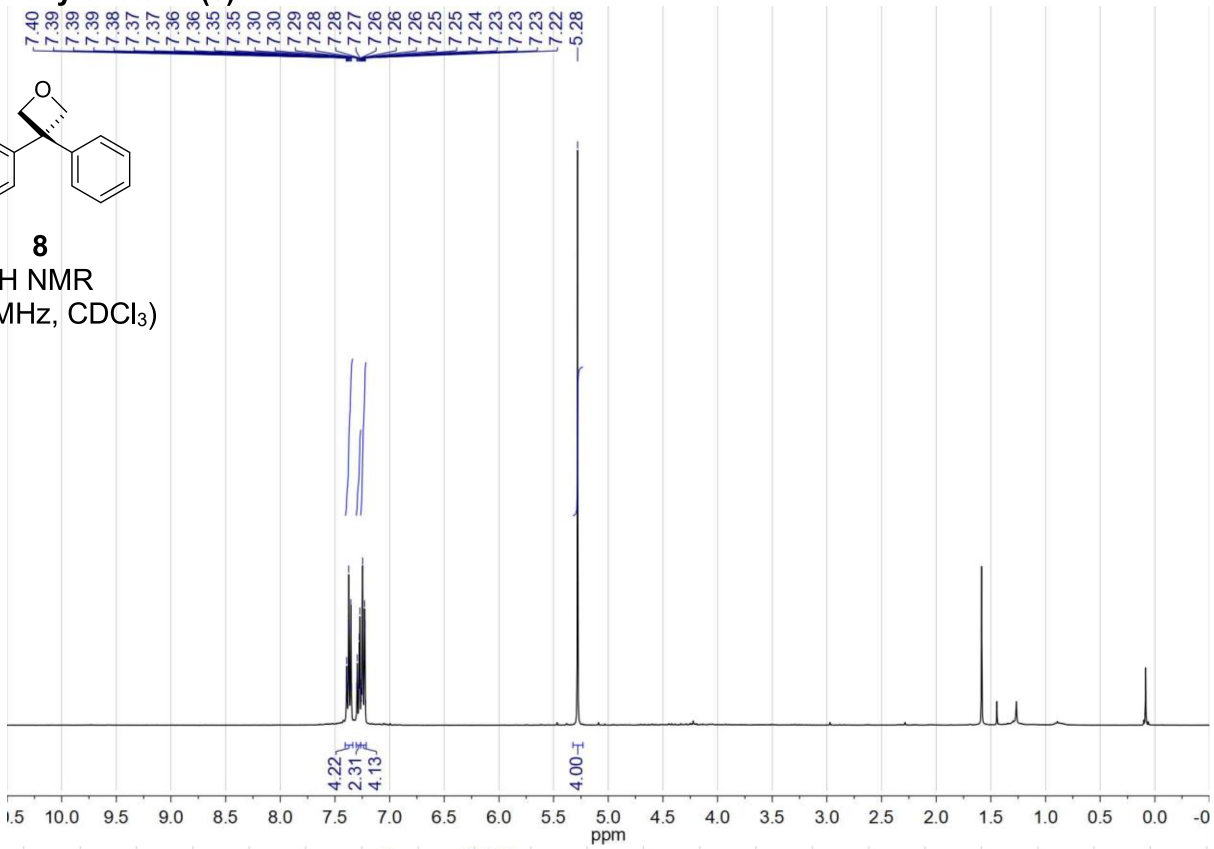

8
$^{13}C\{^{1}H\}$ NMR
(101 MHz, $CDCl_3$)

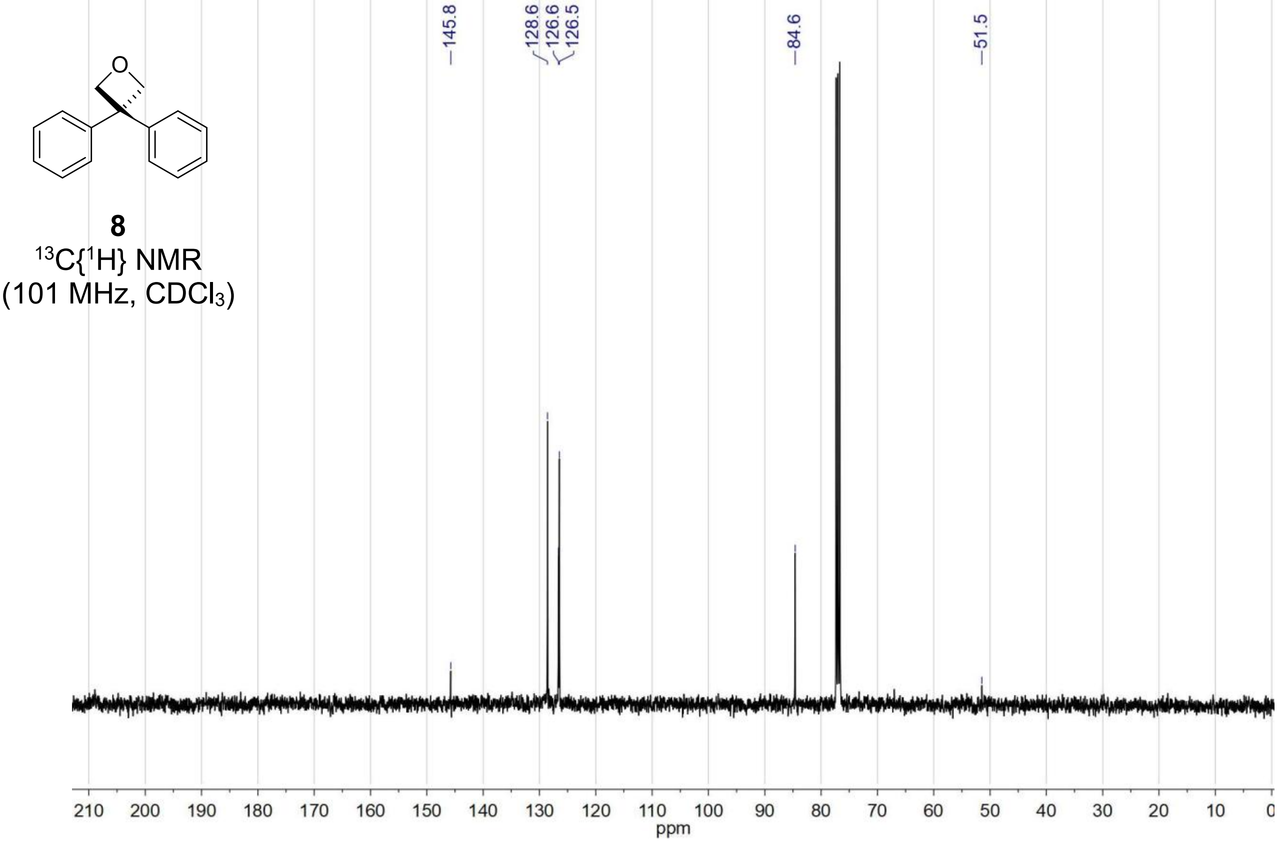

3-Phenyl-3-(*o*-tolyl)oxetane (*o*-9)

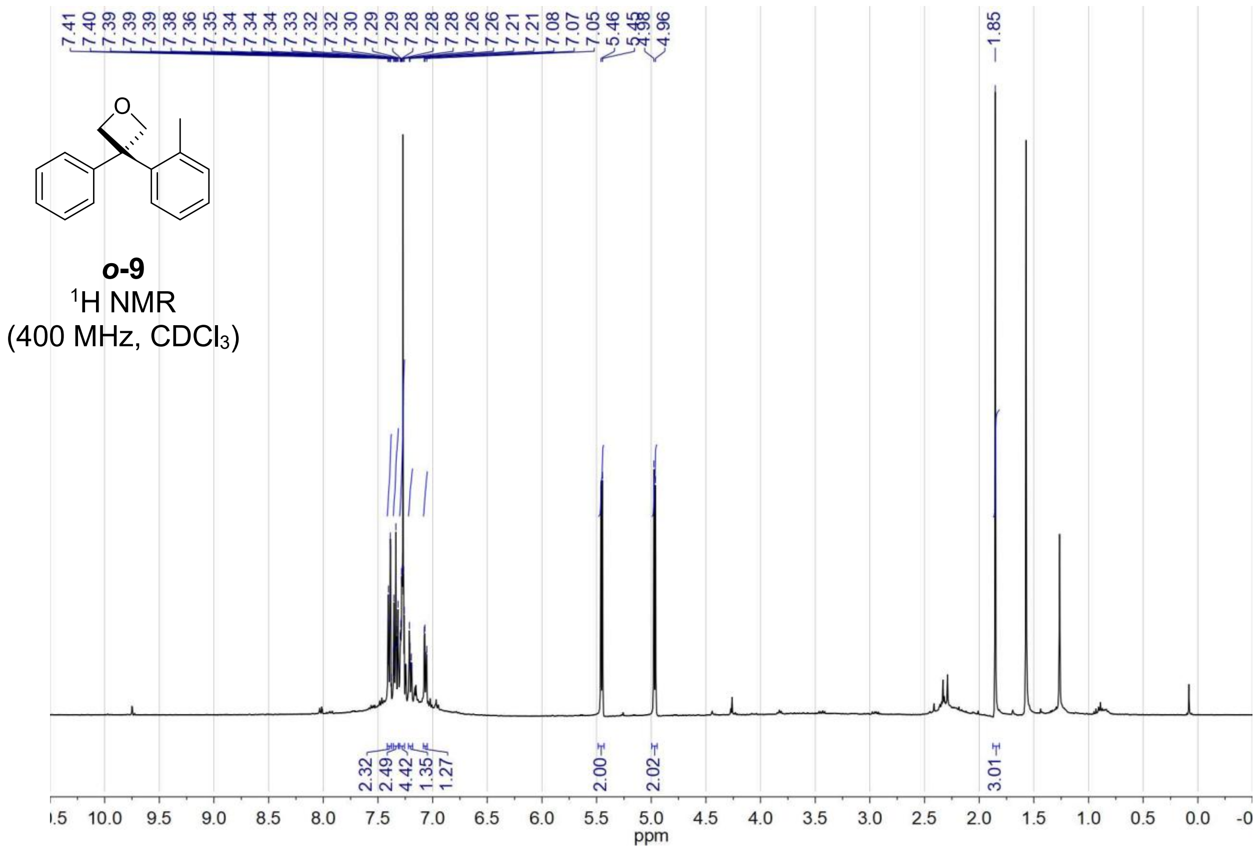

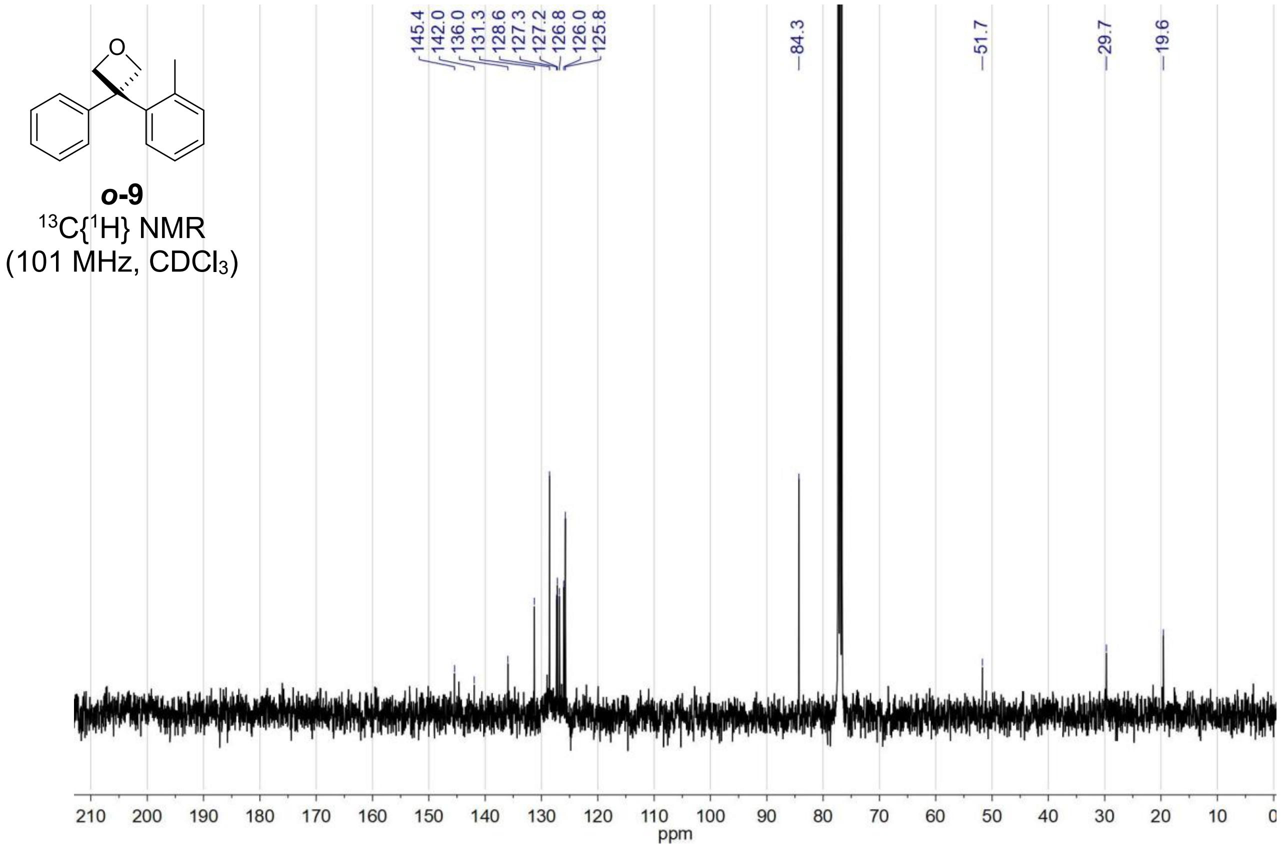

3-Phenyl-3-(*p*-tolyl)oxetane (*p*-9)

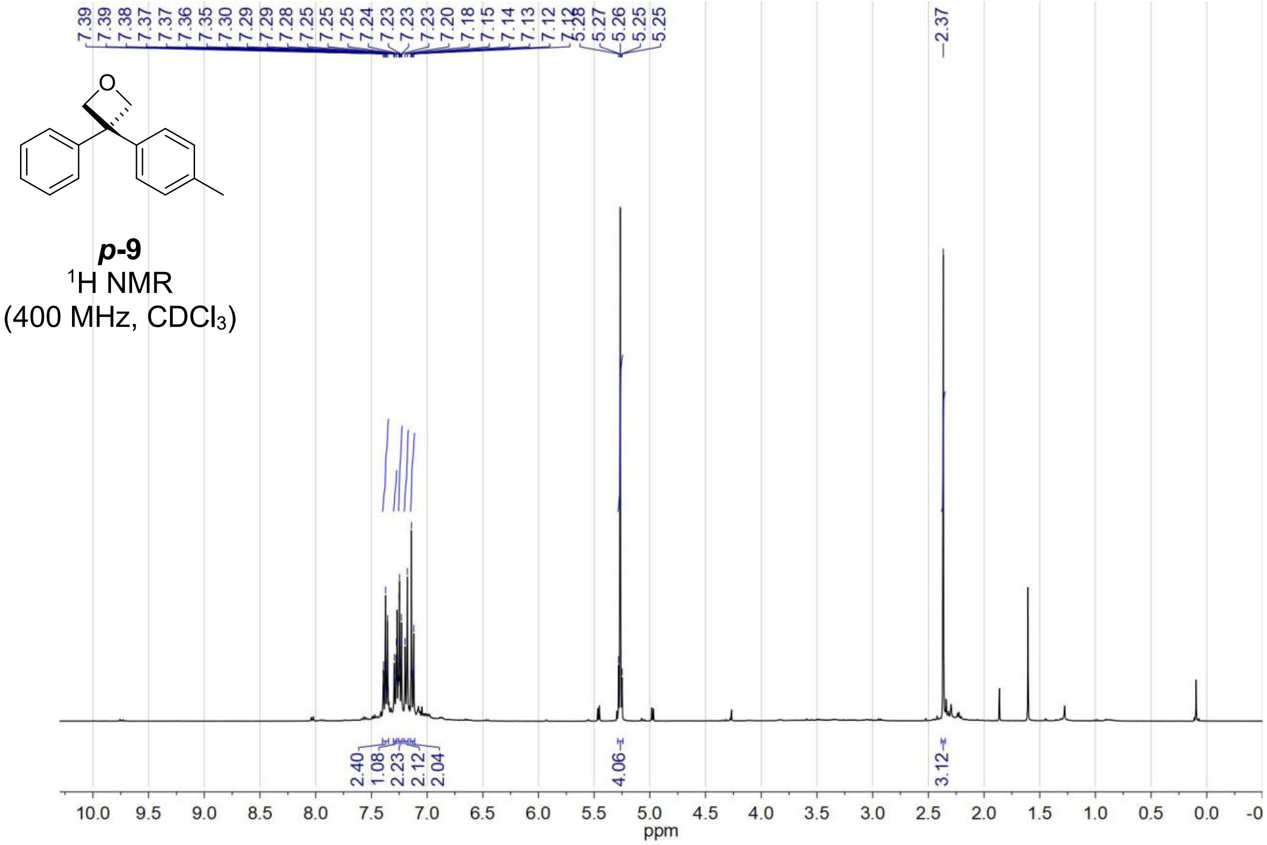

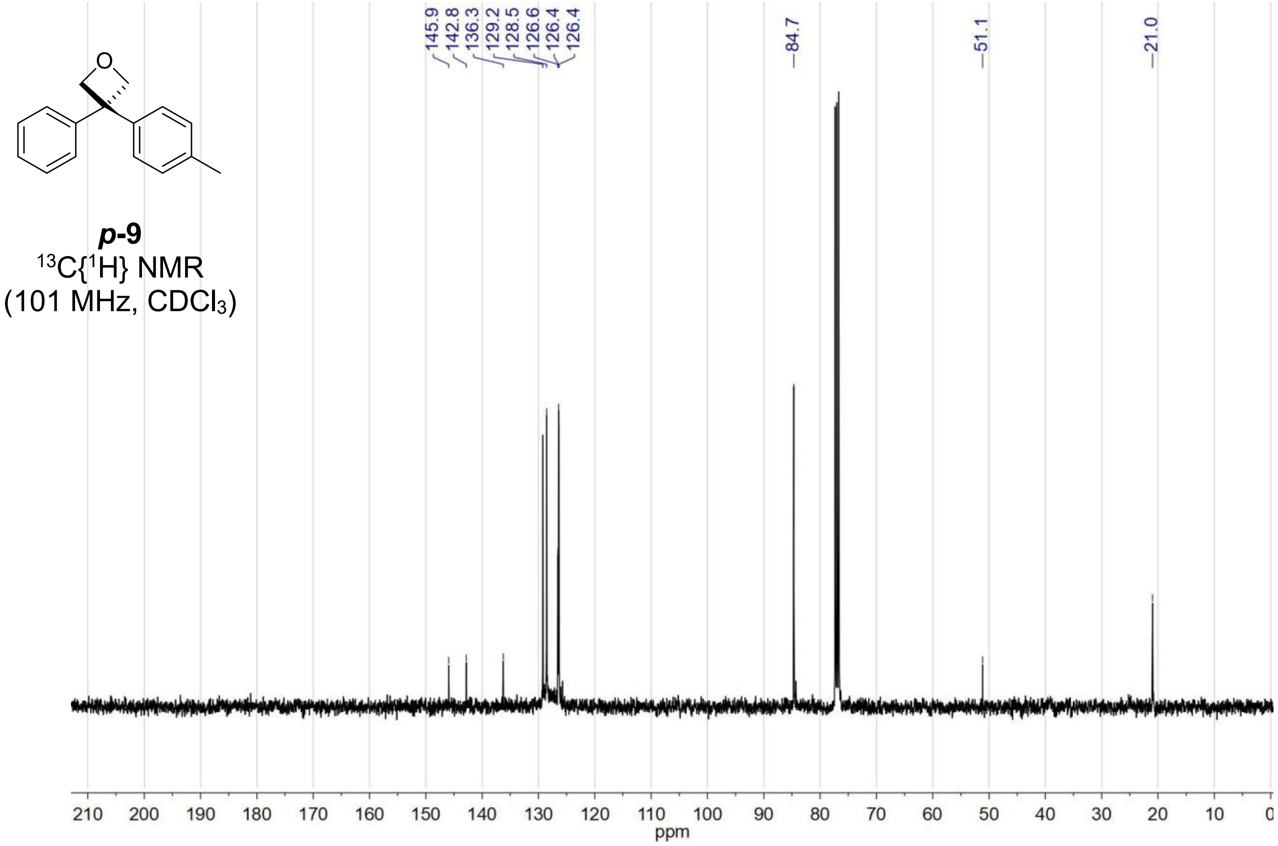

2-Phenyl-3-(p-tolyl)propanal (9a)

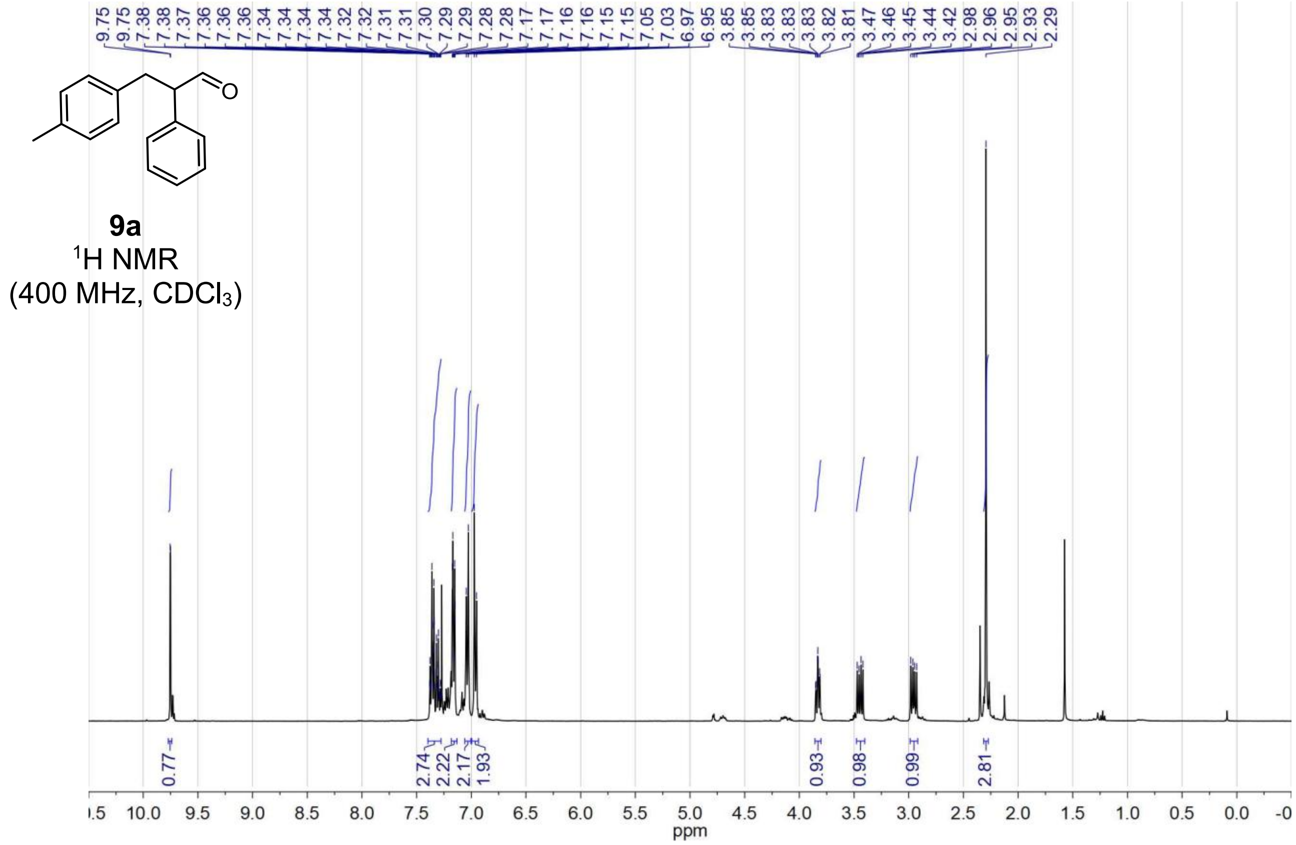

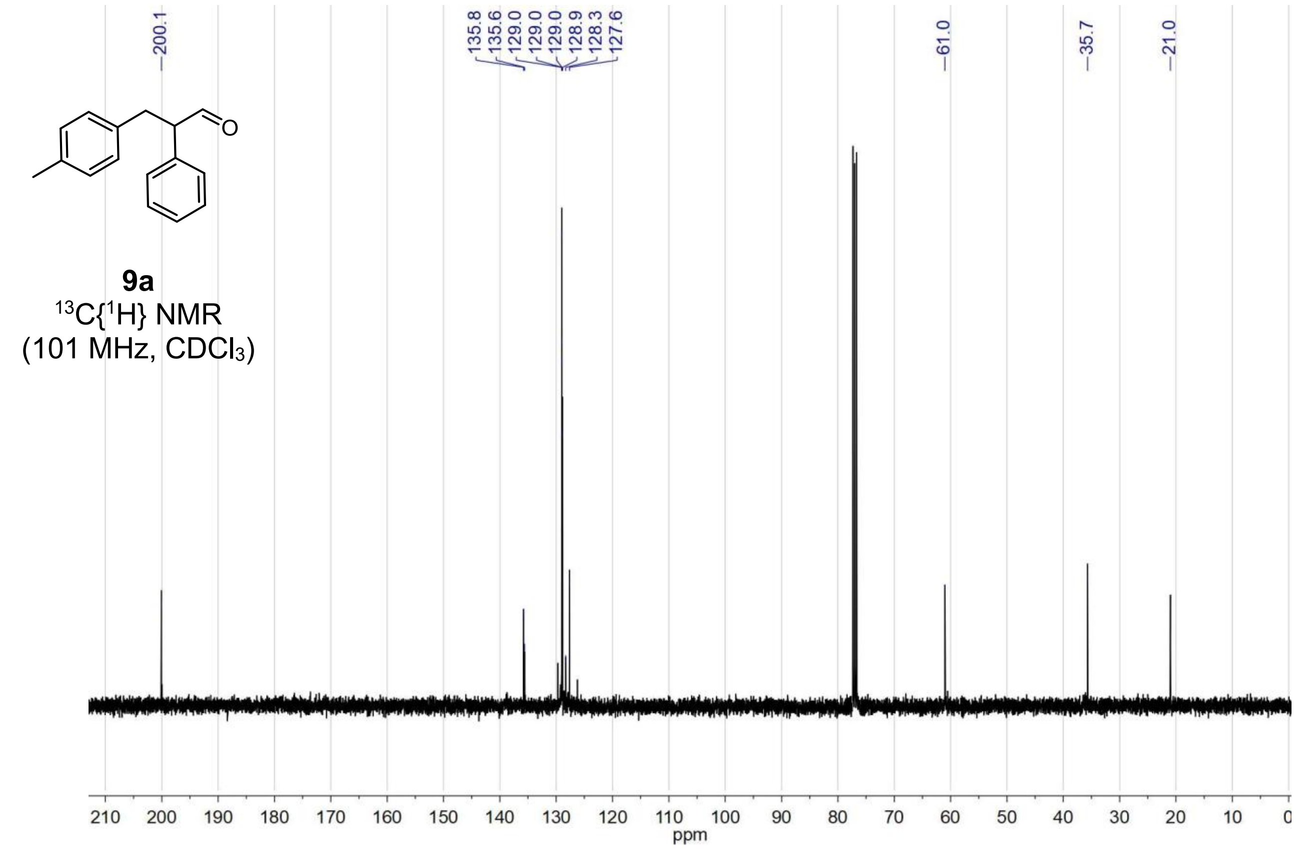

3-Mesityl-3-(*p*-tolyl)oxetane (10)

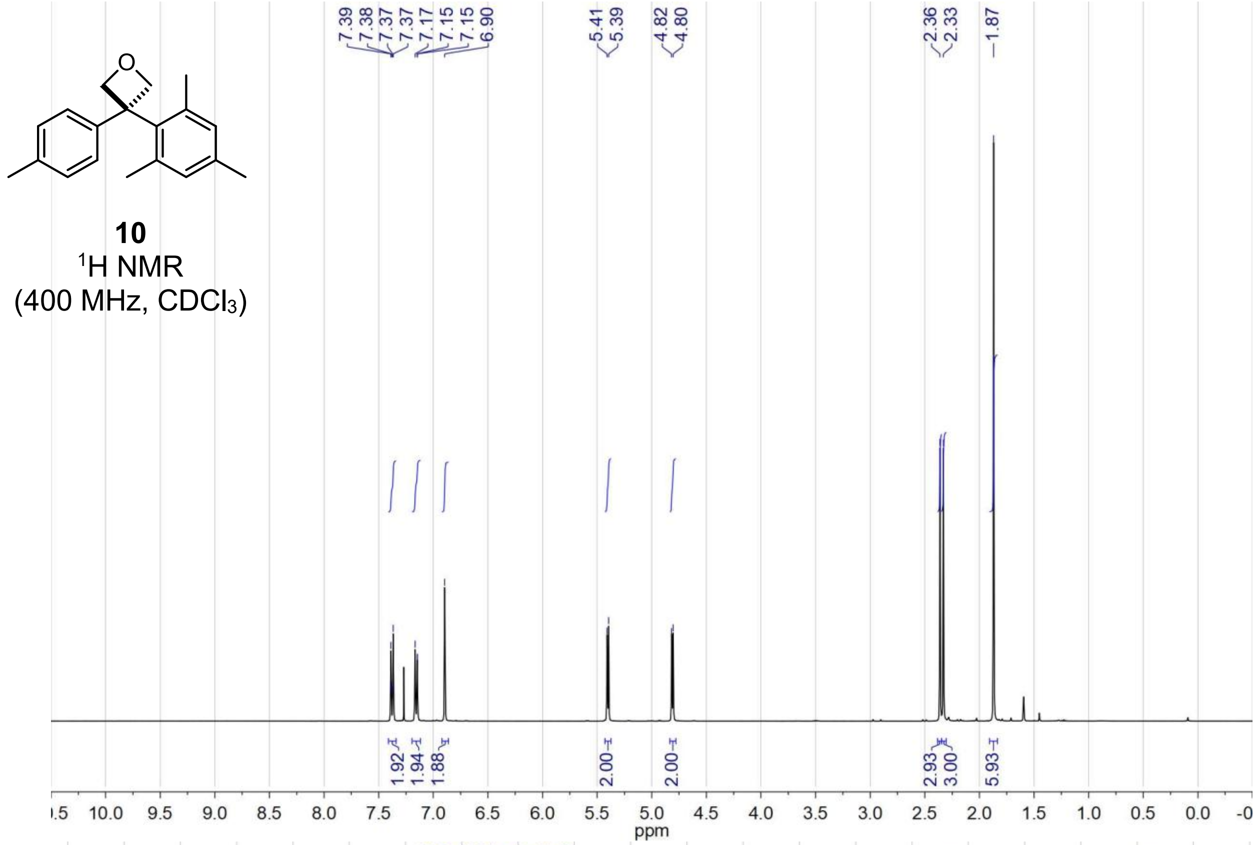

10
^{1}H NMR
(400 MHz, $CDCl_3$)

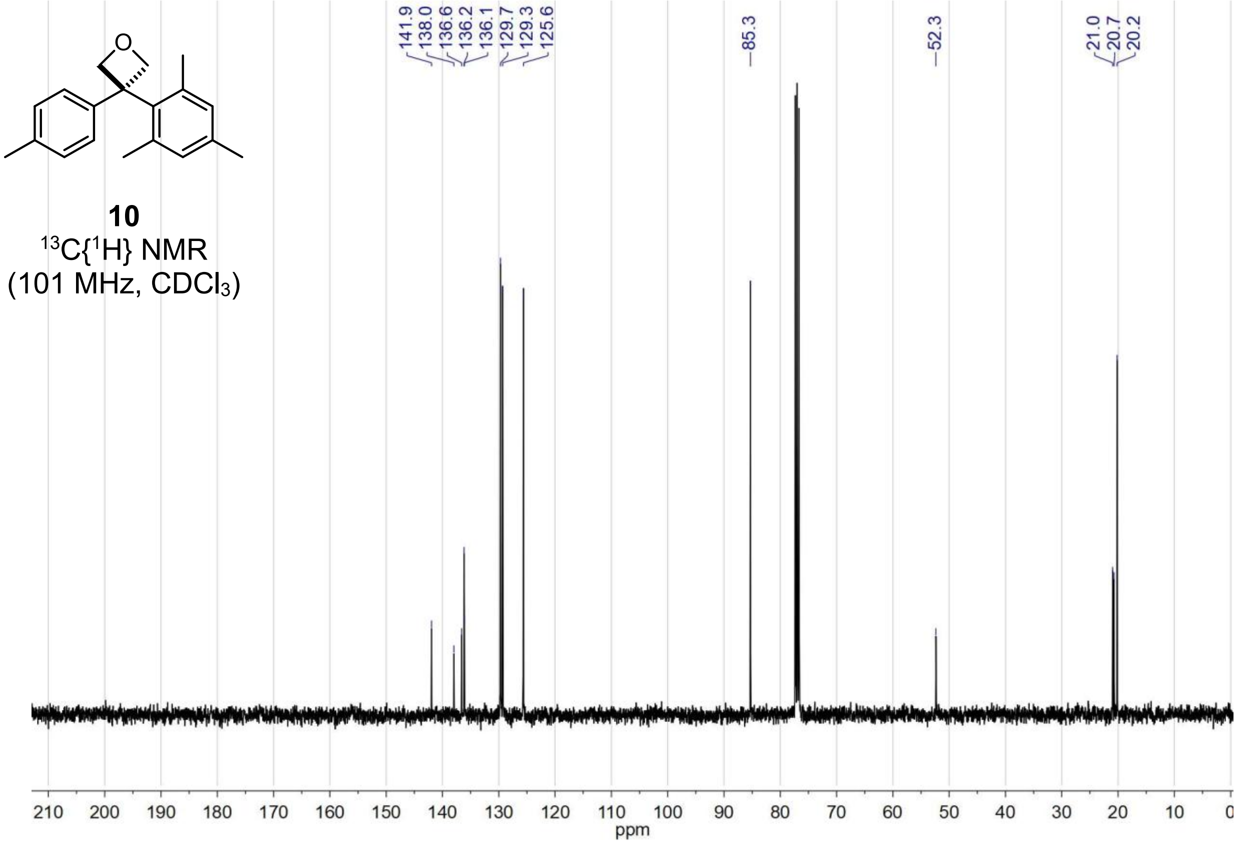

10
$^{13}C\{^1H\}$ NMR
(101 MHz, $CDCl_3$)

3-Mesityl-2-(*p*-tolyl)propanal (10a) and 2-mesityl-3-(*p*-tolyl)propanal (10b)

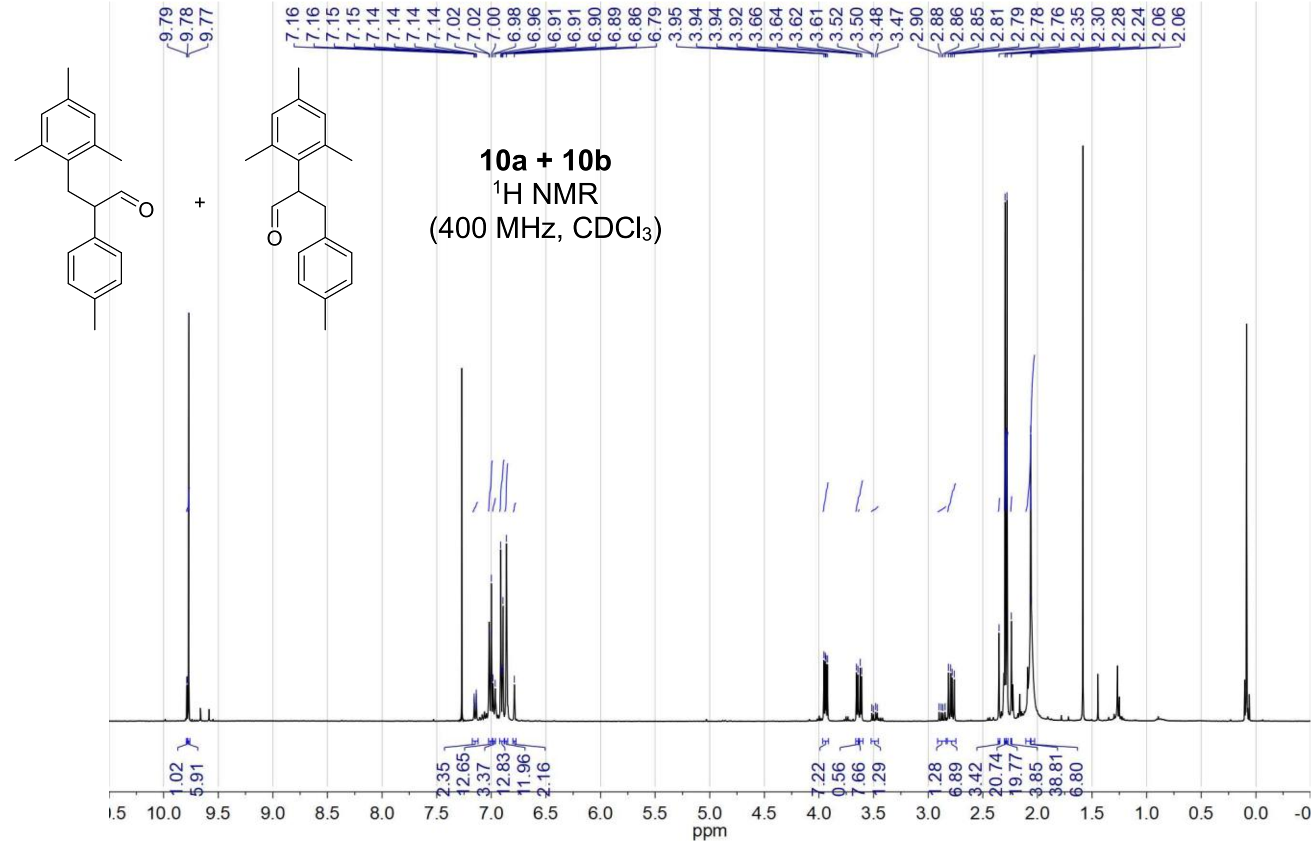

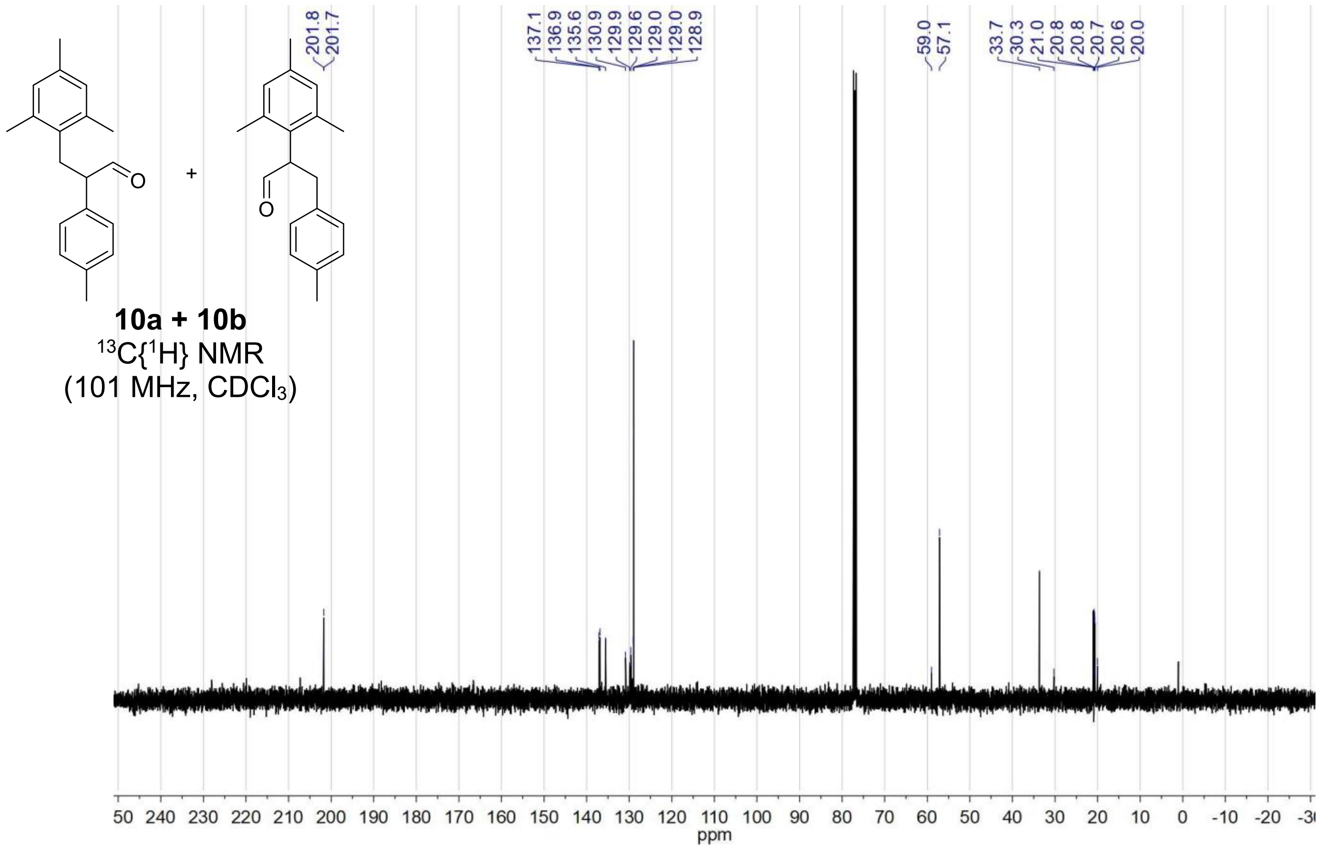

3-(4-Fluorophenyl)-3-mesityloxetane (11)

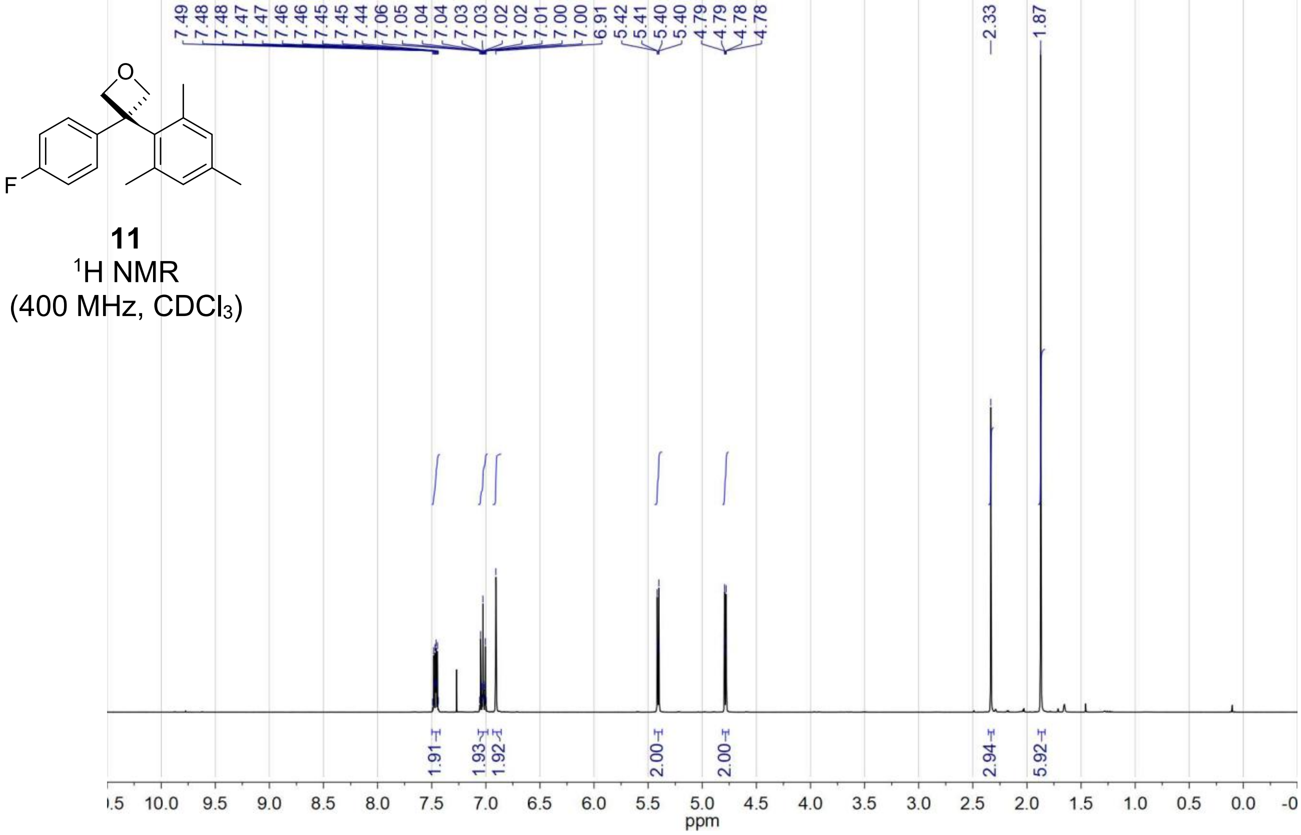

11
^{1}H NMR
(400 MHz, $CDCl_3$)

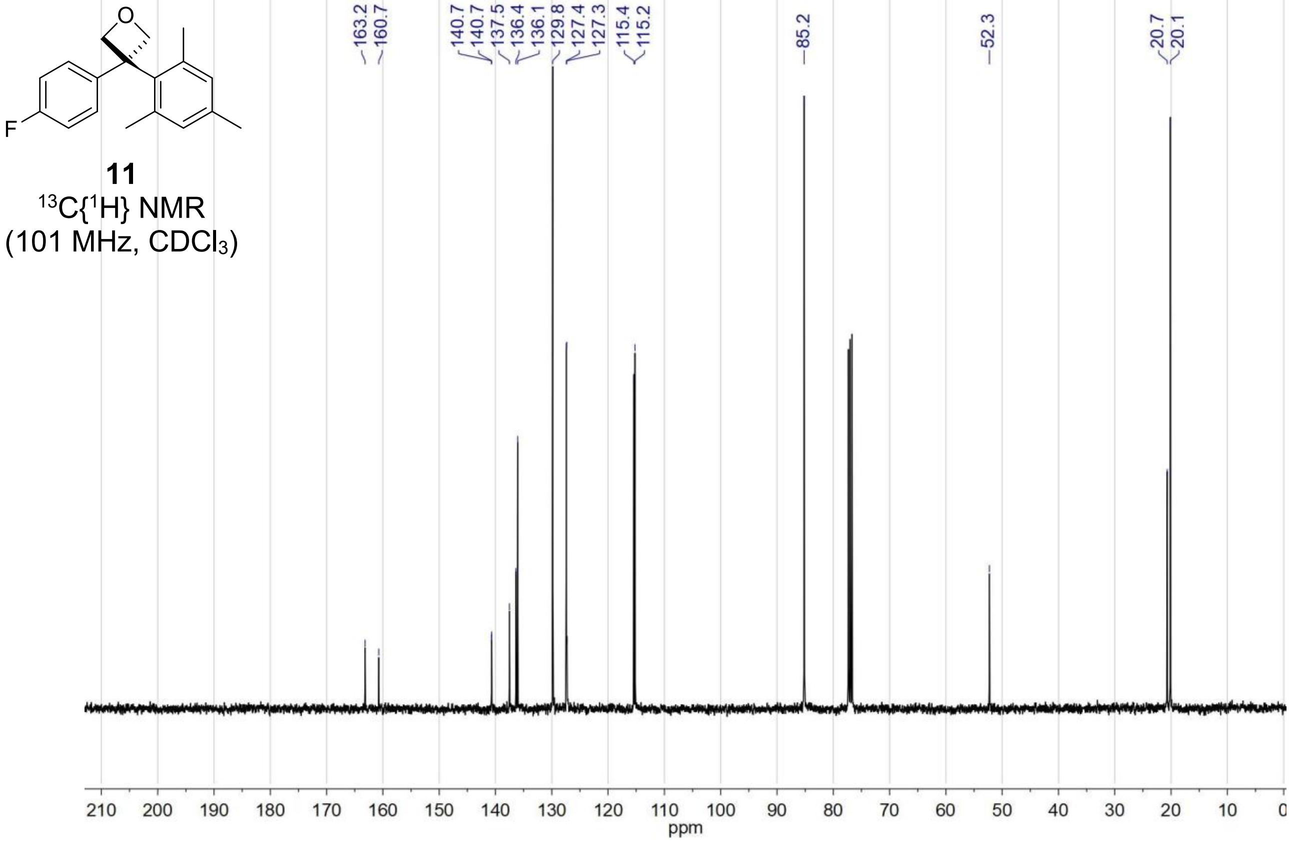

11
^{13}C{^{1}H} NMR
(101 MHz, $CDCl_3$)

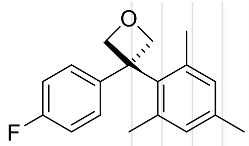

11

^{19}F NMR

(377 MHz, $CDCl_3$)

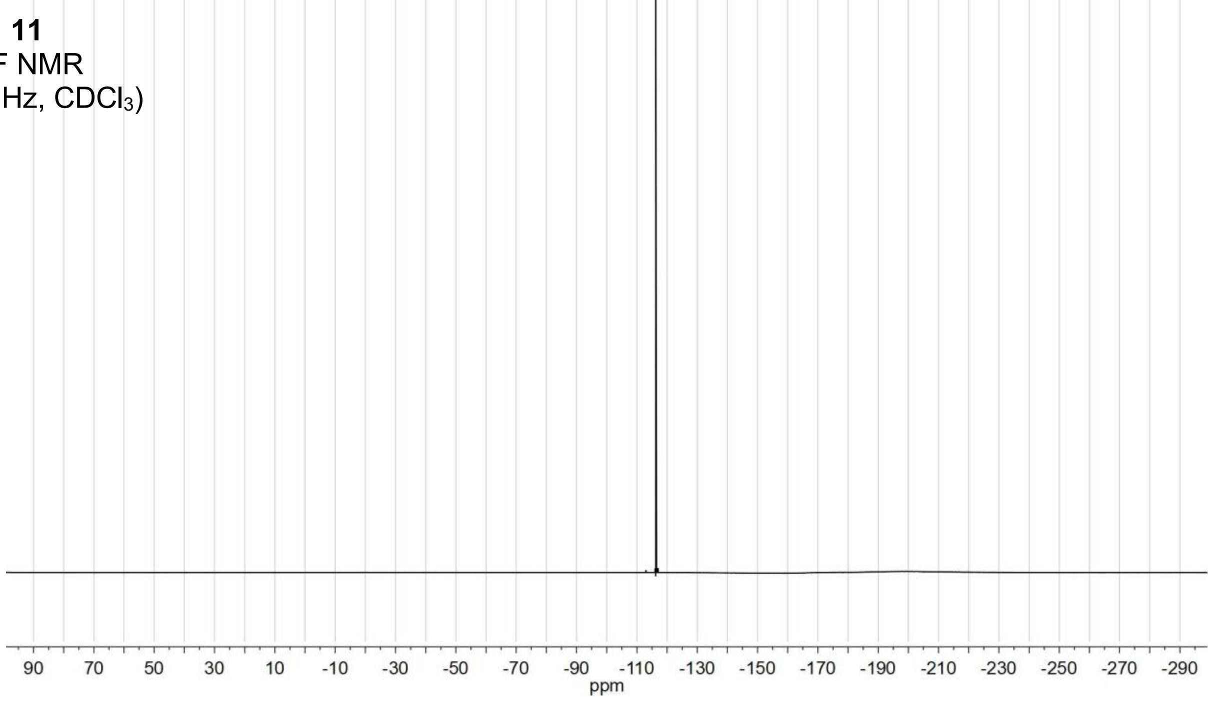

2-(4-Fluorophenyl)-3-mesitylpropanal (11a) and 3-(4-fluorophenyl)-2-mesitylpropanal (11b)

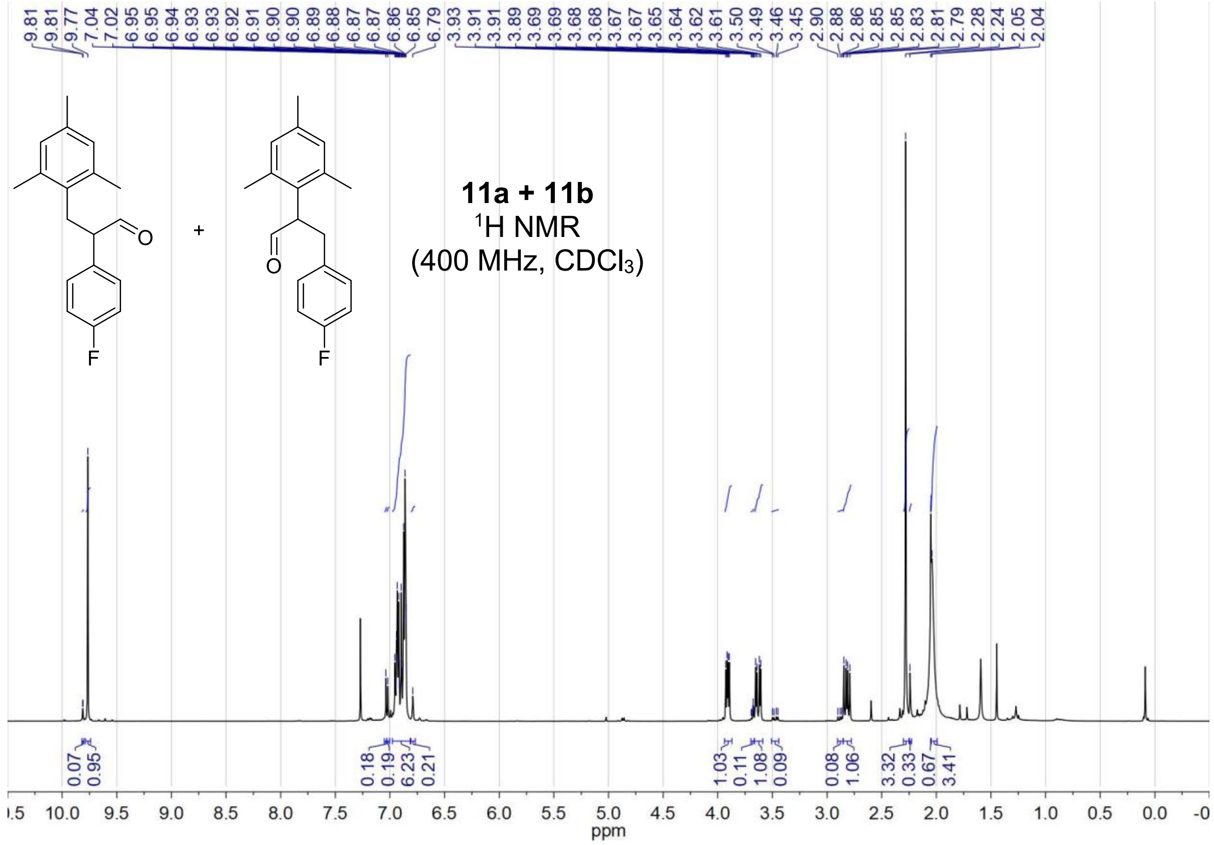

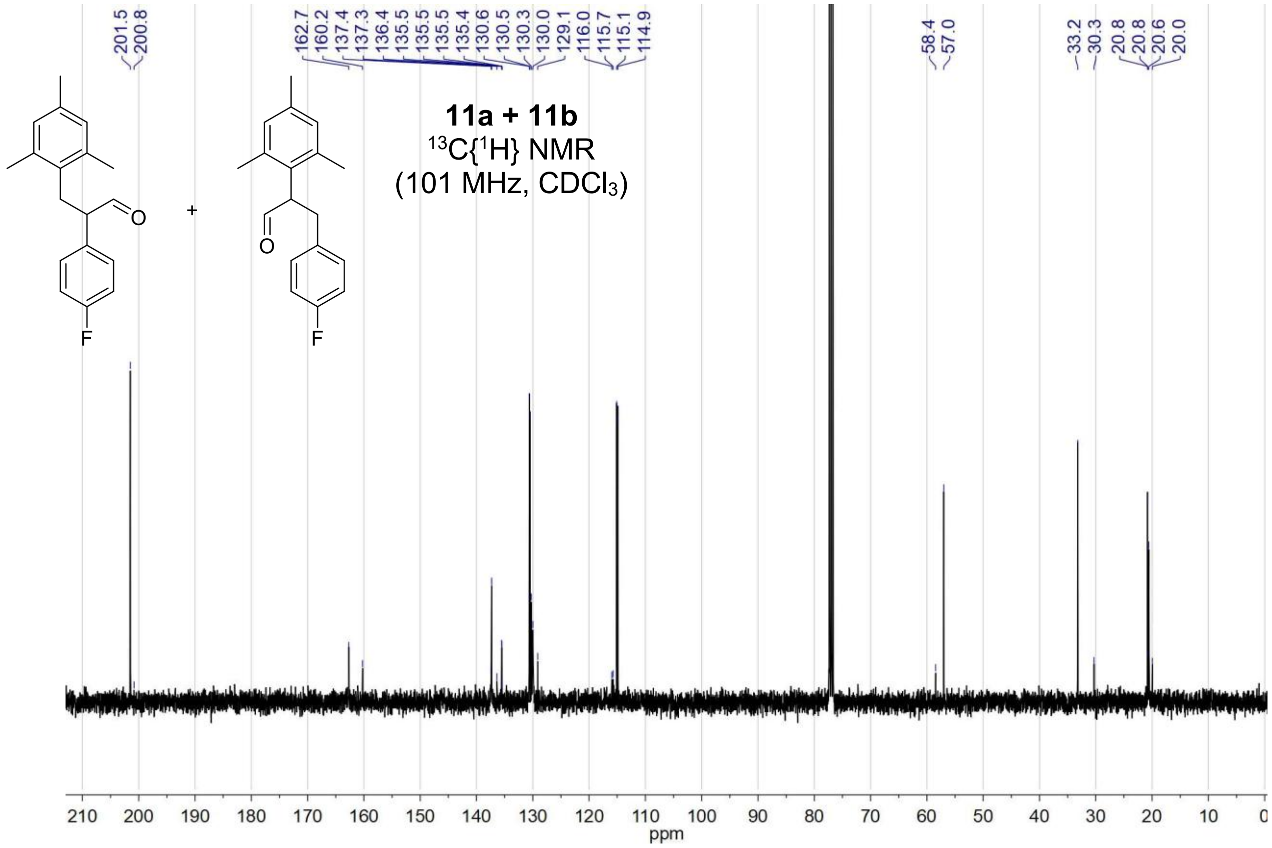

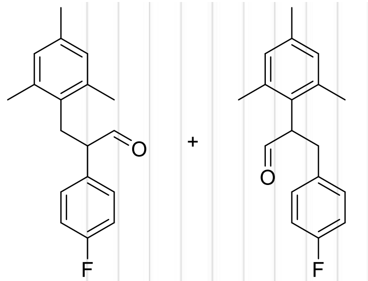

11a + 11b

^{19}F NMR

(377 MHz, $CDCl_3$)

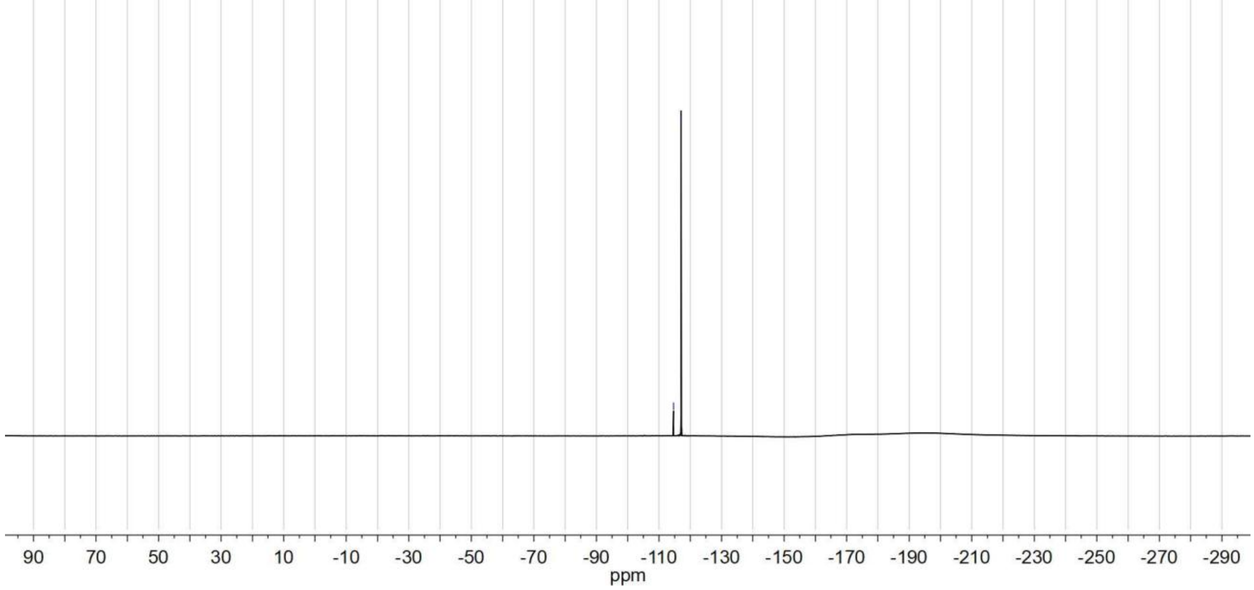

3-(4-Chlorophenyl)-3-mesityloxetane (12)

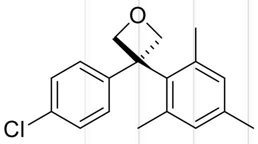

12
^{1}H NMR
(400 MHz, $CDCl_3$)

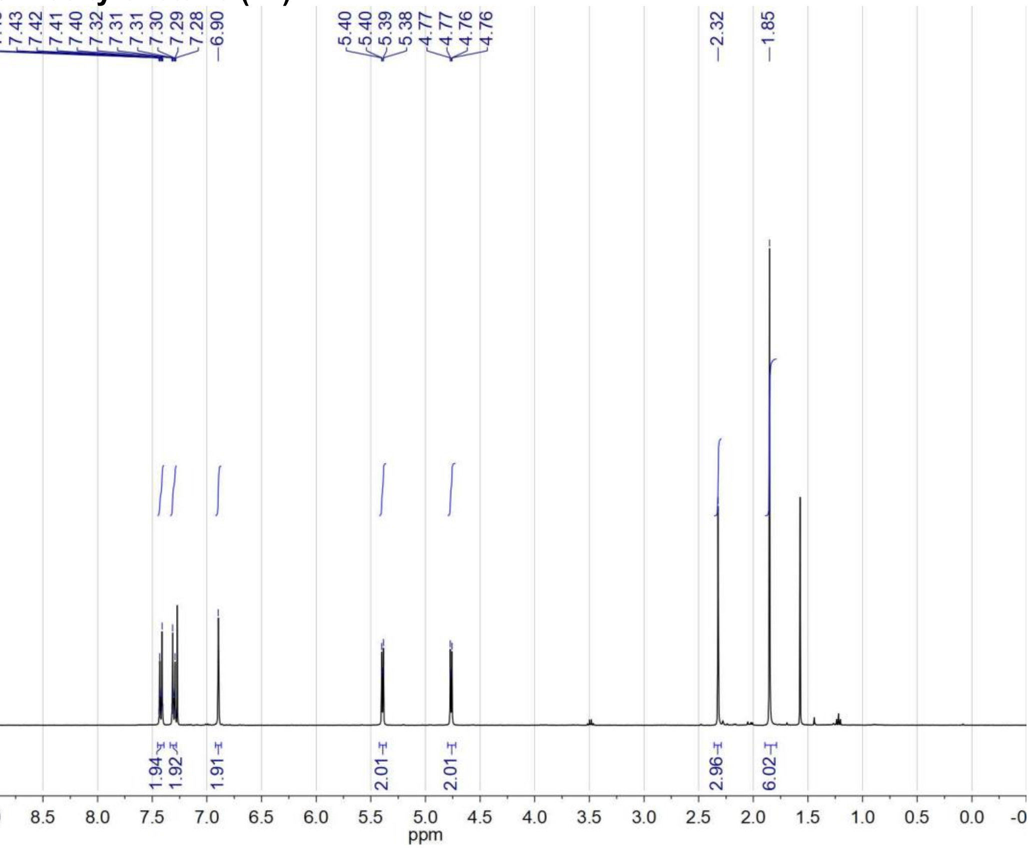

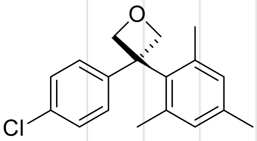

12
^{13}C{^{1}H} NMR
(101 MHz, $CDCl_3$)

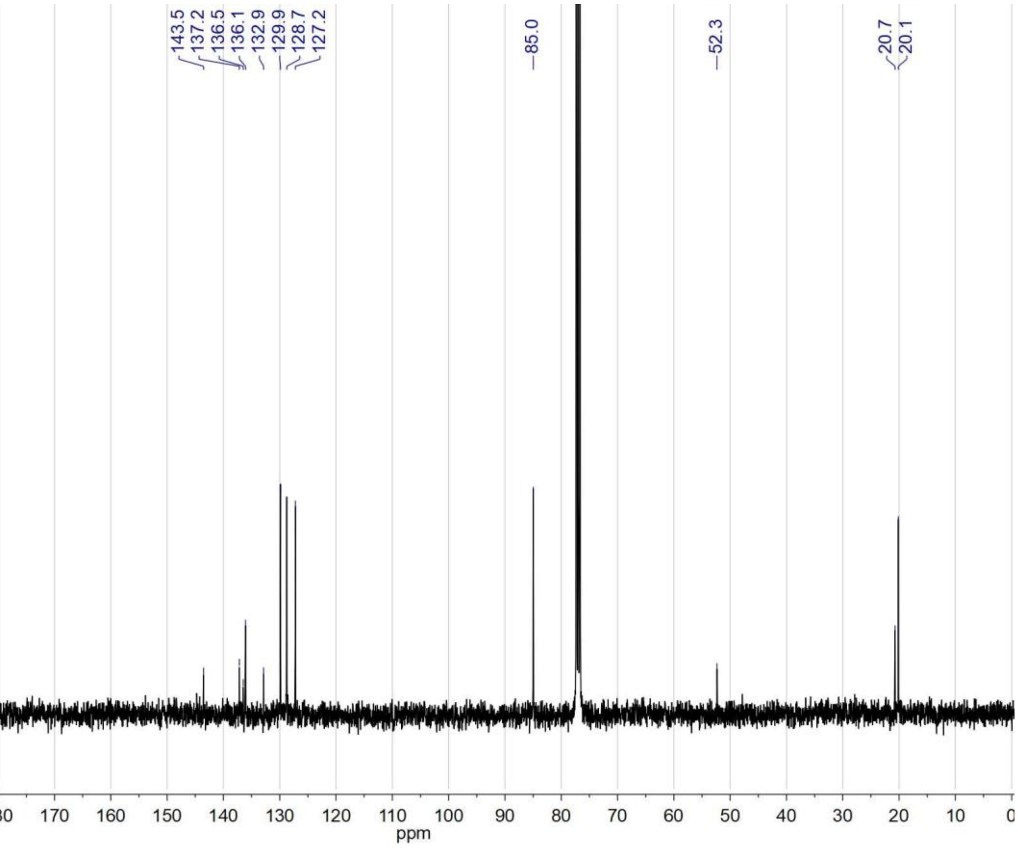

2-(4-Chlorophenyl)-3-mesitylpropanal (12a) and 3-(4-chlorophenyl)-2-mesitylpropanal (12b)

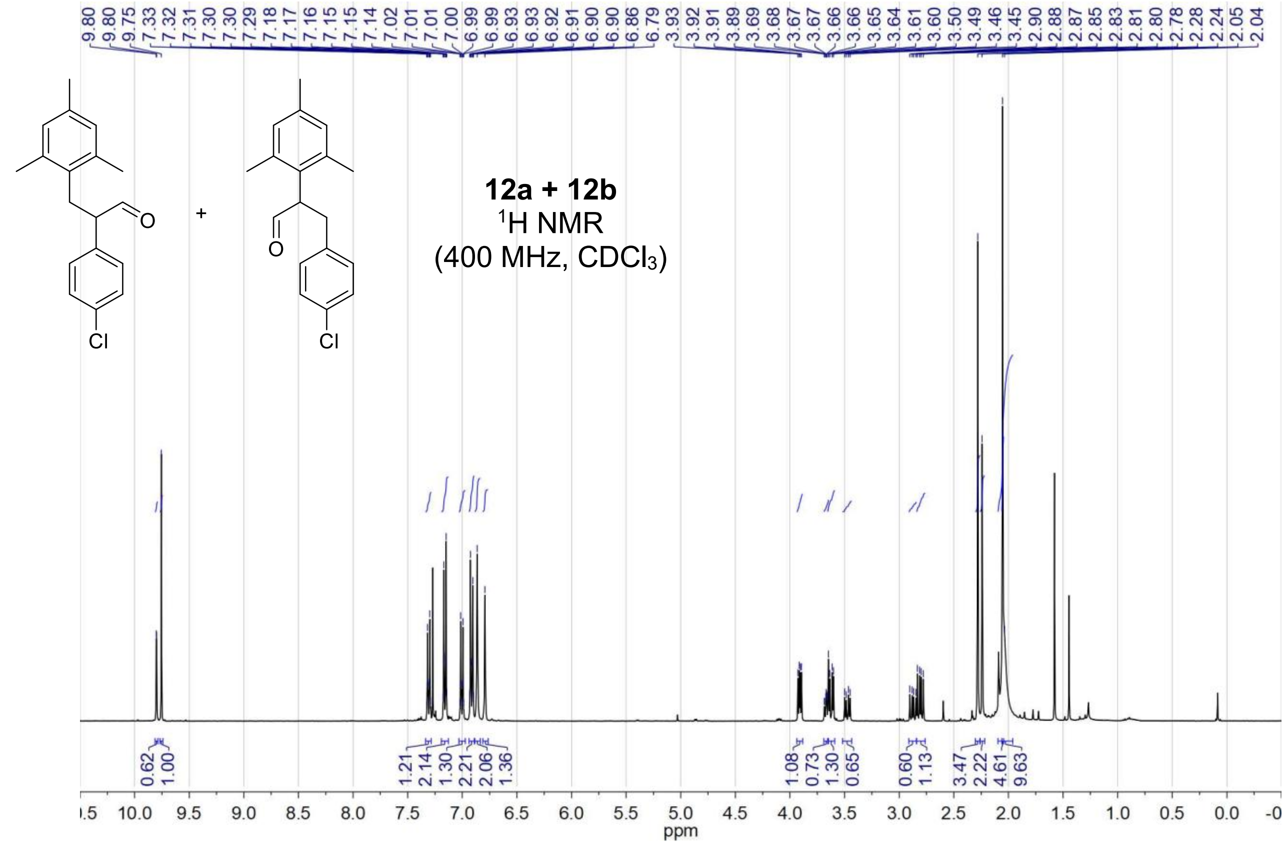

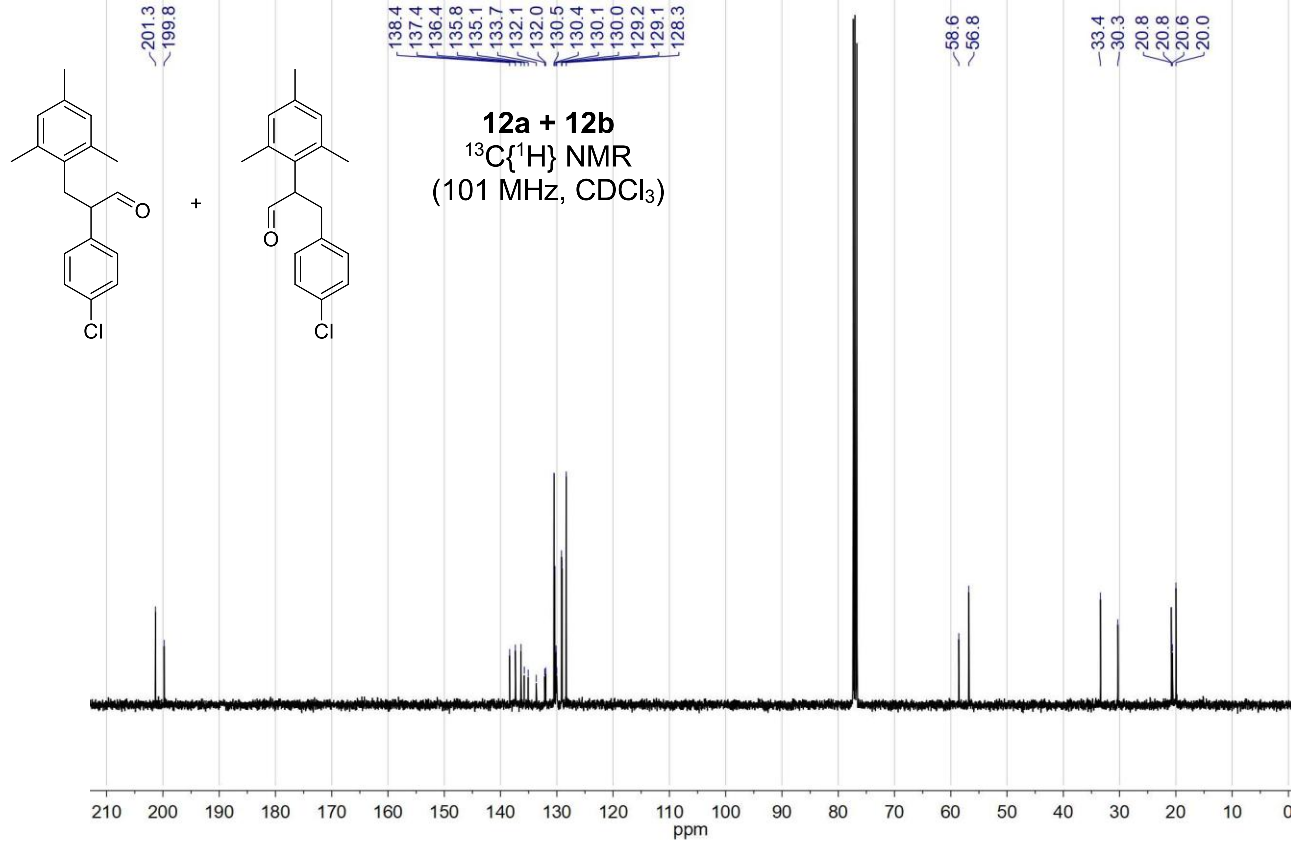

3-(4-Bromophenyl)-3-mesityloxetane (13)

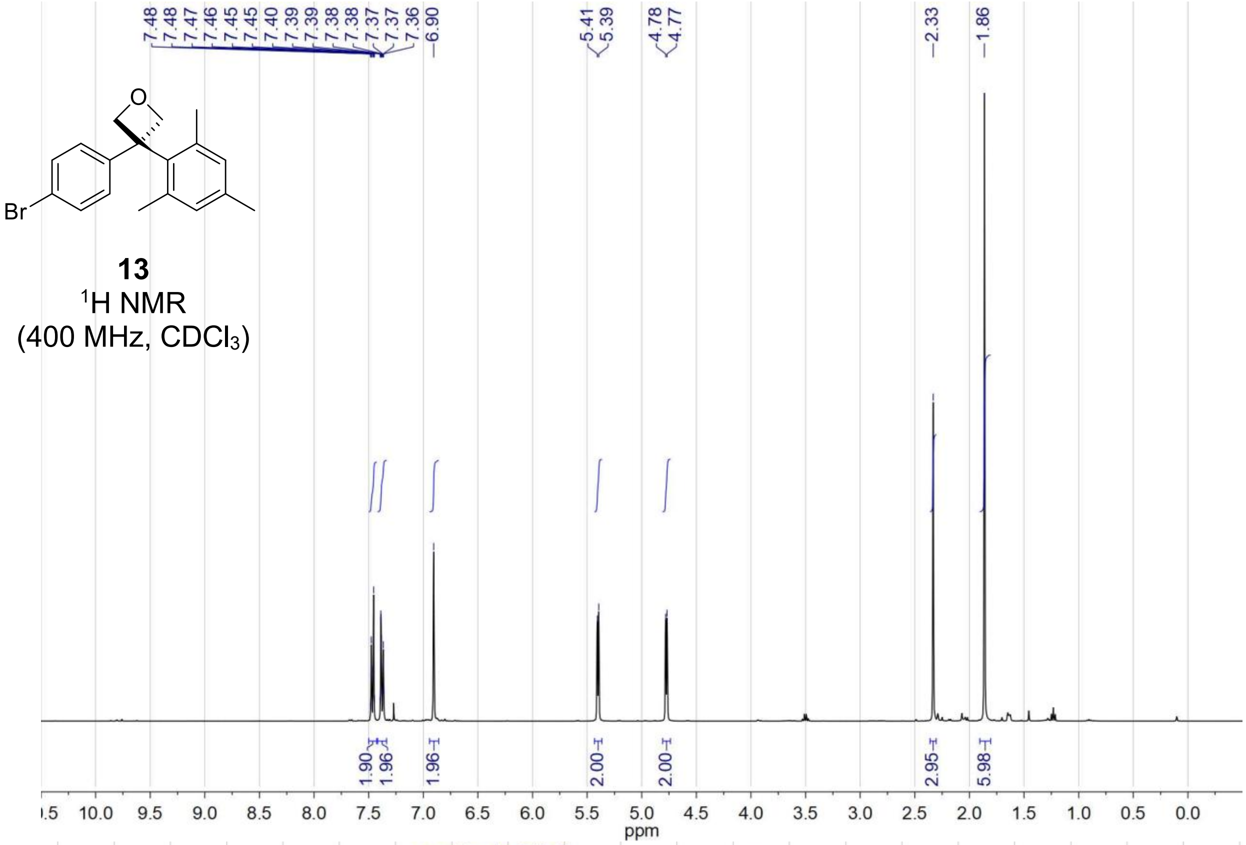

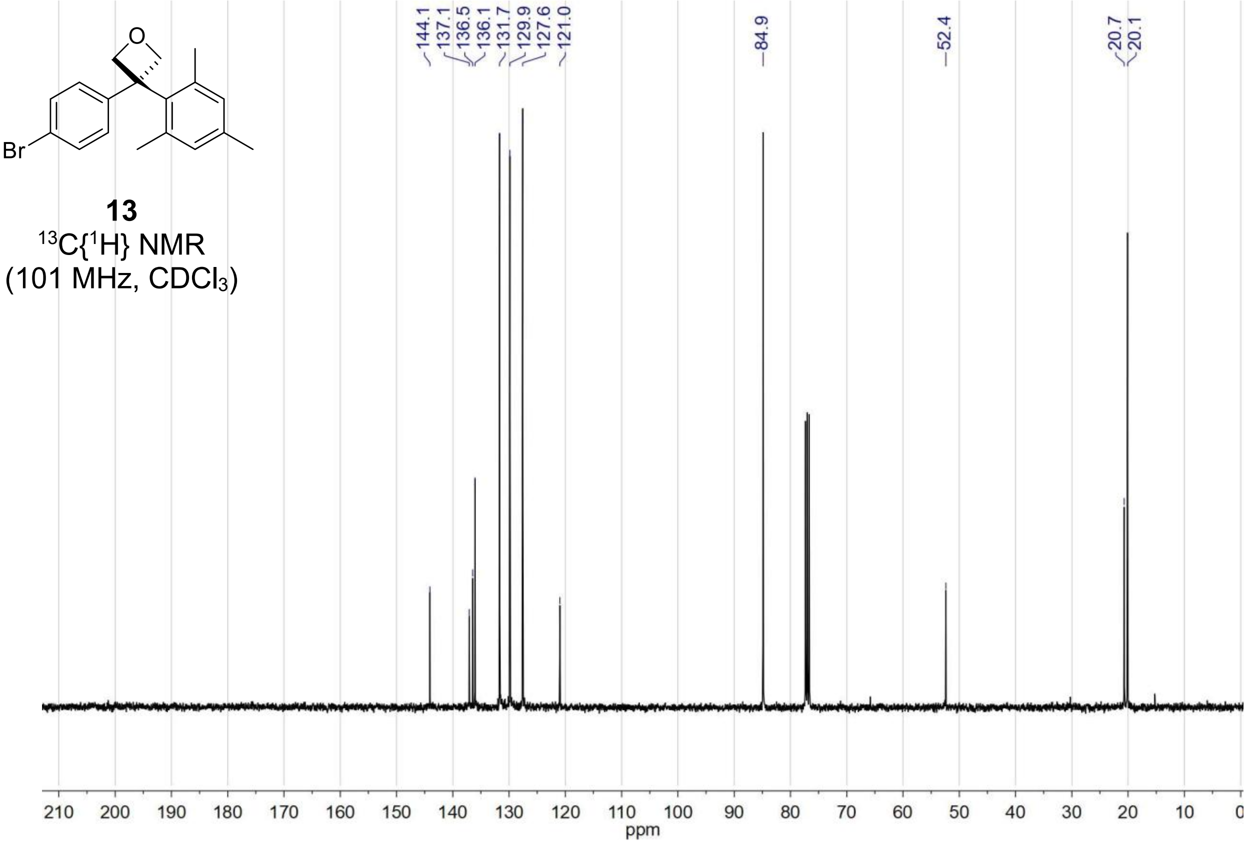

2-(4-Bromophenyl)-3-mesitylpropanal (13a) and 3-(4-bromophenyl)-2-mesitylpropanal (13b)

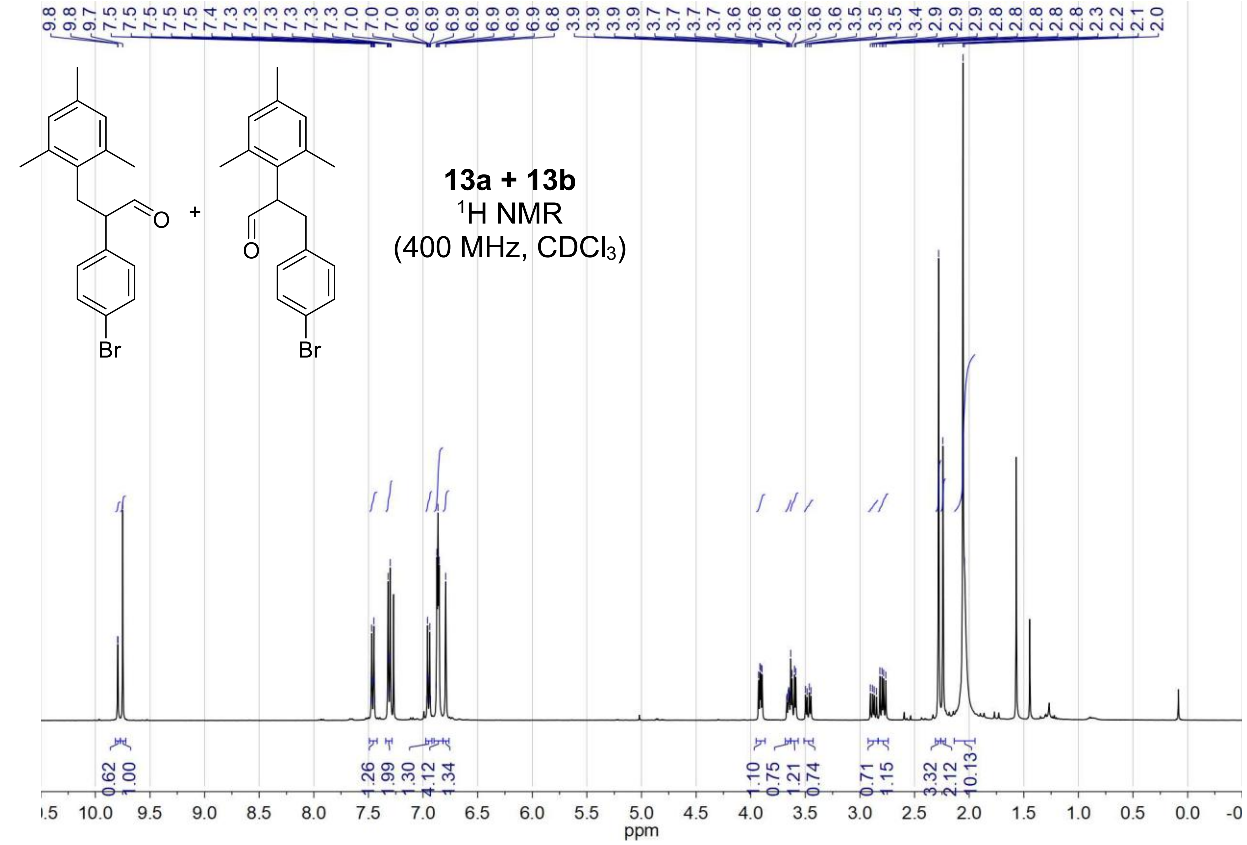

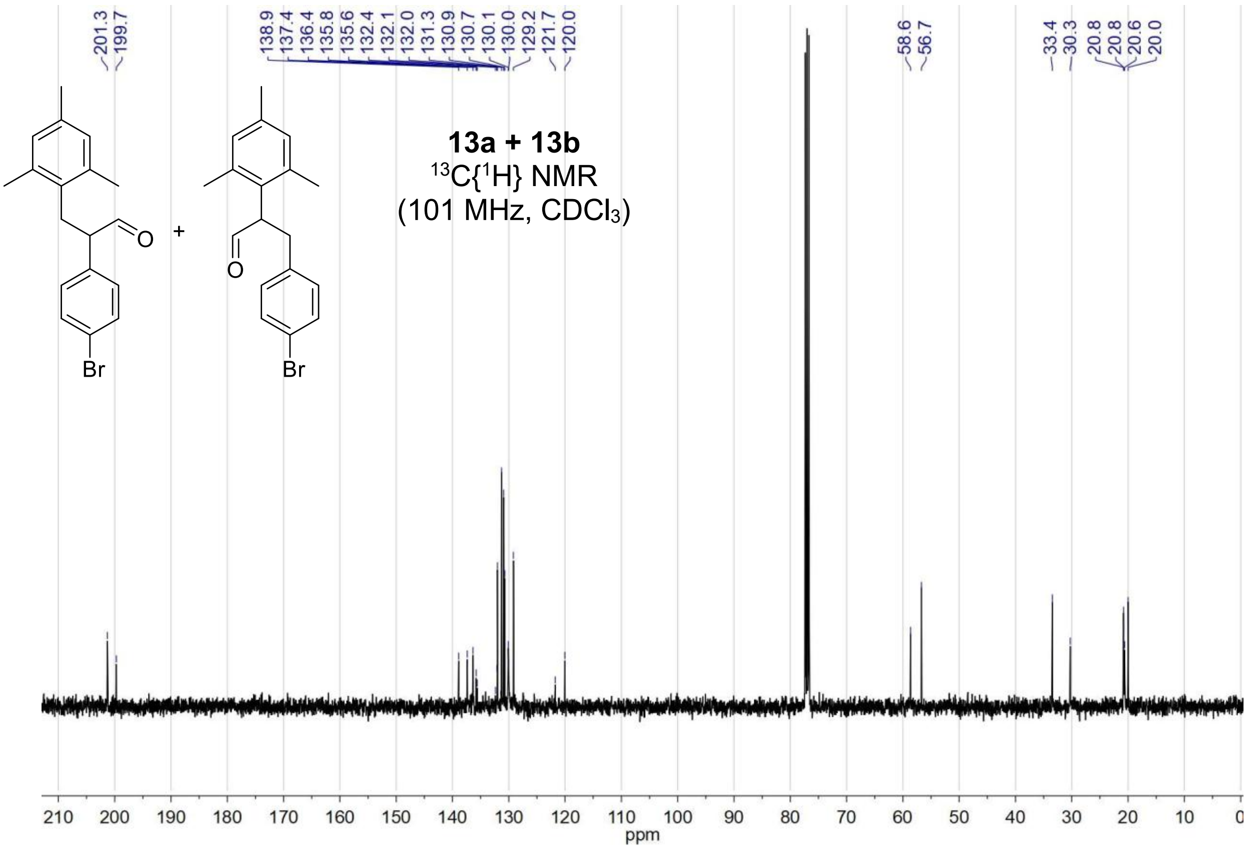

3-(4-Iodophenyl)-3-mesityloxetane (14)

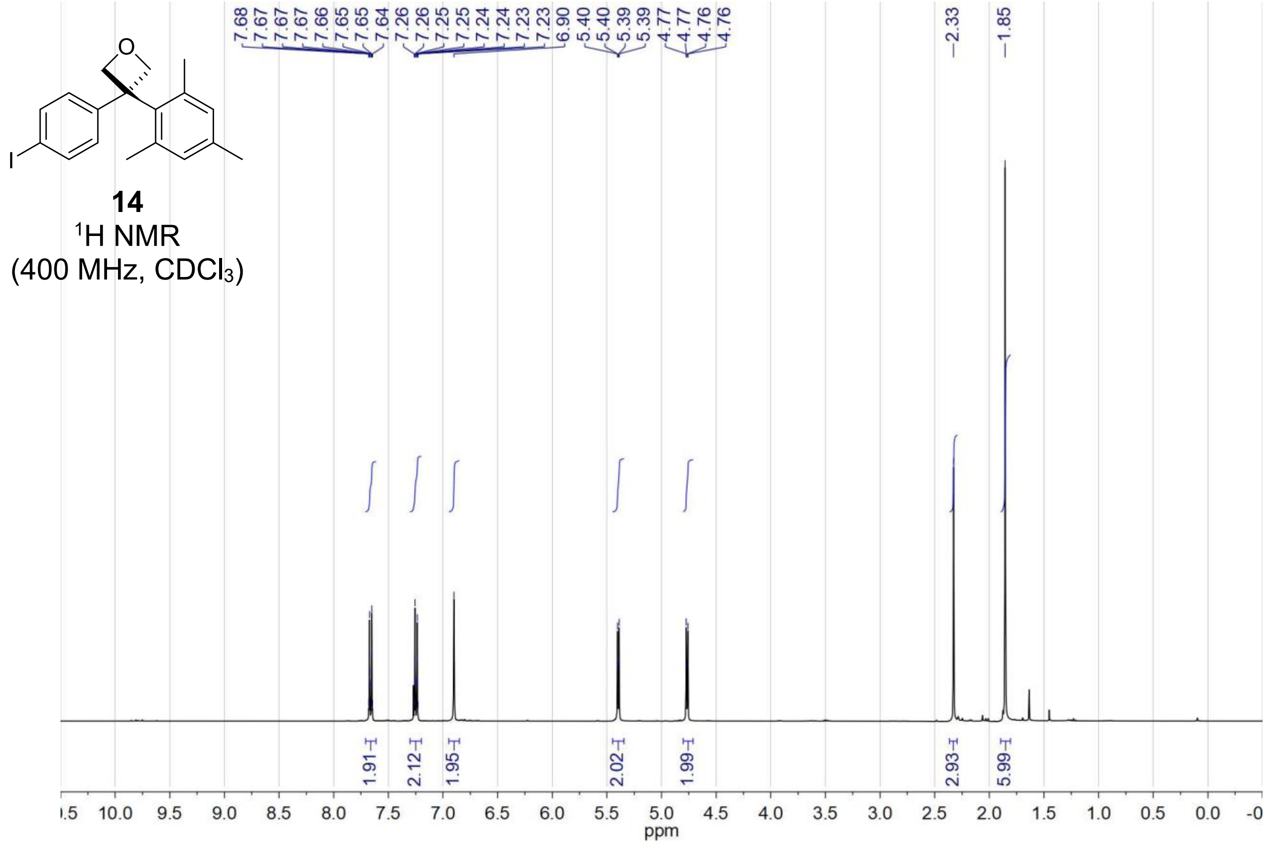

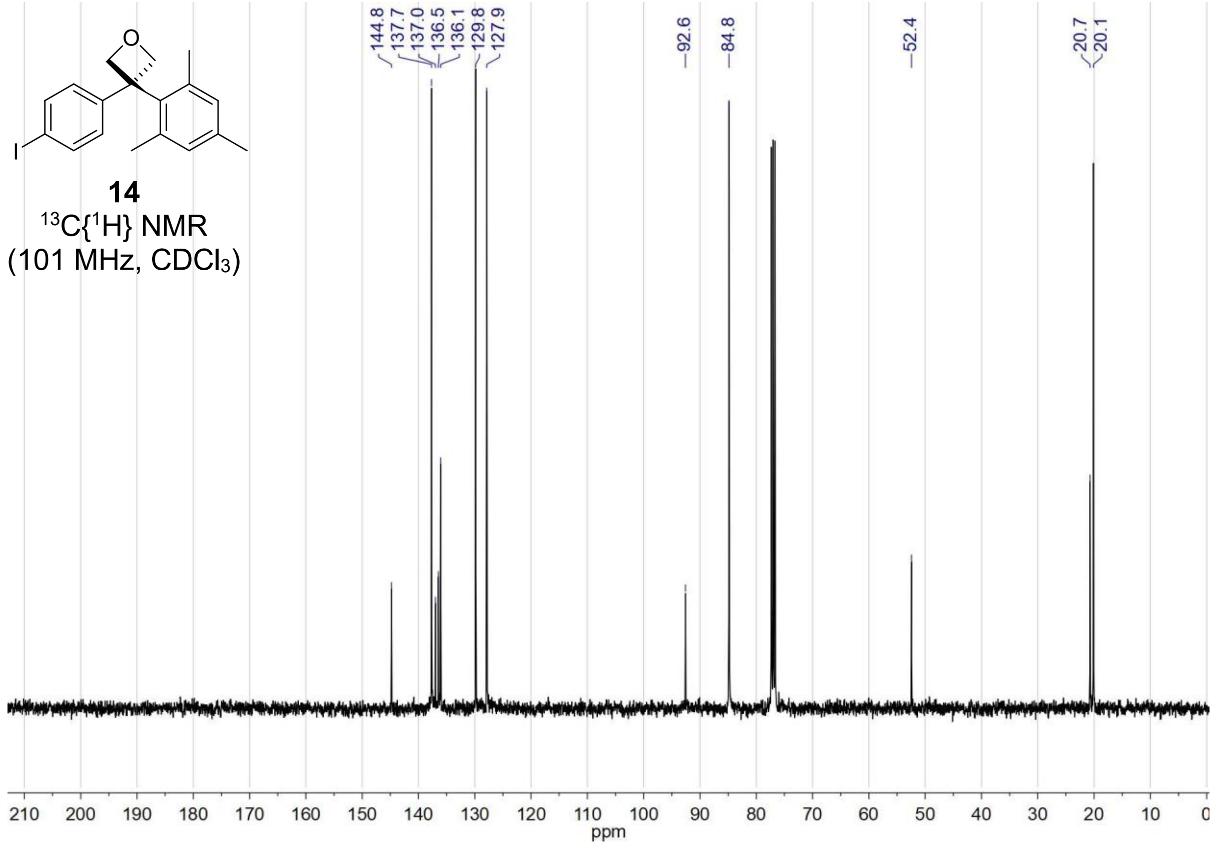

2-(4-Iodophenyl)-3-mesitylpropanal (14a) and 3-(4-iodophenyl)-2-mesitylpropanal (14b)

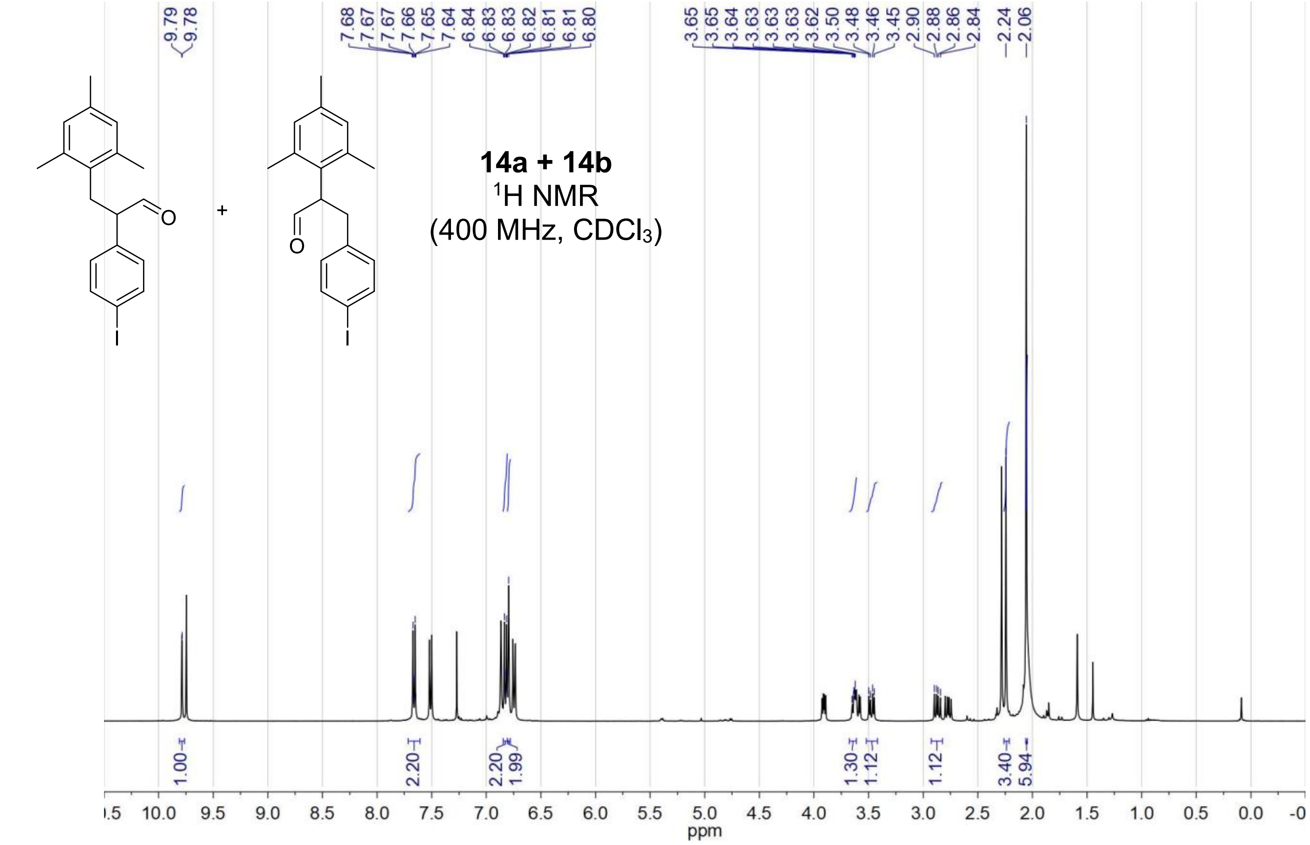

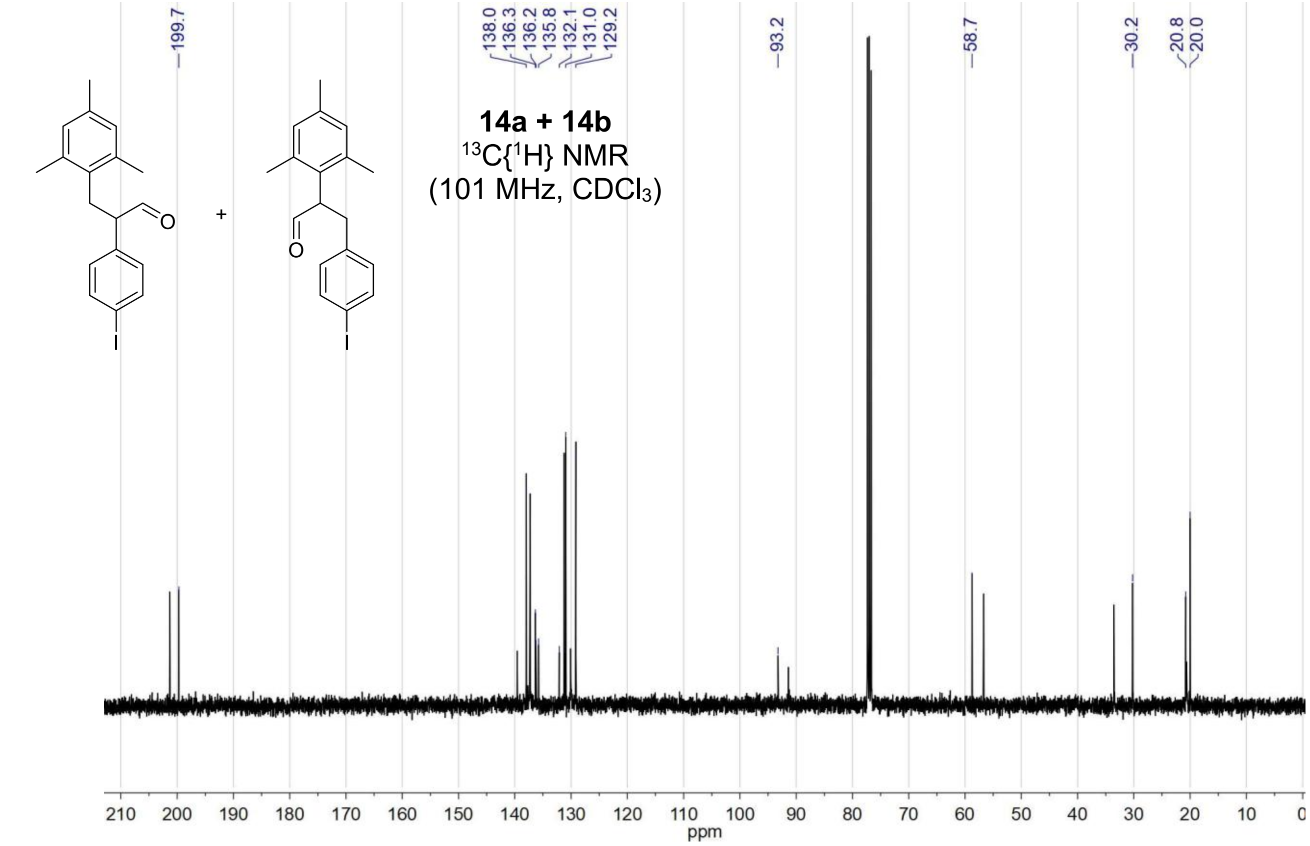

3-Mesityl-3-(4-methoxyphenyl)oxetane (15)

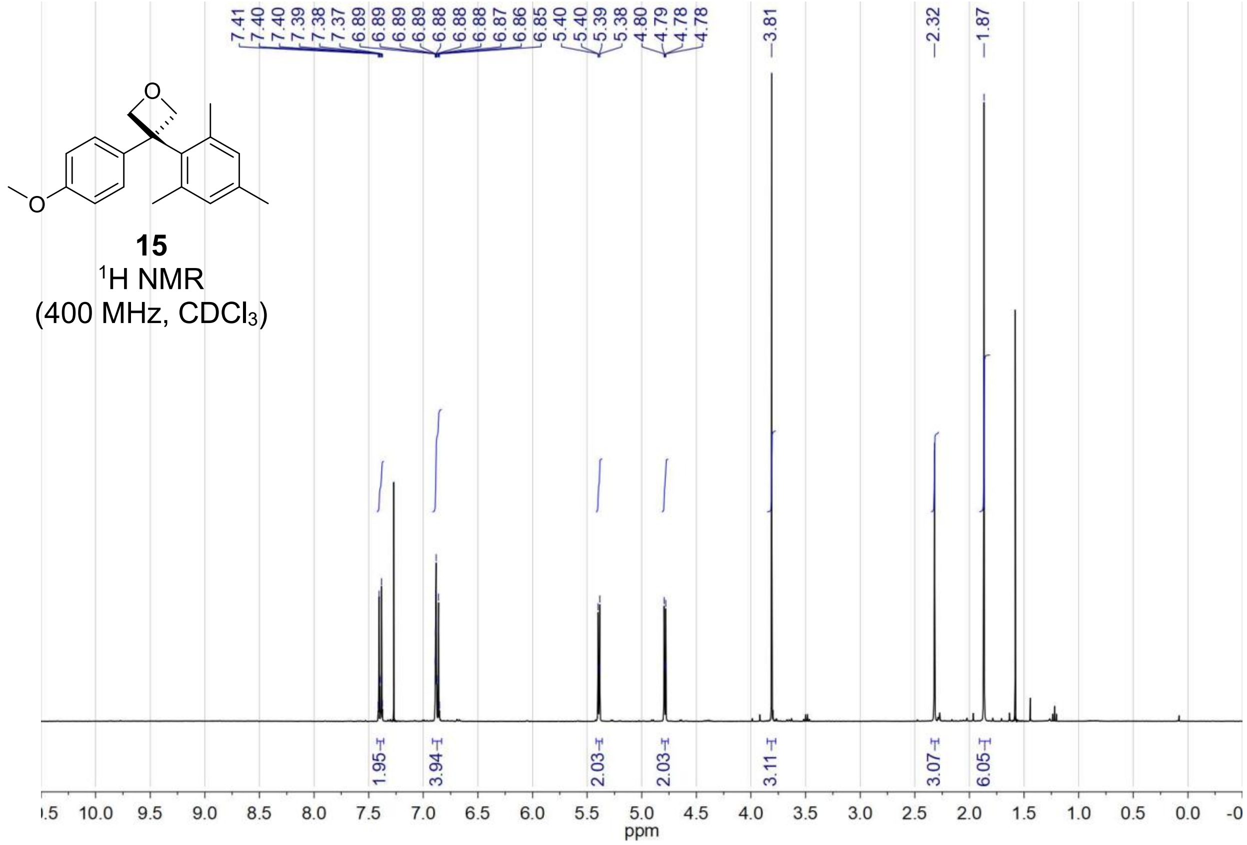

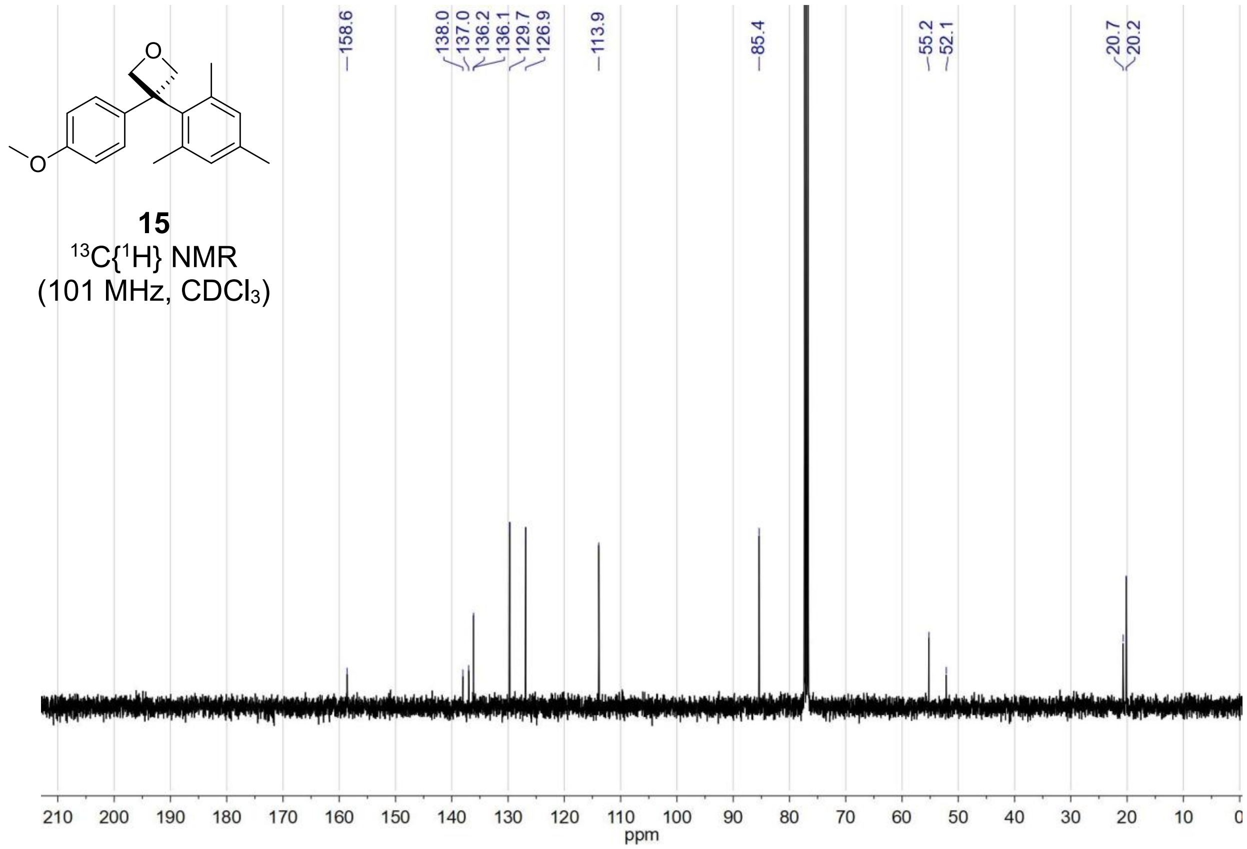

3-Mesityl-2-(4-methoxyphenyl)propanal (15a) and 2-mesityl-3-(4-methoxyphenyl)propanal (15b)

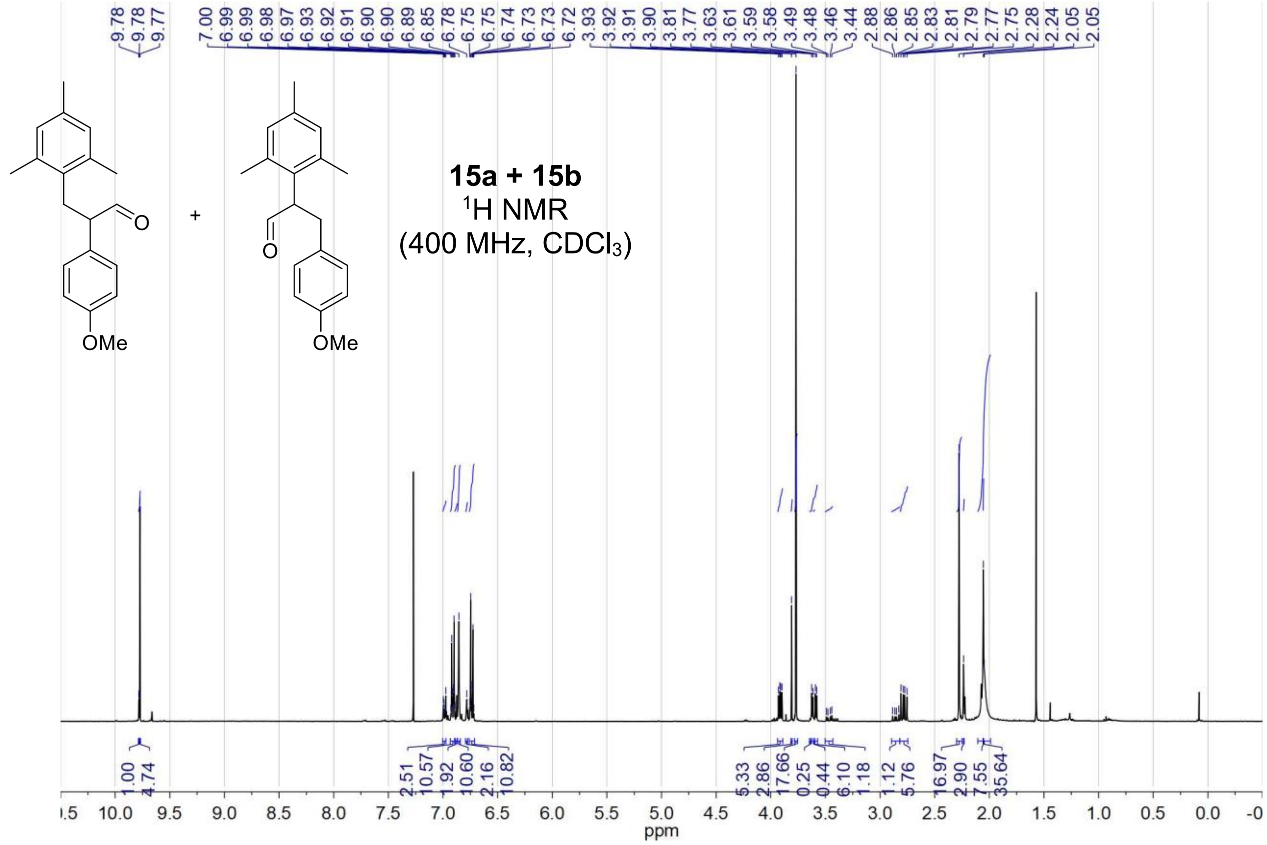

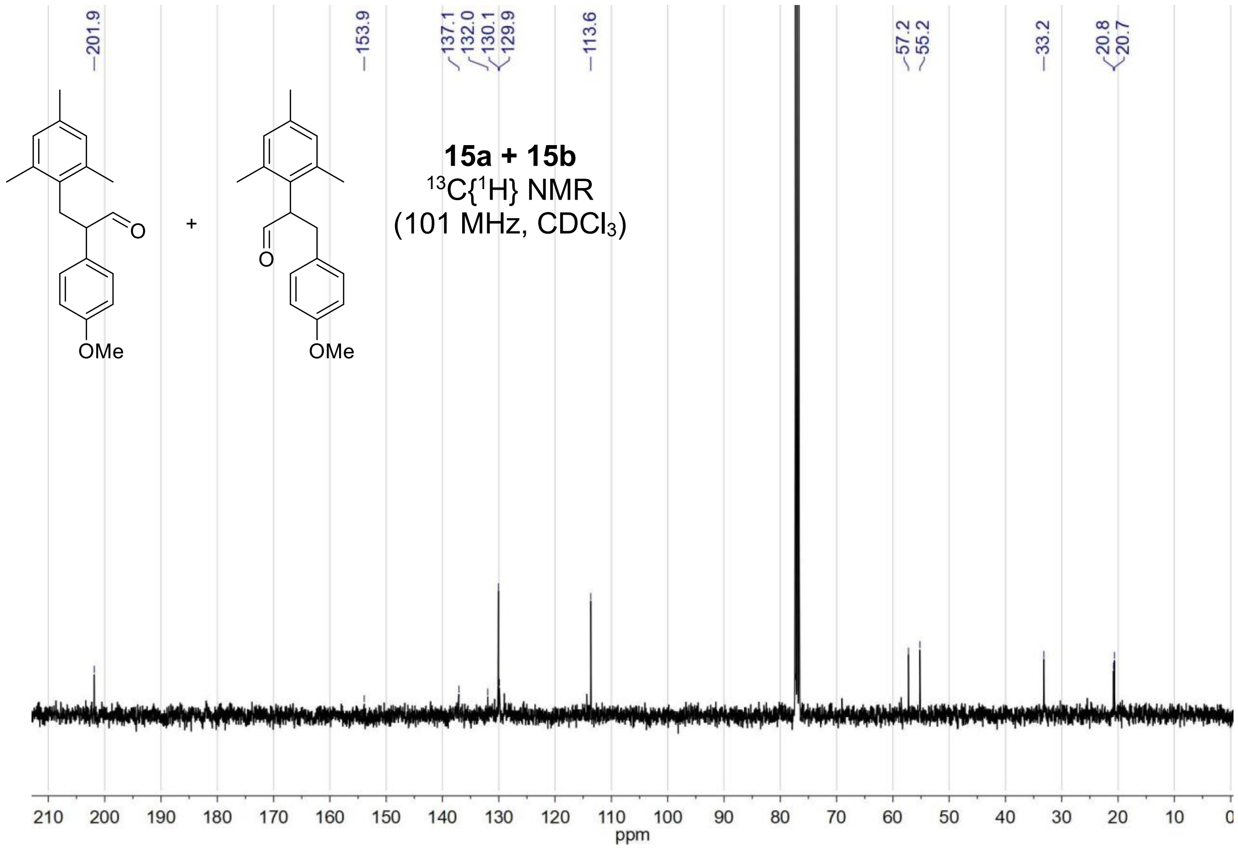

3-Mesityl-3-(3-methoxyphenyl)oxetane (16)

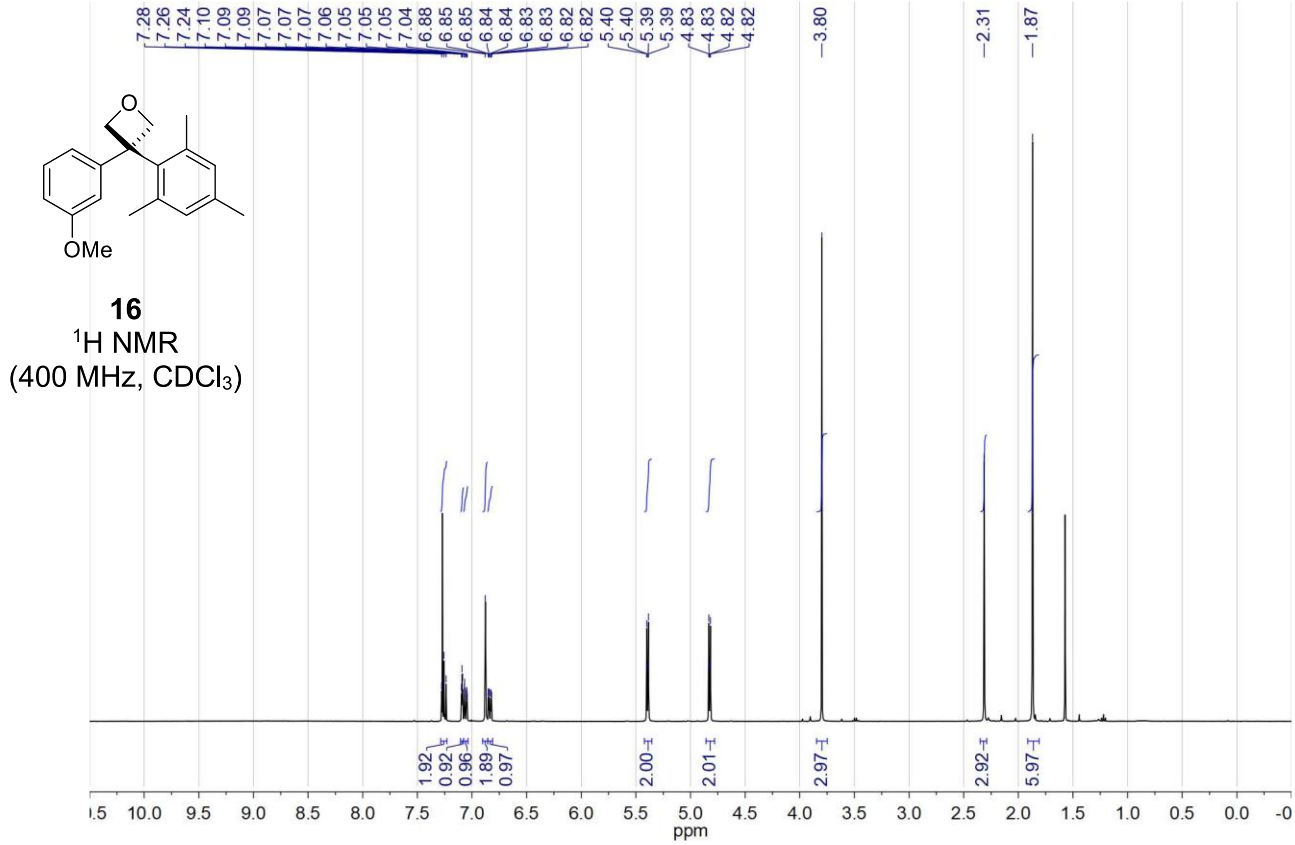

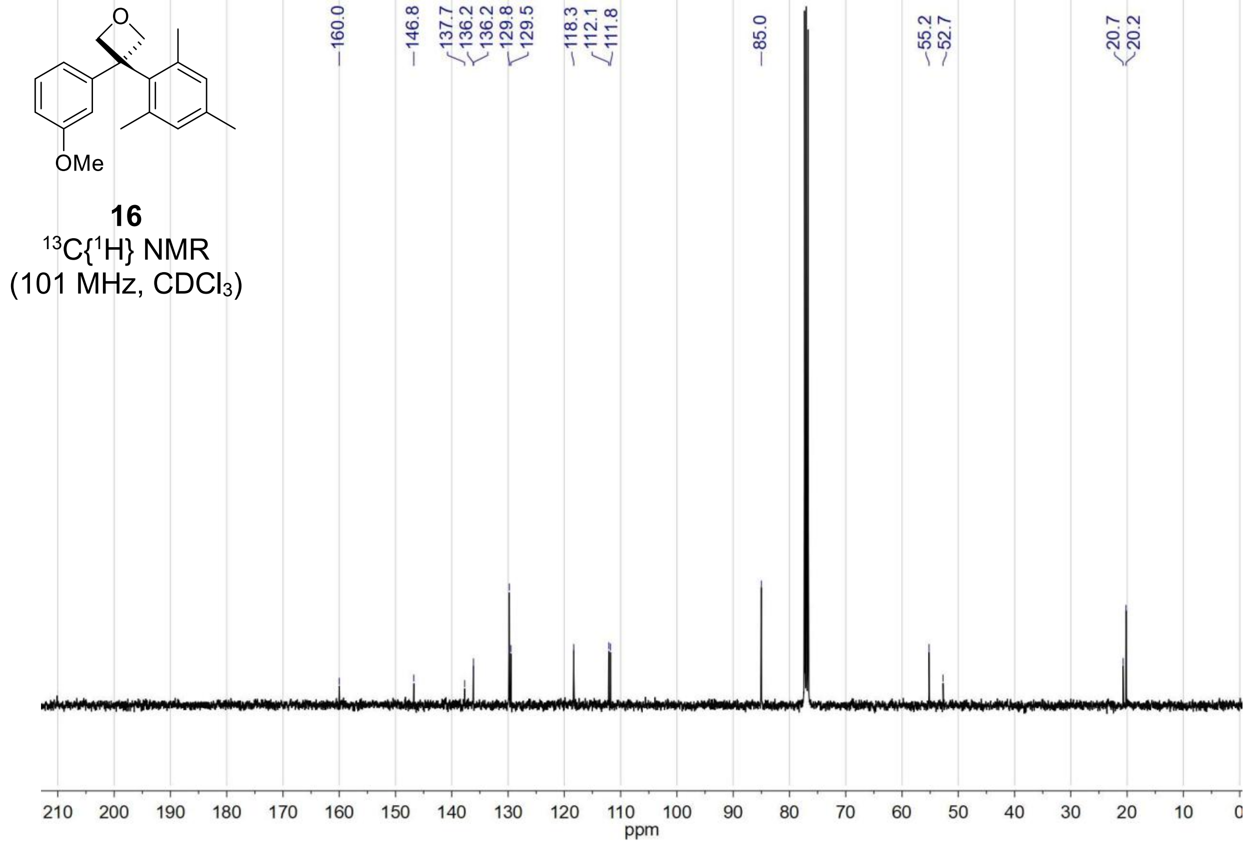

3-(3-Bromophenyl)-3-mesityloxetane (17)

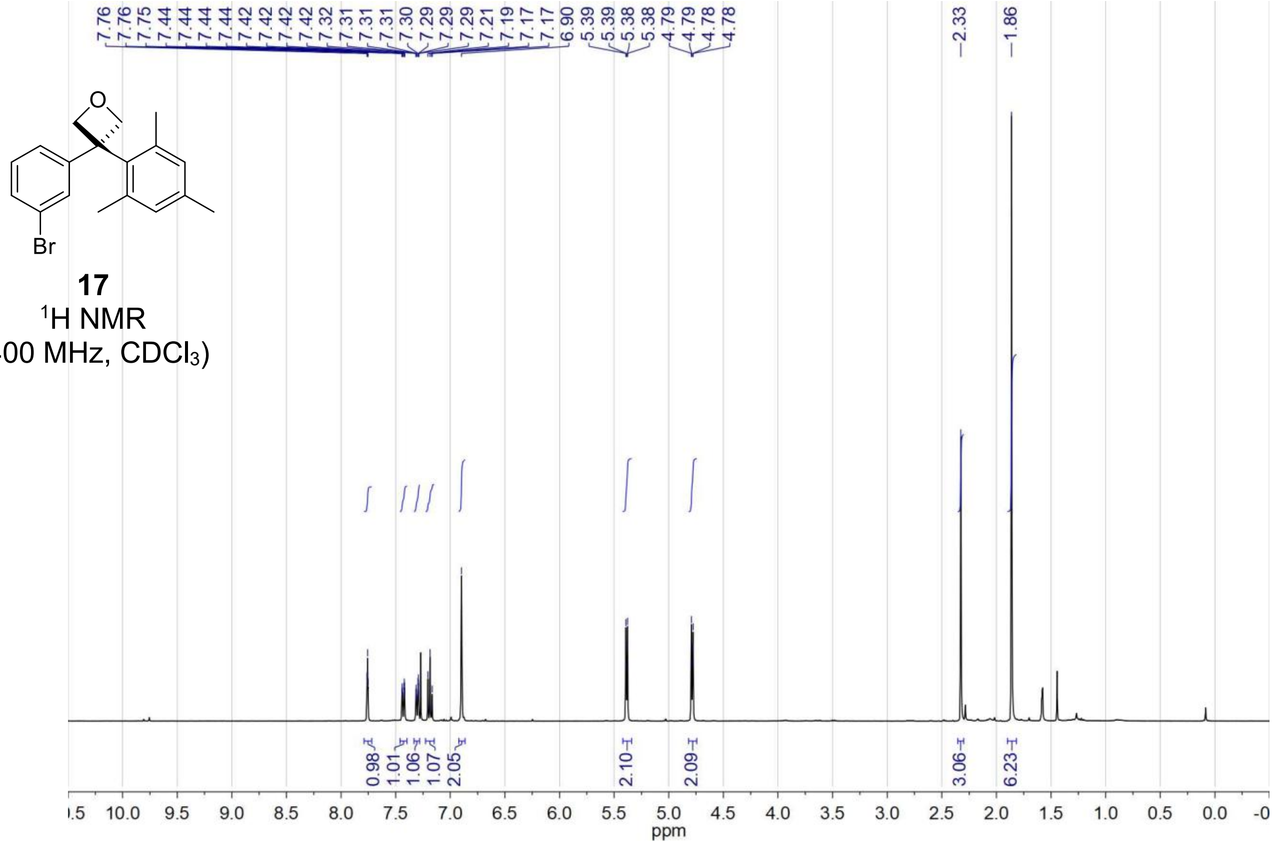

17
^{1}H NMR
(400 MHz, $CDCl_3$)

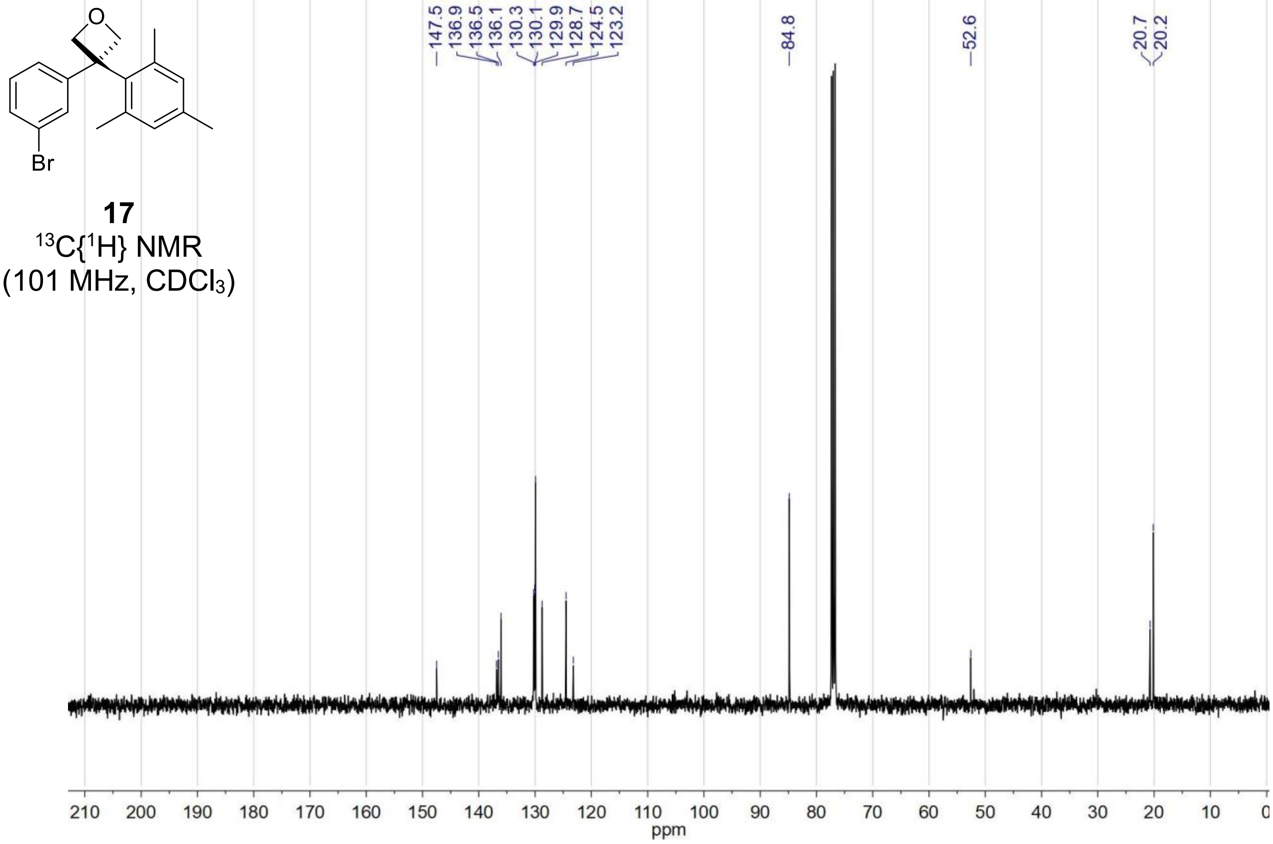

17
$^{13}C\{^1H\}$ NMR
(101 MHz, $CDCl_3$)

2-(2-Bromophenyl)-3-mesitylpropanal (18a)

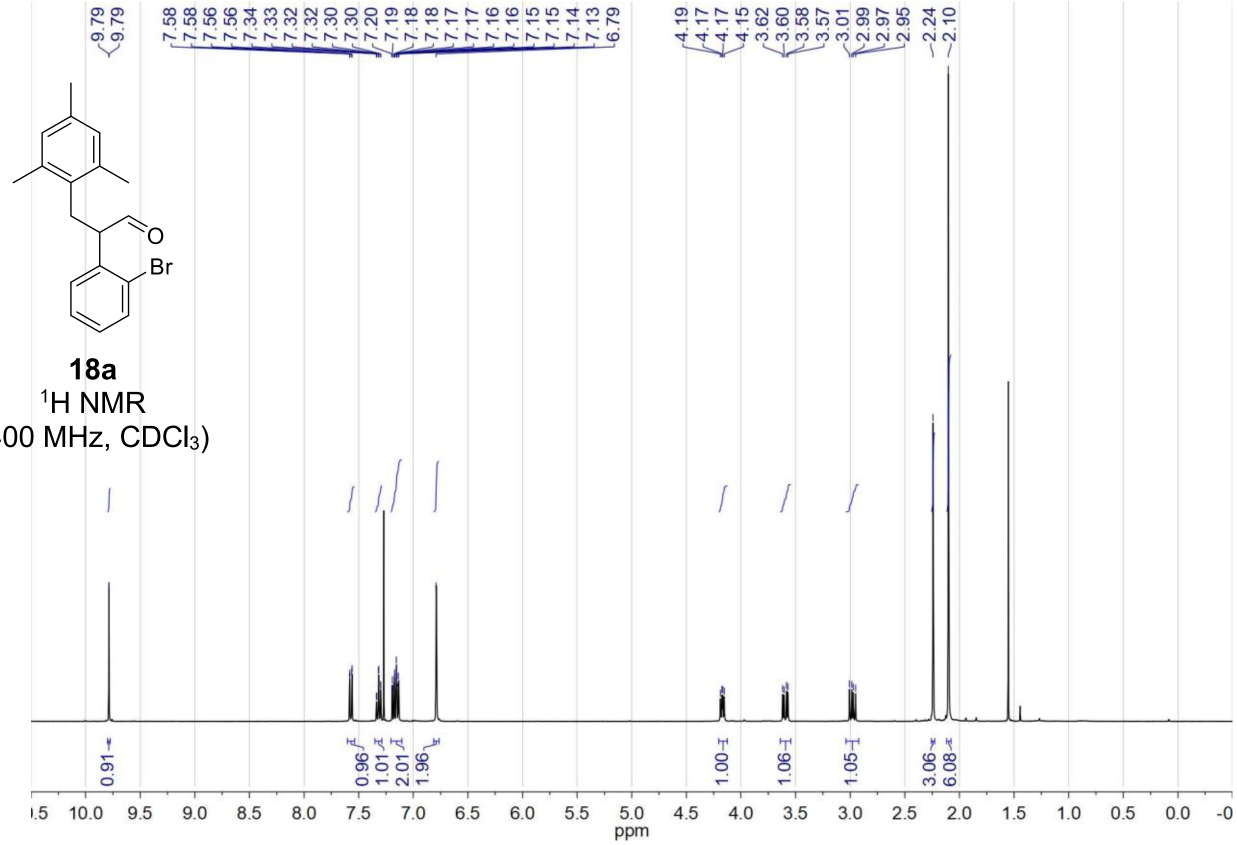

18a
^{1}H NMR
(400 MHz, $CDCl_3$)

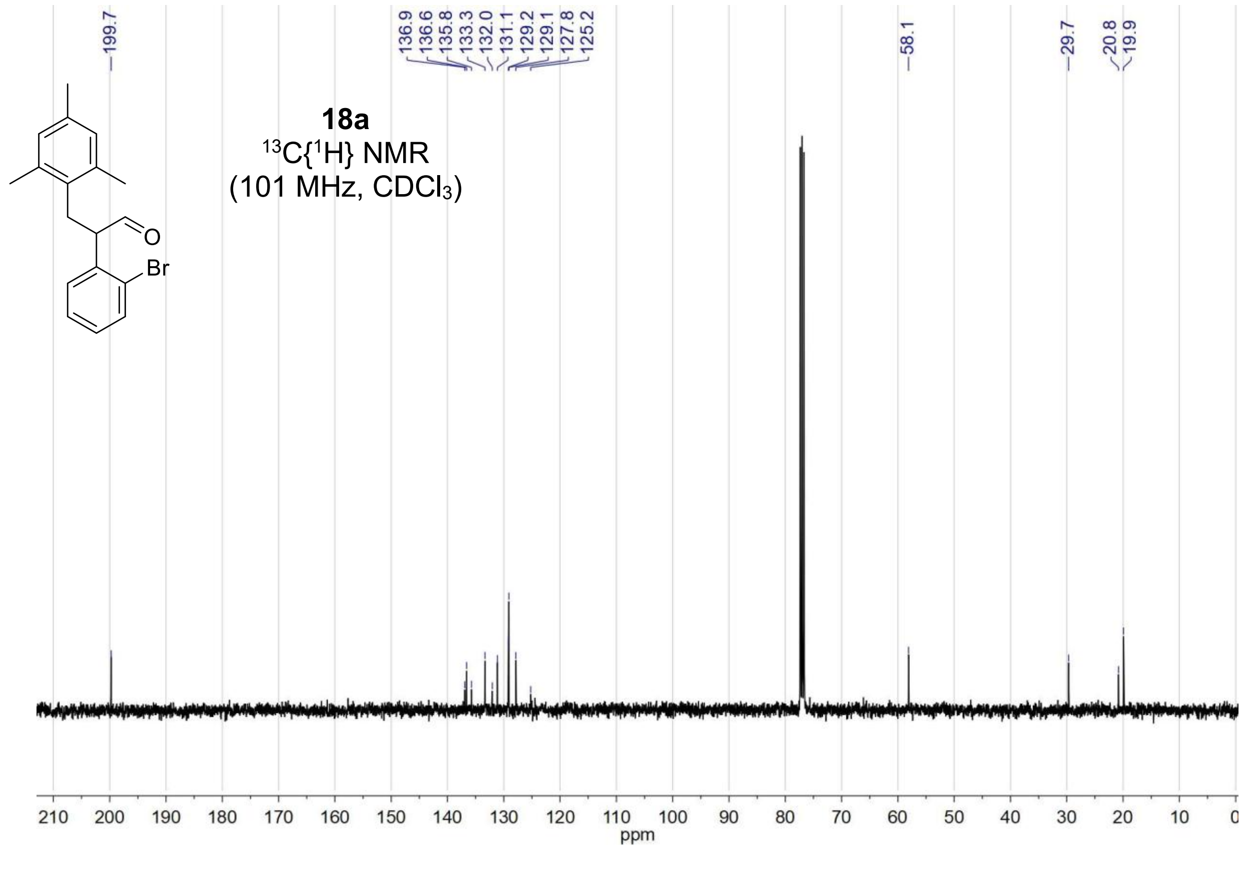

18a
$^{13}C\{^{1}H\}$ NMR
(101 MHz, $CDCl_3$)

3-Mesityl-3-(4-(trifluoromethyl)phenyl)oxetane (19)

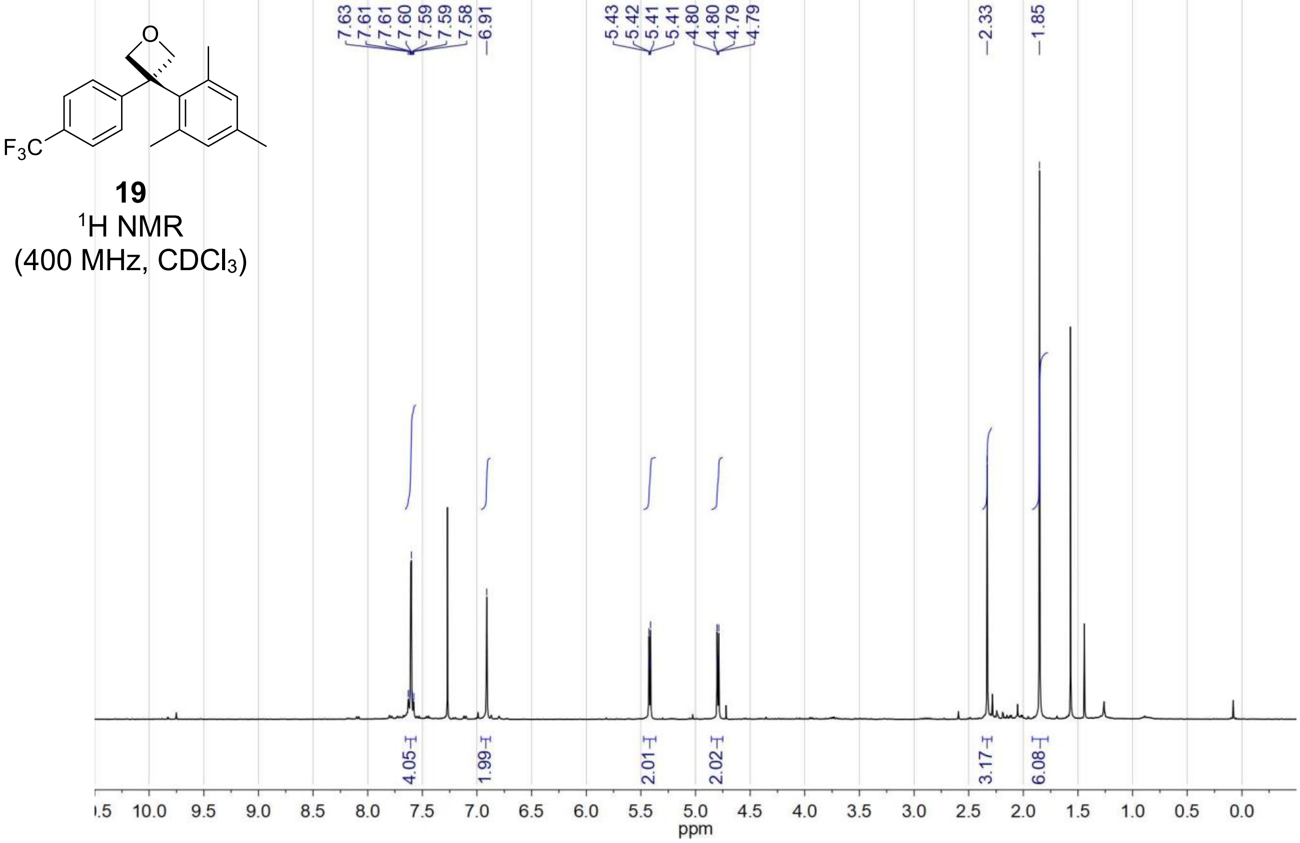

19
^{1}H NMR
(400 MHz, $CDCl_3$)

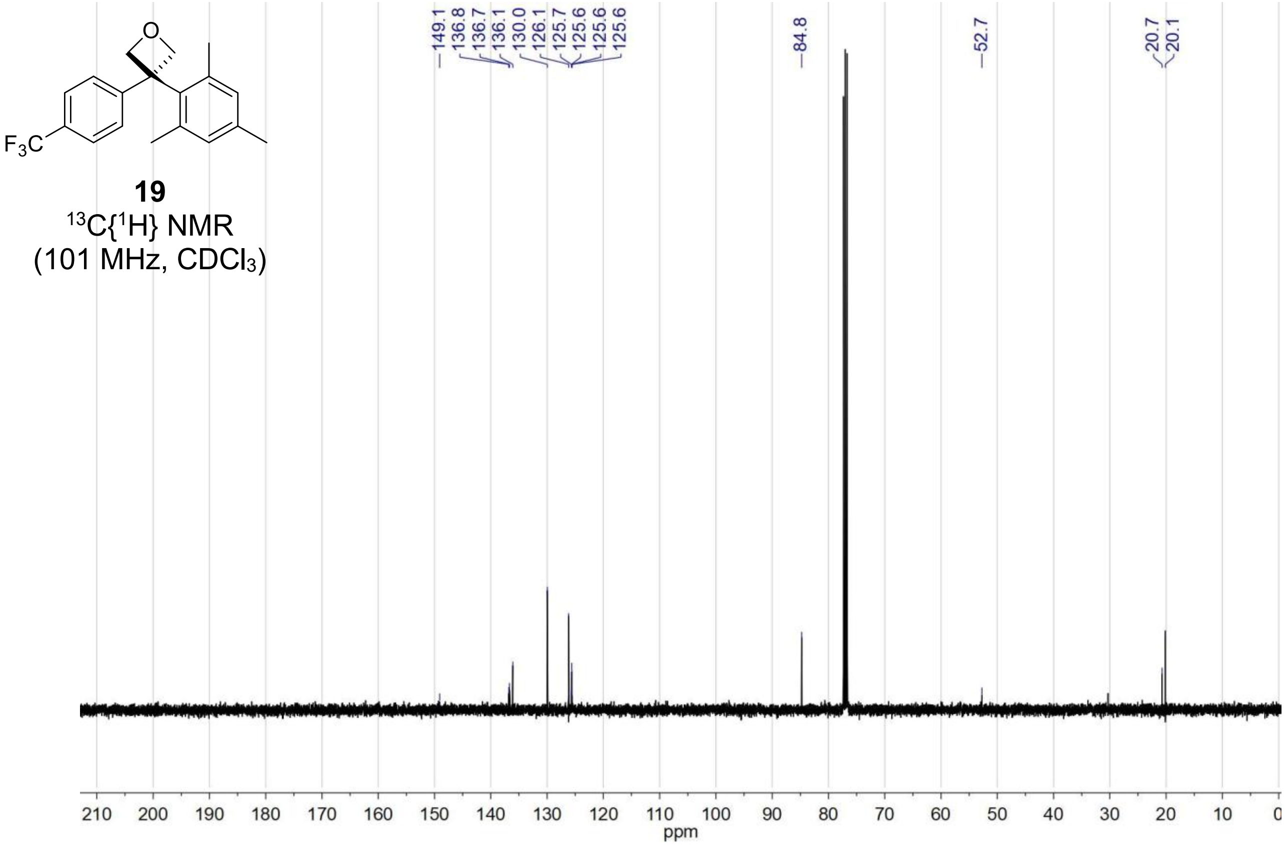

19
$^{13}C\{^{1}H\}$ NMR
(101 MHz, $CDCl_3$)

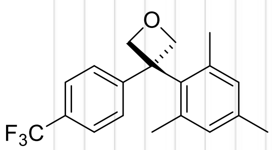

19

^{19}F NMR

(377 MHz, $CDCl_3$)

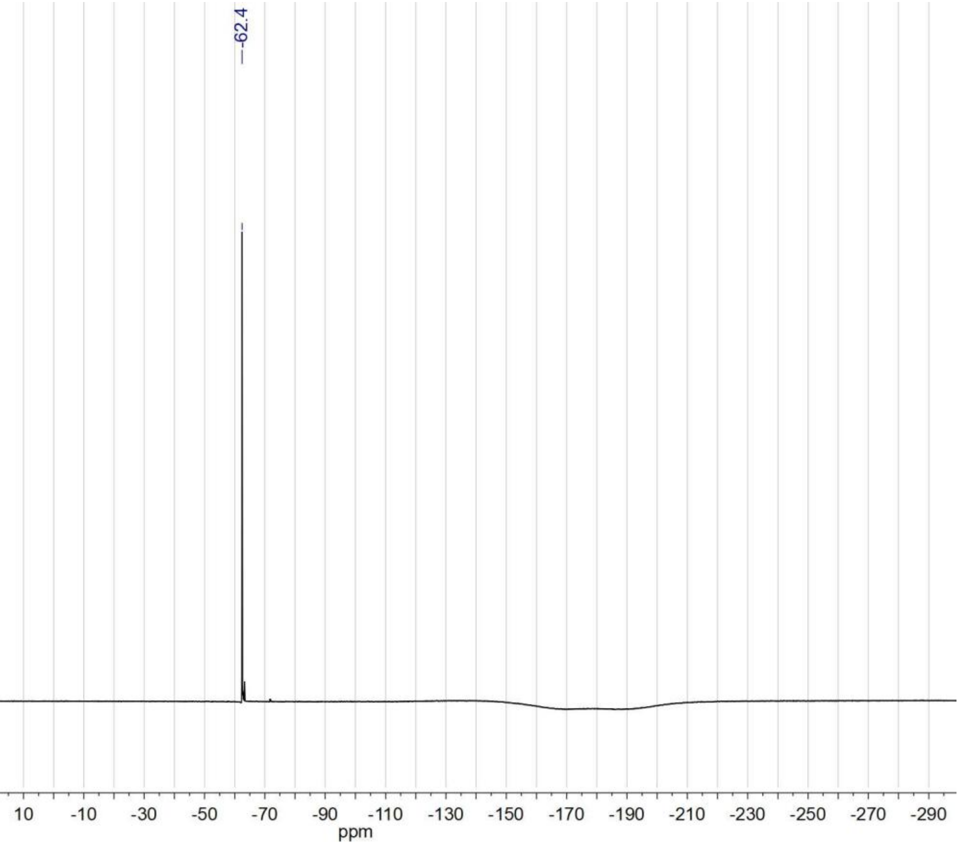

3-Mesityl-3-(3-(trifluoromethyl)phenyl)oxetane (20)

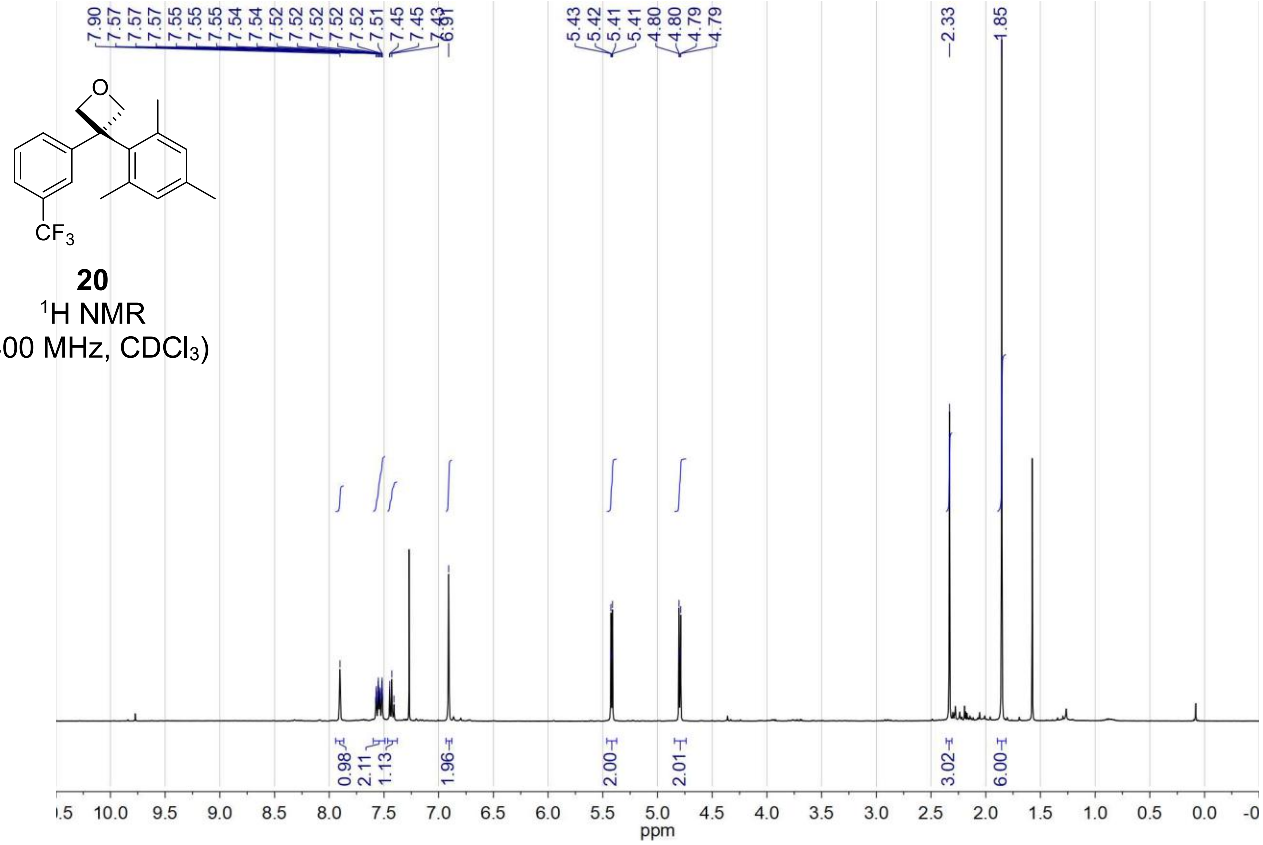

20
^{1}H NMR
(400 MHz, $CDCl_3$)

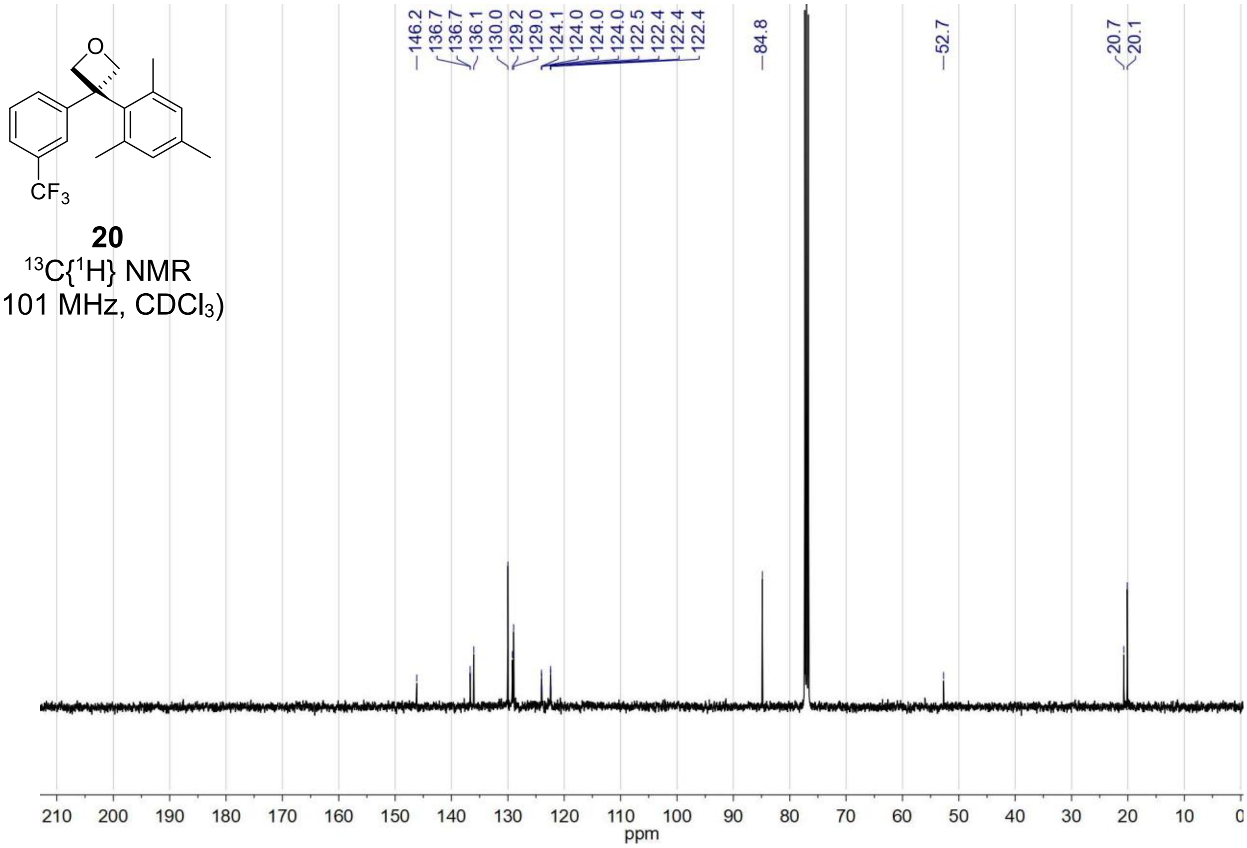

20
$^{13}C\{^1H\}$ NMR
(101 MHz, $CDCl_3$)

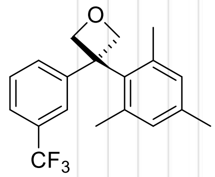

20

^{19}F NMR

(377 MHz, $CDCl_3$)

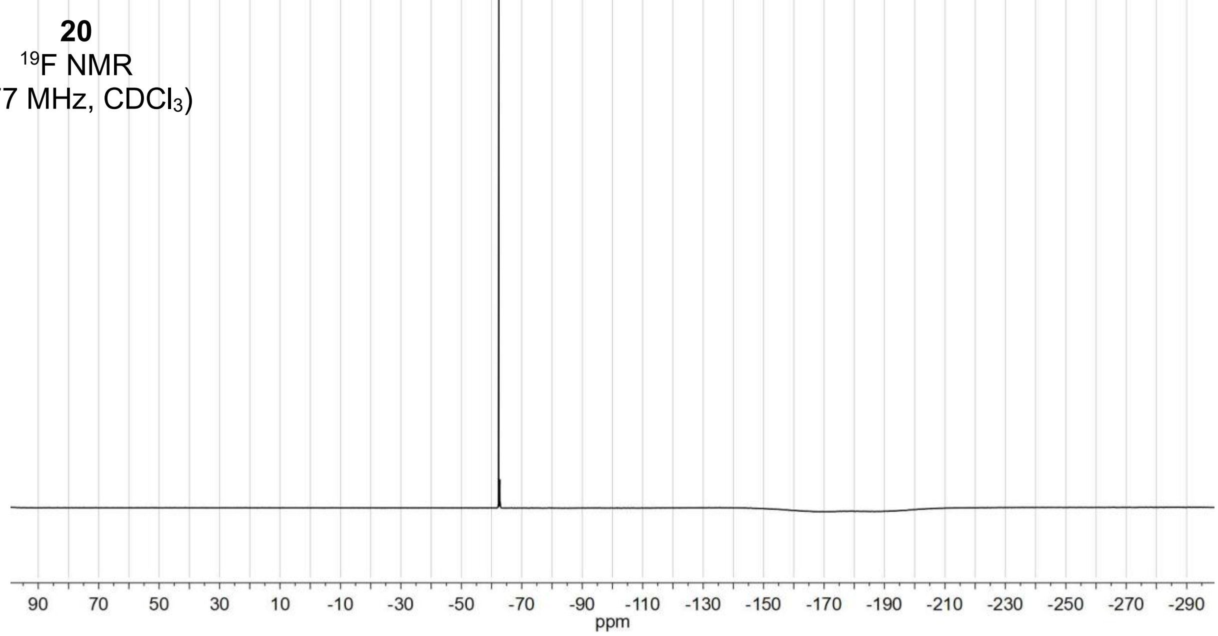

4-(3-(4-Bromophenyl)oxetan-3-yl)-2,6-dimethylphenol (21)

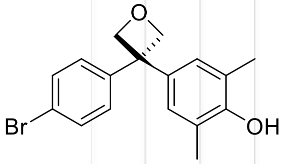

21
^{1}H NMR
(400 MHz, $CDCl_3$)

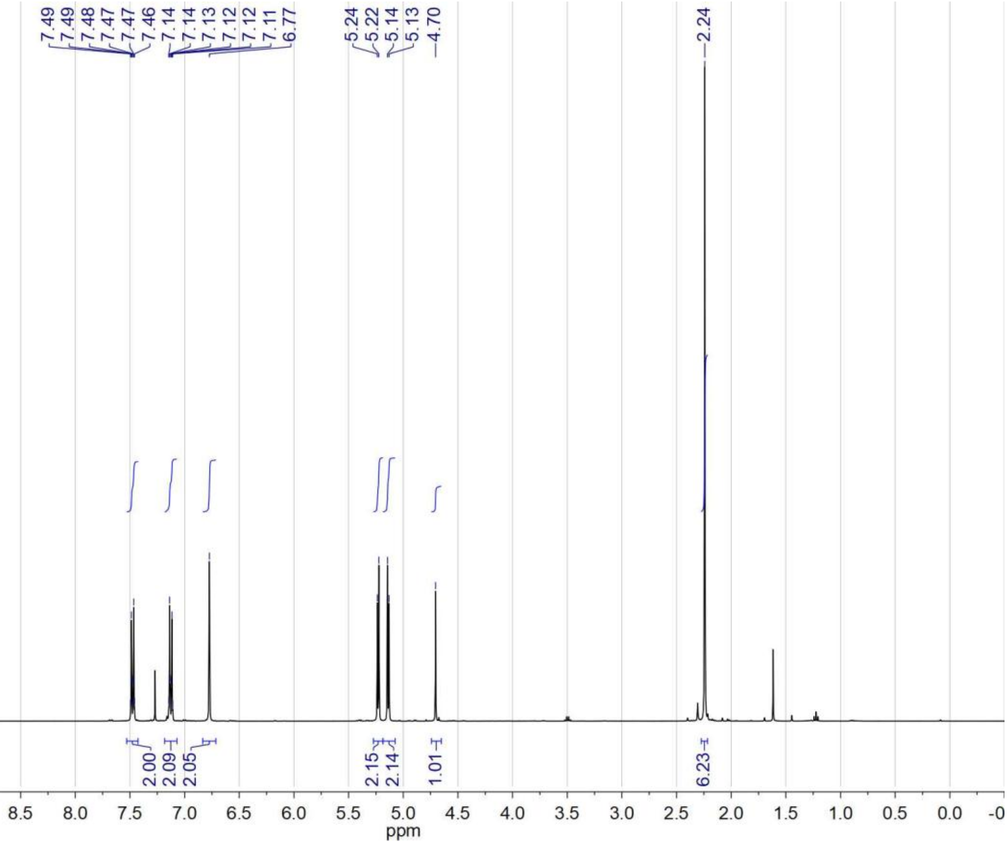

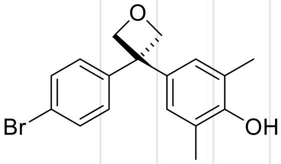

21
$^{13}C\{^{1}H\}$ NMR
(101 MHz, $CDCl_3$)

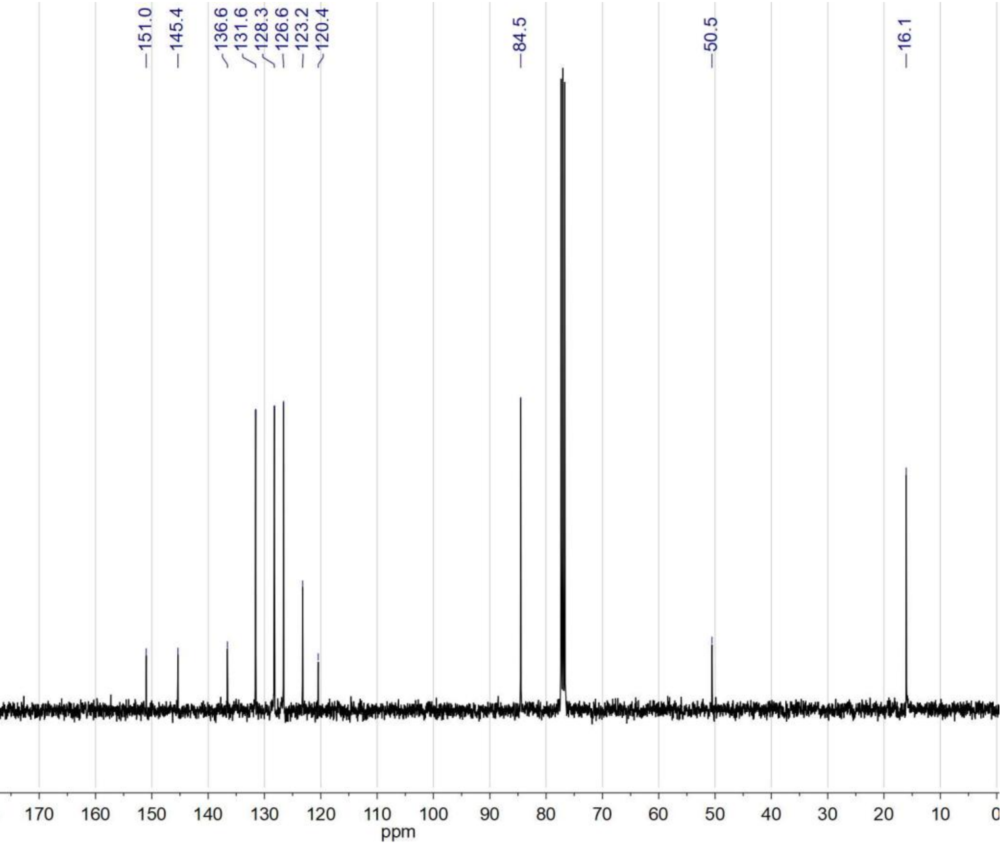

3-([1,1'-Biphenyl]-4-yl)-3-mesityloxetane (22)

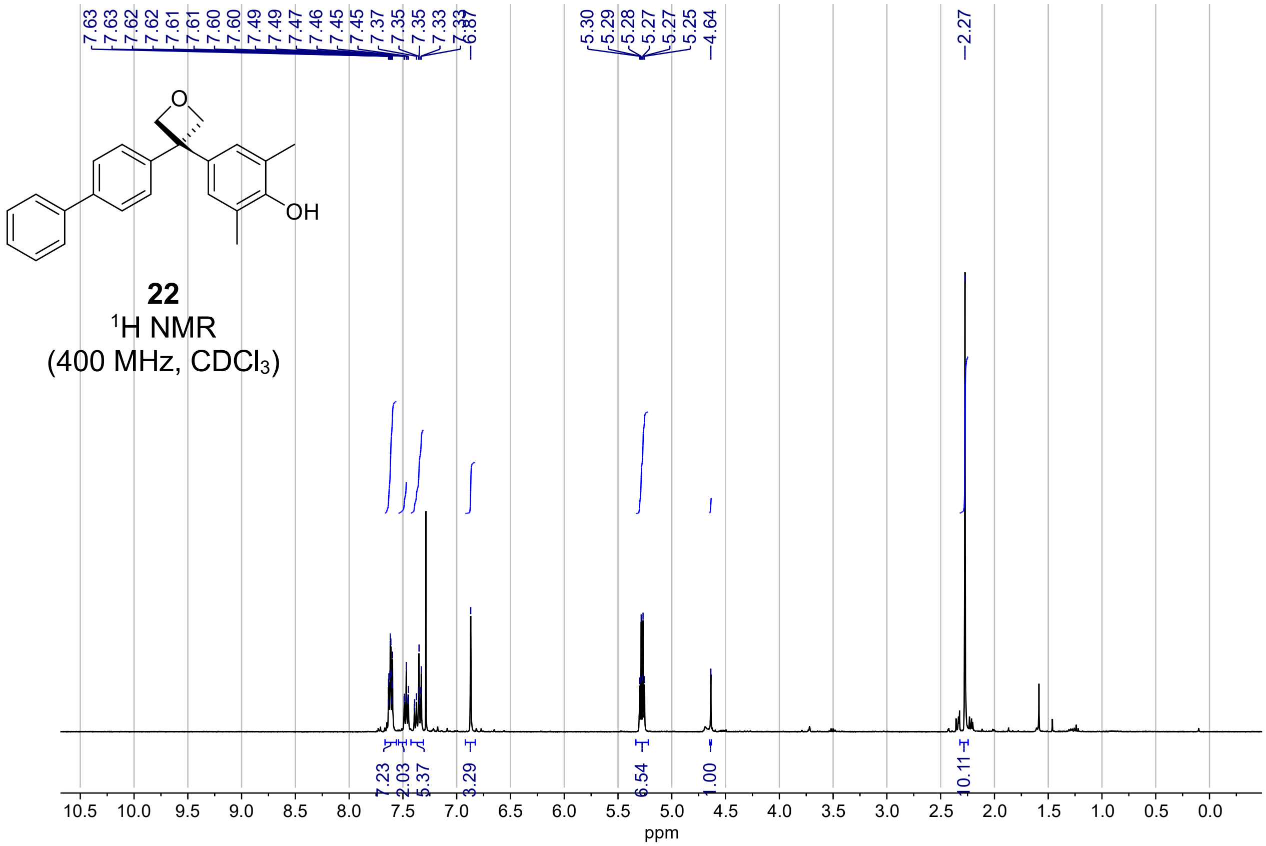

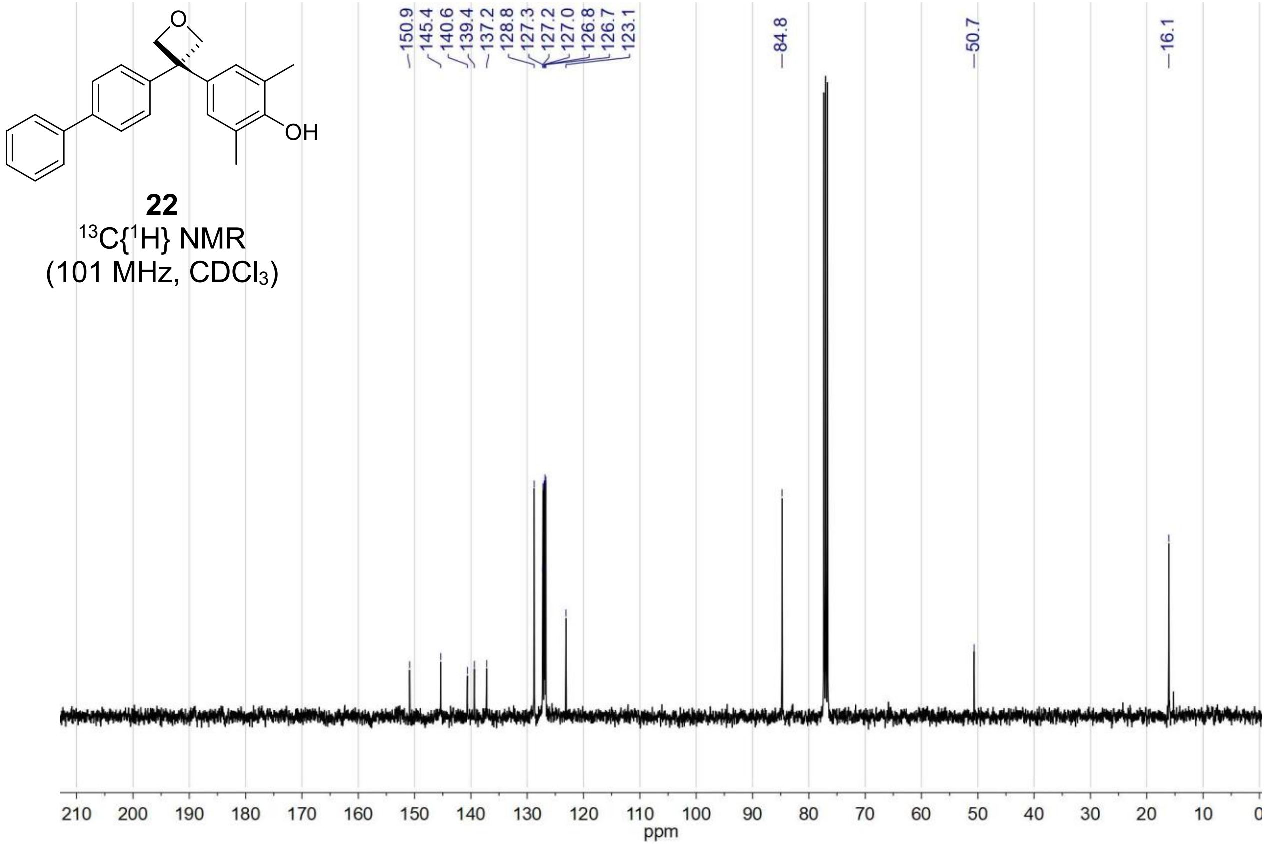

2,6-Dimethyl-4-(3-(naphthalen-2-yl)oxetan-3-yl)phenol (23)

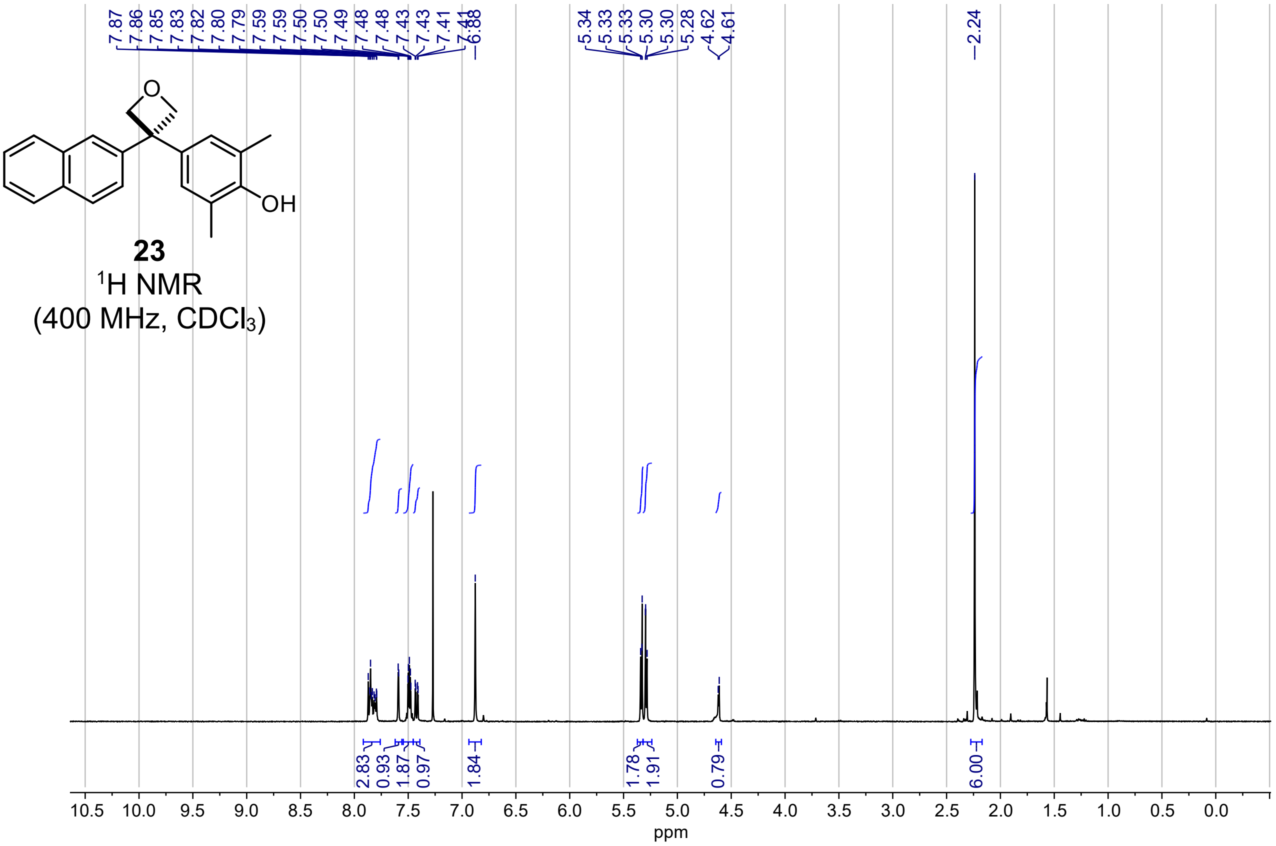

23
^{1}H NMR
(400 MHz, $CDCl_3$)

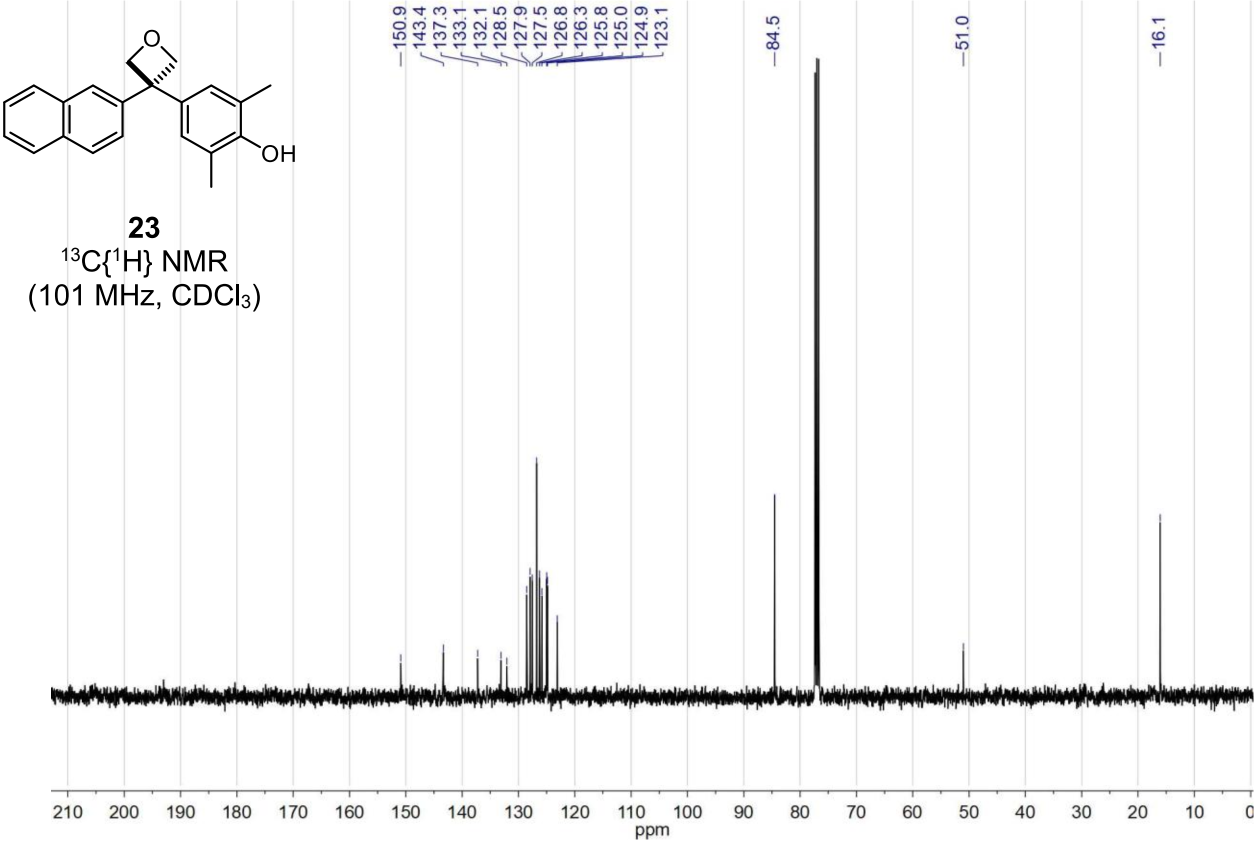

23
$^{13}C\{^{1}H\}$ NMR
(101 MHz, $CDCl_3$)

Triisopropyl(4-(3-phenyloxetan-3-yl)phenoxy)silane (25)

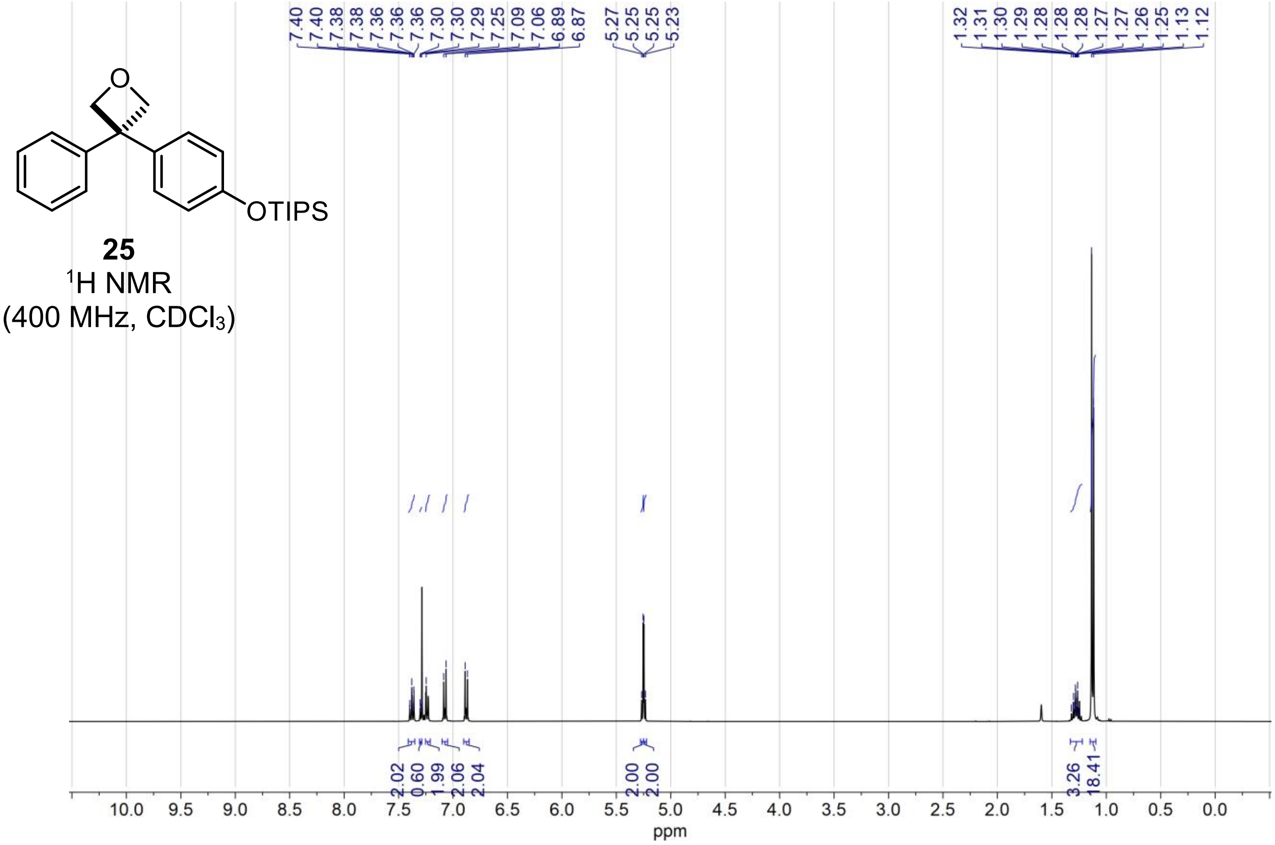

25
^{1}H NMR
(400 MHz, $CDCl_3$)

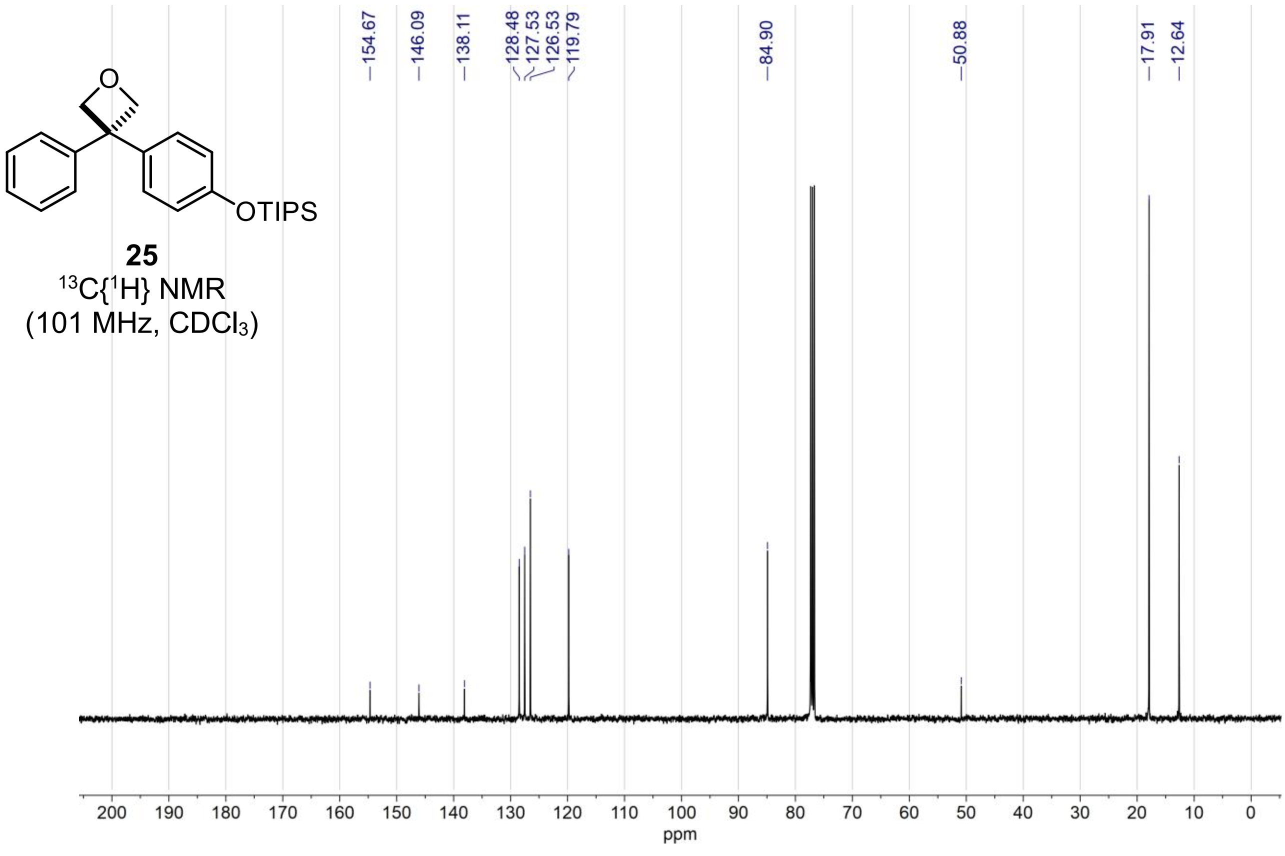

25
$^{13}C\{^1H\}$ NMR
(101 MHz, $CDCl_3$)

3-(4-Bromophenyl)-3-(4-methoxyphenyl)oxetane (27)

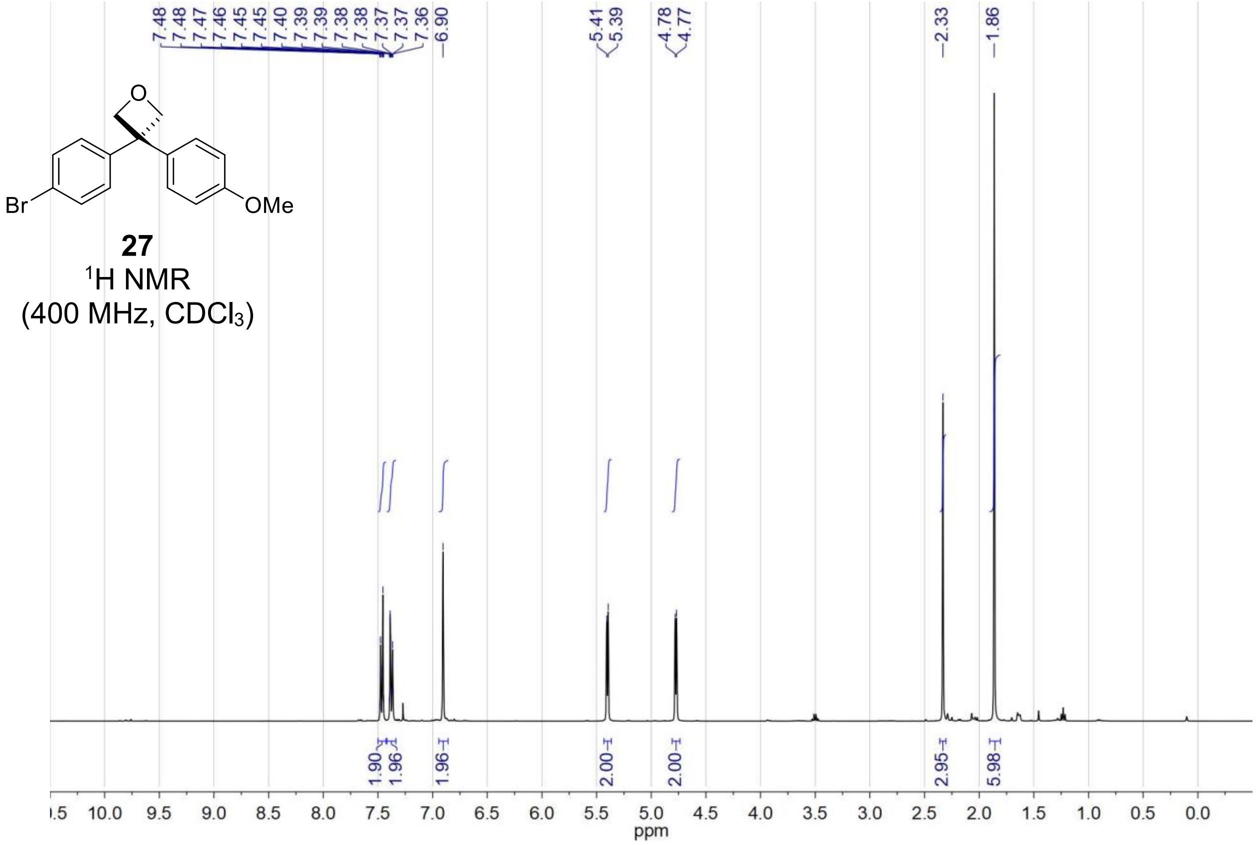

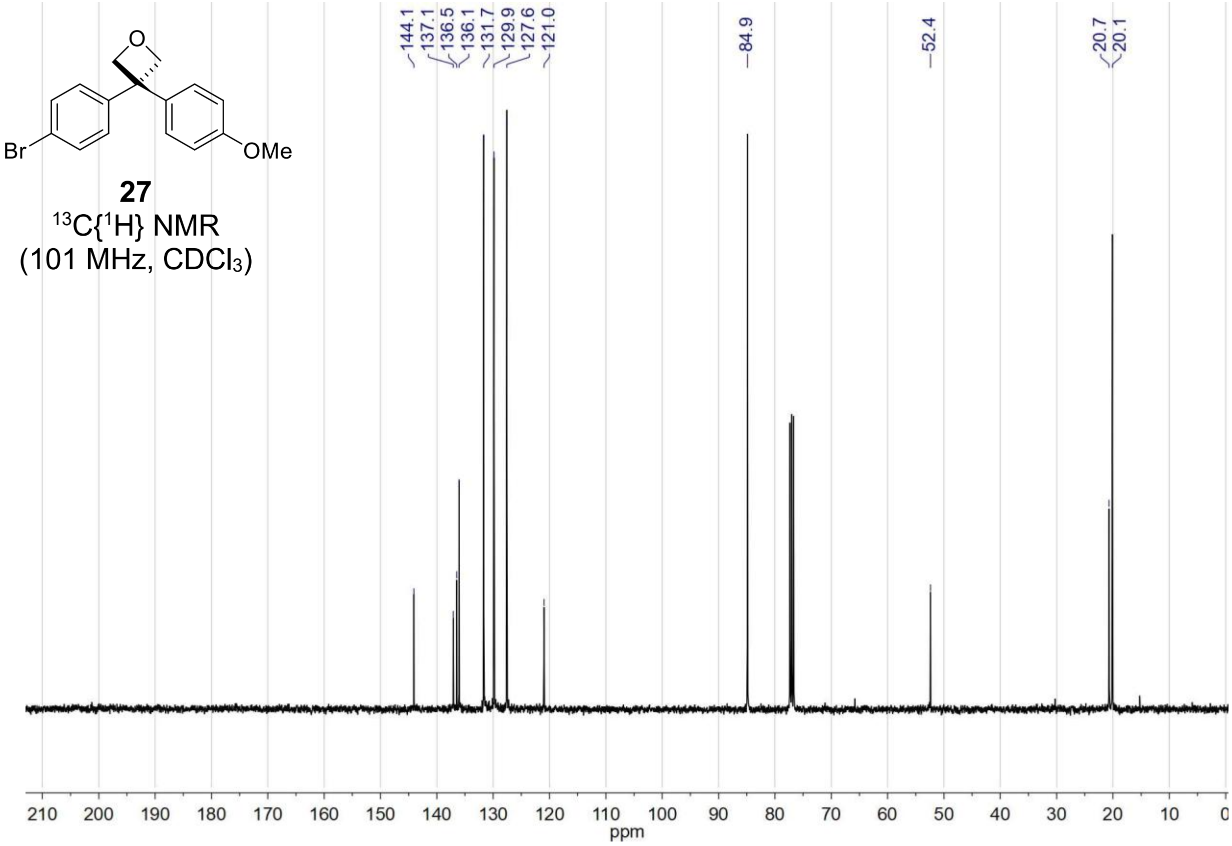

3,3-Bis(4-methoxyphenyl)oxetane (28) and 1,3-bis(4-methoxyphenyl)propan-2-one (28c)

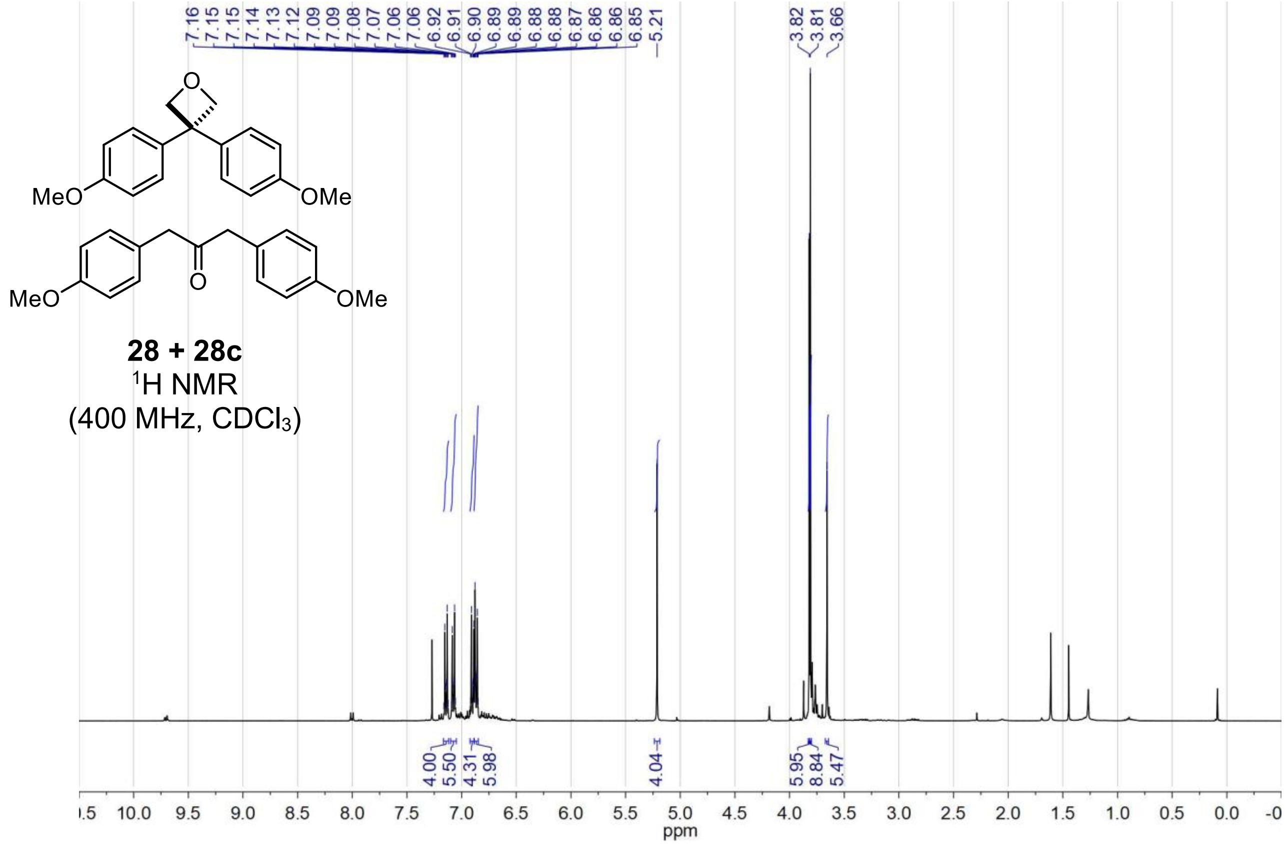

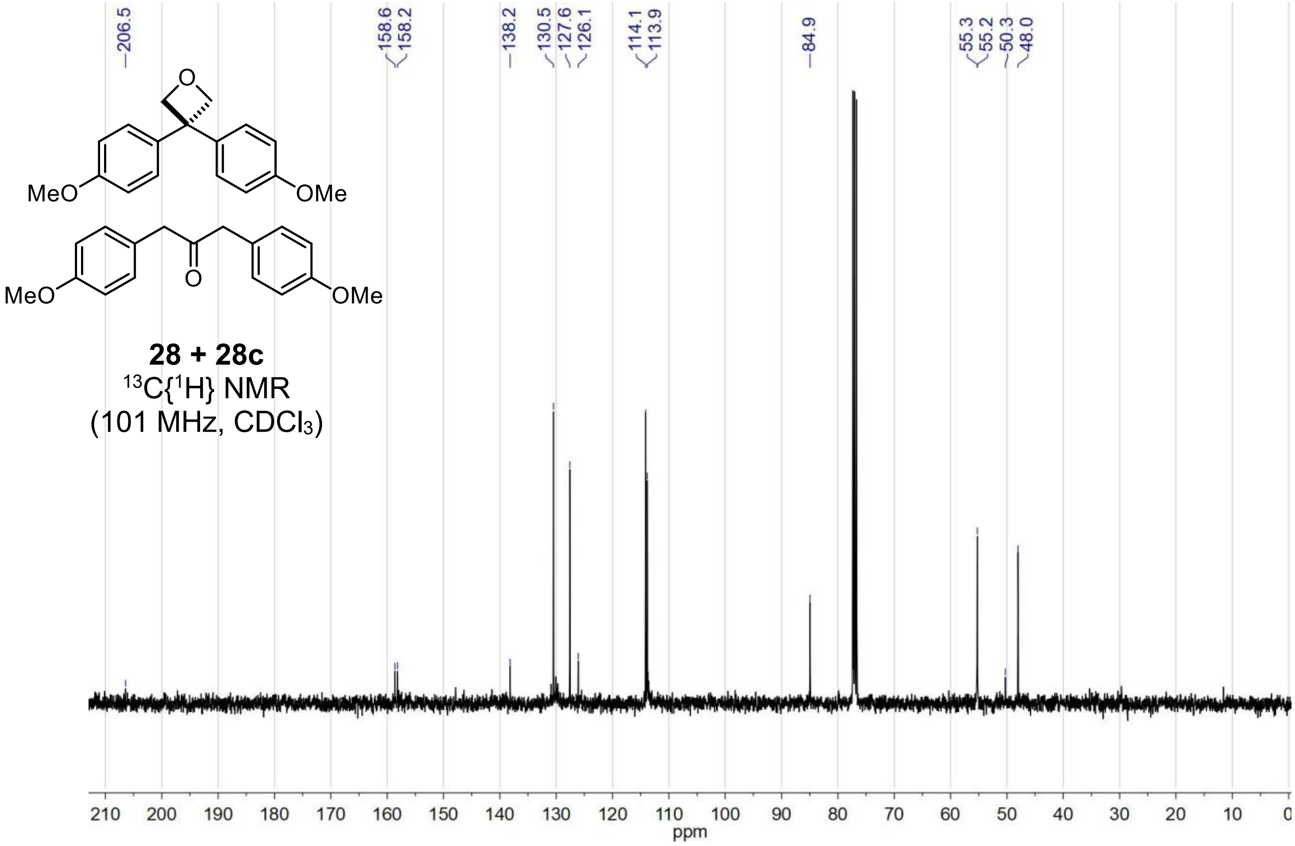

2,3-Bis(4-methoxyphenyl)propanal (28a)

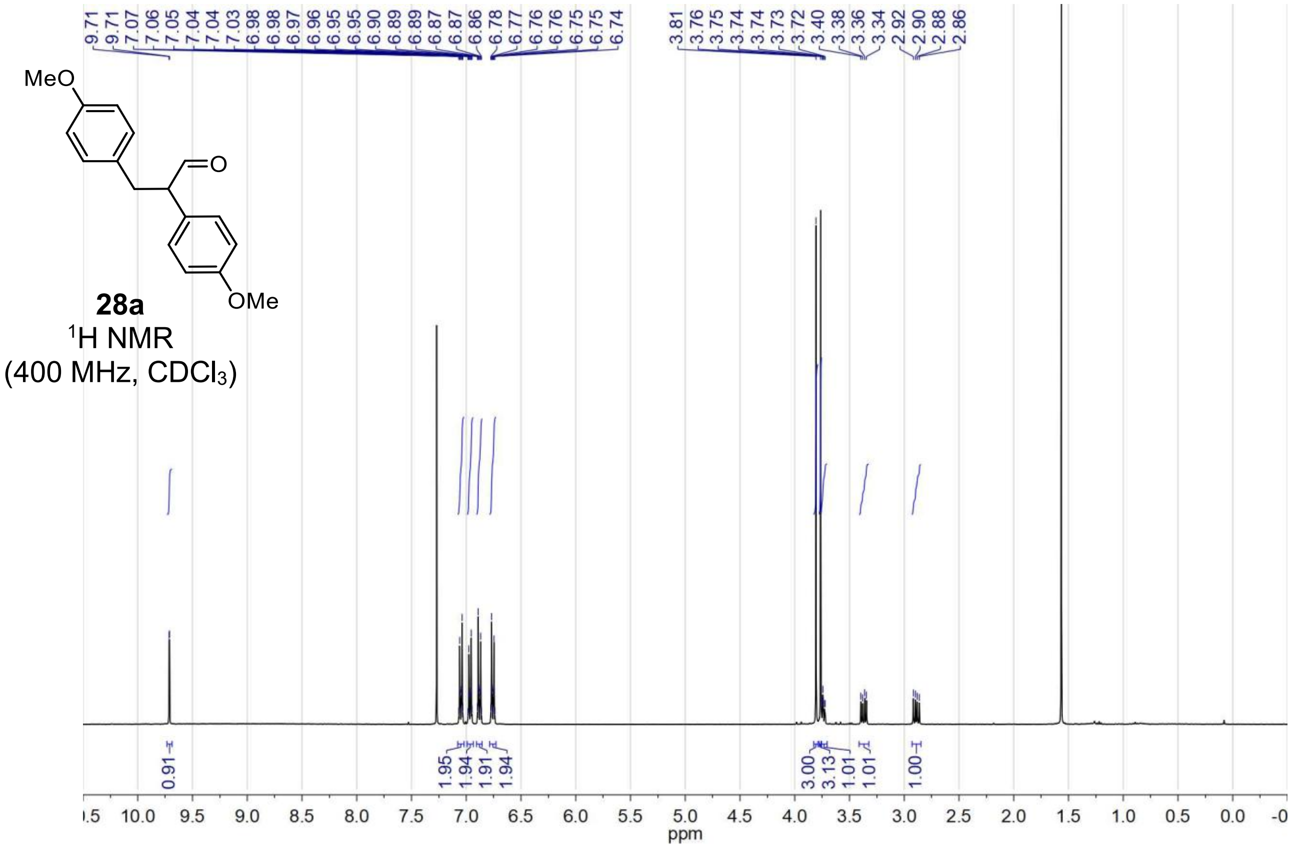

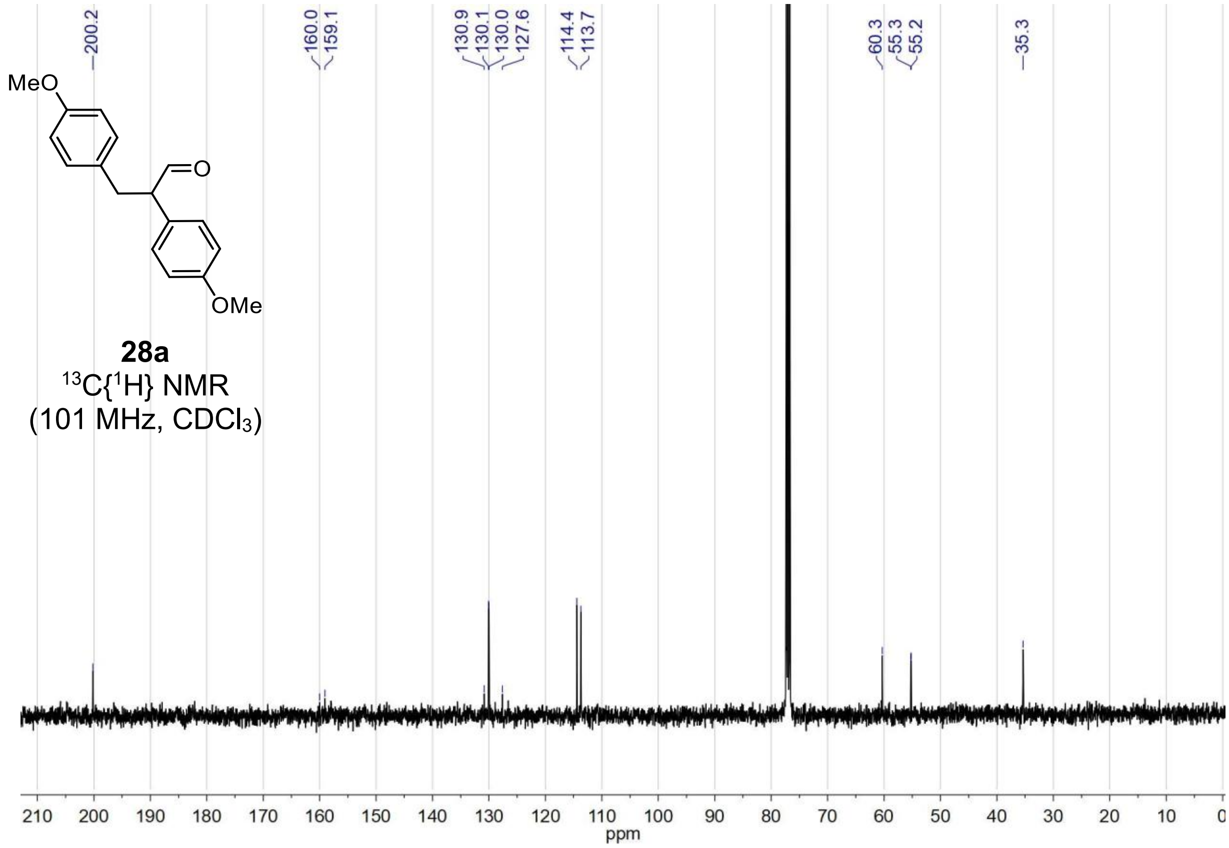

3-(3-Methoxyphenyl)-3-(4-methoxyphenyl)oxetane (29)

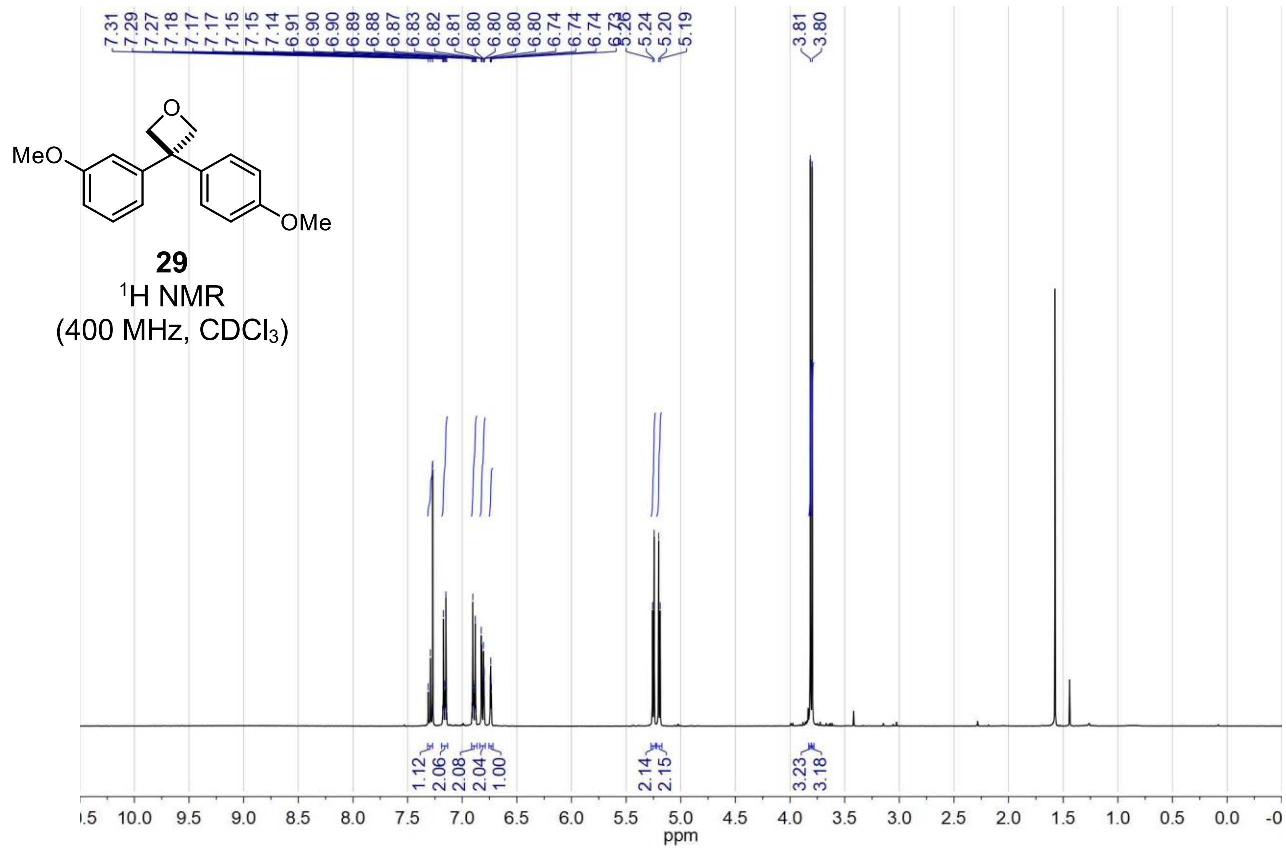

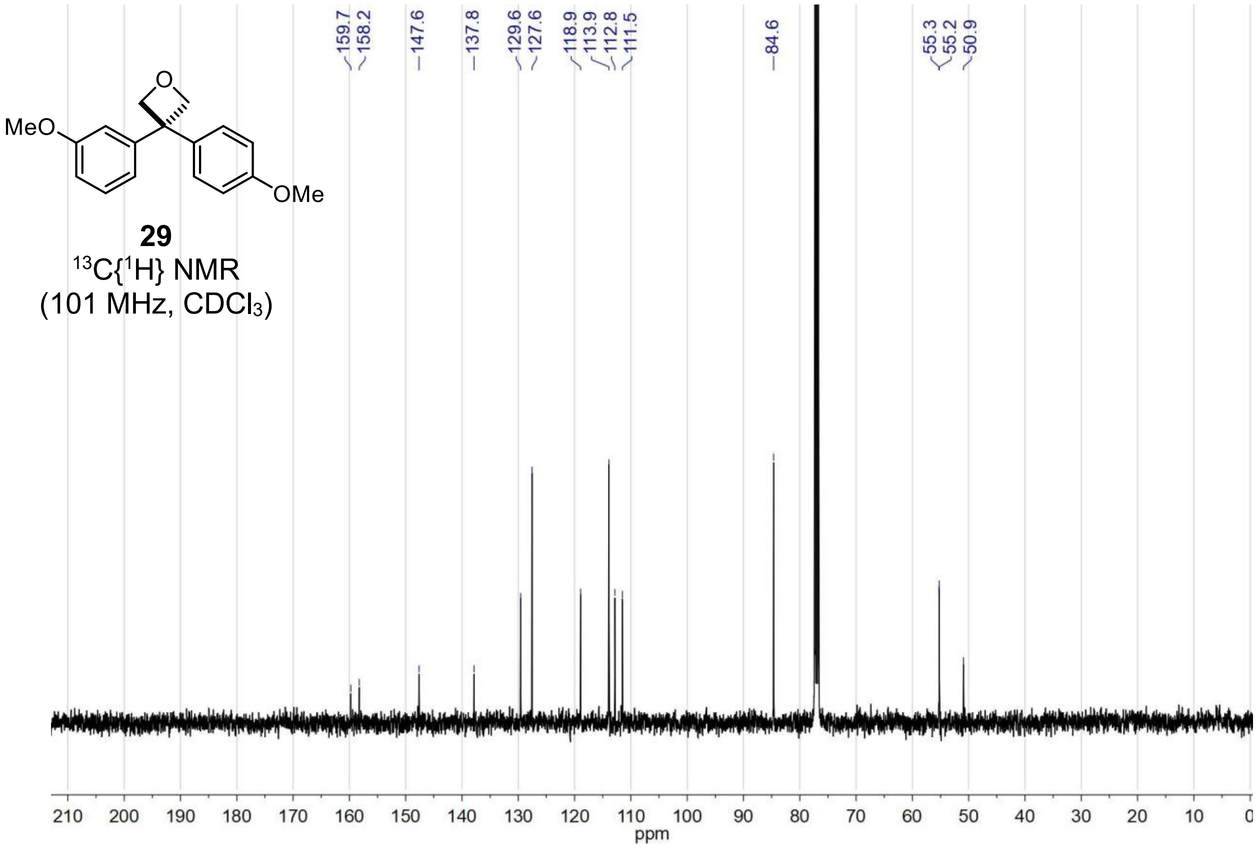

2-(2-Methoxyphenyl)-3-(4-methoxyphenyl)propanal (30a)

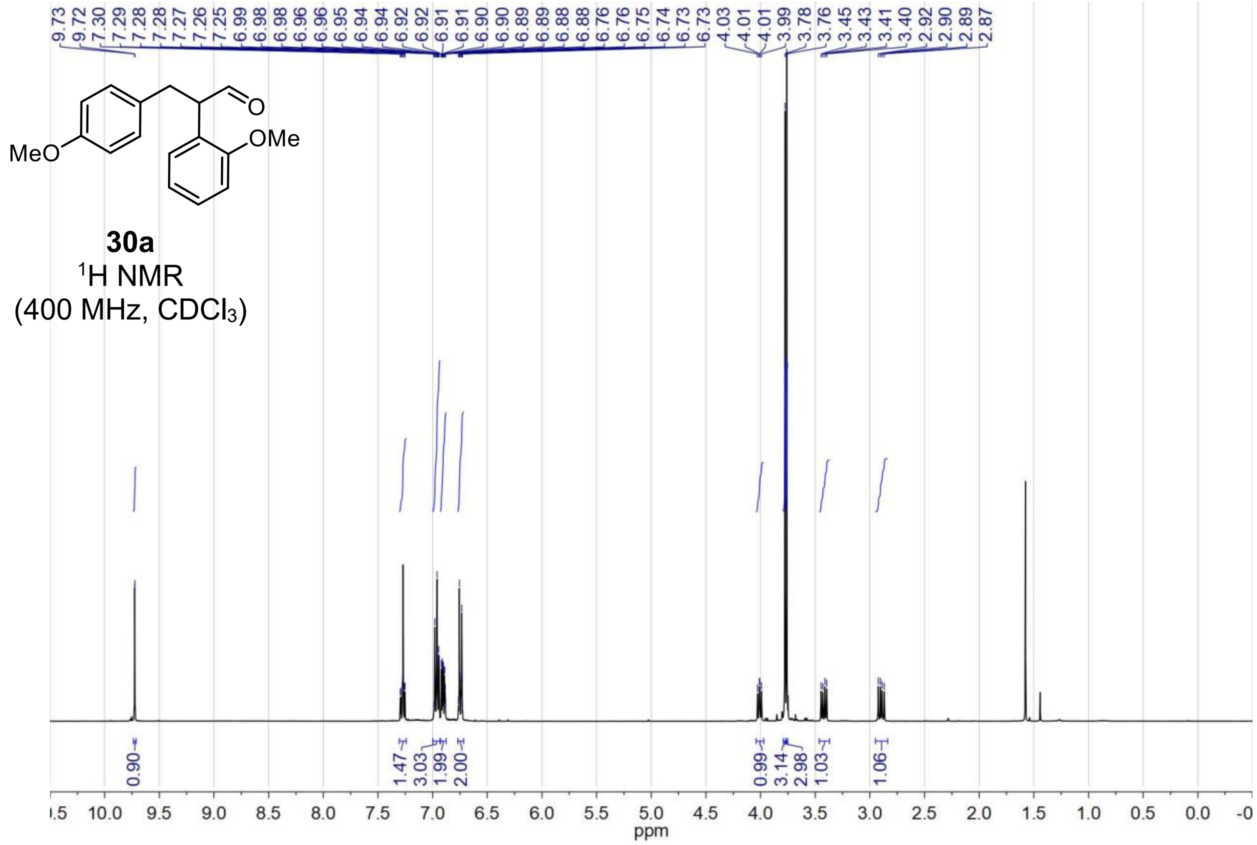

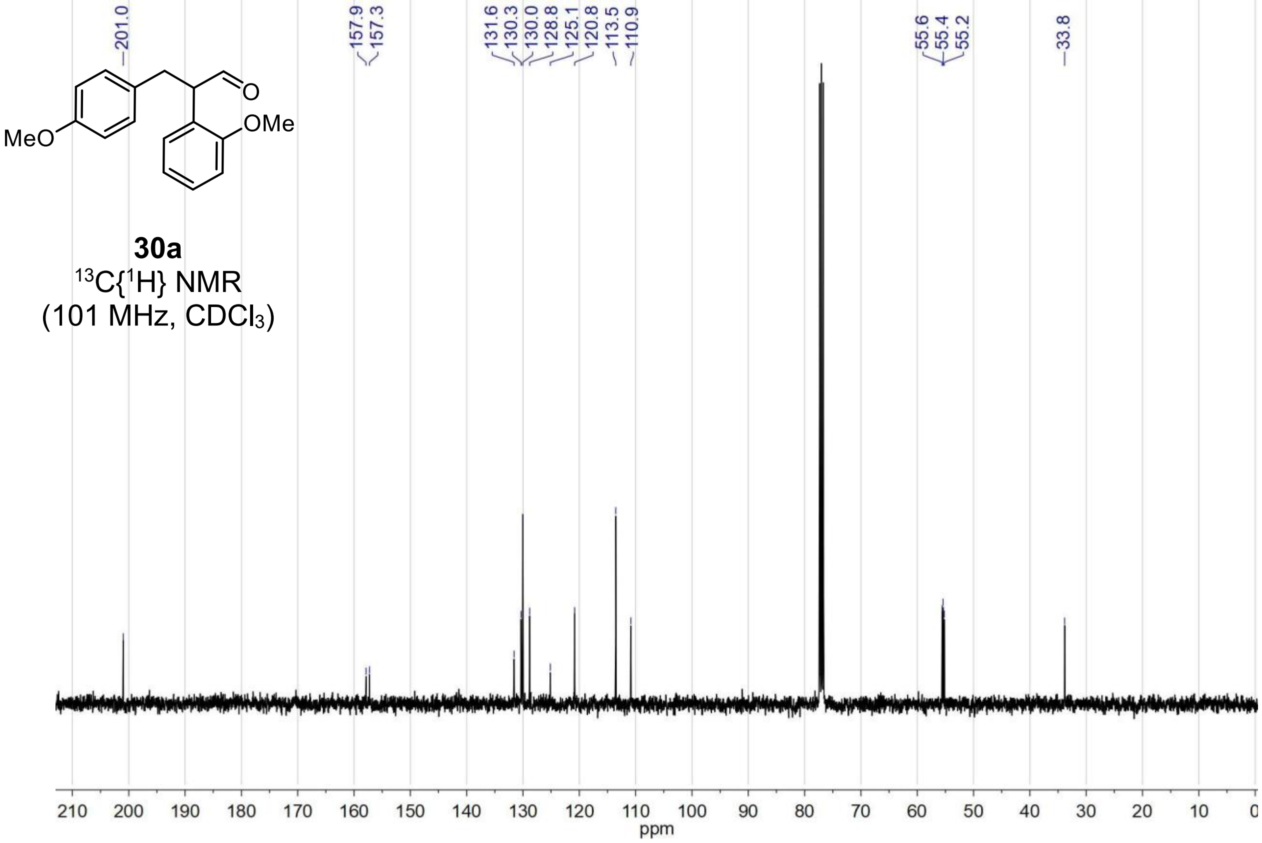

3-(4-(2-Bromoethoxy)phenyl)-3-phenyloxetane (31)

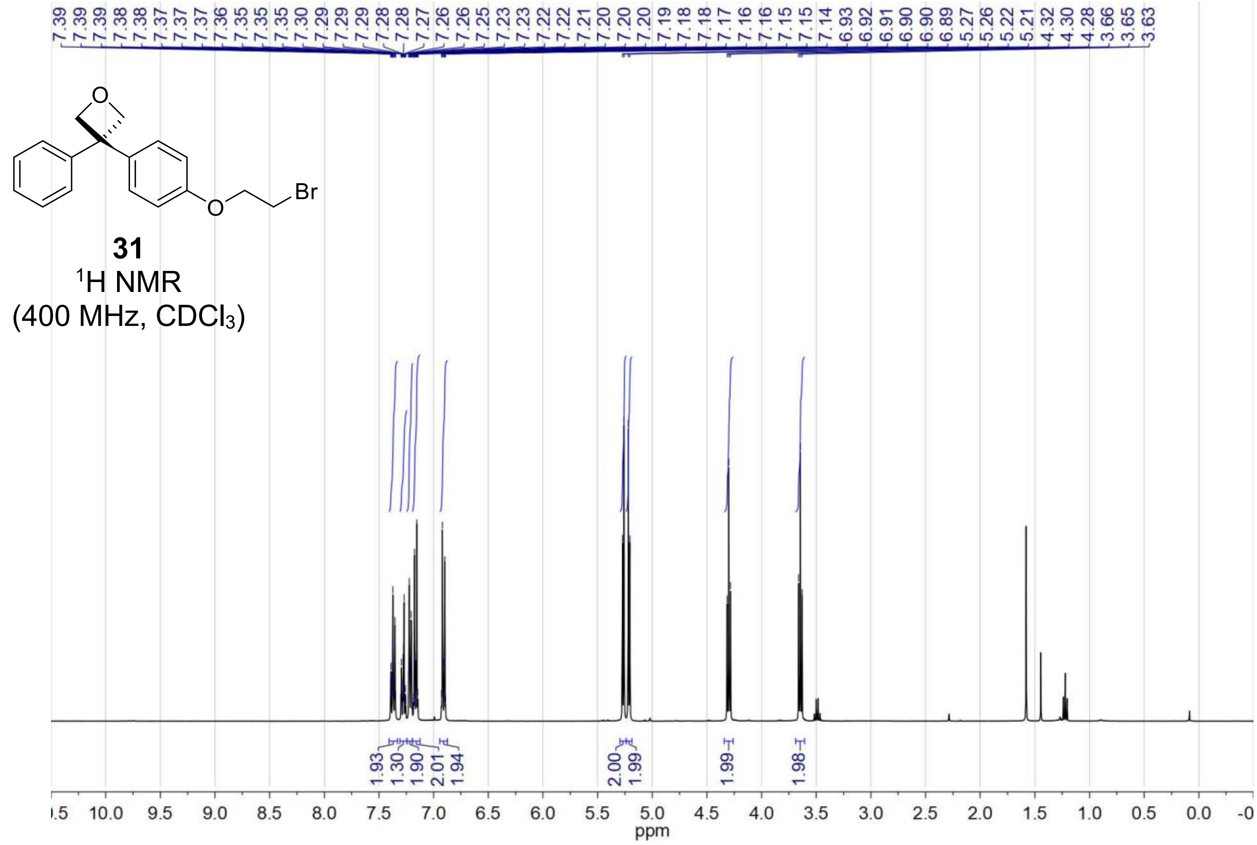

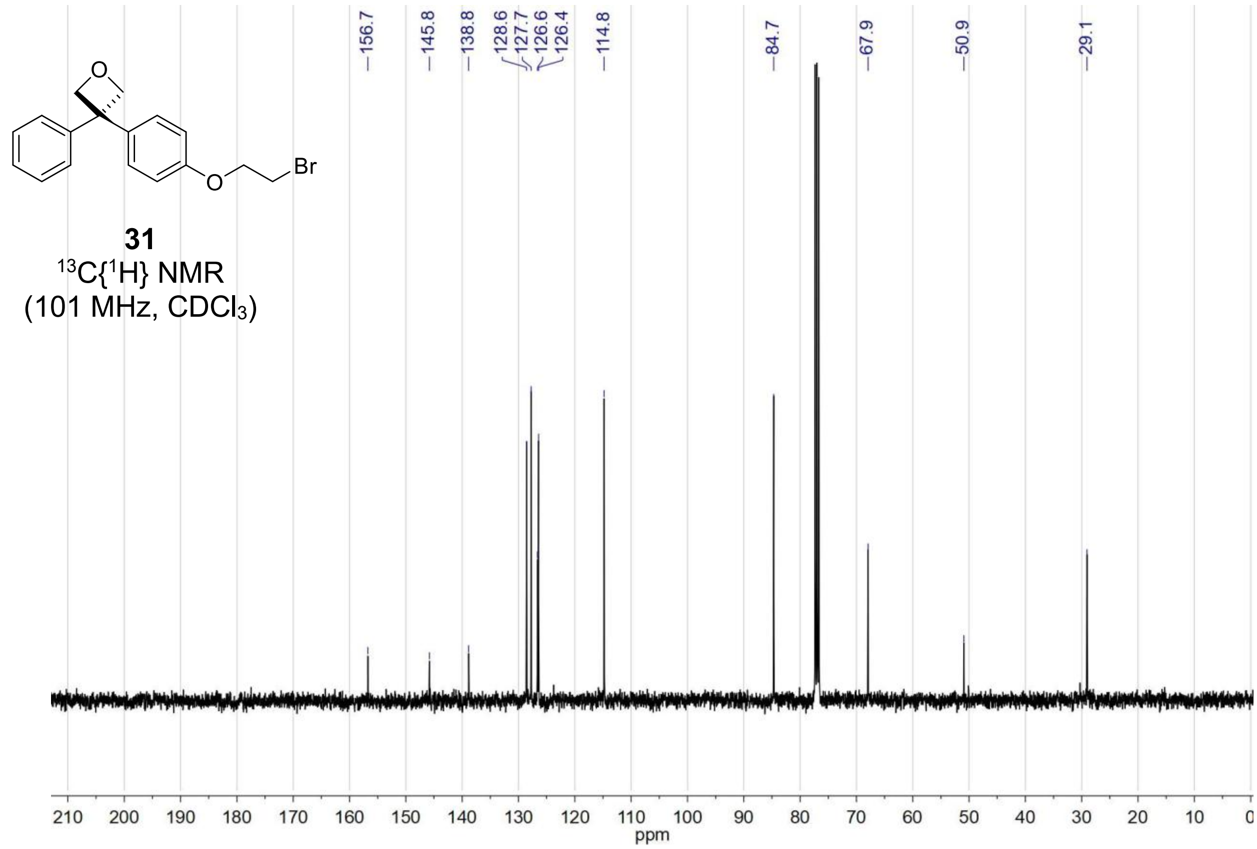

3-(2-(2-Bromoethoxy)phenyl)-3-phenyloxetane (*o*-31)

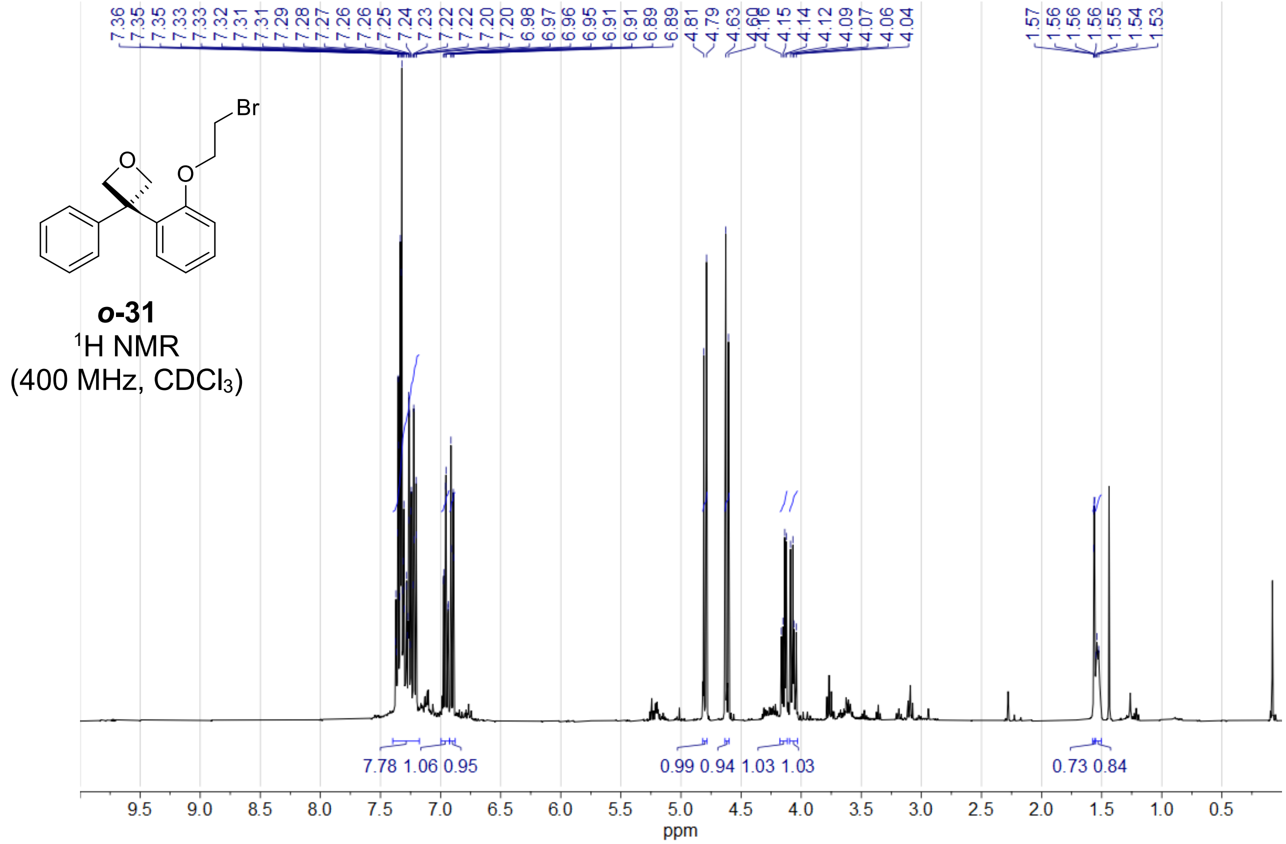

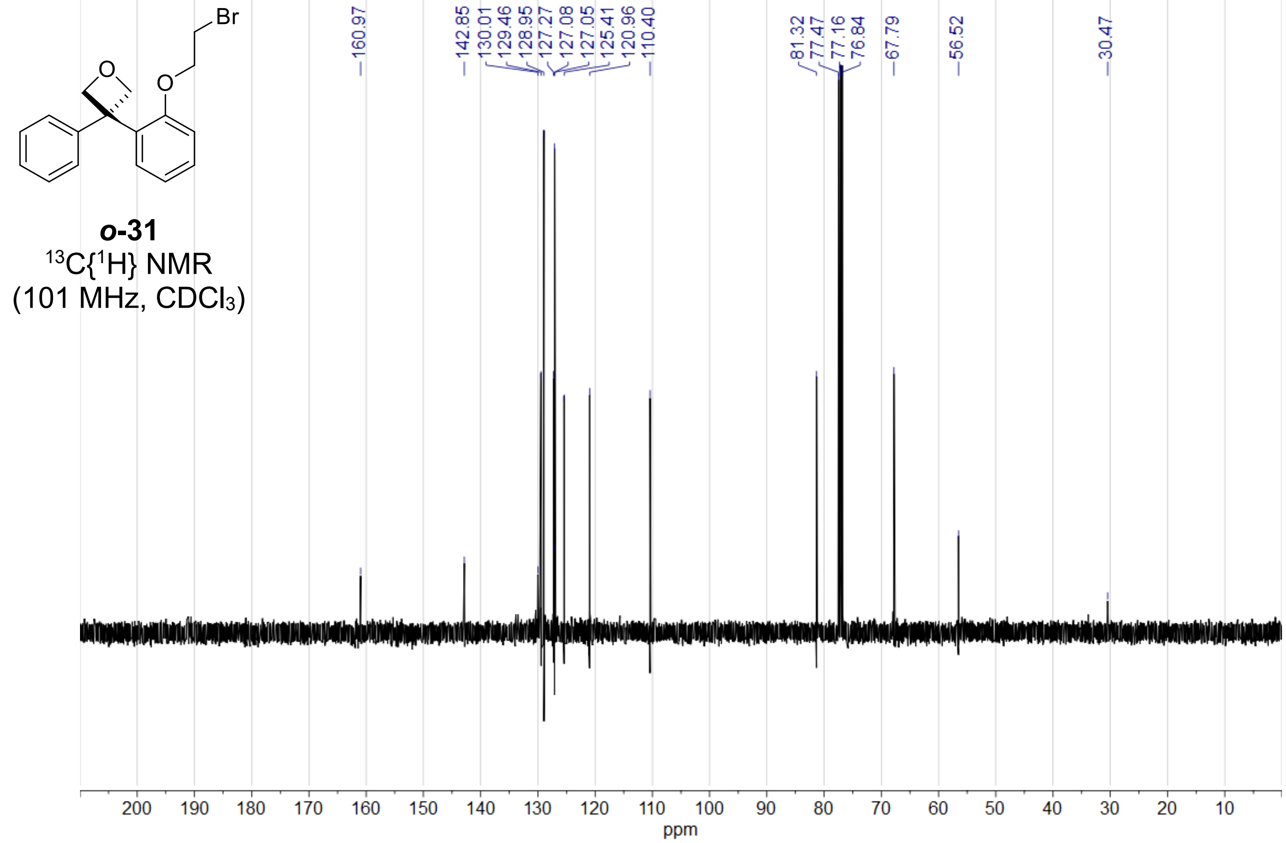

3-(4-(2-Bromoethoxy)phenyl)-2-phenylpropanal (31a)

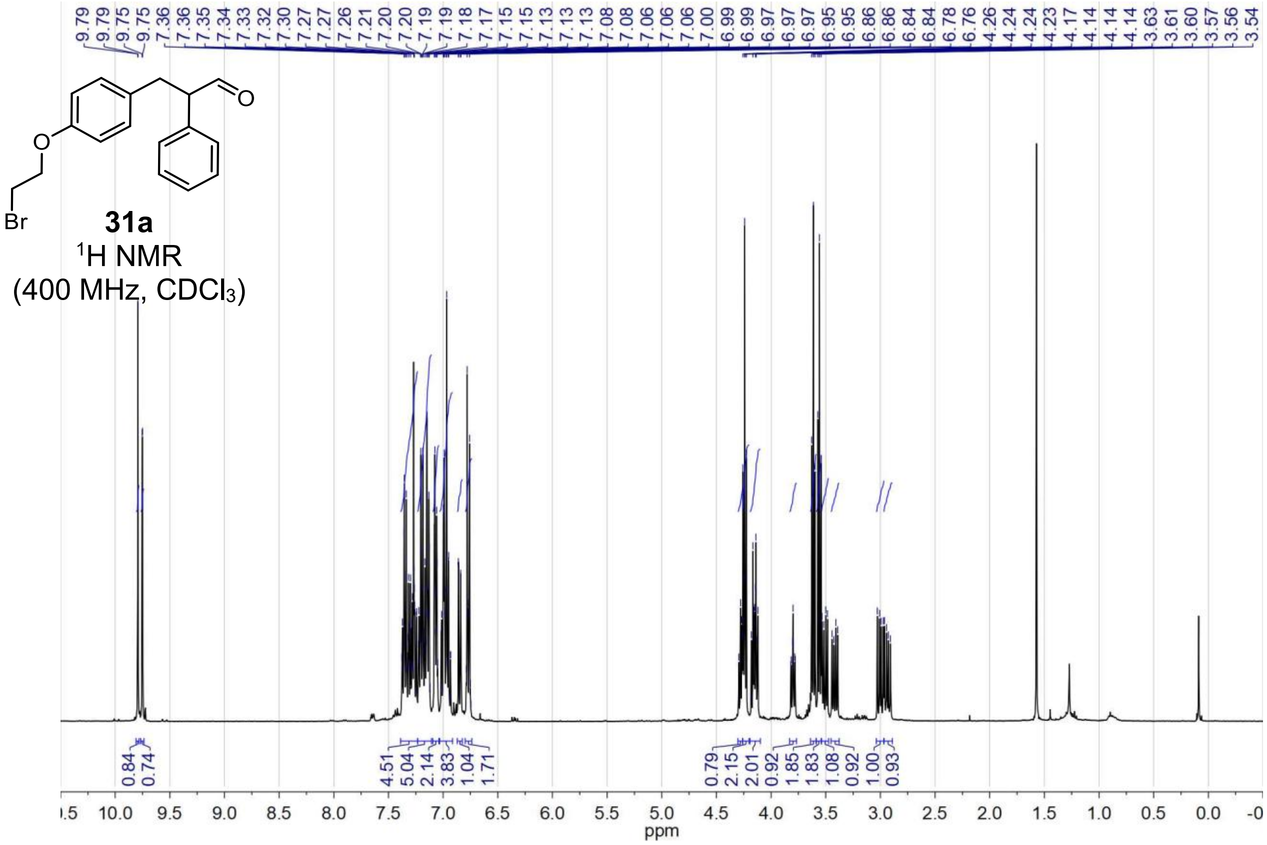

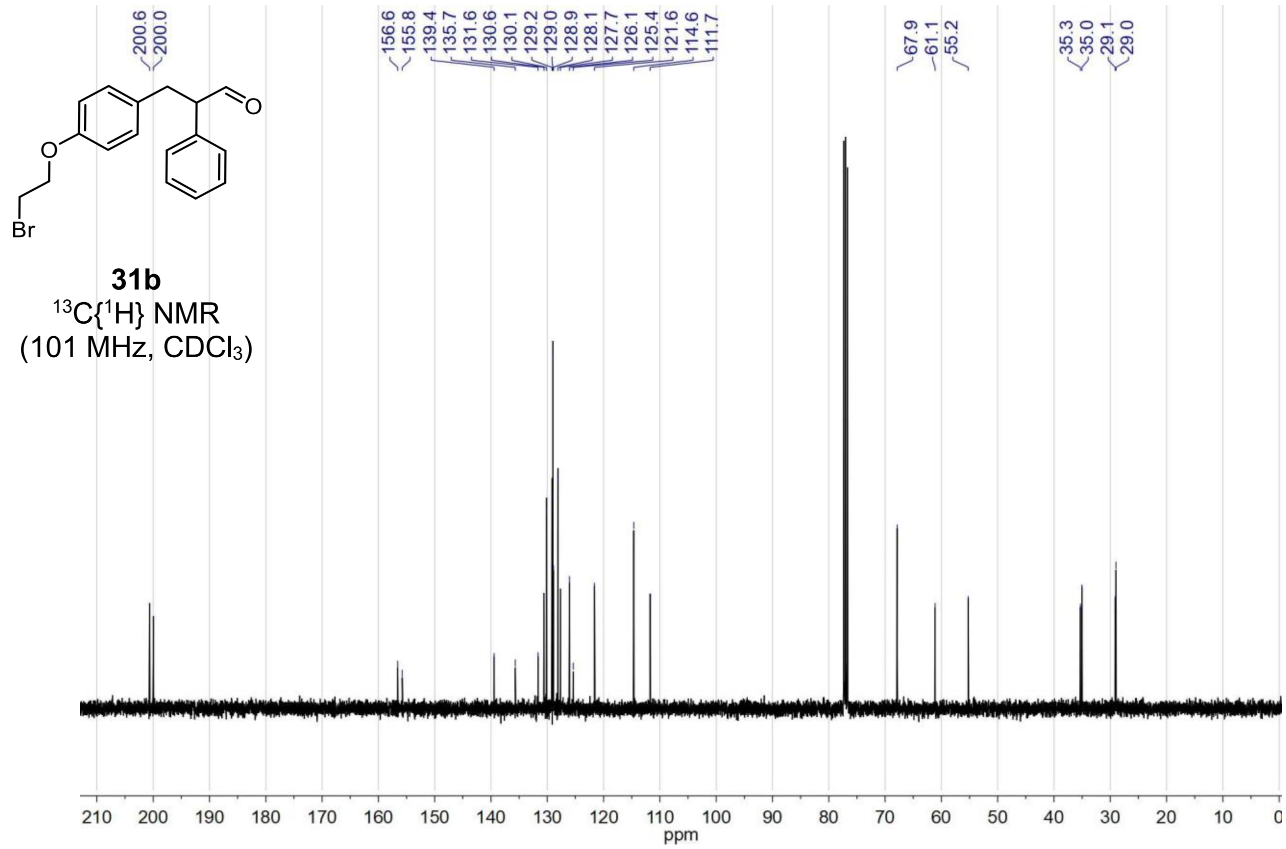

_N_,_N_-Diethyl-2-(4-(3-phenyloxetan-3-yl)phenoxy)ethan-1-amine (32)

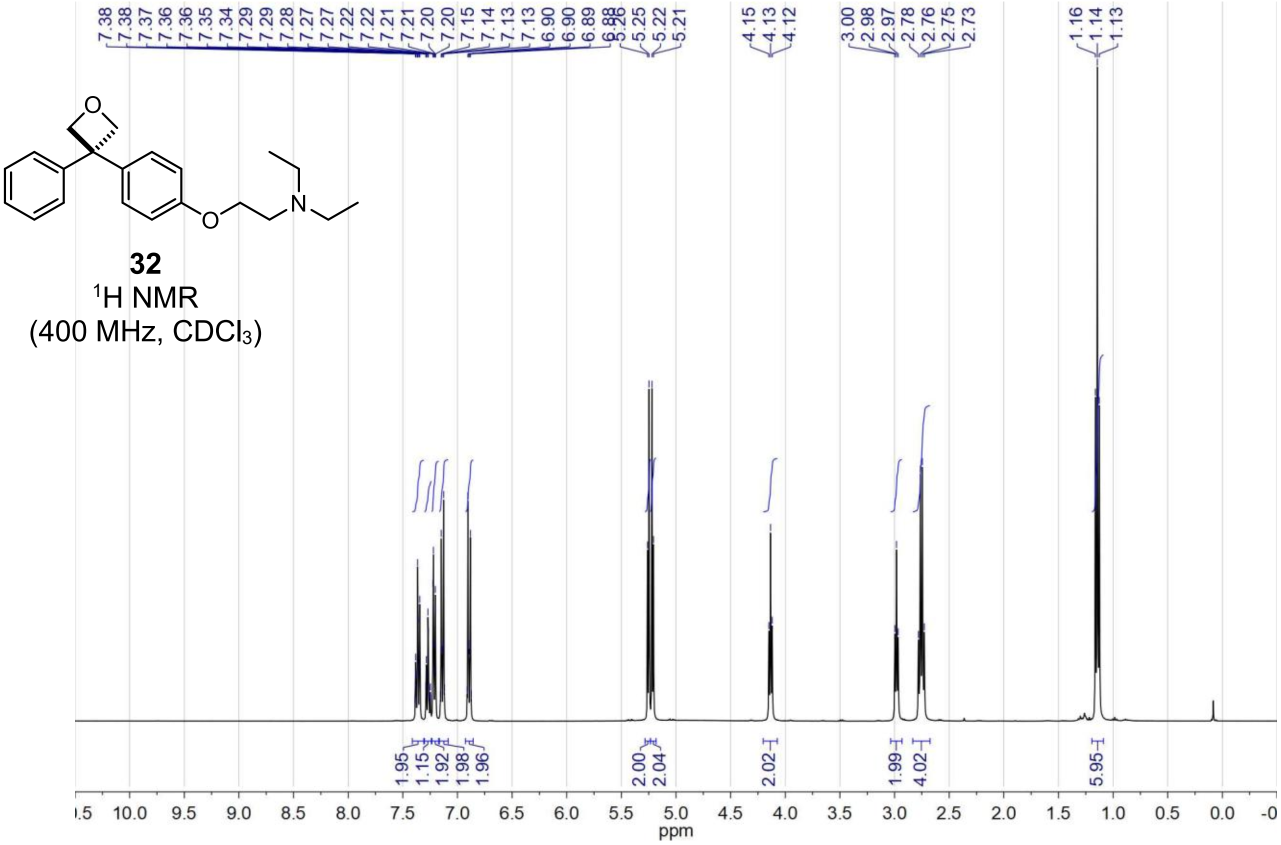

32

^{1}H NMR

(400 MHz, $CDCl_3$)

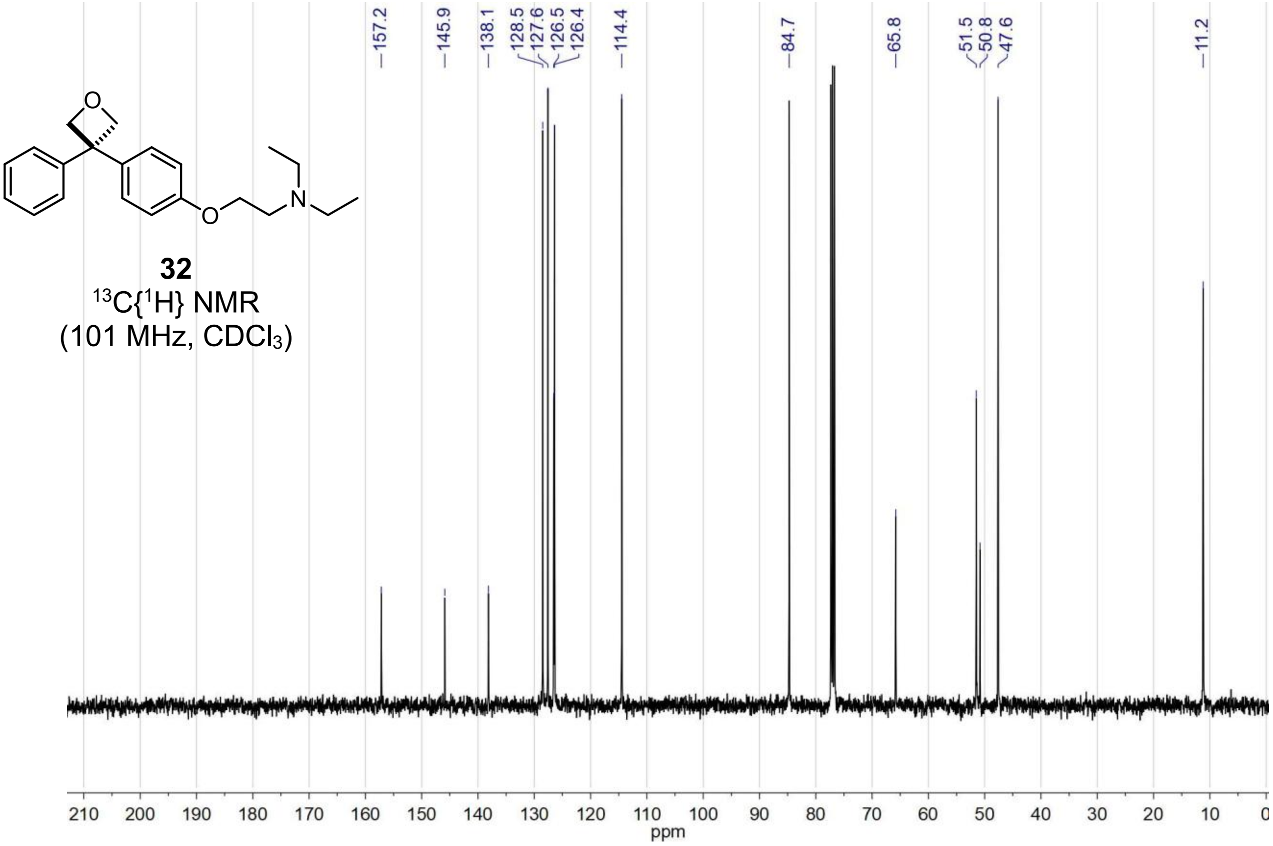

32

$^{13}C\{^{1}H\}$ NMR

(101 MHz, $CDCl_3$)

(4-(3-(4-Chlorophenyl)oxetan-3-yl)phenoxy)triisopropylsilane (33)

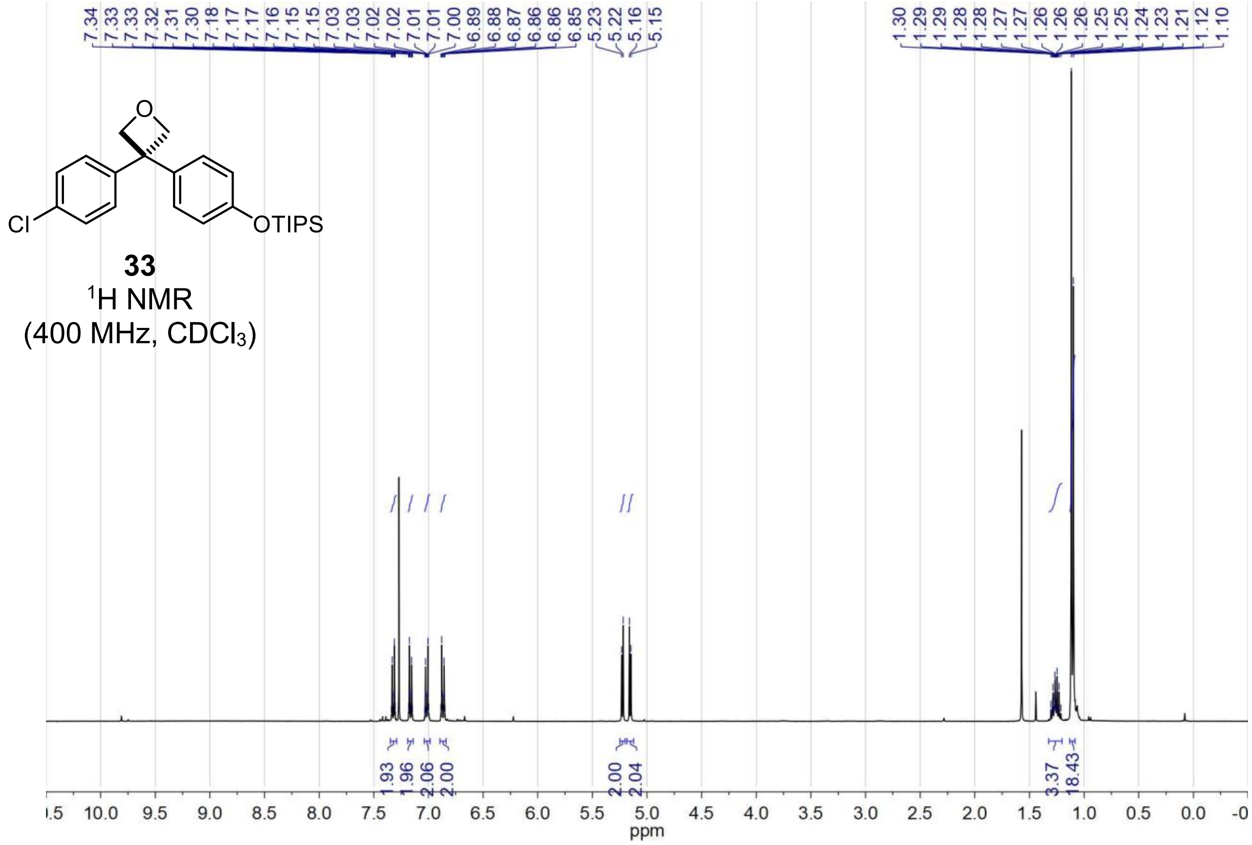

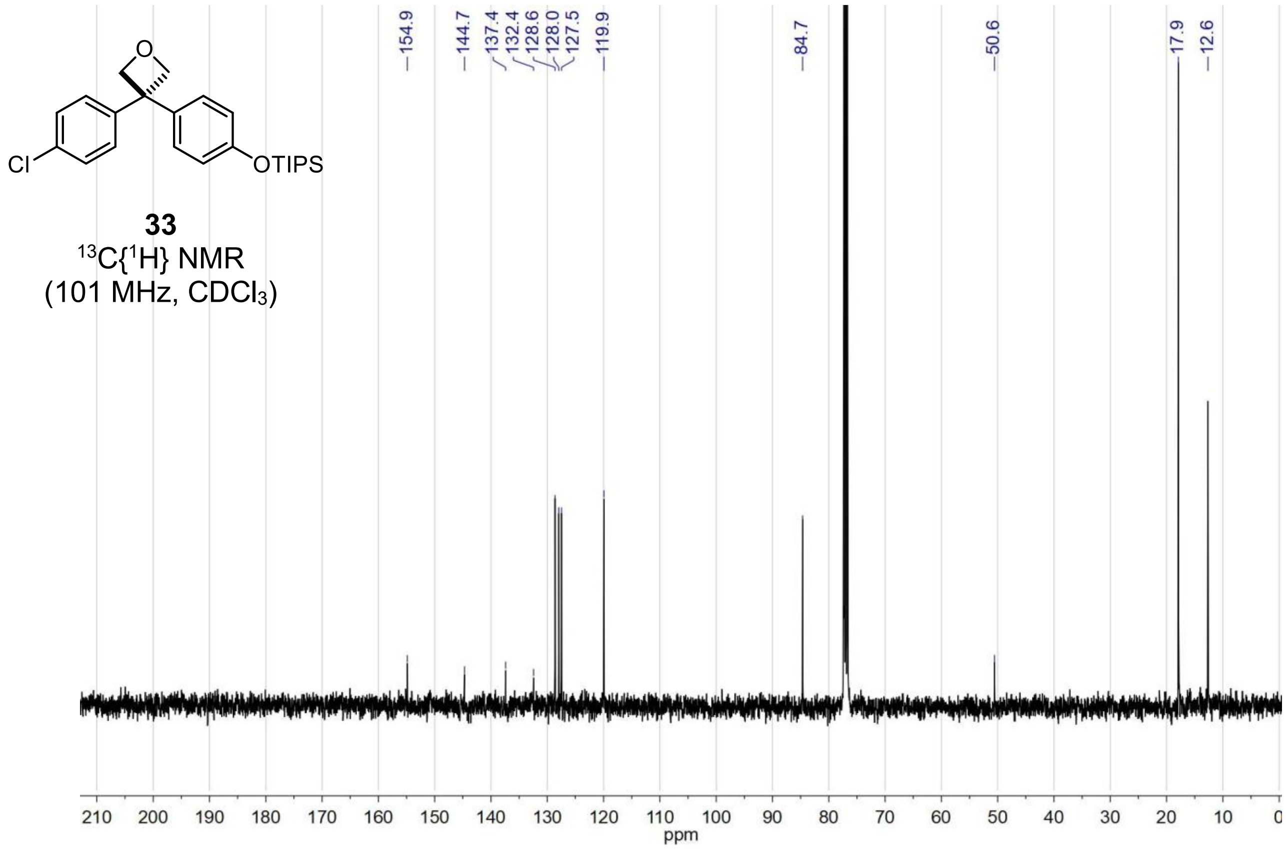

4-(3-(4-Chlorophenyl)oxetan-3-yl)phenol (34)

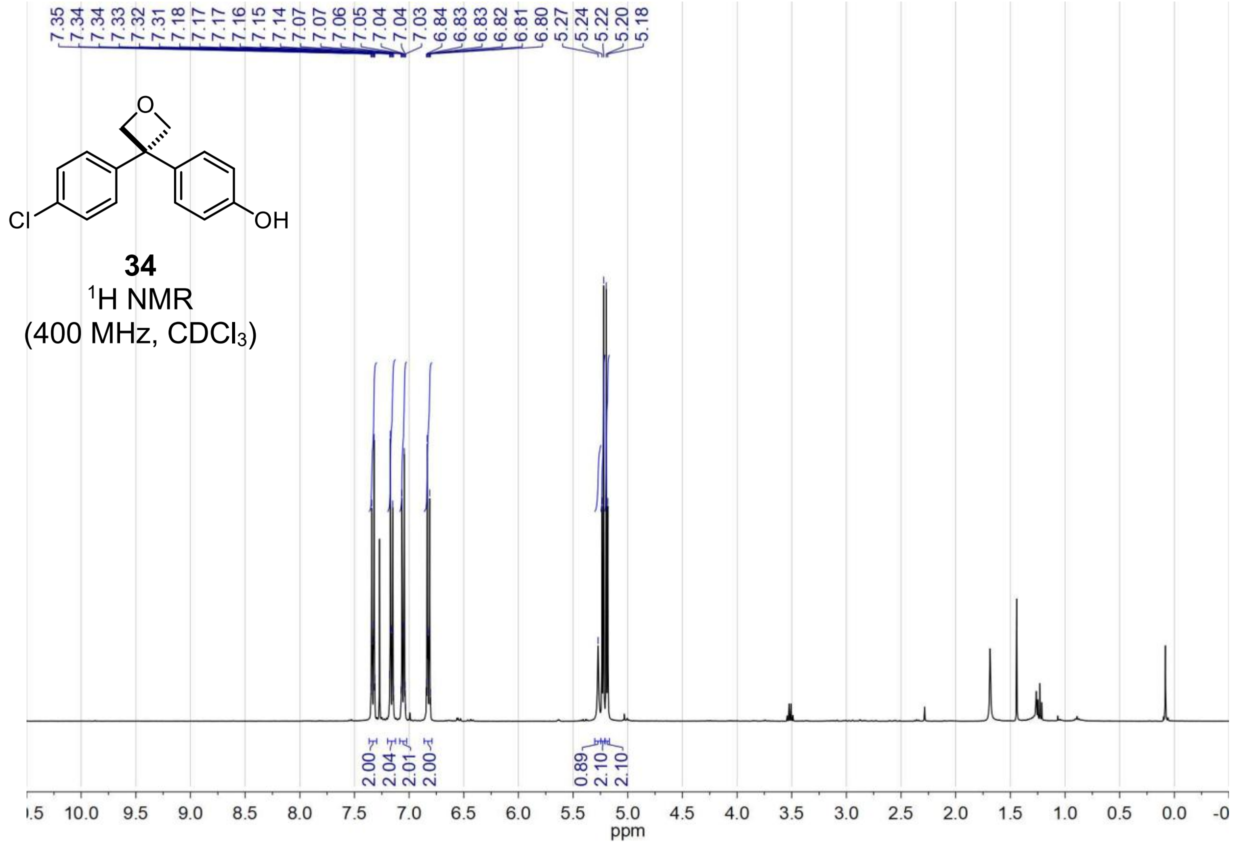

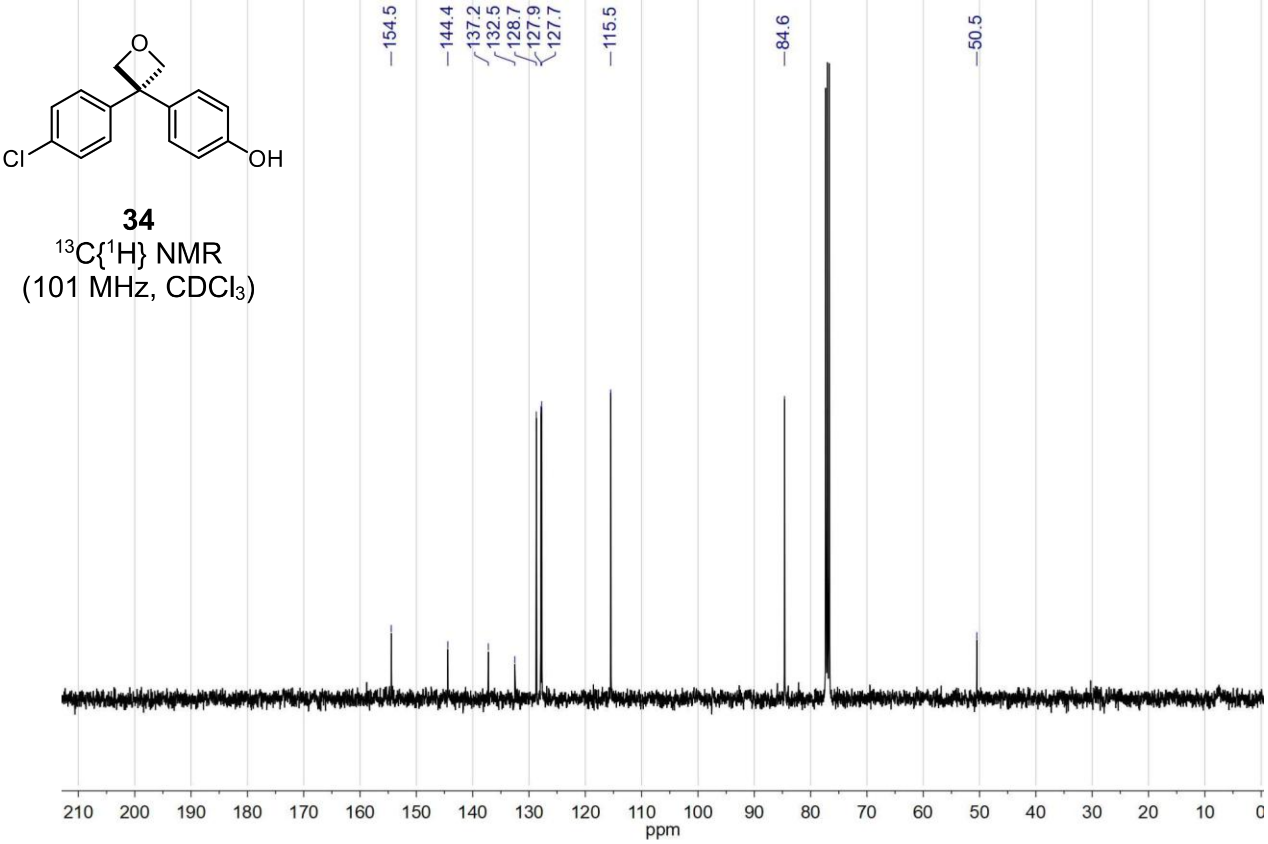

Isopropyl 2-(4-(3-(4-chlorophenyl)oxetan-3-yl)phenoxy)-2-methylpropanoate (35)

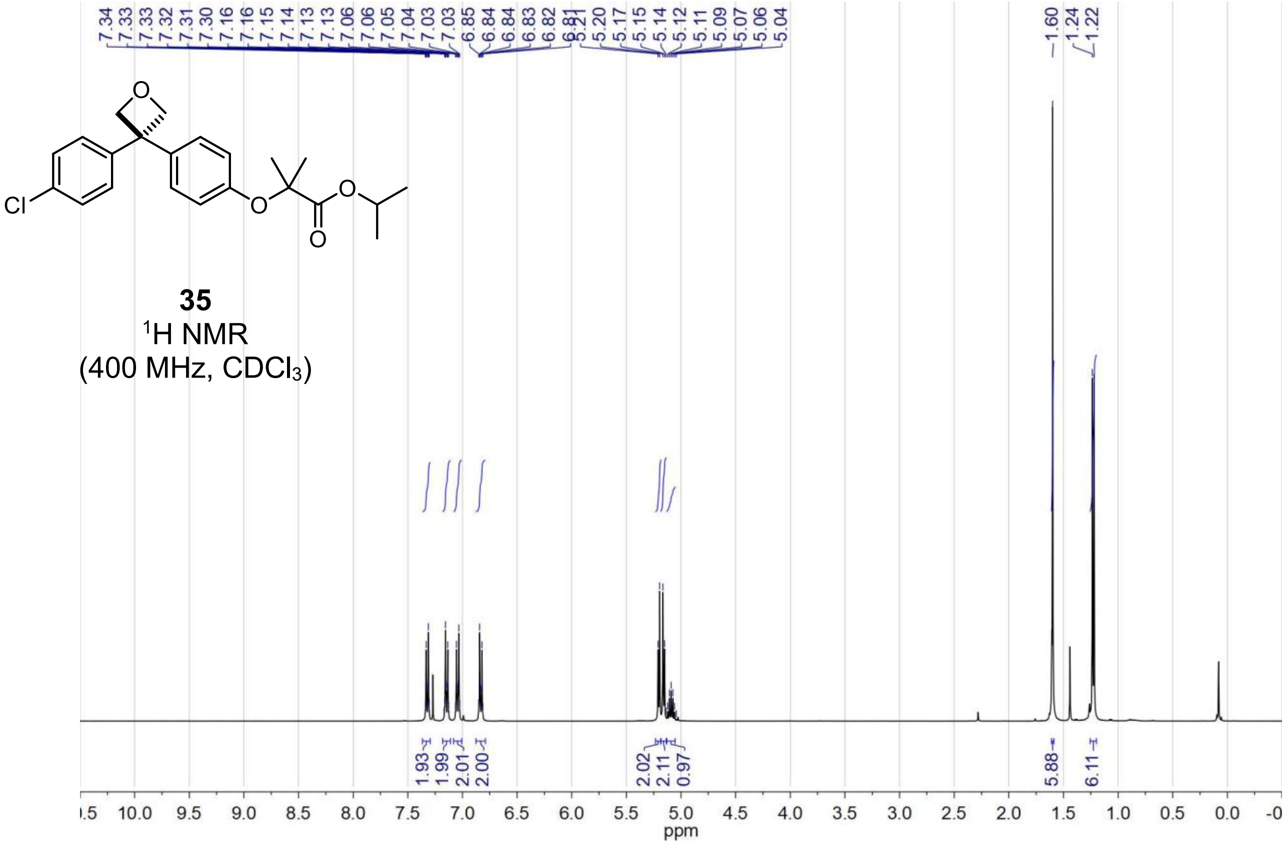

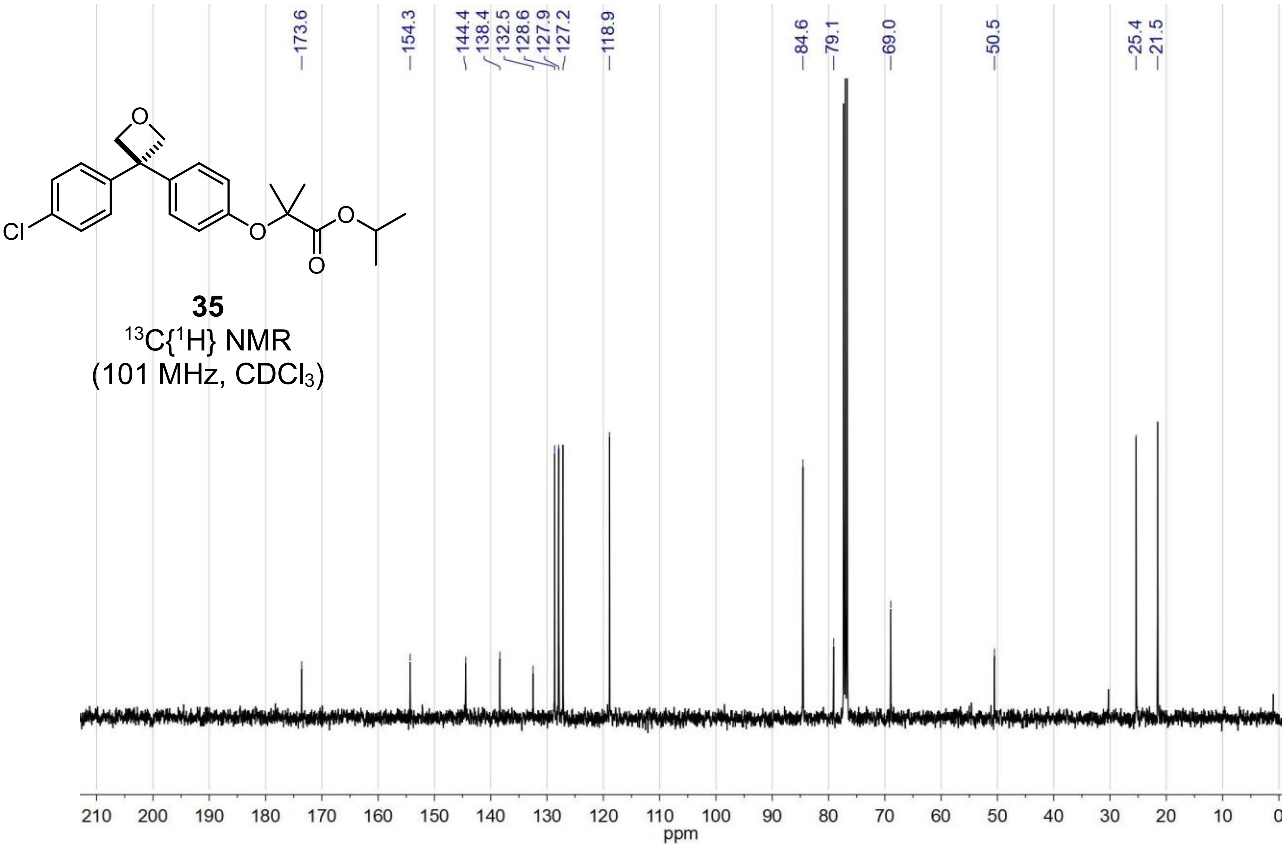

4-(4-(3-Mesityloxetan-3-yl)phenyl)-1-methyl-1H-pyrazole (36)

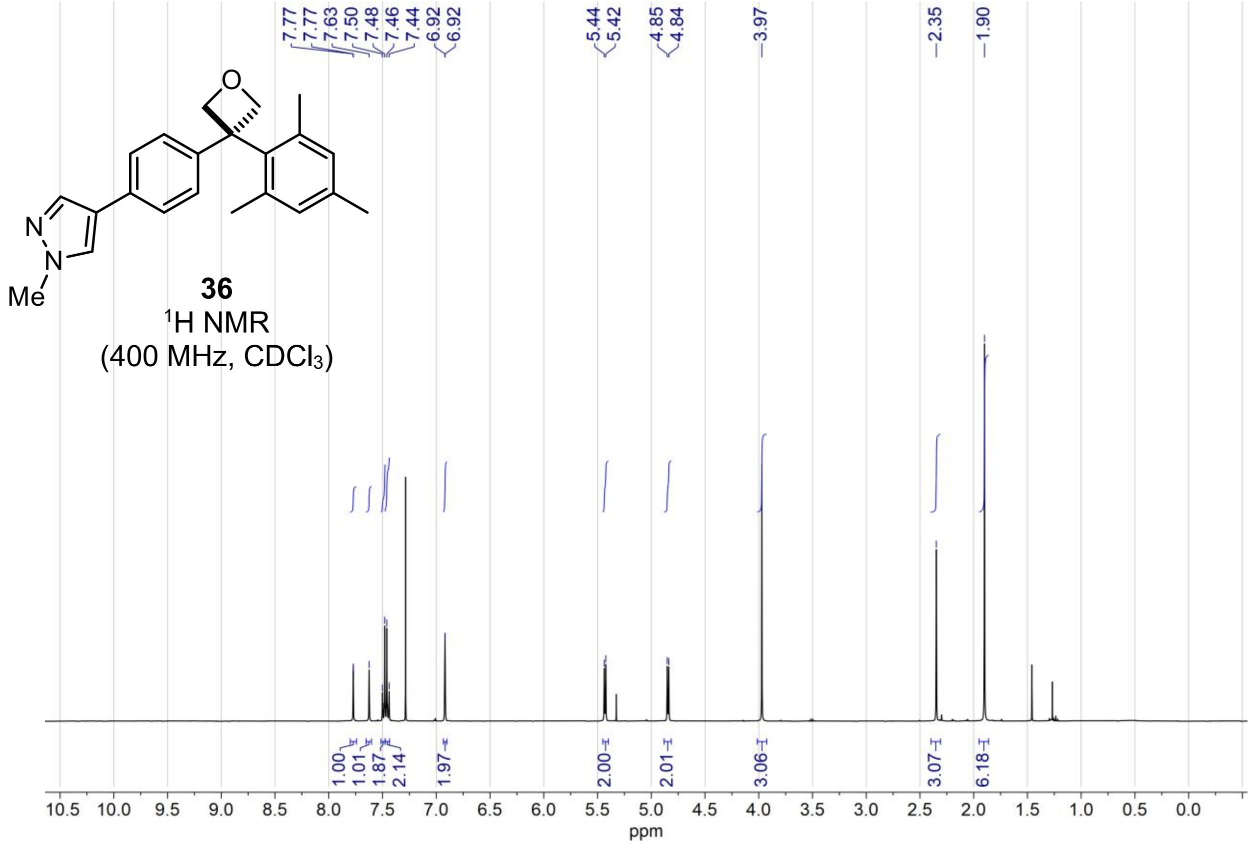

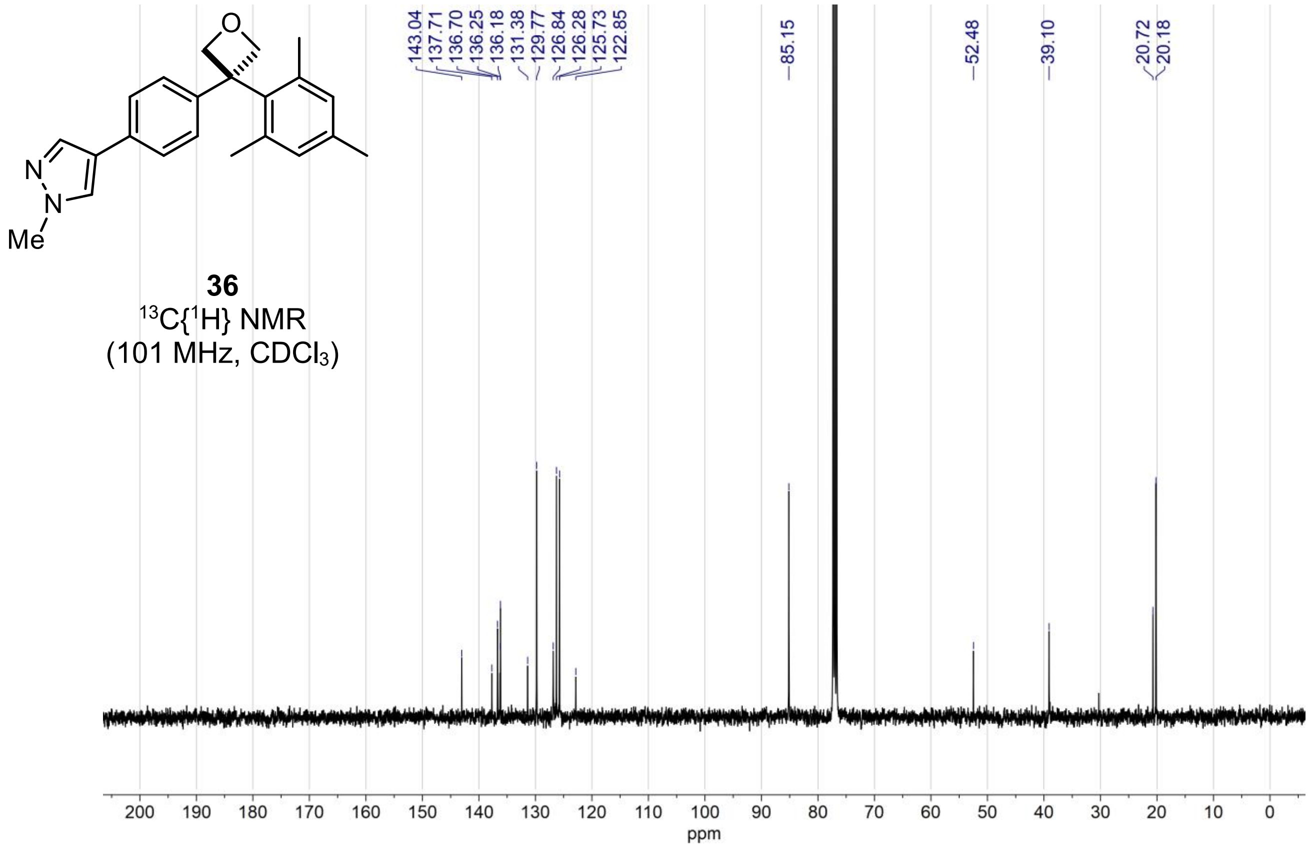

Benzyl 4-(hydroxy(4-(3-mesityloxetan-3-yl)phenyl)methyl)piperidine-1-carboxylate (37)

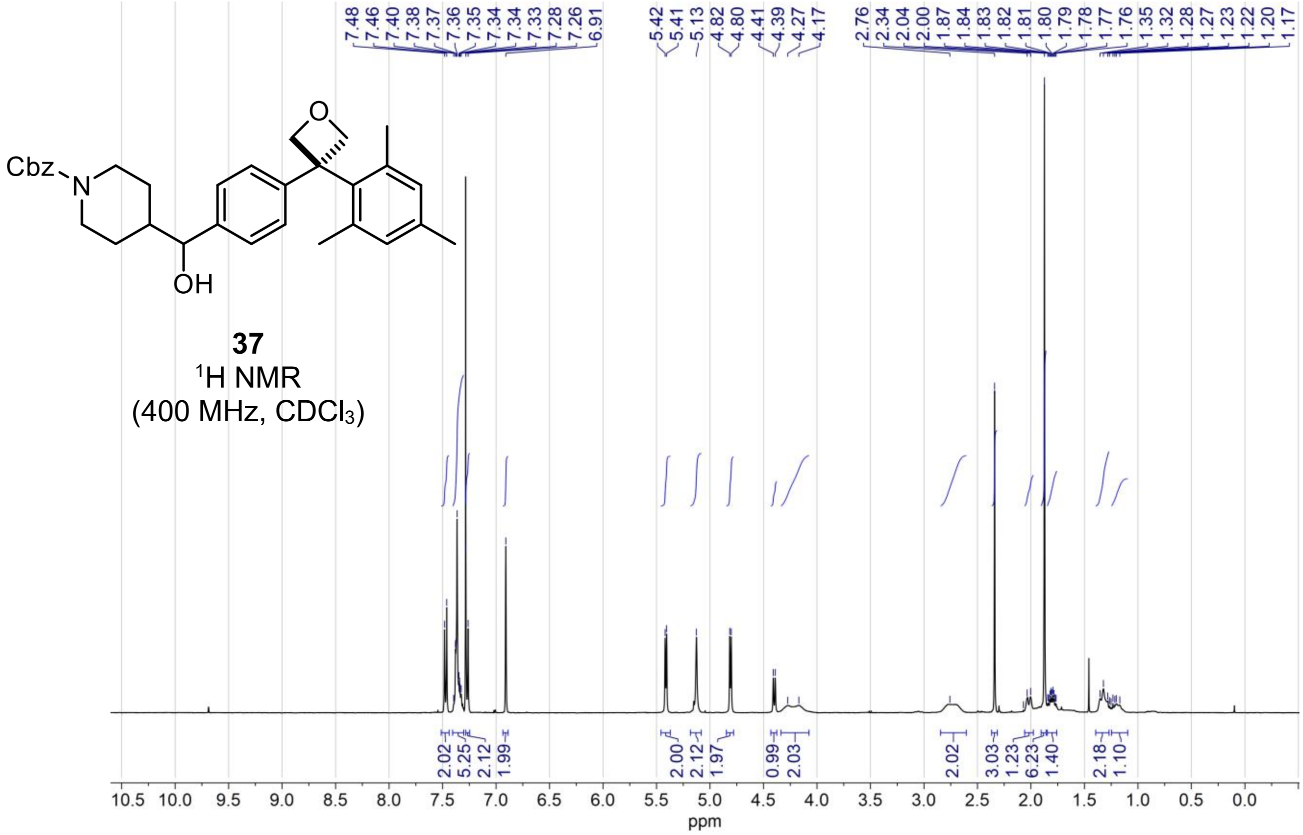

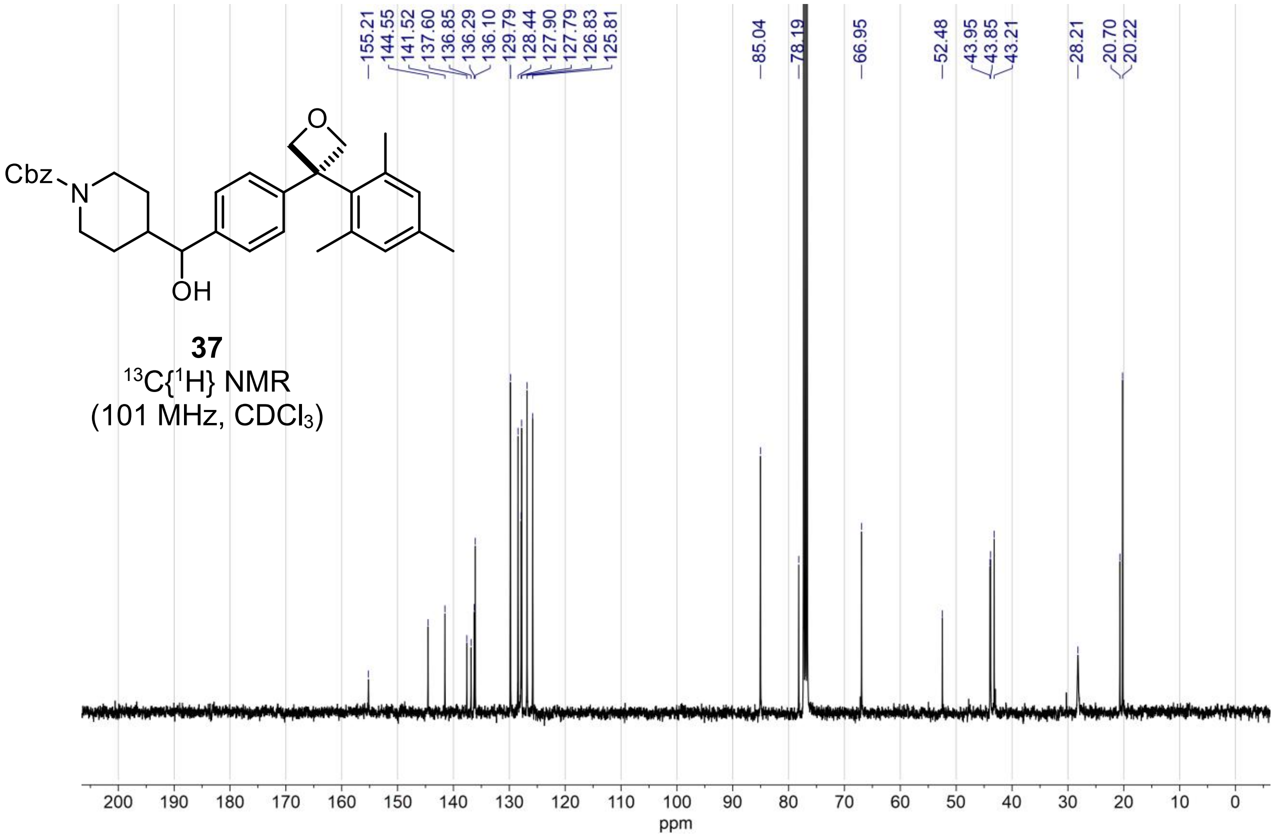

3-Mesityl-3-(4-(*p*-tolylthio)phenyl)oxetane (38)

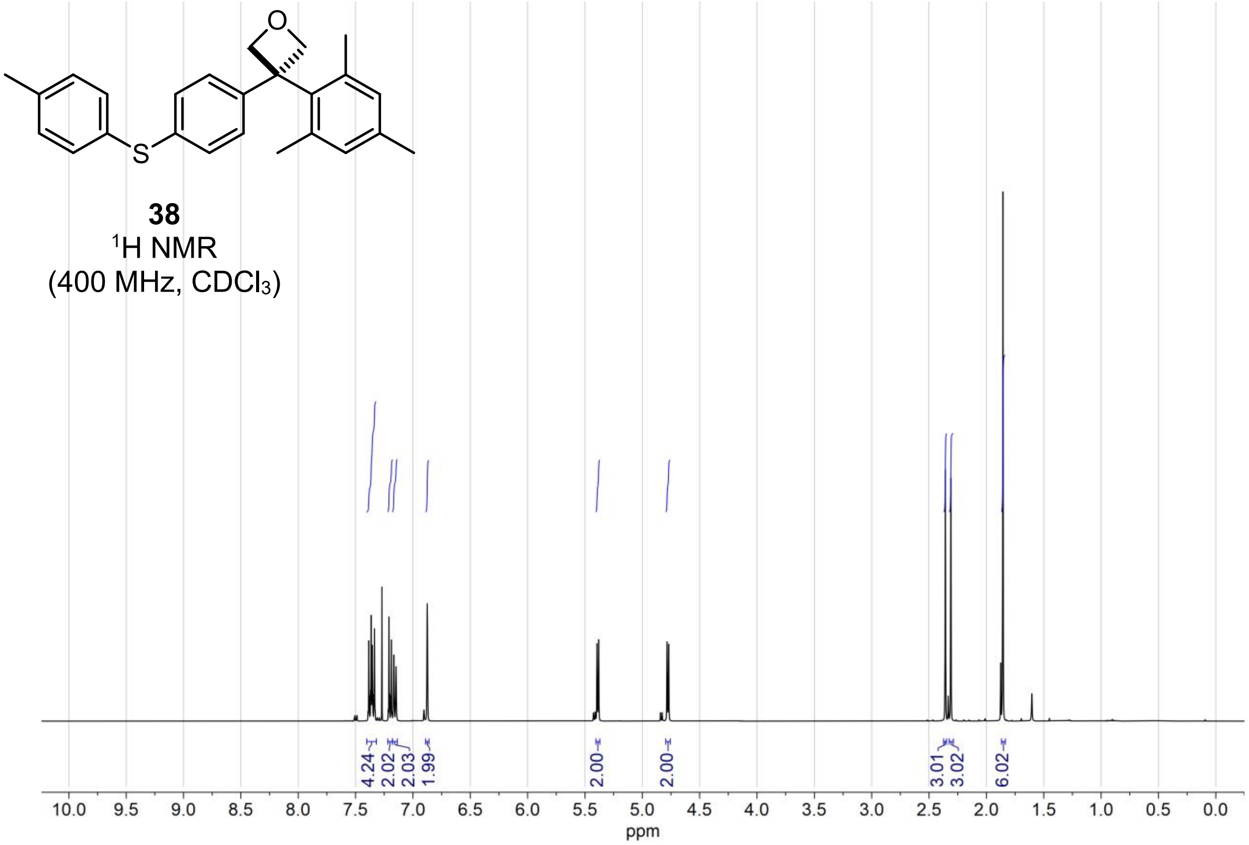

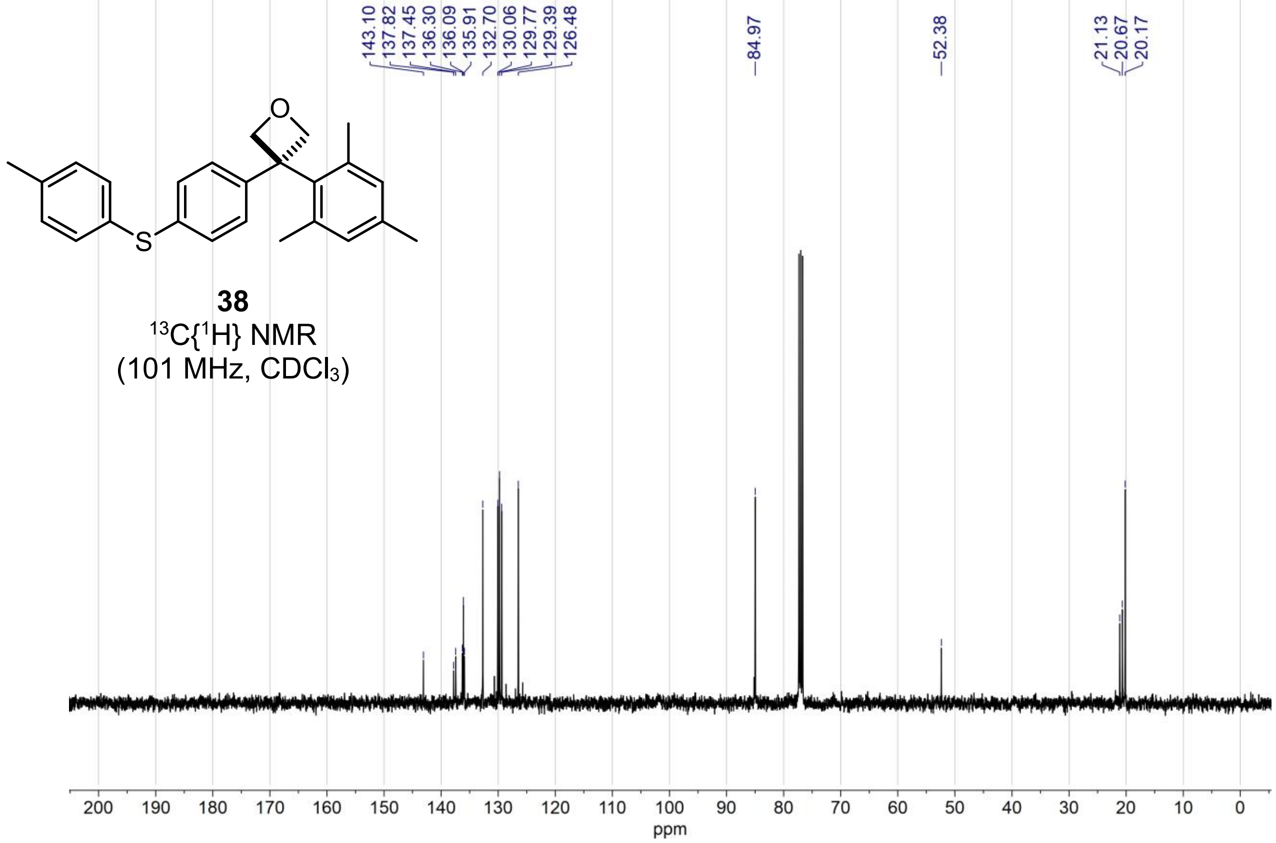

3-((1,1,1,3,3,3-Hexafluoropropan-2-yl)oxy)-3-phenyloxetane (40)

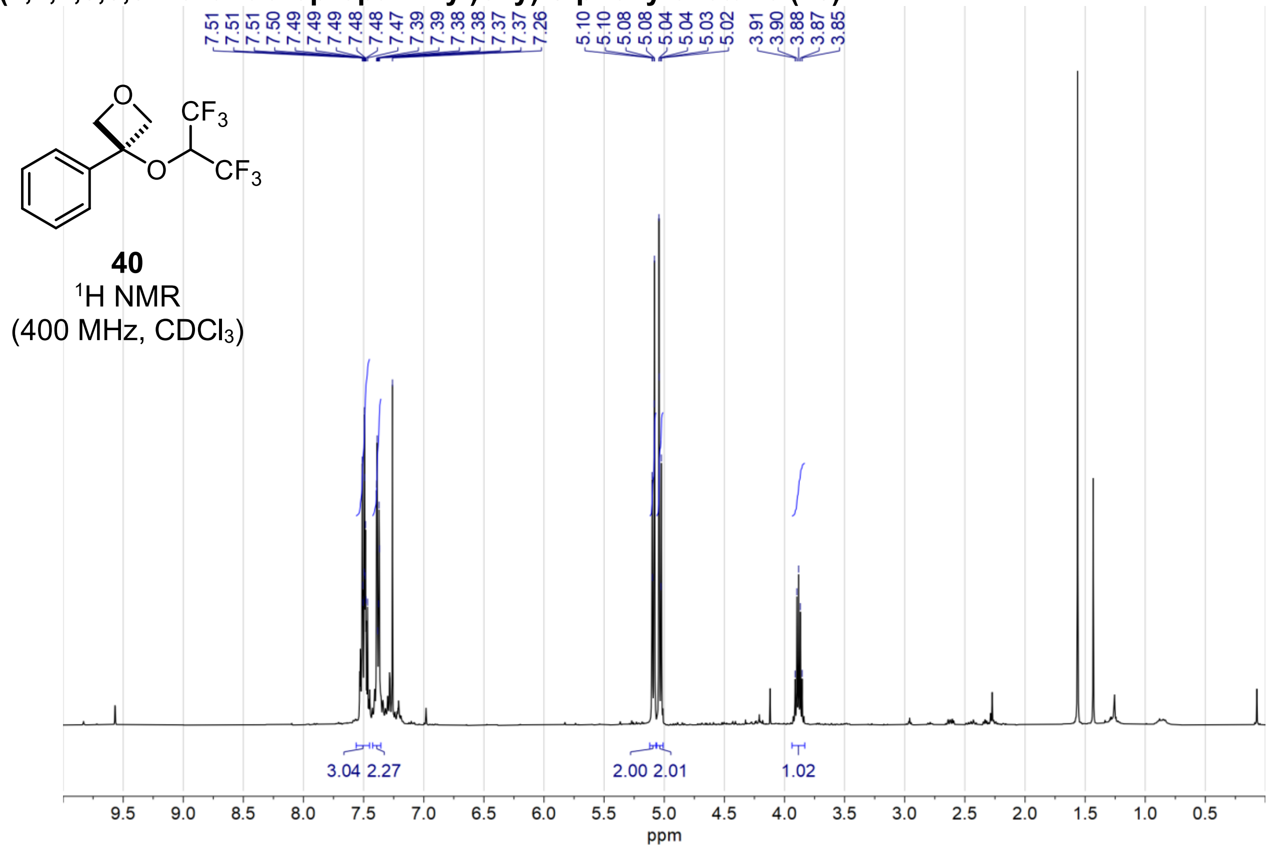

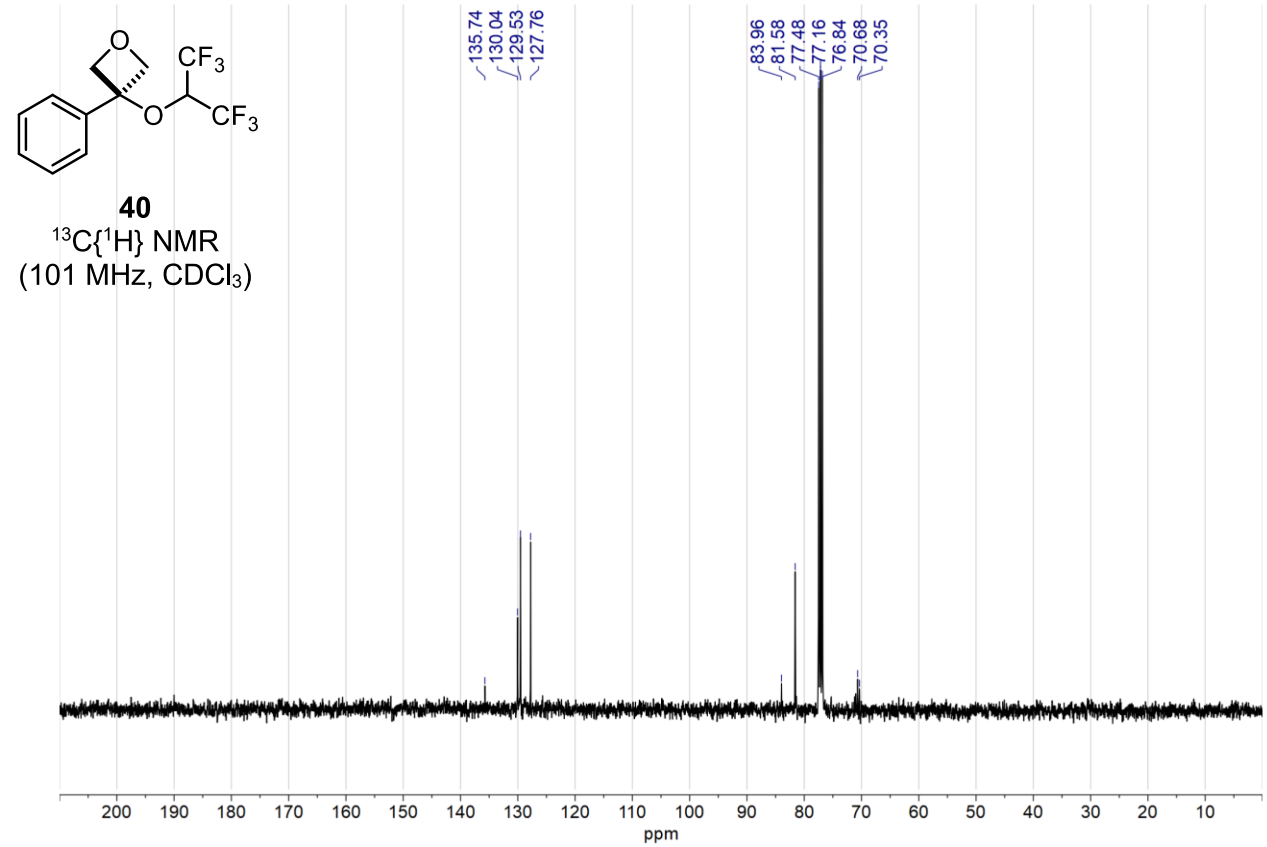

3-Mesityl-2-methylpropanal (41)

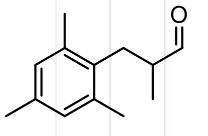

41
^{1}H NMR
(400 MHz, $CDCl_3$)

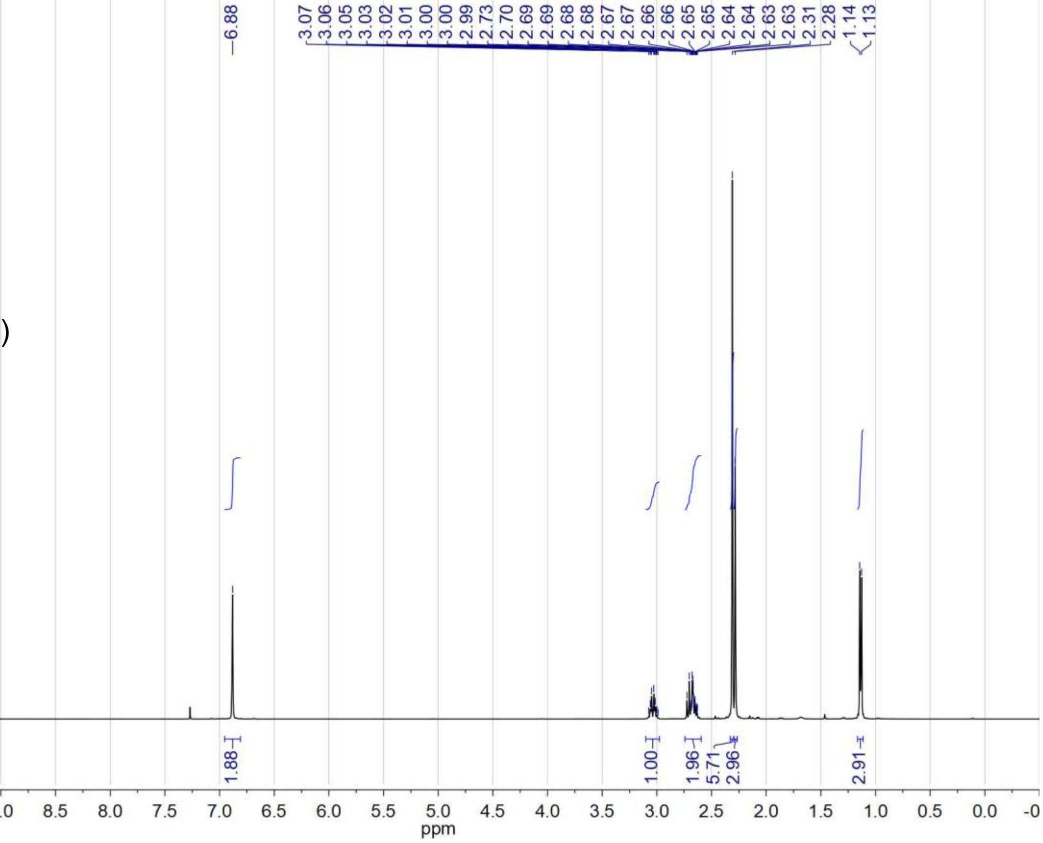

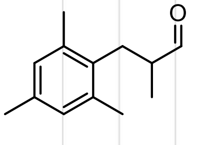

41
^{13}C{^{1}H} NMR
(101 MHz, $CDCl_3$)

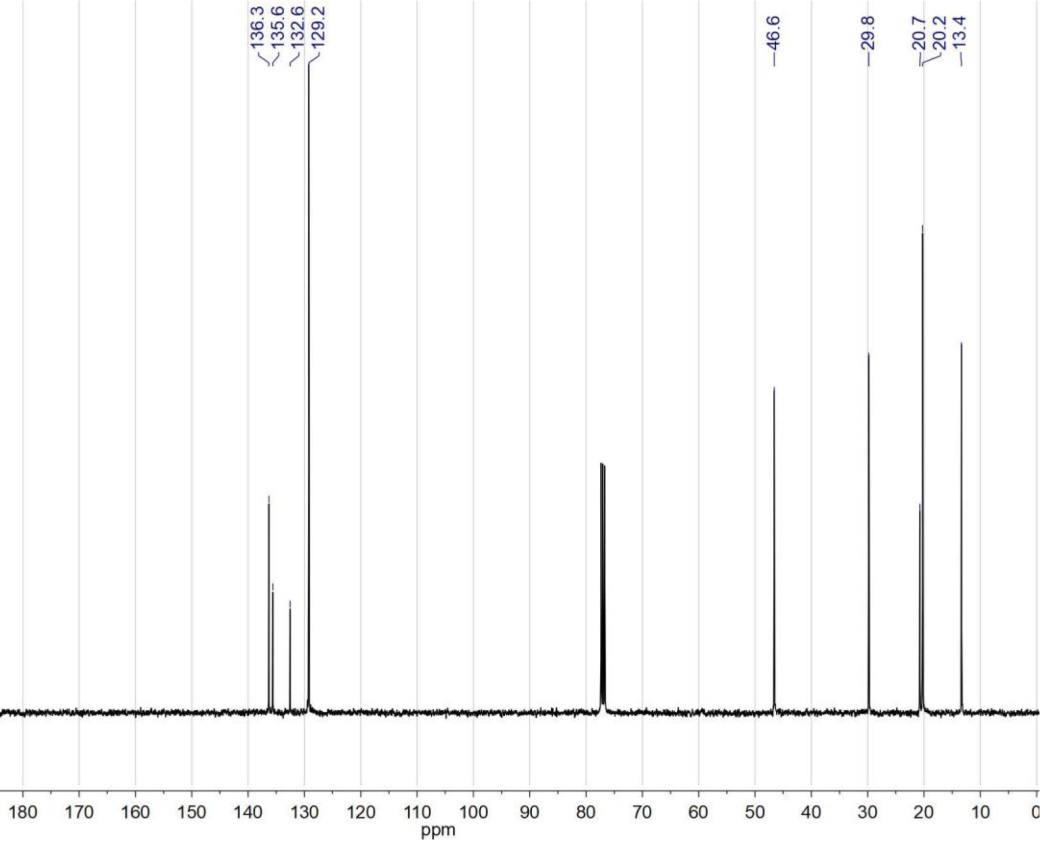

2-Methyl-3-(2,3,5,6-tetramethylphenyl)propanal (42)

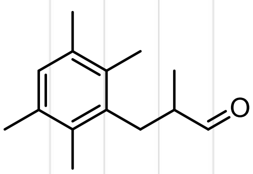

42
^{1}H NMR
(400 MHz, $CDCl_3$)

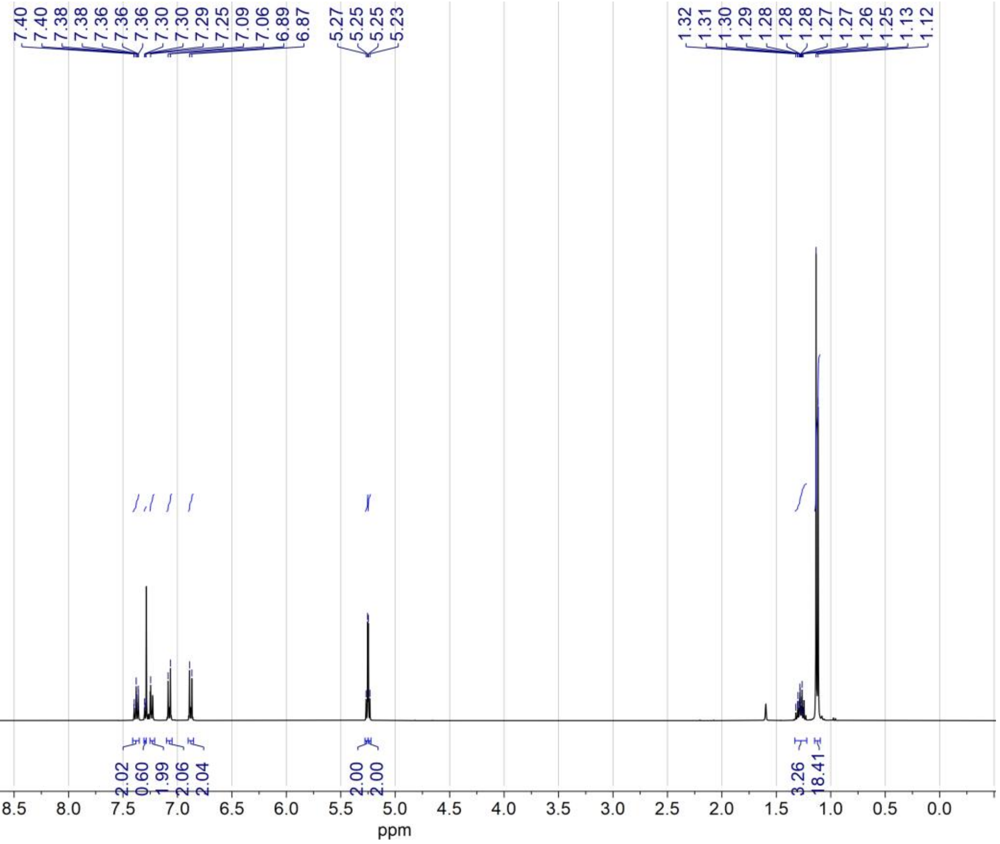

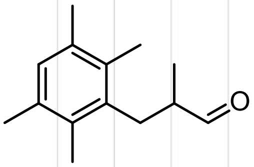

42
$^{13}C\{^1H\}$ NMR
(101 MHz, $CDCl_3$)

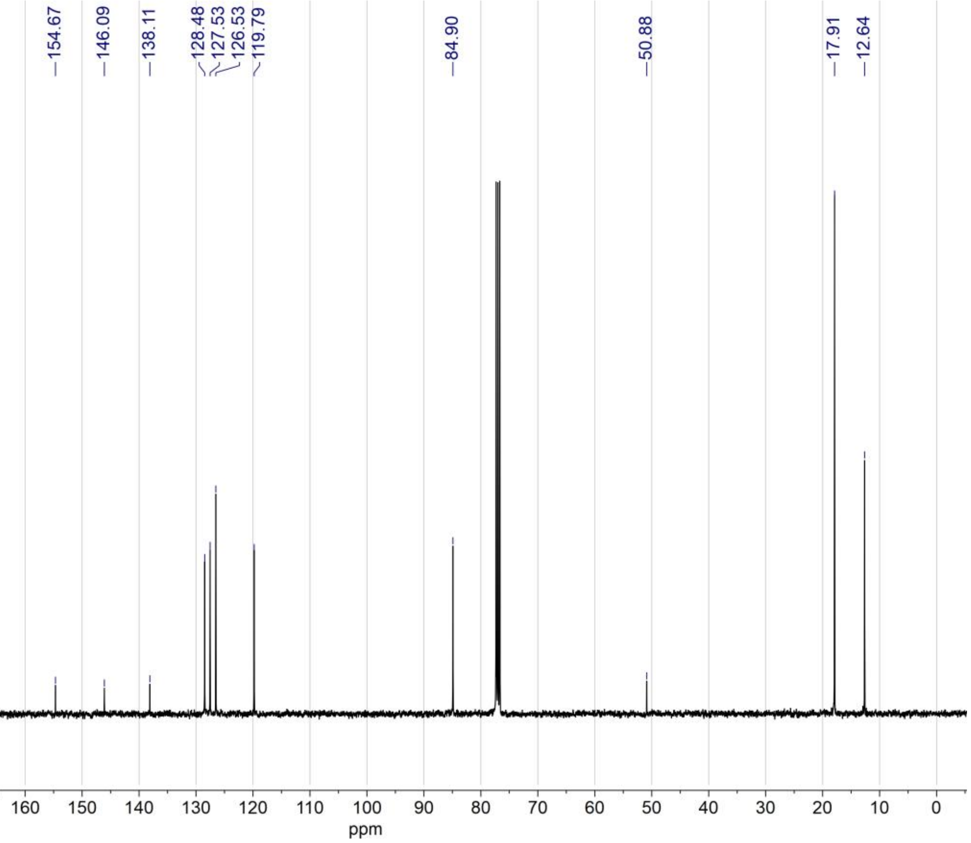

3-Mesitylpropanenitrile (43)

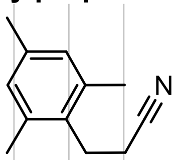

43
^{1}H NMR
(400 MHz, $CDCl_3$)

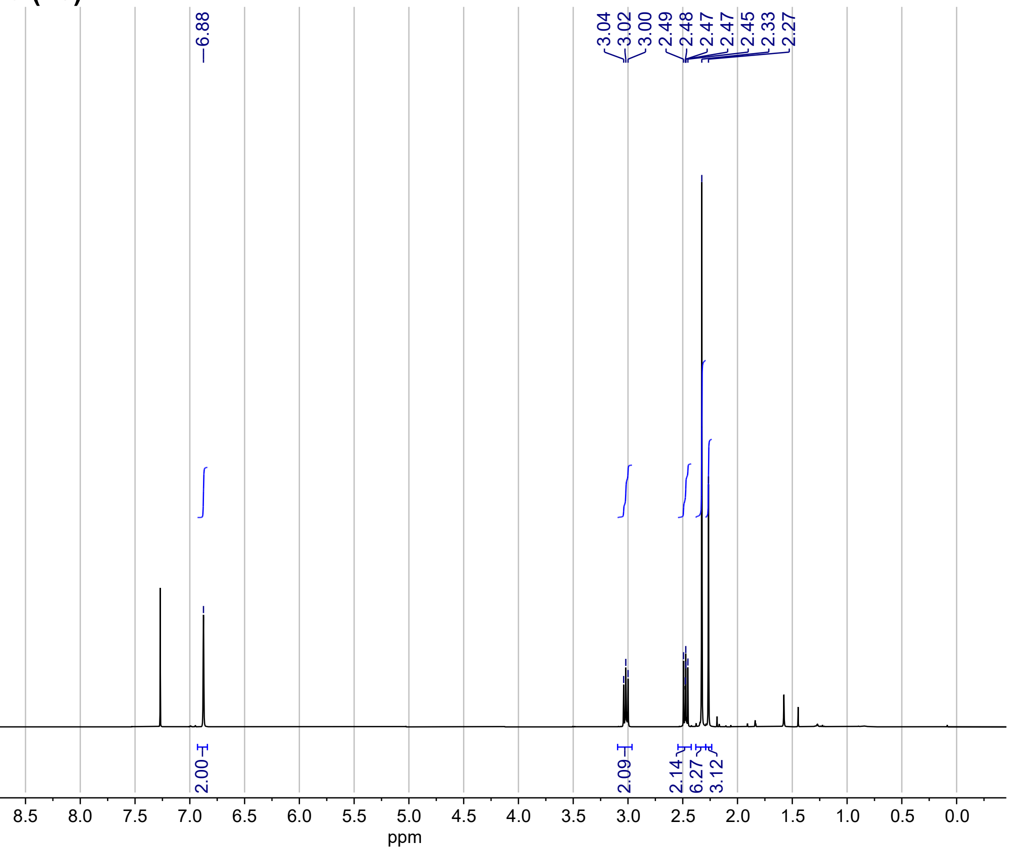

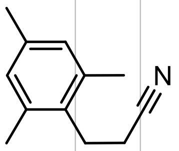

43
^{13}C{^{1}H} NMR
(101 MHz, $CDCl_3$)

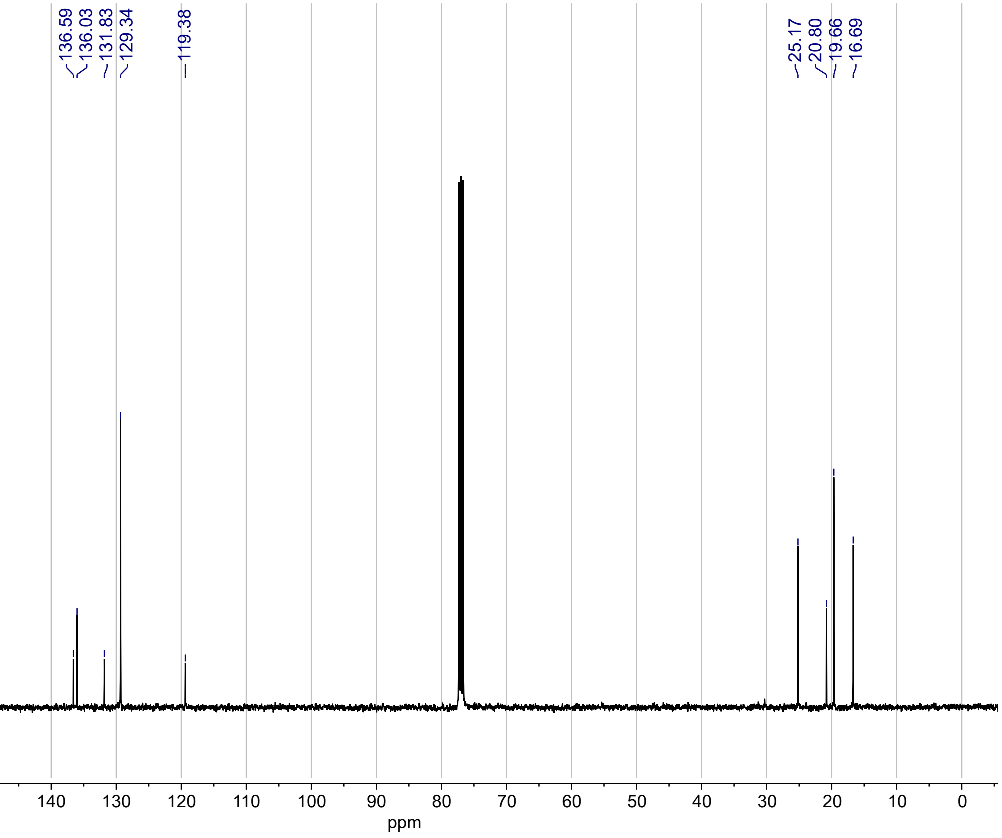

3-(4-Methoxyphenyl)propanenitrile (44)

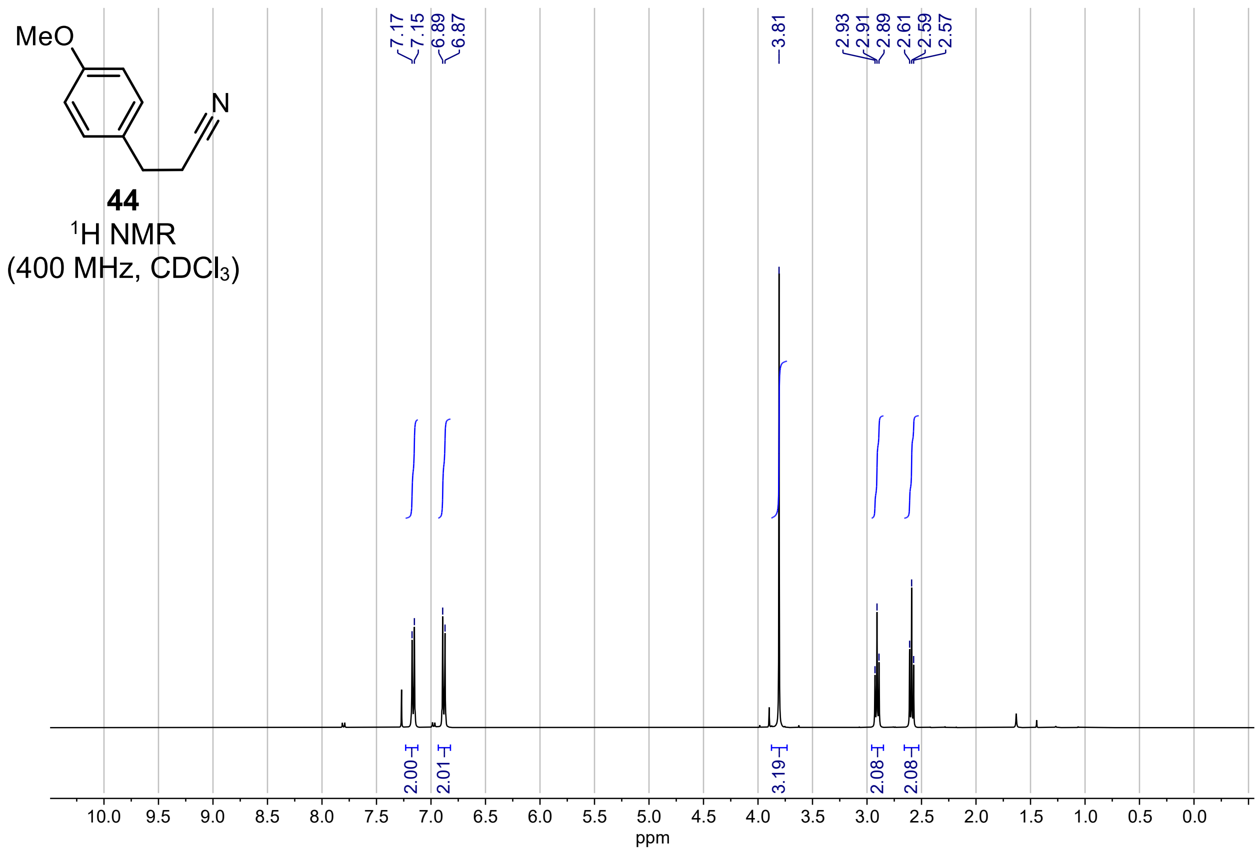

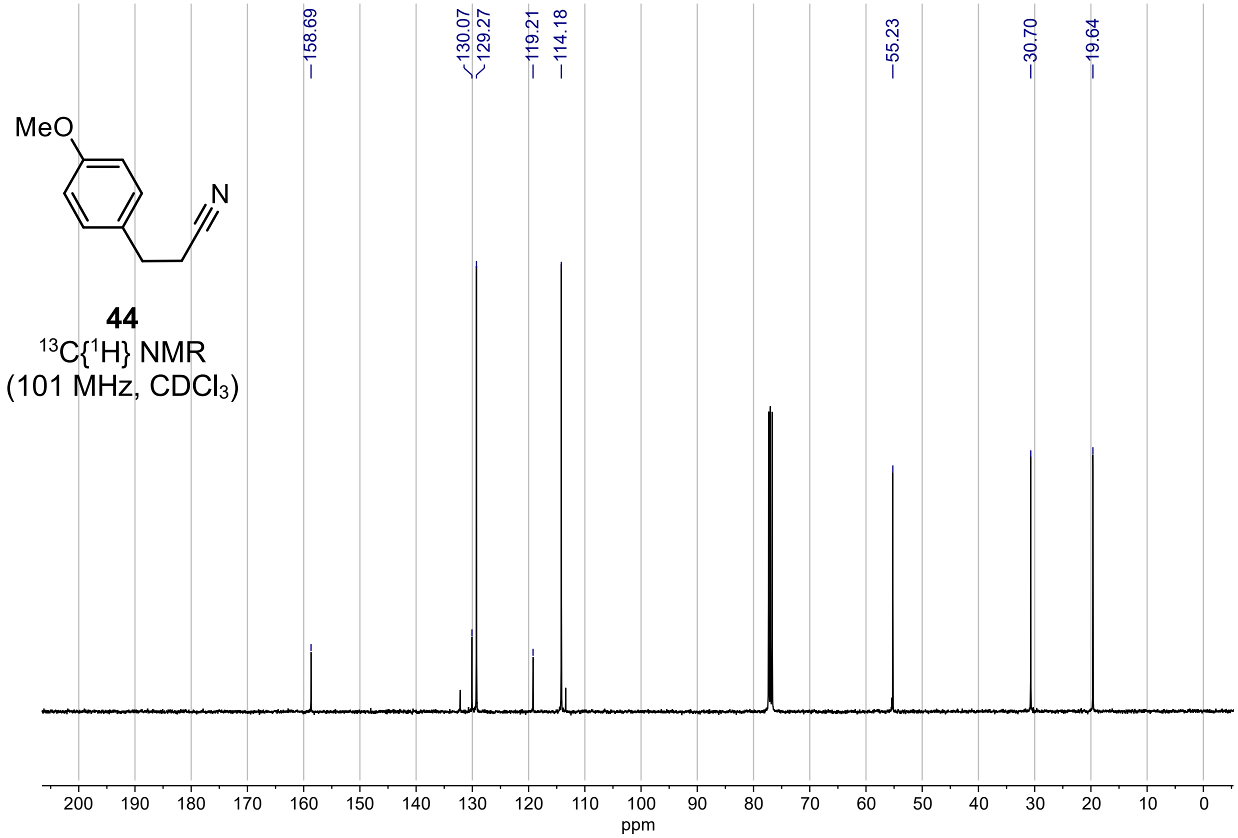

References

1. Hugo E. Gottlieb H. E; Kotlyar V.; Nudelman A.; *J. Org. Chem.* **1997** *62*, 7512-7515.
2. Willot, M.; Chen, J.; Zhu, J. *Synlett* **2009**, 577–580. (b) Mohamadpour, F. 1,1,1,3,3,3-Hexafluoro-2-propanol (HFIP) as a reusable promoting medium for the catalyst-free and green synthesis of dihydropyrano[2,3-c]pyrazole scaffolds. *Results in Chemistry* **2024** 101629.
3. Mo, X.; Yakiwchuk, J.; Dansereau, J.; Adam McCubbin, J.; Hall, D. G. *J. Am. Chem. Soc.* **2015**, *137*, 9694–9703. (b) Vuković, V. D.; Richmond, E.; Wolf, E.; Moran, J. *Angew. Chem. Int. Ed.* **2017**, *56*, 3085–3089. (c) Zhang, S.; Vayer, M.; Noël, F.; Vuković, V. D.; Golushko, A.; Rezajooei, N.; Rowley, C. N.; Lebœuf, D.; Moran, J. Unlocking the Friedel–Crafts Arylation of Primary Aliphatic Alcohols and Epoxides Driven by Hexafluoroisopropanol. *Chem* **2021**, *7*, 3425–3441. (d) Champagne, P. A.; Benhassine, Y.; Desroches, J.; Paquin, J.-F. *Angew. Chem. Int. Ed.* **2014***, 53* (50), 13835–13839. (e) Muller, C.; Horký, F.; Vayer, M.; Golushko, A.; Lebœuf, D.; Moran, J. Synthesis of Functionalised Isochromans: Epoxides as Aldehyde Surrogates in Hexafluoroisopropanol. *Chem. Sci.* **2023**, *14*, 2983–2989. (f) Tang, R.-J.; Milcent, T.; Crousse, B. Friedel–Crafts Alkylation Reaction with Fluorinated Alcohols as Hydrogen-Bond Donors and Solvents. *RSC Adv.* **2018**, *8*, 10314–10317.
4. Dryzhakov, M.; Richmond, E.; Moran, *J. Synthesis* **2016**, *48*, 935–959.
5. Niggemann, M.; Meel, M. J. Calcium-Catalyzed Friedel–Crafts Alkylation at Room Temperature. *Angew. Chem. Int. Ed.* **2010**, *49*, 3684–3687.
6. Brown, H. C.; Okamoto, Y. Electrophilic substituent constants *J. Am. Chem. Soc.* **1958**, *80*, 4979–4987.
7. Croft, Rosemary A.; Mousseau, James J.; Choi, Chulho; Bull, James A. Structurally Divergent Lithium Catalyzed Friedel–Crafts Reactions on Oxetan-3-ols: Synthesis of 3,3-Diaryloxetanes and 2,3-Dihydrobenzofurans. *Eur. J. Org. Chem*. **2016**, 16271–16276.
8. Shen, J.; Wu, G.; Wang, Z.; Fu, Z.; Huang, L. Rhodium-Catalyzed Chemo-Divergent Reaction between Oxetan-3-ones/Azetidin-3-ones and Arylboronic Acids. *J. Org. Chem.* **2025**, *90*, 17190–17197.
9. Surivet, J.-P.; Panchaud, P.; Specklin, J.-L.; Diethelm, S.; Blumstein, A.-C.; Gauvin, J.-C.; Jacob, L.; Masse, F.; Mathieu, G.; Mirre, A.; Schmitt, C.; Lange, R.; Tidten-Luksch, N.; Gnerre, C.; Seeland, S.; Herrmann, C.; Seiler, P.; Enderlin-Paput, M.; Mac Sweeney, A.; Wicki, M.; Hubschwerlen, C.; Ritz, D.; Rueedi, G. Discovery of Novel Inhibitors of LpxC Displaying Potent in Vitro Activity against Gram-Negative Bacteria. *J. Med. Chem.* **2019**, *63*, 66–87.
10. Ren, Z.-G.; Yu, W.-L.; Zheng, H.-X.; Xu, P.-F. PCET-Mediated Ring-Opening alkenylation of cycloalkanols via dual Photoredox and cobalt catalysis. *Org. Lett.* **2022**, *25*, 93–98.
11. Yu, R.; Wang, Y.; Liu, S.; Han, Z.; Sun, J.; Huang, H. Copper-Catalyzed [4+2] annulation of oxetan-3-ols and benzene-1,2-diols for the synthesis of benzodioxanes. *Org. Lett.* **2025**, *27*, 11211–11216.
12. Tian, D.; Chen, G.; Wang, X.; Zhang, H.-J. Modular access to functionalized oxetanes as benzoyl bioisosteres. *J. Am. Chem. Soc.* **2024**, *146*, 18011–18018.
13. Wang, Z.; Chen, Z.; Sun, J. Catalytic enantioselective intermolecular desymmetrization of 3-substituted oxetanes. *Angew. Chem. Int. Ed.* **2013**, 52, 6685–6688.
14. Croft, Rosemary A.; Dubois, Maryne A. J.; Boddy, Alexander J.; Denis, Camille; Lazaridou, Anna; Voisin-Chiret, Anne Sophie; Bureau, Ronan; Choi, Chulho; Mousseau, James J.; Bull, James A. Catalytic Friedel-Crafts Reactions on Saturated Heterocycles and Small Rings for sp^3-sp^2 Coupling of Medicinally Relevant Fragments. *Eur. J. Org. Chem*., **2019**, 5385 – 5395.
15. Dubois, M. A. J.; Smith, M. A.; White, A. J. P.; Jie, A. L. W.; Mousseau, J. J.; Choi, C.; Bull, J. A. Short Synthesis of oxetane and azetidine 3-Aryl-3-carboxylic acid derivatives by selective furan oxidative cleavage. *Org. Lett.* **2020**, *22*, 5279–5283.
16. Dubois, M. A. J.; Smith, M. A.; White, A. J. P.; Lee Wei Jie, A.; Mousseau, J. J.; Choi, C.; Bull, J. A. *Org. Lett.* **2020**, *22*, 5279–5283.
17. Jena, T. K.; Khan, F. A. Direct *α*-Benzylation of Methyl Enol Ethers with Activated Benzyl Alcohols: Its Rearrangement and Access to (±)-Tetrahydronyasol, Propterol A, and 1,3-Diarylpropane. *J. Org. Chem.* **2019**, *84*, 14270–14280.
18. McManus, J. B.; Nicewicz, D. A. Direct C–H cyanation of arenes via organic photoredox catalysis. *J. Am. Chem. Soc.* **2017**, *139*, 2880–2883.
19. Bhunia A.; Bergander K.; Studer A. Cooperative Palladium/Lewis Acid-Catalyzed Transfer Hydrocyanation of Alkenes and Alkynes Using 1-Methylcyclohexa-2,5-diene-1-carbonitrile. *J. Am. Chem. Soc.* **2018**, *140*, 16353–16359.
20. Zhang, Y.; Han, Y.; Zhu, S.; Qing, F.; Xue, X.; Chu, L. Light-Induced divergent cyanation of alkynes enabled by phosphorus radicals. *Angew. Chem. Int. Ed.* **2022**, 61, e202210838.
21. O.V. Dolomanov, L.J. Bourhis, R.J. Gildea, J.A.K. Howard, H. Puschmann, *J. Appl. Cryst.*, 2009, **42**, 339-341.
22. SHELXTL v5.1, Bruker AXS, Madison, WI, 1998.
23. SHELX-2013, G.M. Sheldrick, *Acta Cryst.*, 2015, **C71**, 3-8.
